# A high-quality assembly revealing the *PMEL* gene for the unique plumage phenotype in Liancheng ducks

**DOI:** 10.1093/gigascience/giae114

**Published:** 2025-01-13

**Authors:** Zhen Wang, Zhanbao Guo, Hongfei Liu, Tong Liu, Dapeng Liu, Simeng Yu, Hehe Tang, He Zhang, Qiming Mou, Bo Zhang, Junting Cao, Martine Schroyen, Shuisheng Hou, Zhengkui Zhou

**Affiliations:** State Key Laboratory of Animal Biotech Breeding, Institute of Animal Science, Chinese Academy of Agricultural Sciences, Beijing 100193, China; Precision Livestock and Nutrition Unit, Gembloux Agro-Bio Tech, TERRA Teaching and Research Centre, University of Liège, Gembloux 5030, Belgium; State Key Laboratory of Animal Biotech Breeding, Institute of Animal Science, Chinese Academy of Agricultural Sciences, Beijing 100193, China; State Key Laboratory of Animal Biotech Breeding, Institute of Animal Science, Chinese Academy of Agricultural Sciences, Beijing 100193, China; State Key Laboratory of Animal Biotech Breeding, Institute of Animal Science, Chinese Academy of Agricultural Sciences, Beijing 100193, China; State Key Laboratory of Animal Biotech Breeding, Institute of Animal Science, Chinese Academy of Agricultural Sciences, Beijing 100193, China; State Key Laboratory of Animal Biotech Breeding, Institute of Animal Science, Chinese Academy of Agricultural Sciences, Beijing 100193, China; State Key Laboratory of Animal Biotech Breeding, Institute of Animal Science, Chinese Academy of Agricultural Sciences, Beijing 100193, China; State Key Laboratory of Animal Biotech Breeding, Institute of Animal Science, Chinese Academy of Agricultural Sciences, Beijing 100193, China; State Key Laboratory of Animal Biotech Breeding, Institute of Animal Science, Chinese Academy of Agricultural Sciences, Beijing 100193, China; State Key Laboratory of Animal Biotech Breeding, Institute of Animal Science, Chinese Academy of Agricultural Sciences, Beijing 100193, China; State Key Laboratory of Animal Biotech Breeding, Institute of Animal Science, Chinese Academy of Agricultural Sciences, Beijing 100193, China; Precision Livestock and Nutrition Unit, Gembloux Agro-Bio Tech, TERRA Teaching and Research Centre, University of Liège, Gembloux 5030, Belgium; State Key Laboratory of Animal Biotech Breeding, Institute of Animal Science, Chinese Academy of Agricultural Sciences, Beijing 100193, China; State Key Laboratory of Animal Biotech Breeding, Institute of Animal Science, Chinese Academy of Agricultural Sciences, Beijing 100193, China

**Keywords:** duck, genome assembly, plumage color, *PMEL*, melanin

## Abstract

**Background:**

Plumage coloration is a distinctive trait in ducks, and the Liancheng duck, characterized by its white plumage and black beak and webbed feet, serves as an excellent subject for such studies. However, academic comprehension of the genetic mechanisms underlying duck plumage coloration remains limited. To this end, the Liancheng duck genome (GCA_039998735.1) was hereby *de novo* assembled using HiFi reads, and F2 segregating populations were generated from Liancheng and Pekin ducks. The aim was to identify the genetic mechanism of white plumage in Liancheng ducks.

**Results:**

In this study, 1.29 Gb Liancheng duck genome was *de novo* assembled, involving a contig N50 of 12.17 Mb and a scaffold N50 of 83.98 Mb. Beyond the epistatic effect of the *MITF* gene, genome-wide association study analysis pinpointed a 0.8-Mb genomic region encompassing the *PMEL* gene. This gene encoded a protein specific to pigment cells and was essential for the formation of fibrillar sheets within melanosomes, the organelles responsible for pigmentation. Additionally, linkage disequilibrium analysis revealed 2 candidate single-nucleotide polymorphisms (Chr33: 5,303,994A>G; 5,303,997A>G) that might alter *PMEL* transcription, potentially influencing plumage coloration in Liancheng ducks.

**Conclusions:**

Our study has assembled a high-quality genome for the Liancheng duck and has presented compelling evidence that the white plumage characteristic of this breed is attributable to the *PMEL* gene. Overall, these findings offer significant insights and direction for future studies and breeding programs aimed at understanding and manipulating avian plumage coloration.

## Background

Plumage coloration, a diverse and conspicuous trait among bird species, is a valuable attribute for investigating natural and artificial selection. Melanin, the predominant pigment in avian plumage, occurs as a mix of eumelanin and phaeomelanin across various tissues, leading to the extensive color variation observed in birds [1, 2]. Eumelanin deposition in plumage is responsible for black and brown coloration, constituting the predominant pigment in bird feathers [3]. Extensive research on eumelanin- and melanin-related genes has significantly enhanced public understanding of avian plumage coloration, which is a captivating ornamental feature. The duck (*Anas platyrhynchos*) (NCBI: txid8839), widely distributed globally, displays a continuum of plumage colors spanning white to black, possibly as an adaptation to varied ecological environments. Those diverse plumage color patterns make ducks a key animal model for pigmentation studies. Despite advancements in understanding the biological and evolutionary aspects of plumage coloration, the genetic basis of these colors in ducks remains poorly understood.

The Liancheng (LC) duck possesses a distinctive phenotype marked by white feathers, black beak, and black webbed feet. It is recognized for its significant melanin deposition in the beak and webbed feet, primarily due to the involvement of eumelanin as the main pigment [4]. The biosynthesis of eumelanin is a complex process that involves 3 critical steps: initially, tyrosinase catalyzes the oxidation of tyrosine to dihydroxyphenylalanine (DOPA). Subsequently, an oxidase enzyme converts DOPA into dopaquinone. Ultimately, dopaquinone undergoes a series of cyclic transformations, resulting in the production of pigment and the formation of melanin [5]. This synthetic pathway holds much significance in eumelanin synthesis, particularly in the pigmentation of skin and feathers in ducks. In this process, *MITF* serves as a key target of various signal transduction pathways, also the main regulator of melanin production. However, the genetic basis of melanin deposition and the specific genes involved in the formation of white feathers in LC ducks remain to be further explored.

The initial draft of the duck assembly was first reported in 2013 [6]. Refinements to the Pekin (PK) duck genome have achieved a scaffold N50 length of up to 76.3 Mb by 2020, which now serves as a foundational resource for duck genome analysis [7]. The high quality and variety of the genome assemblies of other birds, especially the one for chickens, necessitate further improvements in duck assembly quality. Previous research on animal breeding and genetics in ducks has primarily focused on meat quality [8], adipose tissue deposition [9], and muscle weight [10]. However, genetic mechanisms of plumage coloration remain largely unexplored, and no reference genome of the famous LC duck has been published, making an upgraded LC duck genome necessarily important to provide foundational data for relevant future studies. The accuracy of gene localization greatly depends on the quality of the genome assembly [9]. A recent study leveraging the widely utilized duck genome assembly (GCA_003850225.1) has pinpointed a 6.6-kb intronic insertion within *MITF*. This insertion is suspected to influence splicing events, consequently resulting in the white feather phenotype observed in ducks [11]. Additionally, another study has uncovered 4 new single-nucleotide polymorphisms (SNPs) in the *MC1R* regulator region associated with black plumage in ducks [12]. This partially explains the mechanism of melanin formation in duck feathers. However, questions regarding feather coloration remain unanswered. Long-read sequencing technology, also known as third-generation sequencing, is capable of generating reads exceeding 10 kb in length. This capability allows it to span highly repetitive genomic regions and resolve assembly gaps previously intractable, thereby enhancing the continuity of genomic assemblies. As an alternative to relying on short-read data polishing, PacBio introduces high-fidelity reads (HiFi reads), which can provide more accurate, continuous, and complete genetic information. It has thus become a key technology for research [13]. Meanwhile, advancements in gene chips and genome resequencing technologies have made genome-wide association studies (GWAS) powerful tools for identifying genetic variations linked to phenotypes. GWAS analysis has uncovered mutations in the *MuPKS* gene responsible for yellow and blue plumage in parrots [14]. Additionally, GWAS has localized a nonsense mutation (W49X) in the *SLC2A11B* gene, which is associated with the white eye trait in pigeons [15]. To this end, the presence of the *PMEL* gene, which was previously considered “missing” gene in Pekin ducks, was hereby explored based on a high-quality *de novo* genome by HiFi sequencing. Building on previous findings, this research identified 2 closely linked SNPs in the regulatory region that might influence *PMEL* transcription, leading to the white plumage observed in LC ducks. Overall, this investigation provides a valuable genome assembly, molecular markers for duck breeding, and offers insights into plumage color patterns in avian species.

## Data Description

To understand the genetic basis of the unique plumage phenotype of Liancheng ducks, a high-quality *de novo* assembly of the Liancheng duck genome (GCA_039998735.1) was hereby utilized, and 4 different plumage color phenotypes were collected from a crossbreeding population involving LC and PK ducks. Meanwhile, whole-genome resequencing of 366 ducks was performed, and the resequencing data were aligned to our assembled Liancheng duck reference genome for variation calling and subsequently GWAS analysis. In the present study, the *PMEL* gene associated with plumage coloration in Liancheng ducks was successfully identified through transcriptome sequencing analysis across black- and white-feathered ducks and 9 duck tissues, and the genetic mechanisms underlying the formation of duck plumage coloration were evidently elucidated.

## Materials and Methods

### Ducks and sampling

Herein, all animal procedures adhered to the guidelines for the care and use of experimental animals set by the Chinese Academy of Agricultural Sciences (IAS2022-105). The study was approved by the Ethics Committee of the Chinese Academy of Agricultural Sciences. A blood sample was obtained from a female Liancheng duck for the purpose of conducting a *de novo* genome assembly. A total of 366 parental and intercross population duck plumage color phenotypes were recorded from a previous gradient consanguinity population [6]. These included 117 PK ducks, 59 LC ducks, 38 white-feathered ducks with yellow beaks (WY), 42 white-feathered ducks with black beaks (WB), 67 gray-feathered ducks with black beaks (GF), and 41 black-feathered ducks with black beaks (BF). All underwent whole-genome resequencing (Supplementary Table S1). Additionally, genome data from 23 black-feathered ducks, comprising 20 Mallards (MD) and 3 Putian (PT) ducks, were used for comparative analysis [2].

For transcriptomics analysis, skin tissues from LC ducks were collected at embryonic stages of 12 days, 15 days, 20 days, 28 days, and 1 week after birth. Each sample group, except for the four 12-day embryo samples, consisted of 3 biological replicates. Additionally, tissues from an 8-week-old black-feathered MD duck, including heart, fat, muscle, brain, spleen, lung, liver, kidney, and skin, were collected, with 1 replicate per tissue (Supplementary Table S2) [2]. Feather bulb specimens were also collected from feather follicle from 1-week-old black-feathered MD ducks, LC ducks, PK ducks, and WB ducks, with 3 biological replicates in each sample group [16, 17]. A total of 37 samples were used for RNA sequencing (RNA-seq) (Supplementary Table S2). For the immunofluorescence assay, skins containing hair follicles from three 1-week-old MD ducks and 3 LC ducks were collected for protein analysis.

### Genome assembly and gene annotation

A combination of PacBio long-read HiFi sequencing and High-through chromosome conformation capture (Hi-C) technologies was hereby utilized to conduct the *de novo* assembly of the Liancheng duck genome. Long-read and long-HiFi (RRID:SCR_021966) sequencing data (PacBio) were used for the species assembly [18]. Sequencing was performed on the PacBio Sequel II platform, and genome assembly was conducted using the hifiasm versions (v0.19.3 NOV-2023) for contig generation. The 3D-DNA pipeline (RRID:SCR_017227) (NOV-2023) was utilized for manual curation and to orient the genome sequences based on the Hi-C contact map [19–21]. Ultimately, BUSCO was used to evaluate the completeness and quality of Liancheng duck genome assembly (IASCAAS_LianchengWhiteDuck, GCA_039998735.1) [22].

Protein-coding genes were predicted via the combination of evidence-based prediction and *de novo* prediction. RNA-seq data were used for evidence-based annotation through Maker2 (Version 2.31.10) (i.e., a powerful open-source genome annotation tool). RNA-seq data were aligned against the genome using Program to Assemble Spliced Alignments (PASA) (RRID:SCR_014656) to construct a training model for *Augustus* (RRID:SCR_008417), a tool designed to predict genes in eukaryotic genomic sequences. Subsequently, *de novo* annotation of the genome was performed utilizing *Augustus. De novo* genome assembly refers to a method of piecing together a complete sequence of a new genome, rather than comparing it based on a known genome. Subsequently, the results of the RNA-seq–based annotation and the *de novo–*based annotation were integrated on the principle that the evidence results were better than the prediction ones. In the final step, genes were filtered to retain those with less than 50% repeated sequences, encoding proteins longer than 50 amino acids, and having at least 1 count of expression. Functional annotation of genes was performed using eggNOG software (RRID:SCR_002456) [23].

### Whole genome resequencing

A total of 366 samples, consisting of Pekin ducks, Liancheng ducks, and intercross populations, were hereby selected for resequencing (Supplementary Table S1). The genome data of 23 black-feathered ducks included 20 Mallards and 3 Putian ducks. DNA-eligible samples were identified for further testing. Libraries were established, involving an average read length of 150 bp for all samples. Subsequently, they were sequenced on an Illumina HiSeq X-Ten platform, and an average raw read sequence coverage of 5× was yielded. This 5× coverage ensured the accuracy of variant calling and genotyping when linking back to the Liancheng duck reference genome (IASCAAS_LianchengWhiteDuck, GCA_039998735.1) [6]. Following the elimination of read pairs containing adapter sequences, a quality assessment of the raw reads was performed using TRIMMOMATIC (RRID:SCR_011848) (version 0.36) and Cutadapt [24, 25]. Following that, the high-quality reads were aligned to the Liancheng duck reference genome using the Burrows–Wheeler aligner (BWA-aln) with parameter “bwa aln genome.fa sample.fastq > sample.sai, bwa sampe genome.fa sample.sai sample.fastq > sample.sam” [26]. Besides, genetic variants were identified from the sequencing data in this study using the Genome Analysis Toolkit (GATK) [27]. SNPs underwent filtration based on the following criteria: (i) SNPs with minor allele frequency (MAF) >0.05, (ii) the maximum missing rate per SNP set at <0.7, and (iii) SNPs restricted to possessing only 2 alleles.

### Genome-wide association analysis

The GWAS was conducted utilizing a mixed linear model implemented through the EMMAX program (RRID:SCR_024012) [28] with genome-wide SNP data and the plumage color phenotype observed in 366 individuals from the resequencing population. The analytical model adopted the form *y* = Xb + Ga + e, with *y* representing the phenotypic value (plumage color of each duck), X denoting the matrix corresponding to fixed effects, and b signifying the magnitude of the fixed effects. The fixed effects included sex-related influences. G represents the genetic matrix associated with population kinship, while e stands for the random residual. Furthermore, principal component analysis (PCA) was executed using all SNPs, with the top 3 components incorporated as fixed effects within the mixed model to adjust for population stratification. A Bonferroni correction threshold of 0.01/N (−log_10_P = 8.95) was established to pinpoint significant sites in the GWAS findings [2, 8, 29], where N indicates the total number of whole genome SNPs (8,887,194). In addition, fine-mapped analyses were conducted in 328 ducks via identity by descent (IBD) analysis, for the efficiency of Identity By Descent (IBD) fragments in reflecting the genetic relationship between individuals and detecting trait variation. The correlation between IBD fragments and phenotype was used to identify regions affecting trait variation in the genome [28, 30]. For this analysis, the filtered SNPs (*n* = 117) met the standard allele frequency difference (ΔAF) >0.8 between the Liancheng ducks and Pekin ducks. In the candidate region (Chr33: 5.1–5.5 Mb), 4 recombination breakpoints were identified across the 36 SNPs, and the segregating individuals were subsequently classified using these 4 recombinant breakpoints.

### Transcriptome sequencing and analysis

Skin tissues from LC ducks were collected at embryonic stages of 12 days, 15 days, 20 days, 28 days, and 1 week after birth. Additionally, tissues including heart, fat, muscle, brain, spleen, lung, liver, kidney, and skin were collected from an 8-week-old black-feathered MD duck. Feather bulb specimens were also obtained from feather follicles of 1-week-old black-feathered MD ducks, LC ducks, PK ducks, and WB ducks. The total RNA was initially extracted from the above tissues using Trizol reagent (Vazyme). The RNase enzyme was inactivated by adding pyrrole diethyl carbonate. Final RNA-seq libraries were prepared for the experiment and sequenced on an Illumina platform using the 150-bp paired-end sequencing module. The effective read length was increased by Illumina sequencing, yielding an average production of 6 Gb per library. Using TopHat (RRID:SCR_013035), RNA-seq paired-end reads from each library were mapped to the aforementioned reference genome of the Liancheng duck. Besides, the expression was calculated by using TopHat, and read counts per million (CPM) values for the genes were obtained running htseq-count in the Figshare database (10.6084/m9.figshare.27311937) [6, 31].

### Quantitative PCR analysis on *PMEL* in feather bulb specimens

Complementary DNA (DNA) from feather bulb specimens, including those of black-feathered, gray-feathered, Liancheng, and Pekin ducks, was reversely transcribed using HiScript III All-in-One RT SuperMix Perfect for qPCR (Vazyme). The quantitative PCR (qPCR) was conducted in a total volume of 10 μL, which included 5 μL Tap Pro Universal SYBR qPCR Master Mix (Vazyme), 0.8 μL forward and reverse primers, 0.5 μL cDNA, and 3.7 μL distilled water. β-Actin was selected as the internal reference gene. The primer sequence is shown in Supplementary Table S3. All reactions were run in triplicate. The relative messenger RNA (mRNA) expression levels were calculated using the normalized relative quantification method, followed by the 2−^ΔΔCT^ calculation [32].

### Immunofluorescence experiment

The skin samples of ducks with black and white feathers were embedded in paraffin, fixed in 4% buffered paraformaldehyde, and sectioned into 5-µm slices. Upon overnight fixation at 4°C, the duration was kept within 24 hours to ensure effective preservation of tissue integrity. Subsequently, the sections were dewaxed and rehydrated to enhance adhesion and facilitate the dewaxing process. Antigen retrieval was performed by incubating the sections in EDTA (Servicebio) at 100°C for 20 minutes. Following retrieval, antigens were fixed with Tris-EDTA, and sections were washed thrice in phosphate-buffered saline (PBS), followed by a further rinse. For immunostaining, sections were incubated with a PMEL antibody (ABclonal) at 4°C for 12 hours after pretreatment with 3% bovine serum albumin (Solarbio) for 30 minutes. The PMEL antibody, an anti-rabbit species, was hereby utilized for immunodetection. Ultimately, feather follicle tissues displaying diverse plumage colors were counterstained with DAPI.

### Causative mutation screening and identification

The candidate regions (Chr33: 5.24–5.32 Mb) among 117 Pekin ducks, 59 Liancheng ducks, and 152 intercross population ducks (excepting 38 WY ducks) were compared based on the Liancheng duck genome. Among the candidate IBD fragments, only regions featuring consistent genotypes and phenotypes were further investigated as *Rr* candidate regions. To eliminate variations with a lower likelihood of being causally involved, the following 3-step procedures were conducted: first, the genotype and phenotype information from the 328 parents and intercross ducks was utilized to exclude SNPs based on the standard *F*_ST_ < 0.8 (LC vs. PK duck). Second, information from 117 Pekin ducks, 59 Liancheng ducks, 20 Mallards, 42 WB ducks, 43 BF ducks, and 3 Putian ducks was applied. Candidate regions were selected based on the highest *F*_ST_ values shared between Liancheng ducks and other breeds. Third, only genotypes that were completely concordant with the phenotypes from the Mallard and Putian duck populations were retained as candidate causative mutations. Subsequently, all indels within the candidate regions were excluded using the method described above. Ultimately, only 2 SNP variations that showed genotype–phenotype concordance across multiple duck breeds were considered causative variations for the *Rr* locus.

### Hi-C sequencing and analysis

Skin fat tissue samples from a Liancheng duck were subjected to cross-linking in 20 mL of fresh ice-cold nuclear isolation buffer. The chromatin extraction methodology adhered to previous protocols [2]. Subsequently, the purified DNA underwent digestion and fragmentation using the *DpnII* restriction enzyme, followed by a repair of the DNA ends, and biotin-labeled DNA fragments were then isolated using streptavidin C1 beads. Afterward, library preparation was conducted utilizing an Illumina TruSeq DNA Sample Prep Kit following the manufacturer’s guidelines. Quality assessment of the Hi-C library was performed through TaqMan Arbitration (TA) cloning, and the Hi-C libraries were sequenced on an Illumina HiSeq X Ten system. The Hi-C experiments were performed independently on 2 occasions, with the experimental and sequencing procedures executed by Gene Technology Co., Ltd.

Raw Hi-C data were processed to eliminate low-quality reads and trim adapters using TRIMMOMATIC (RRID:SCR_011848) [24]. All reads were trimmed to 150 bp, and clean reads were aligned to the duck genome using a 2-step approach integrated into the HiC-Pro (RRID:SCR_017643) software [33]. Reads of low mapping quality, multiple mappings, and singletons were excluded. Subsequently, uniquely mapped reads were consolidated into a single file. Read pairs were filtered out if they did not align near a restriction site or did not meet the expected fragment size after shearing. Subsequent filtering steps were applied to eliminate read pairs derived from invalid ligation products, such as dangling-end and self-ligation products, as well as PCR artifacts. The remaining valid read pairs were categorized into intrachromosomal and interchromosomal pairs, and contact maps were then generated using chromosome bins of uniform sizes ranging from 3 kb to 1 Mb. The initial contact maps were normalized using a sparse-based iteration correction method within HiC-Pro and visualized utilizing HiCPlotter [34]. Finally, regions resembling topologically associated domains (TADs) and boundaries were delineated using the default algorithm within HiCPlotter at a resolution of 5 kb [34].

### Structural variation detection

In the GWAS candidate region, all copy number variation (CNV) structural variations in the selected populations were analyzed, including Pekin ducks, Liancheng ducks, WB ducks, GF ducks, and BF ducks. Ducks from these different feather groups were randomly selected, and the genotype of all individual CNVs was investigated using CNVcaller (RRID:SCR_015752) software (version 0.11) [35]. The CNV calling and genotyping procedures were consistent with those previously described [36]. Log_2_ fold change values reflected the ratio of sequencing read depths in the 1,000-bp window region to that of Pekin duck reads. Therefore, the distribution of all CNV genotypes in the aforementioned populations was examined. Copy numbers of 1, 0.5, 0, 1.5, and 2 corresponded to normal diploidy, loss of heterozygosity, homozygous loss, heterozygous duplication, and homozygous duplication, respectively. Absolute copy numbers above 2 suggested the presence of complex duplications [36].

### Luciferase reporter assay

Herein, a total of 4 haplotypes of candidate variations SNP1 and SNP2, along with their upstream and downstream regions, were cloned into the pGL3-basic and pGL3-promoter vector. The *XhoI* and *KpnI* restriction sites were utilized as insertion points in the pGL3-basic vector for promoter activity analysis, whereas the *BamHI* and *SalI* sites were chosen in the pGL3-promoter vector for enhancer activity assessment. A375 and duck embryo fibroblast (DEF) cells were plated in 48-well plates at a density of 0.5 × 10^5^ per well and cultured for 24 hours in Dulbecco’s modified Eagle’s medium (Pricella) mixed with 10% fetal bovine serum (Pricella). Subsequently, the cells were transfected using Lipofectamine 8000 (Beyotime), with each well receiving an equal amount of DNA (237.5 ng), encompassing the 4 sequences that included the SNP1 and SNP2 sites. Meanwhile, 12.5 ng pRL-TK vector was added to each well. Following the manufacturer’s protocol, cell lysates were harvested postlysis, and luciferase activity was measured using the Veritas Microplate Luminometer (Promega). Each sample was assayed in triplicate, with *Renilla* luciferase activity employed to normalize the firefly luciferase readings [12, 37].

## Analyses

### A newly assembled high-quality Liancheng duck genome

**Figure 1:**
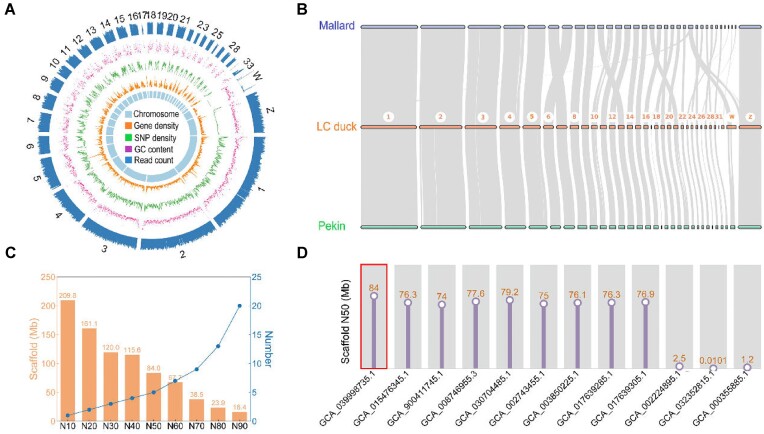
Overview of the assembly quality and characteristics of the Liancheng duck genome. (A) Circular diagram illustrating the characteristics of the genome assembly. The tracks from the inner to outer circles represent the following: chromosomes, gene density, SNP density, GC content (%), and read count. The window size of each circle was 200 kb. (B) Genome synteny analysis between the Liancheng duck and Mallard, as well as Pekin duck. Chromosomes 1–33, as well as the 2 sex chromosomes. (C) Genome statistics for the hifiasm genome assemblies of the Liancheng duck genome in this study. (D) The length of scaffold N50 (Mb) of Liancheng duck in this study (GCA_039998735.1) was compared with all previously published duck (*Anas platyrhynchos*) genomes.

To facilitate a comprehensive analysis of the white plumage Liancheng duck, a *de novo* genome was constructed utilizing HiFi long-read sequencing data, achieving an 88× genome coverage, and 584.72 Gb of Hi-C data were generated (Supplementary Table S4). These datasets were then utilized for *de novo* assembly for Liancheng duck genome with contig N50 of 12.17 Mb. The contigs were further scaffolded, corrected, and ordered based on Hi-C contact map with scaffold N50 of 83.98 Mb (IASCAAS_LianchengWhiteDuck, GCA_039998735.1) (Fig. 1A, Supplementary Table S5). Finally, our *de novo* assembled 1.29 Gb Liancheng duck genome exhibited perfect collinearity with Mallards (GCA_008746955.3), and the Pekin duck reference genome (GCA_015476345.1) demonstrated the high quality of our genome assembly (Fig. 1B). The length of scaffold N50 in our Liancheng duck genome assembly was the longest among all previously published duck (*Anas platyrhynchos*) genomes, indicating high continuity of our assembly (Fig. 1C, D, Supplementary Table S6). BUSCO analysis (RRID:SCR_015008) [8] revealed 96.7% universal single-copy orthologs, suggesting high completeness of our genome assembly (Supplementary Table S7). Based on the high-quality *de novo* assembled of our Liancheng duck genome, a total of 18,819 genes were predicted (Supplementary Table S8).

### Inheritance of F2 population traits conforming to the law of independent assortment

**Figure 2:**
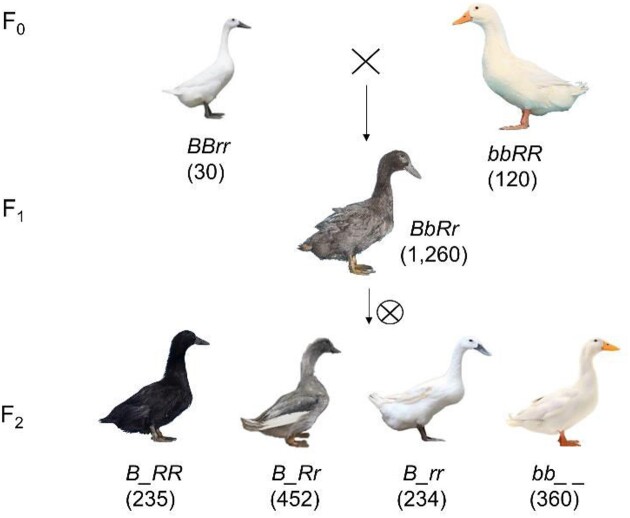
The diagram depicted the segregation of plumage colors in the F2 population. The Liancheng duck showed the white-feathered ducks with black beaks (WB), whereas the Pekin duck exhibited the white-feathered ducks with yellow beaks (WY). The F1 generation displayed the gray-feathered ducks with black beaks (GF). In the subsequent F2 generations, ducks were observed with phenotypes, including black-feathered ducks with black beaks (BF), GF, WB, and WY ducks.

A crossbreeding study involving 30 Pekin and 120 Liancheng ducks was conducted. All F1 individuals (1,260) displayed a gray plumage color and pattern. In the F2 populations, 4 phenotypes were observed: BF, GF, WB, and WY ducks (Fig. 2). The ratio of BF/GF/WB/WY ducks in the F2 population was 235:452:234:360, which closely approximated the theoretical ratio of 3:6:3:4 (Table 1 and Supplementary Fig. S1). The phenotypic ratio observed followed Mendel’s law of independent assortment for 2 genes. It was hypothesized that the genetic mechanism controlling plumage color in Liancheng ducks was governed by 2 sites (*Bb* and *Rr* sites), with the allele at the *Rr* site, in interaction with the *Bb* site, determining white plumage in Liancheng ducks (Table 1). Within the F2 population, 2 alleles (*B*, dominant, enabling melanin synthesis, and *b*, recessive, inhibiting melanin synthesis) segregated at the *Bb* locus. The other locus, denoted as *Rr*, possessed 2 alleles that regulated melanin accumulation in the feather: *R* (dominant, allowing melanin synthesis in the feather) and *r* (recessive, repressing melanin synthesis). The *B* allele at the *Bb* locus displayed an epistatic effect, while the *R* allele at the *Rr* locus demonstrated an incomplete dominance effect. The cross between the Liancheng duck (*BBrr*) and the Pekin duck (*bbRR*) resulted in the production of gray feather ducks (*BbRr*), with the genotypes of BF, GF, WB, and WY being *B_RR, B_Rr, B_rr*, and *bb_ _*, respectively (Table 1, Fig. 2). Importantly, no significant difference was observed between the actual and expected numbers within the F2 population (*n* = 1,281, *P* = 0.345), with a squared value of 3.322 (χ2_0.05(3)_ = 7.81, χ2_0.01(3)_ = 11.34). The *Bb* locus had previously been identified as the primary gene responsible for white plumage in ducks [2].

**Table 1:** The number of F2 populations in different phenotypes and the chi-squared test

Comparison	BF duck (*B_RR*)	GF duck (*B_Rr*)	WB duck (*B_rr*)	WY duck (*bb_ _*)	Ratios	χ^2^ value	*P* value
Observed number	235	452	234	360	3:5.8:3:4.6	3.322	0.345 (ns)
Expected number	240	481	240	320	3:6:3:4		

Note: BF represents black-feathered ducks with black beaks in the F2 population; GF represents gray-feathered ducks with black beaks in the F2 population; WB represents white-feathered ducks with black beaks in the F2 population; WY represents white-feathered ducks with yellow beaks in the F2 population. χ^2^_0.05(3)_ = 7.81, χ^2^_0.01(3)_ = 11.34; ns, no significant difference.

### Genome-wide association analysis for segregating population duck plumage color

**Figure 3:**
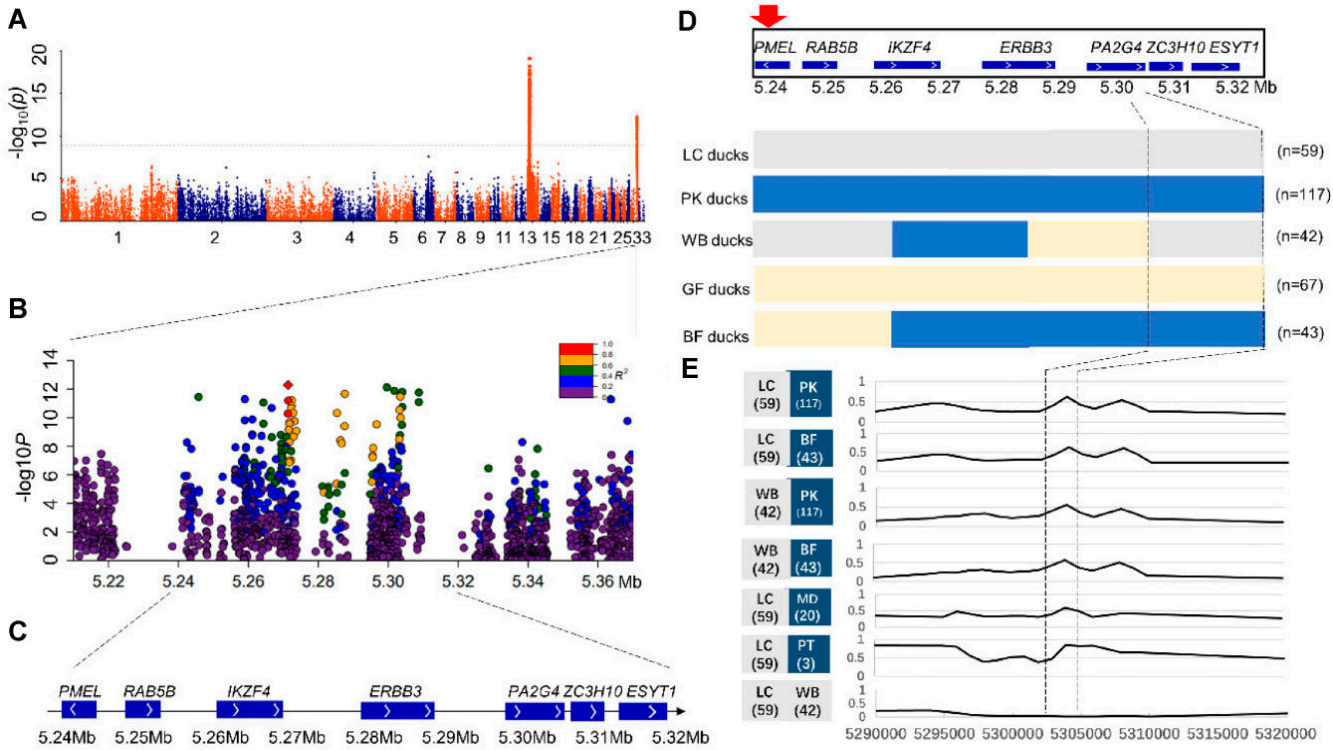
Screening for the candidate region associated with the white plumage of Liancheng ducks involved a GWAS on a cohort of 188 ducks from a cross between Pekin ducks and Liancheng ducks. (A) A Manhattan plot showed the genetic effects on plumage color. (B) Locuszoom in of the regions on chromosome 33 (5.21–5.37 Mb) linked to white plumage in Liancheng ducks. Genotypic SNPs were identified based on linkage imbalance values compared to the leading SNP in the intercross population duck (Chr33: 5,303,413). (C) Candidate genes in the region (5.24–5.32 Mb) were identified, with white and black arrows indicating gene orientation and chromosome 33 direction, respectively. (D) IBD analysis used color schemes to refine candidate regions, with blue for Pekin ducks and black-feathered ducks, gray for Liancheng ducks and white-feathered black beak ducks, and yellow for gray plumage ducks (LC vs. PK, *F*_ST_ > 0.8). (E) Genome divergence analysis among 6 duck breeds, including LC versus PK ducks, BF ducks, Mallards (MD), and Putian (PT) ducks within the candidate region (Chr33: 5.29–5.32 Mb), with averaged *F*_ST_ values in a 10-kb region in each comparison group.

The duck samples were resequenced with the average depth of 5×. A cohort of 188 ducks from a segregating population derived from Liancheng ducks and Pekin ducks was hereby selected for GWAS analysis. Initially, using the genome of the Liancheng duck as a reference, the present study identified 2 sites controlling the white feather phenotype on chromosomes 13 and 33 (Fig. 3A). This research highlighted a specific gene on chromosome 13 that regulated melanin synthesis in Liancheng ducks (Supplementary Figs. S2, S3). Given that the white plumage phenotype in Liancheng ducks did not show sex-related patterns, cytoplasmic inheritance considerations for white plumage were deemed unnecessary. Subsequently, the *Rr* gene was pinpointed to the 5.24–5.32Mb region of chromosome 33 in Liancheng ducks (Fig. 3B), which contained potential candidate genes such as *PMEL, RAB5B, IKZF4, ERBB3, PA2G4, ZC3H10*, and *ESYT1* (Fig. 3C). Within the candidate region (Chr33: 5.24–5.32 Mb), 4 minimal recombination haplotypes were identified based on the parents and segregating populations from 117 SNPs with an *F*_ST_ > 0.8 (PK vs. LC ducks). Only the haplotypes in block 4 (Chr33: 5,303,111–5,304,416,101,305 bp) located upstream of the *PMEL* gene corresponded to the observed phenotypes (Fig. 3D). Additionally, a significantly high peak in the *F*_ST_ value was observed in the *RR* versus *rr* duck populations within the selected candidate region, while no peak was spotted in *rr* versus *rr* duck populations (Fig. 3E).

### *PMEL* causes melanin deposition in duck plumages

**Figure 4:**
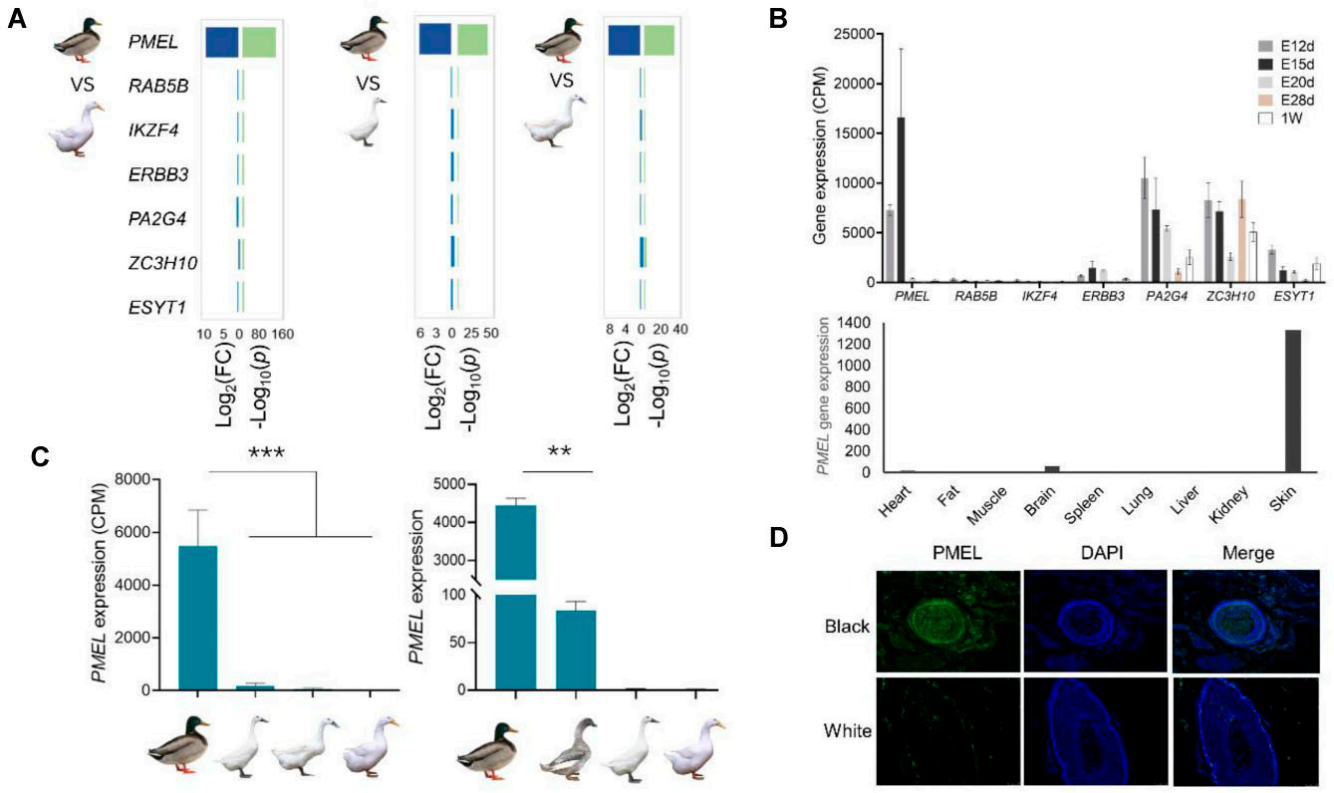
Identification of the candidate gene for white plumage in Liancheng ducks (A) Gene expression of 7 GWAS candidate region genes (*PMEL, RAB5B, IKZF4, ERBB3, PA2G4, ZC3H10, ESYT1*) in 1-week-old feather follicle samples of white- and black-feathered ducks, with 3 replicates per sample. Log_2_(FC) values were used to analyze gene expression differences in blue, and values where −Log_10_(*p*) values were shown in green. (B) Analysis of the expression levels of the 7 candidate genes in skin tissues of Liancheng ducks at different developmental stages. E12d, E15d, E20d, and E28d (also the first day of birth) represent 12, 15, 20, and 28 days of the embryonic period, respectively. Data are shown as mean ± standard error (*n* = 3). (C) CPM and qPCR results of *PMEL* in 1-week-old feather bulb samples of ducks. Data are presented as mean ± standard error (*n* = 3). **P < 0.01, ***P < 0.001. (D) Immunofluorescence results showing PMEL distribution in feather bulb specimens of black- and white-feathered ducks. Black (Mallards) and white (Liancheng ducks).

The region on chromosome 13 was found to encompass the *MITF* gene in the GWAS analysis (Supplementary Fig. S2). Comparison with the Pekin duck genome assembly (GCA_003850225.1) revealed a 6.6-kb insertion in the *MITF* gene, strongly associated with melanin synthesis in ducks (Supplementary Table S9). Following the exclusion of the *MITF* signal, subsequent GWAS analysis identified a single signal on chromosome 33 (Supplementary Fig. S3). Candidate region included *PMEL, RAB5B, IKZF4, ERBB3, PA2G4, ZC3H10*, and *ESYT1*. Results indicated that only the *PMEL* gene showed significant differential expression (Fig. 4A, B), with higher expression levels in black-feathered ducks compared to gray-feathered ducks (−Log10(*p*) > 30). RNA-seq results showed no expression of the *PMEL* gene in feather bulb specimens of white-feathered ducks (Fig. 4C, D). Other genes within the GWAS candidate range (Chr33: 5.24–5.32 Mb) were excluded due to similar gene expression levels in different plumage populations or inconsistent gene expression patterns related to melanin regulation. Furthermore, qPCR results confirmed *PMEL* as the *Rr* gene (Fig. 4C). Notably, the *PMEL* gene exhibited an elevated average GC content of 72.4% (Supplementary Fig. S4). Additionally, the *PMEL* gene expression correlated with the plumage color phenotype of Liancheng ducks at various developmental stages, with high expression levels observed in the skin tissue (Fig. 4B), further underscoring the importance of establishing a high-quality genome for Liancheng ducks.

### *Rr* variation being fine mapped to *PMEL* upstream regulatory region

**Figure 5:**
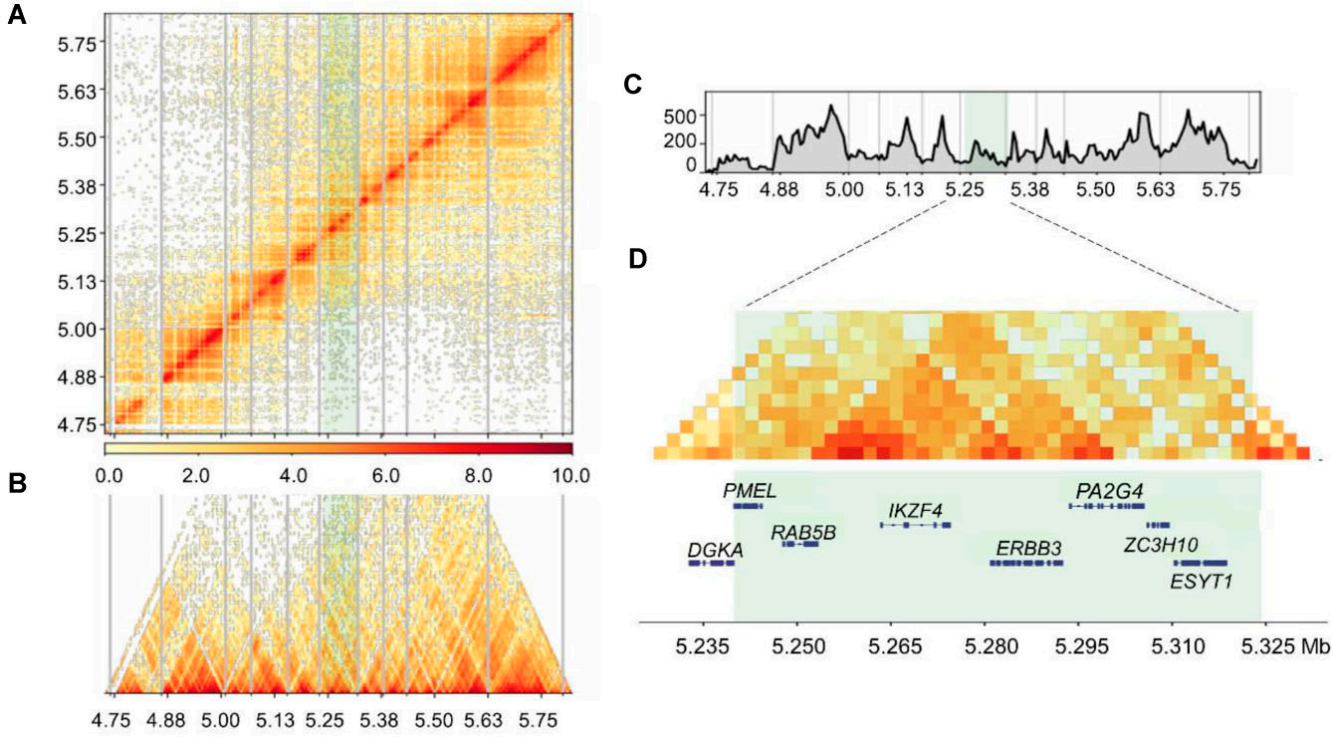
High-through chromosome conformation capture (Hi-C) result of the end of chromosome 33 (4.75–5.8 Mb) in skin fat of Liancheng ducks. (A) Log_2_(interaction matrix) analysis of Chr33 (resolution: 5 kb). Strong contacts are shown in red, and weak contacts are shown in white. The heatmap shows a normalized contact matrix in 5-kb bins. Light green indicates the *Rr* site candidate region by GWAS analysis. (B) Triangular result of Log_2_ (interaction matrix) of Chr33 (4.75–5.75 Mb) and the (C) TAD-like and boundary-like regions were identified with the default algorithm built in HiCPlotter at a resolution of 5 kb. (D) Log_2_ (interaction matrix) of Chr33 (5.235–5.325 Mb). Candidate regions identified from Chr33: 5.24–5.32 Mb by GWAS result and genes within it (light green regions).

The results of the IBD analysis indicated that only the haplotypes in block 4 (Chr33: 5,303,111–5,304,416,101,305 bp), positioned upstream of the *PMEL* gene, were associated with the observed phenotypes (Fig. 3D). Moreover, a pronounced peak was observed in the *RR* versus *rr* duck populations within the candidate region (Fig. 3E), further substantiating this segment (Chr33: 5,303,111–5,304,416,101,305 bp) as the *Rr* locus. Among the identified candidate variations in this region, 1 CNV (Supplementary Figs. S5 and S6) was initially excluded. Only 12 SNP variants and 2 indels were retained after applying a threshold of *F*_ST_ > 0.8 (PK vs. LC ducks) (Supplementary Table S10). It should be noted that all 12 SNP were found in the upstream regulatory region of the *PMEL* gene (Chr33: 5,239,969–5,244,318). Finally, 2 SNPs (Chr33: 5,303,994A>G; 5,303,997A>G) were hereby identified as the potential causal variants across all duck breeds (Supplementary Table S10). Intriguingly, these 2 SNPs were observed to be in complete linkage disequilibrium. Additionally, Hi-C data revealed that the *PMEL* gene, along with its upstream region harboring the 2 candidate SNPs, resided within a TAD (Fig. 5).

### Functional analysis of 2 candidate SNPs

**Figure 6:**
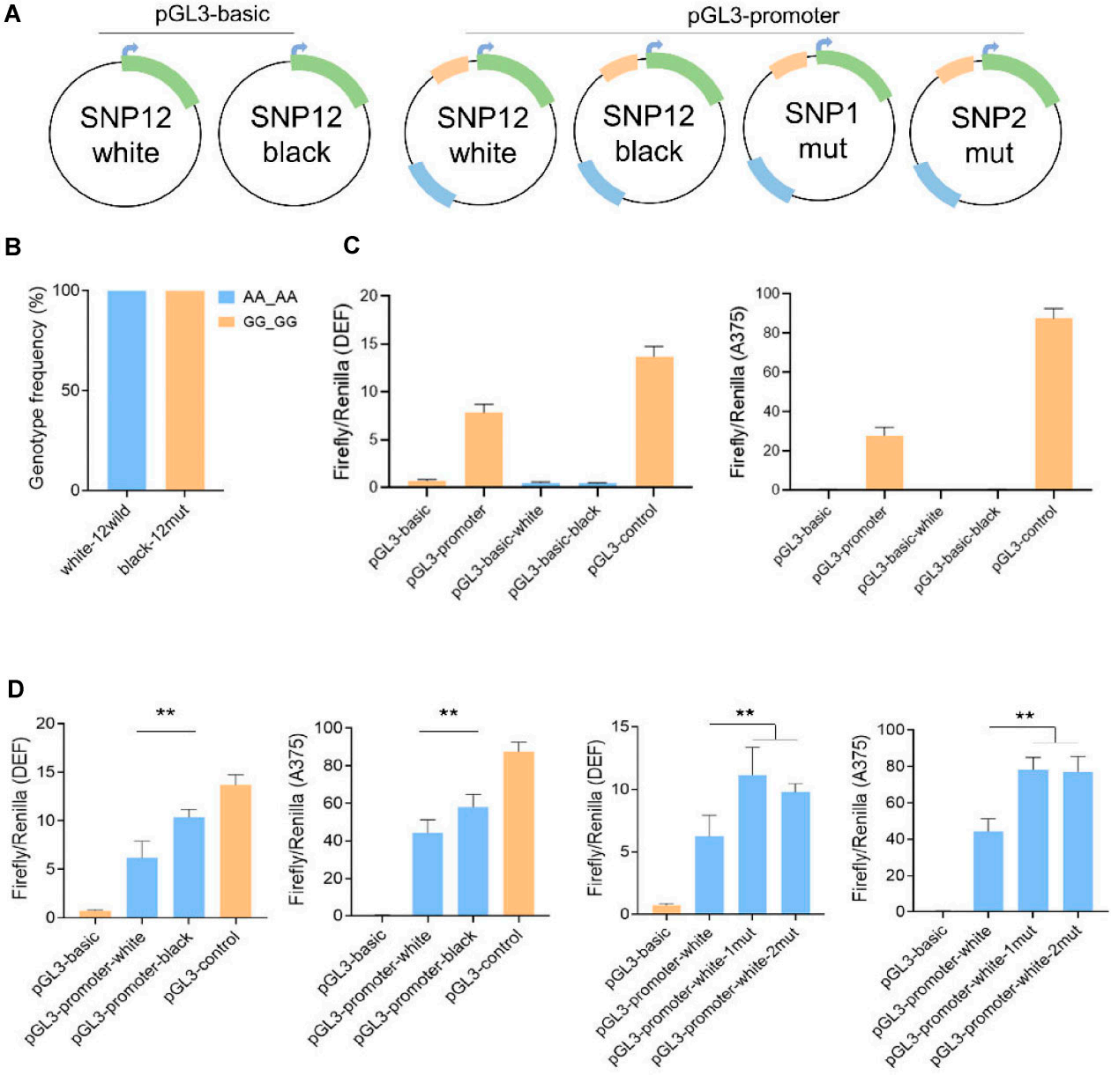
Functional analysis of the candidate variation controlling the white plumage phenotype of Liancheng duck. (A) Diagram of 6 pGL3 vectors for the luciferase reporter gene experiment. Candidate SNP1 and SNP2 (Chr33: 5,303,994A>G and 5,303,997A>G) of Liancheng duck and Pekin duck were inserted into empty pGL3-basic and pGL3-promoter vectors. pGL3-basic, pGL3-promoter, and pGL3-control were used as negative and positive control. Insertion fragments are marked in yellow and blue in pGL3-basic to verify the promoter activity. The yellow and blue boxes represents insertion fragment of the pGL3-promoter to verify enhancer activity. Additionally, vectors also contained a green box (luciferase reporter gene), grey box (promoter region) and a blue arrow (transcription site). (B) Genotype distribution of SNP1 and SNP2 in Liancheng duck (AA_AA, *n* = 59) and Pekin duck (GG_GG, *n* = 117). (C) Validation of promoter activity of homozygous wild-type (AA_AA) and homozygous mutation (GG_GG) in DEF cells and A375 cells. Data are presented as mean ± standard error (*n* = 8) (ANOVA, *P* > 0.05). (D) Enhancer activity of AA_AA, GG_GG, GG_AA, and AA_GG vectors in DEF cells and A375 cells. Data are presented as mean ± standard error (*n* = 8) (ANOVA, **P < 0.01).

Considering the location of the 2 candidate SNPs (SNP1 and SNP2) (Chr33: 5,303,994A>G; 5,303,997A>G) in the noncoding upstream region of the *PMEL* gene, their promoter and enhancer effects were assessed using pGL3 luciferase vectors (Fig. 6A, B). Both pGL3 vectors (pGL3-basic-white and pGL3-basic-black) with inserts showed minimal luciferase activity in DEF cells and human melanoma cells (A375) (Fig. 6C), indicating no promoter activity at these SNP sites. However, for enhancer activity, those vectors with inserts (pGL3-promoter-white and pGL3-promoter-black) displayed significantly different luciferase activity in DEF cells and A375 cells (analysis of variance [ANOVA], *P* < 0.01). Notably,results revealed the higher luciferase activity of both pGL3-promoter-white-1mut and pGL3-promoter-white-2mut compared with pGL3-promoter-white (Fig. 6D). This indicated a synergistic enhancement activity by the black alleles of variations SNP1 and SNP2.

Analysis on the JASPAR transcription prediction website revealed that multiple transcription factors might bind differently to sequences surrounding candidate SNPs located at Chr33: 5,303,944–5,304,098. The results suggested that variations in SNP1 and SNP2 could impact the binding of various transcription factors, as illustrated in Supplementary Fig. S7. Furthermore, differential expression of the SOX5 transcription factor was observed in the feather follicles of white- and black-feathered ducks. It was thereby hypothesized through these findings that variations in SNP1 and SNP2 could potentially be key mutations responsible for the white feather phenotype.

## Discussion

The white plumage phenotype is considered a common trait in various avian species, such as chickens [38, 39], peafowl [40], geese [41], and ducks [2, 42]. The unique white plumage phenotype of Liancheng ducks has been extensively delved into [42–44], but the genetic mechanisms behind it remain unclear. The present findings suggested that the inheritance of the plumage color phenotype in Liancheng ducks was likely governed by 2 autosomal genes, independent of the sex chromosomes (Table 1). Despite using the previous Pekin duck reference genome (IASCAAS_PekingDuck_PBH1.5, GCA_003850225.1), 3 signals were identified through the current GWAS analysis (Supplementary Fig. S8), which was considered to be attributed to an incomplete genome assembly. To further investigate the white feather phenotype of Liancheng ducks, a high-quality genome was therefore first established for Liancheng ducks (Fig. 1). This new reference genome featured a size of 1.29 Gb, with contig and scaffold N50 values of 12.17 and 83.98 Mb, respectively (Supplementary Table S5). The scaffold N50 length of the Liancheng duck genome was higher than that of other duck genomes [45–48], representing a more complete and better continuity of the duck genome assembly. However, the chromosomes number identified from this newly genome should still be further improved compared to those of the Muscovy duck and crested duck [49, 50]. Further pan-genome and functional gene‐mining analysis could be conducted in the future [29, 47, 51]. Taken together, these findings represent the first construction of the Liancheng duck genome, resulting in enhanced genome contiguity compared to previous duck genomes.

*MITF* is a key regulator of melanin synthesis, controlling the expression of enzymes involved in this process as well as receptors critical to melanocyte function [52–54]. This gene produces multiple isoforms through the use of alternative promoters, which share coding exons but feature different amino termini [55]. While *MITF* variants are known to influence melanin regulation, the regulation of these isoforms remains to be explored. Herein, the expression of 2 *MITF* isoforms, *MITF-B* and *MITF-M*, were discovered in ducks, with only the latter being crucial for melanin synthesis in duck plumage [2, 9, 56]. *MITF-M* isoforms have also been shown to regulate white coloration in the fur of dogs [57], llamas [58], and mice [59]. SNPs, indels, and structural variants were previously found in *MITF* as possible causes of white plumage in ducks [60]. Two synonymous SNPs (c.114T>G and c.147T>C) and a 14-bp indel (GCTGCAAACAGATG) in intron 7 of duck *MITF* were significantly associated with the black- and white-colored breeds (*P* < 0.001) [61]. One variant in the 5′UTR of *MITF* was significantly associated with feather color phenotypes in geese [62]. A 6.6-kb insertion within the *MITF* gene demonstrated a strong correlation with melanin production in ducks [2] and indicated the on/off role of *MITF* in the melanin generation pathway of Pekin ducks. *MITF* could promote differentiation-related functions, including regulation of genes involved in pigmentation, such as *PMEL, TYR, TYRP1, DCT, MLANA, SILV*, and *SLC24A5* [60]. In the years following the separation of the *MITF* gene, the number of potential target genes increased sharply. Based on GWAS analysis, the role of the *MITF* gene as an epistatic gene controlling melanin synthesis in Liancheng ducks was hereby confirmed, aligning with previous research findings [44]. Overall, this highlights the significant regulatory role of *MITF* in melanin synthesis in Liancheng ducks and underscores its importance as a key genetic factor in pigmentation.

PMEL, a type I transmembrane transport glycoprotein, is synthesized in the endoplasmic reticulum and plays a crucial role in amyloid fiber formation during stages I and II of melanosome formation in the L-dopa pathway [63, 64]. Upon synthesis, *PMEL* is transported to the melanosomes, where it undergoes proteolytic processing to form fibrils [65]. These fibrils act as a scaffold for the deposition of melanin pigments, catalyzed by enzymes like tyrosinase [66, 67]. Mutations in the *PMEL* gene can lead to abnormalities in melanosome formation and melanin deposition, impacting plumage coloration in various bird species, including chickens [38, 68], Junco hyemalis [69], Japanese quail [70], and Indian peafowl [40]. To date, only 21 bird species have annotated the *PMEL* gene among 120 bird genomes (Supplementary Table S11). However, the association between the *PMEL* gene and duck plumage color phenotype has not been previously reported.

In this study, the *PMEL* gene was found to be significantly differentially expressed between the feather bulb specimens of white- and black-feathered ducks (ANOVA, *P* < 0.001). The immunofluorescence results indicated high expression of the *PMEL* protein in feather follicle specimens of black and gray plumage ducks, contrasting with low expression in white plumage ducks. Many other studies suggested the possible involvement of the *PMEL* gene in the deposition of feather melanin [69, 71–73]. Meanwhile, the *PMEL* gene is also implicated in the formation of white feathers in quail [74] and in the white feathers of chickens at the hatch stage [75]. Endogenous *PMEL* expression is regulated by *MITF*, with alterations observed in melanoma cells [76]. However, the specific interplay between these 2 genes in determining the plumage color of Liancheng ducks requires further investigation. Overall, this research has annotated the *PMEL* gene in the domestic duck genome (Supplementary Fig. S4, Supplementary Table S12), a gene previously thought to be absent in Pekin ducks. *MITF*, a key regulator of melanin production in ducks, was also identified. The inactivation of *PMEL* in feather bulb specimens led to the distinct white and black feathering characteristic of Liancheng ducks.

In the candidate region identified through GWAS analysis, a total of 12 SNPs, 2 indel variations, and 1 CNV variation were investigated (Supplementary Table S10, Supplementary Figs. S7 and S8). Among these variants, only 2 SNPs (Chr33: 5,303,994A>G and 5,303,997A>G) were found to be consistently associated with the observed plumage color phenotypes across multiple breeds. Data from promoter activity assays indicated that the SNPs in question were unlikely to serve as direct *PMEL* promoters for gene expression (Fig. 6C). Instead, the present genetic findings indicated that these 2 linked SNP variations, located in the upstream region of the *PMEL* gene, exhibited functional enhancer activity that might remotely regulate *PMEL* gene expression (Supplementary Figs. S5 and S6). The long-range regulation probably modulates *PMEL* gene expression, affecting the black feather pigmentation in ducks, aligning with the melanin phenotype of duck feathers.

Remote regulation elements are thought to engage with target promoters through physical proximity [77], yet the precise functional implications of this proximity are not well defined. In the current research, potential regions encompassing *PMEL* and 2 candidate SNPs were identified within a single topologically associated domain region (Fig. 5), demonstrating this area as a possible part of a genomic region with frequent interactions. The process of loop extrusion not only promoted interactions within the TAD but also shielded the TAD from external influences [78]. Furthermore, enhancer–promoter interactions might intensify during mammalian development [79], potentially accounting for the variation in plumage colors of Liancheng ducks from embryonic to postnatal stages. It was also noted that only SOX5, the predicted transcription factor, displayed varying expression levels in feather bulbs of different plumage colors (Supplementary Fig. S7). Notably, other transcription factors might also contribute to the regulatory mechanism. Moreover, the gray plumage, an intermediate phenotype observed in this study, could be related to a haploinsufficiency effect [80].

Feather phenotype is a complex trait that encompasses a series of interlinked modules [81]. Birds exhibit a wide array of elaborate pigmentation patterns, which serve various functions such as attracting mates or providing camouflage or intimidation against predators. Melanin is a crucial pigment in feather coloration, and its role is mediated through the regulated presence, distribution, and differentiation of these melanocytes. Recent studies have reported that the variation of *MITF, PMEL, TYR, EDNRB2, SLC45A2*, and *MC1R* genes and Agouti signaling protein can regulate the production of feather melanin in ducks [2, 82]. However, the role of the *PMEL* gene in feather melanin formation in ducks has not been confirmed. The present study makes the meaningful attempt to elucidate the role of the *PMEL* gene in pigmentation within duck feathers, offering valuable insights into the genetic mechanisms behind plumage coloration. It also holds practical implications for selective breeding and conservation initiatives.

## Supplementary Material

giae114_Supplemental_Files

giae114_GIGA-D-24-00213_Original_Submission

giae114_GIGA-D-24-00213_Revision_1

giae114_GIGA-D-24-00213_Revision_2

giae114_Response_to_Reviewer_Comments_Original_Submission

giae114_Response_to_Reviewer_Comments_Revision_1

giae114_Reviewer_1_Report_Original_Submission Gene Ng -- 7/25/2024

giae114_Reviewer_1_Report_Revision_1 Gene Ng -- 10/21/2024

giae114_Reviewer_2_Report_Original_Submission Filippo Biscarini -- 9/15/2024

giae114_Reviewer_2_Report_Revision_1 Filippo Biscarini -- 10/8/2024

## Data Availability

The whole-genome sequence data reported in this article have been deposited in the NCBI under accession number No. PRJNA1107839. The resequencing raw data have been deposited in the NCBI SRA under accession No. PRJNA844232. The transcriptomic raw data have been deposited in the NCBI under accession No. PRJNA1109286. All additional supporting data are available in the *GigaScience* repository, GigaDB [83].

## Additional Files

**Supplementary Fig. S1**. Phenotypic characteristics of 1,281 F2 ducks from Liancheng and Pekin ducks.

**Supplementary Fig. S2**. Screening for the candidate region associated with the white plumage of Liancheng ducks by GWAS in 188 ducks from a cross between Liancheng and Pekin ducks.

**Supplementary Fig. S3**. Manhattan plot showing the genetic effects on the plumage color according to a GWAS from a cross of Liancheng and Pekin ducks except WY ducks based on the Liancheng duck genome (GCA_039998735.1).

**Supplementary Fig. S4**. The collinearity analysis of comparison between the *PMEL* gene and its upstream and downstream 100-kb region in Liancheng duck and other birds.

**Supplementary Fig. S5**. Illustration of the read depth analysis that confirmed the copy number variations on the GWAS candidate region (Chr33: 5.24–5.32Mb).

**Supplementary Fig. S6**. Genotypes of candidate CNV variation (Chr33:5,282,001–5,284,500) in different plumage color populations.

**Supplementary Fig. S7**. Heatmap cluster analysis of transcription factors.

**Supplementary Fig. S8**. Manhattan plot showing the genetic effects on the plumage color according to a GWAS in ducks from a cross of Liancheng and Pekin ducks based on previous reference genome of the Pekin duck (GCA_015476345.1).

**Supplementary Fig. S9**. Phenotypic characteristics of Liancheng ducks, Pekin ducks, and Mallards during embryonic and postnatal periods.

**Supplementary Table S1**. List of 366 duck genome resequencing used in the study.

**Supplementary Table S2**. List of 37 ducks for RNA-seq used in the study.

**Supplementary Table S3**. The information on the primers used for qPCR.

**Supplementary Table S4**. Table summary of Hi-C reads mapping results.

**Supplementary Table S5**. The genome scaffolds and contigs information of the Liancheng duck genome (GCA_039,998,735.1).

**Supplementary Table S6**. Assembly statistics of the Liancheng duck genome and previous duck genomes.

**Supplementary Table S7**. Complete evaluation table of Liancheng duck genome BUSCOs.

**Supplementary Table S8**. Summary of chromosome regions on the genome.

**Supplementary Table S9**. Frequency distribution of a 6.6-kb insertion within the*MITF* gene in the duck population.

**Supplementary Table S10**. Genotypic distribution of SNP candidate variations in different duck breeds based on reference genome Liancheng duck.

**Supplementary Table S11**. All birds with *PMEL* genes in their avian genomes.

**Supplementary Table S12**. The blast results of sequence identity between the newly annotated mRNA sequence of *PMEL* gene in Liancheng duck and other birds.

## Abbreviations

A375: human melanoma cells; BF: black-feathered ducks with black beaks; BLAST: Basic Local Alignment Search Tool; bp: base pairs; BUSCO: Benchmarking Universal Single Copy Orthologs; BWA: Burrows–Wheeler aligner; CNV: copy number variation; CPM: counts per million; DEF: duck embryo fibroblast; eggNOG: Evolutionary genealogy of genes: Non-supervised Orthologous; FC: fold change; FST: fixation index; Gb: gigabase pairs; GC: guanine–cytosine; GF: gray-feathered ducks with black beaks; GO: Gene Ontology; GWAS: genome-wide association study; Hi-C: high-throughput/resolution chromosome conformation capture; HiFi: high-fidelity reads; IBD: identity by descent; Indel: insertion and deletion; kb: kilobase pairs; KEGG: Kyoto Encyclopedia of Genes and Genomes; LC: Liancheng duck; MAF: minor allele frequency; Mb: megabase pairs; MD: Mallard; NCBI: National Center for Biotechnology Information; NR: Non-Redundant Protein Database; PacBio: Pacific Biosciences; PASA: Program to Assemble Spliced Alignments; PCA: principal component analysis; PK: Pekin duck; PT: Putian ducks; RNA-seq: RNA sequencing; SNP: single-nucleotide polymorphism; SRA: Sequence Read Archive; SWISS-PROT: Swiss-Prot Protein Sequence Database; TAD: topologically associated domain; WB: white-feathered ducks with black beaks; WY: white-feathered ducks with yellow beaks; ΔAF: allele frequency difference.
