# Supplementary material for: A high-quality assembly revealing the PMEL gene for the unique plumage phenotype in Liancheng ducks
Source: Gigascience. 2025 Jan 13;14:giae114. doi: 10.1093/gigascience/giae114 (PMC11727711; doi:10.1093/gigascience/giae114)
Supplement: giae114_Supplemental_Files [file giae114_supplemental_files.zip › Supplementary Figures.docx]

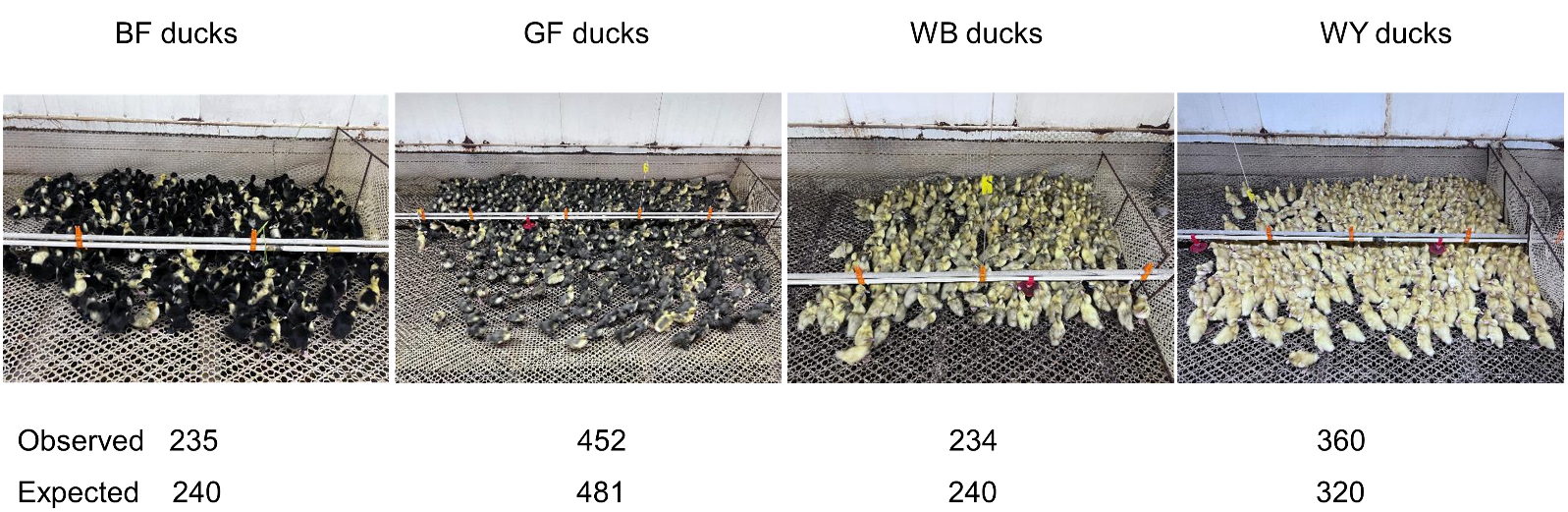


**Figure S1**. Phenotypic characteristics of 1,281 F2 ducks from Liancheng (LC) and Pekin (PK) ducks. BF: Black-feathered ducks with black beaks in F2 population; GF, Grey feathered ducks with black beaks in F1 and F2 population; WB, White feathered ducks with black beaks in F2 populations; WY, White feathered ducks with yellow beaks in F2 population. According to Mendel's law of free combination of two genes (3:6:3:4), the values below the figures represent the number of observed and expected values.


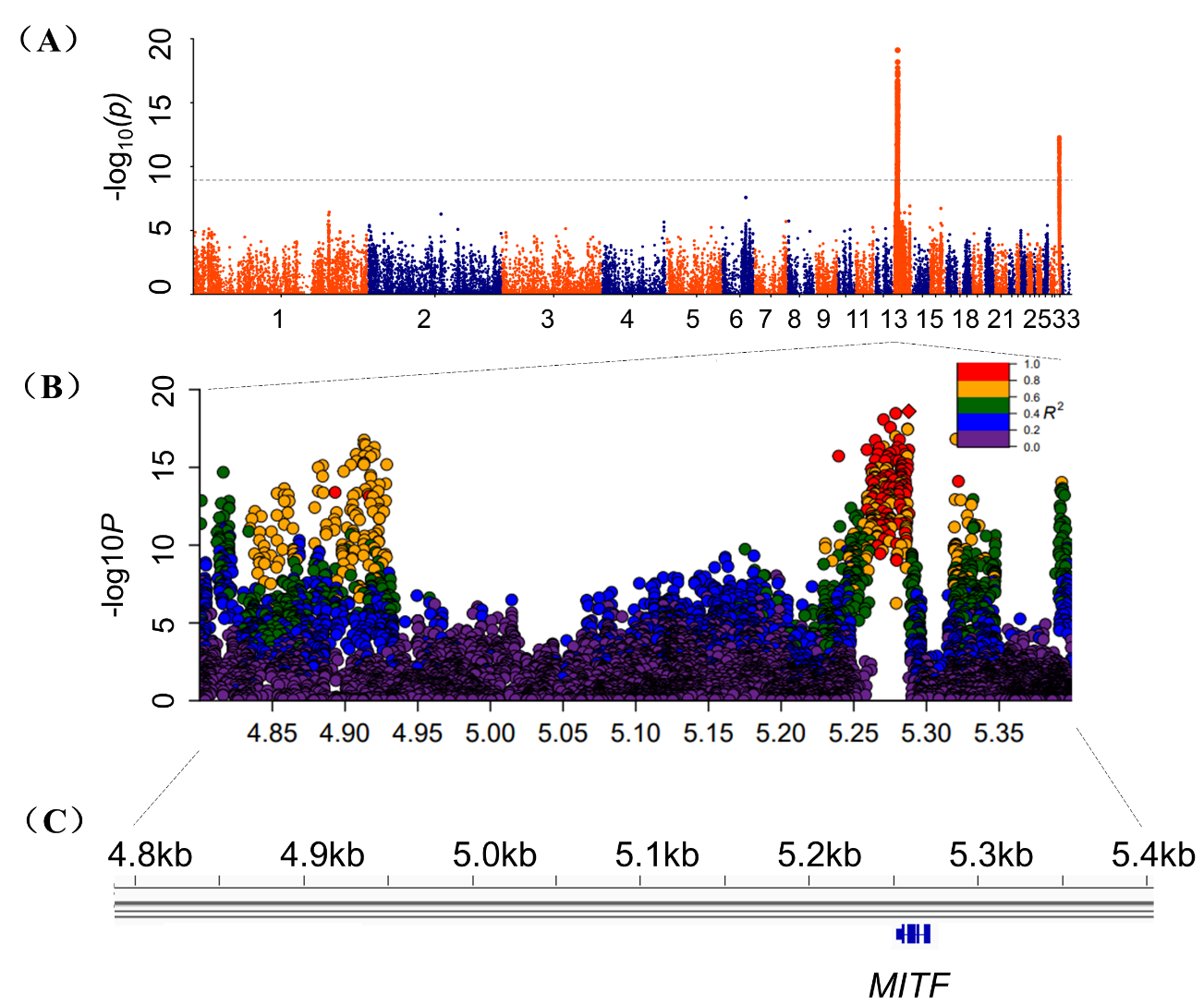


**Figure S2**. Screening for the candidate region associated with the white plumage of Liancheng ducks by GWAS in 188 ducks from a cross between Liancheng and Pekin ducks. (A) Manhattan plot showing the genetic effects on the plumage color according to a GWAS in ducks from a cross of Liancheng and Pekin ducks based on newly reference genome of Liancheng ducks (GCA_039998735.1). (B) Locuszoom results for the loci ranging from 4.8 Mb to 5.4 Mb along chromosome 13. All genotypic SNPs were derived from their linkage imbalance values against the leading SNP calculated in the intercross population duck (Chr13: 5,287,850). (C) *MITF* genes was captured in the candidate region, and *MITF* gene is a known switch gene for duck melanin synthesis in previous report (Zhou et al., 2018).


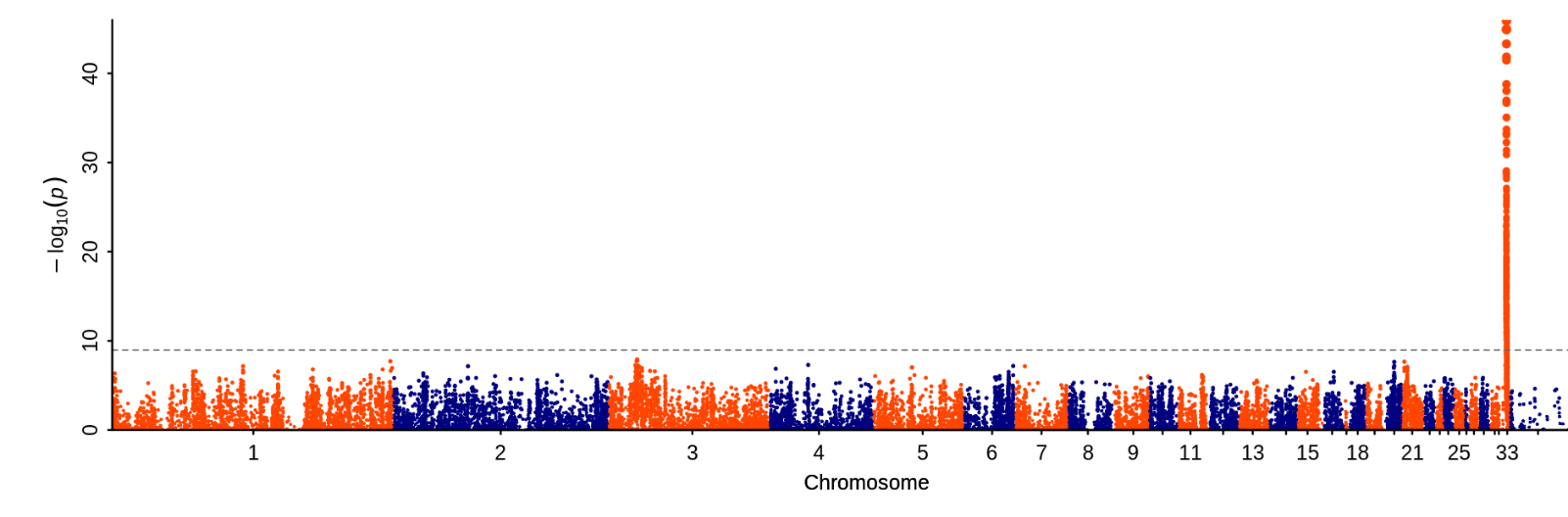


**Figure S3**. Manhattan plot showing the genetic effects on the plumage color according to a GWAS from a cross populations of Liancheng and Pekin ducks except WY ducks based on Liancheng duck genome (GCA_039998735.1). The results showed the lead SNP (Chr33:5,308,761) was associating with the white plumage phenotype of Liancheng duck and -log10(*p*)=45.88.


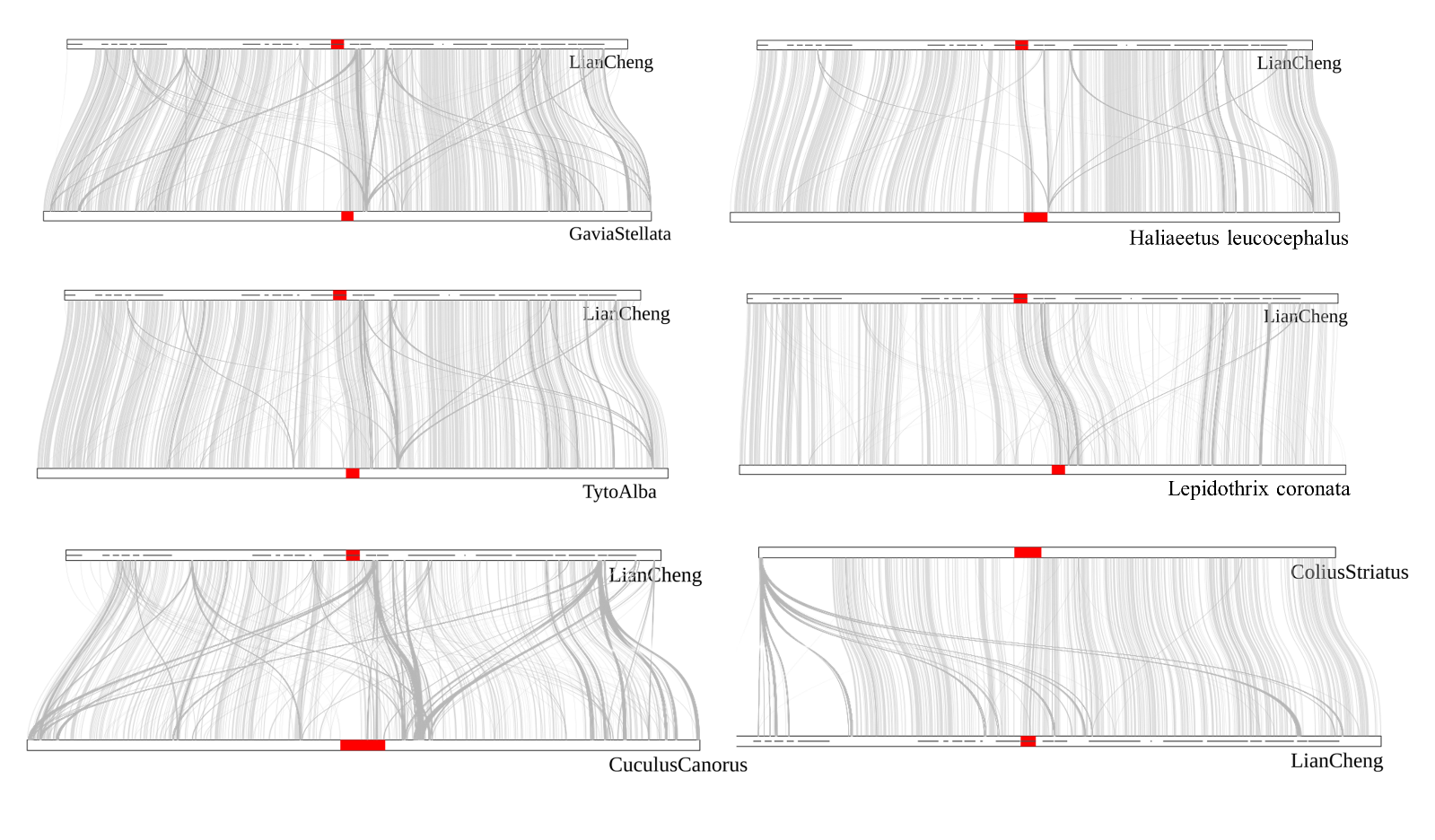


**Figure S4**. The collinearity analysis of comparison between the *PMEL* gene and its upstream and downstream 100kb region in Liancheng duck and other birds. The red areas represent newly annotated *PMEL* genes in Liancheng ducks and other birds.


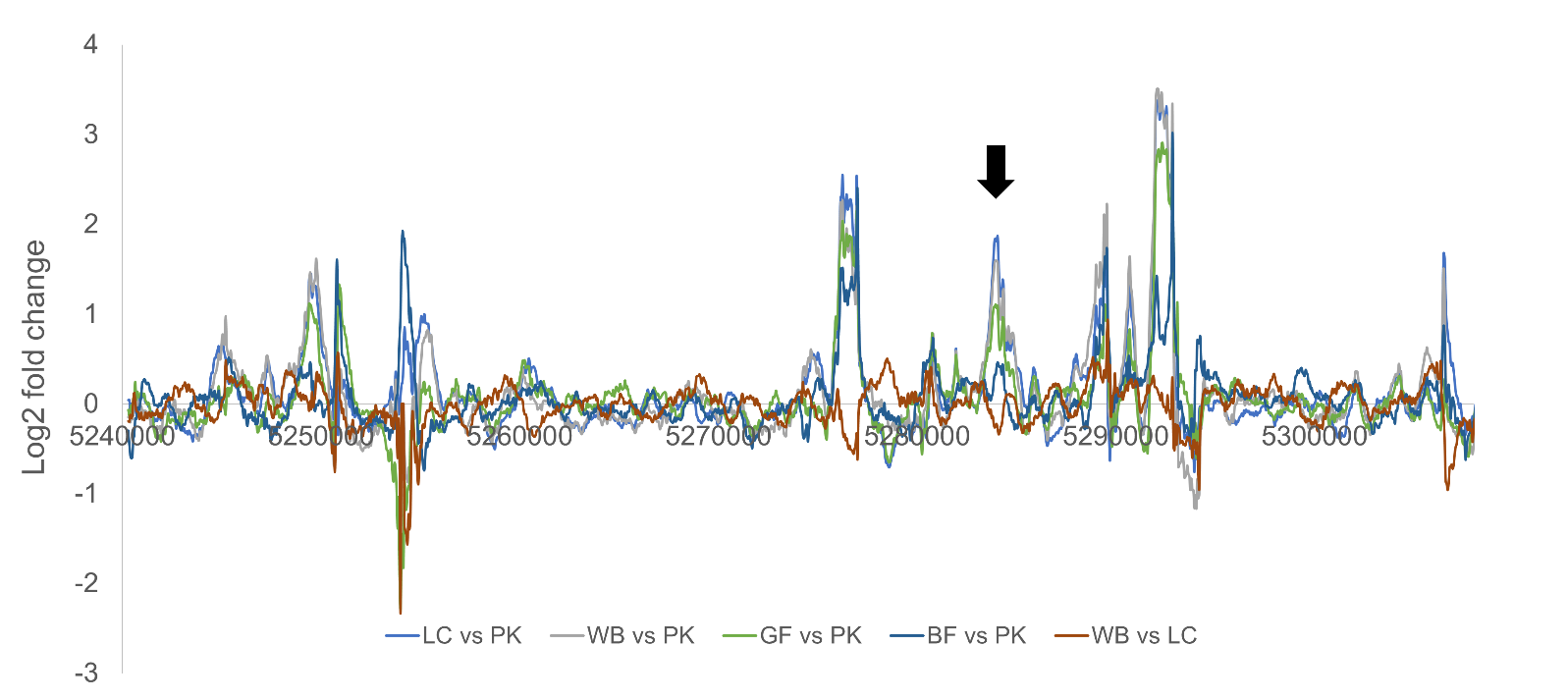


**Figure S5**. Illustration of the read depth analysis that confirmed the copy number variations on GWAS candidate region (Chr33: 5.24-5.32Mb). The log_2_ fold-change values from whole genome re-sequencing data illustrating the read depth differences between LC, WB, GF, BF and PK ducks, as well as WB vs LC ducks. The region represented by the black arrow indicates that this CNV variant (Chr33:5,282,001- 5,284,500) is a candidate CNV variant consistent with the duck plumage color phenotype.


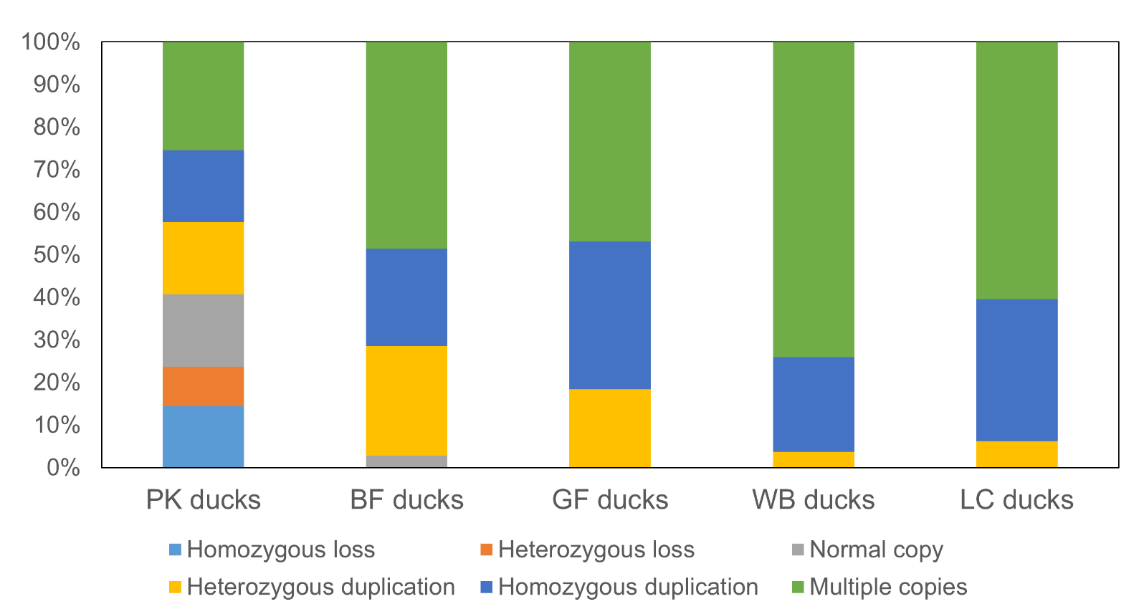


**Figure S6**. Genotypes of candidate CNV variation (Chr33:5,282,001- 5,284,500) in different plumage color populations, including PK ducks (n=31), BF ducks (n=18), GF ducks (n=21), WB ducks (n=12), LC ducks (n=24). When the absolute copy number is 1, it is normal copy number, that is, normal diploid. 0.5 indicates loss of heterozygosity; 0 means homozygous loss; 1.5 indicates heterozygous duplication; 2 represents homozygous duplication; An absolute copy number greater than 2 indicates complex copies.


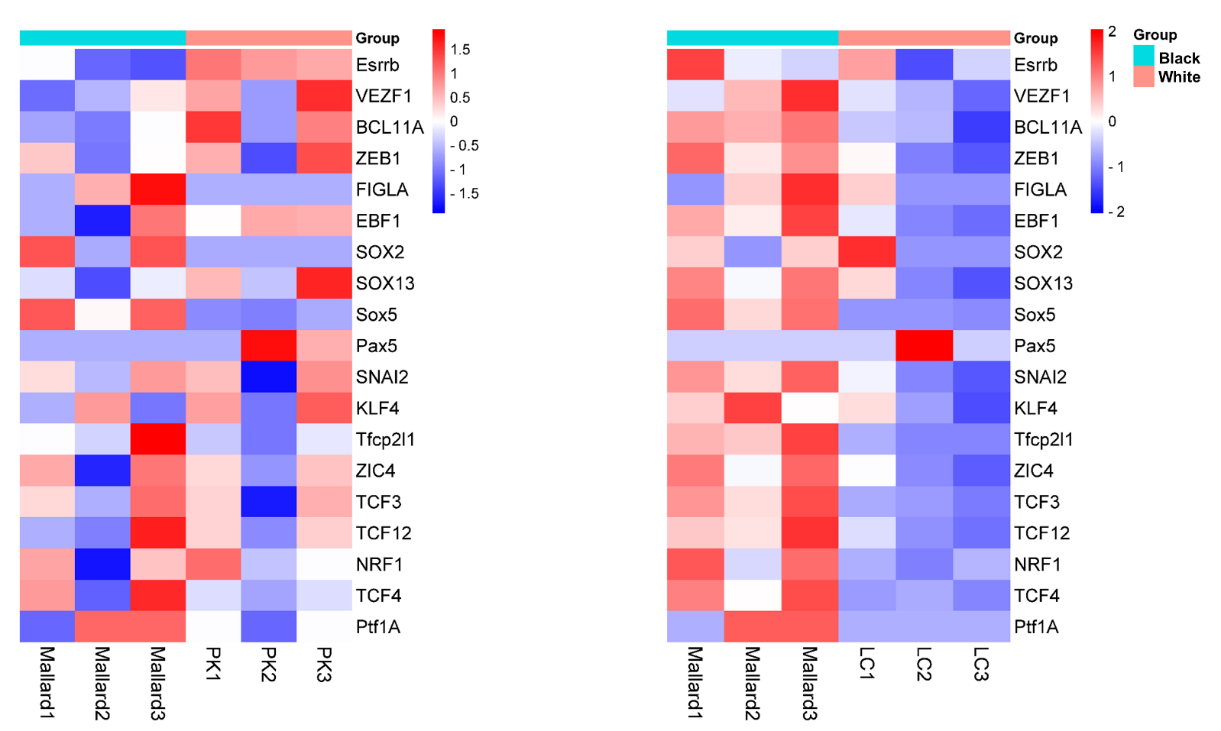


Figure S7*.* Heatmap cluster analysis of transcription factors. Three web tools were used to predict the transcription factors of SNP1 and SNP2 sites by inputting the Homozygous wildtype and homozygous mutant sequence. All expression data of transcription factors were obtained from transcriptome data of feather bulb specimens of 3 Mallards, 3 LC ducks, and 3 PK ducks.


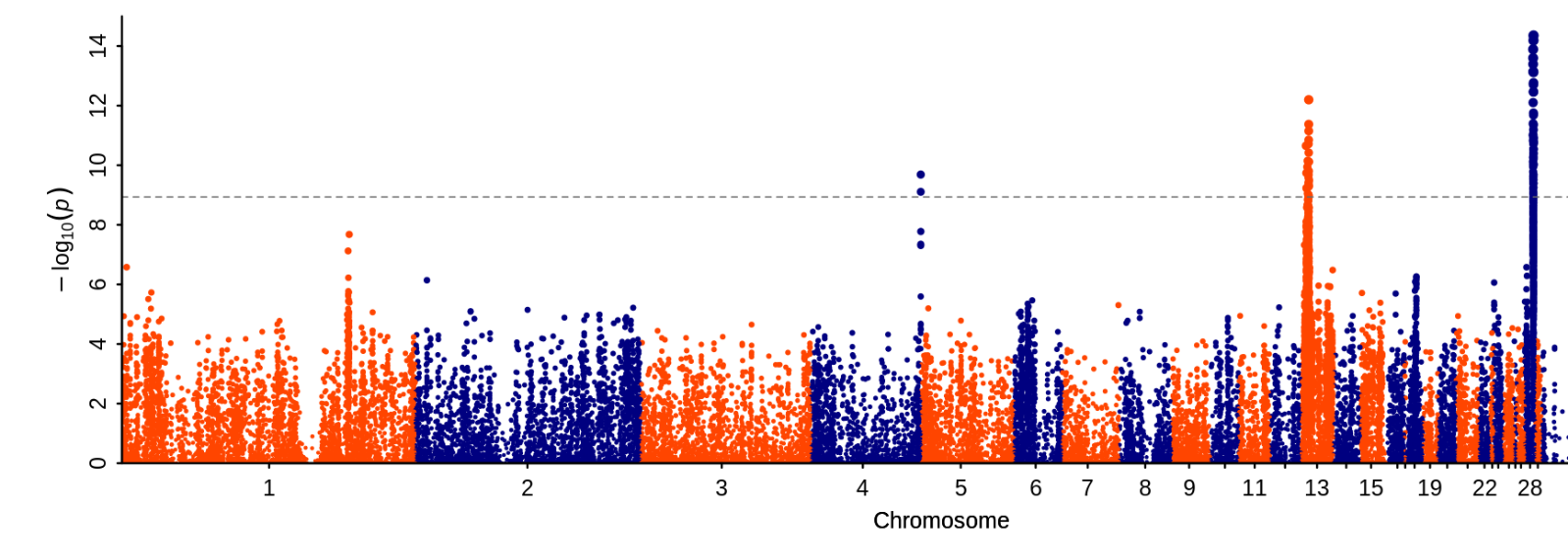


**Figure S8**. Manhattan plot showing the genetic effects on the plumage color according to a GWAS in ducks from a cross of Liancheng and Pekin ducks based on previous reference genome of Pekin duck (GCA_015476345.1).


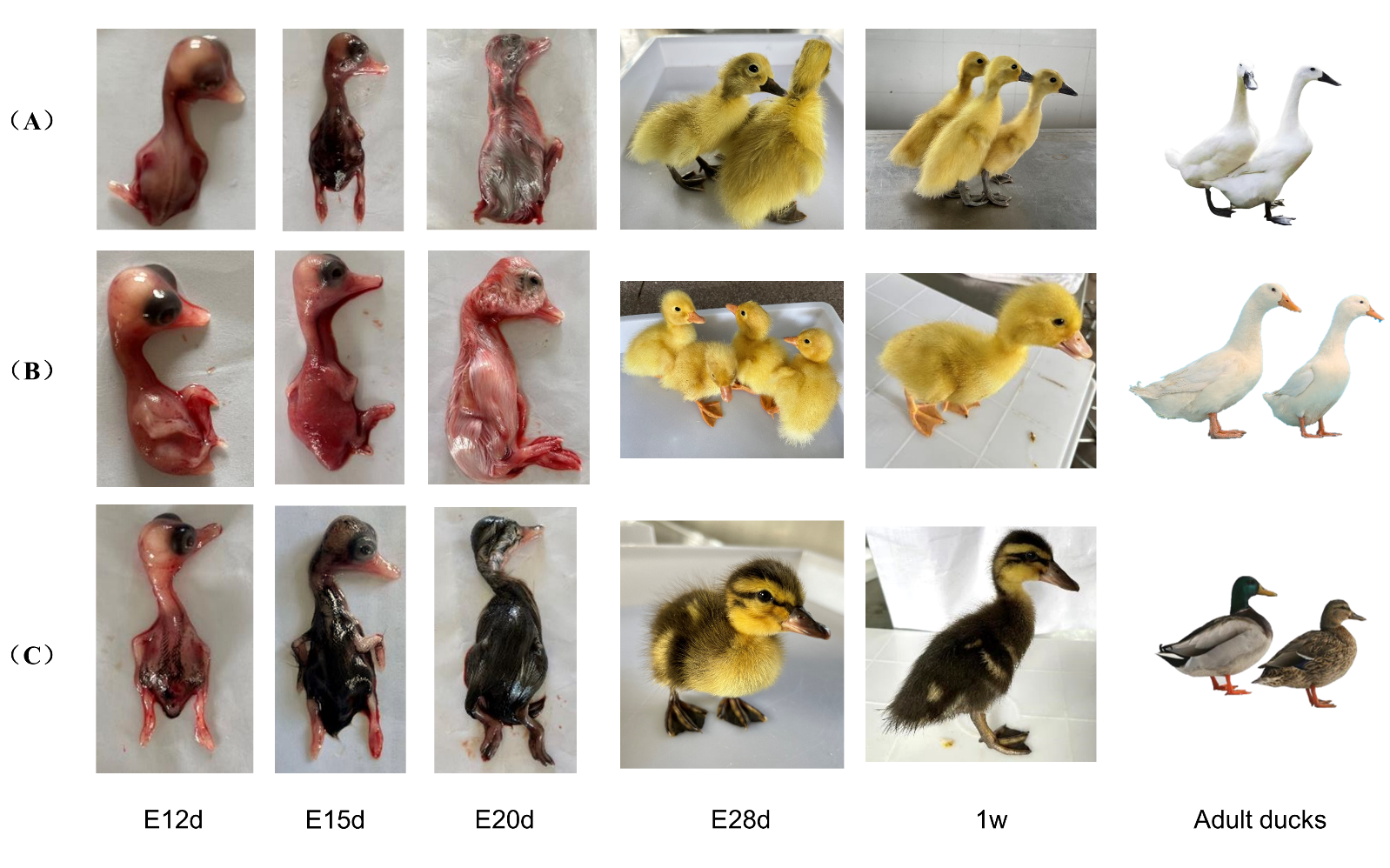


**Figure S9**. Phenotypic characteristics of Liancheng ducks (A), Pekin ducks (B), Mallards (C) during embryonic and postnatal periods. E12d, E15d, E20d, and E28d (also the first day of birth) represent 12, 15, 20, and 28 days of the embryonic period, respectively. Pictures of adult duck from previous paper (Zhou et al., 2018).
