# Supplementary material for: A high-quality assembly revealing the PMEL gene for the unique plumage phenotype in Liancheng ducks
Source: Gigascience. 2025 Jan 13;14:giae114. doi: 10.1093/gigascience/giae114 (PMC11727711; doi:10.1093/gigascience/giae114)
Supplement: giae114_GIGA-D-24-00213_Original_Submission [file giae114_giga-d-24-00213_original_submission.pdf]

GigaScience

A high-quality assembly reveals causal gene for unique plumage phenotype of Liancheng ducks

--Manuscript Draft--

**Manuscript Number:**	GIGA-D-24-00213
**Full Title:**	A high-quality assembly reveals causal gene for unique plumage phenotype of Liancheng ducks
**Article Type:**	Research
**Funding Information:**	National Science Fund for Distinguished Young Scholars (32325047) — Prof. Zhengkui Zhou
**Abstract:**	Background: Plumage color is a notable characteristic that has captured the attention of researchers worldwide, particularly the distinctive white plumage and black markings on the beak and feet of the Liancheng duck. However, the genetic basis of duck plumage coloration continues to present a puzzling enigma. By generating F2 segregating populations from Liancheng (LC) and Pekin (PK) ducks and examining plumage color traits alongside the newly assembled high-quality HiFi genome of the Liancheng duck (GCA_039998735.1). This study aims to identify the genetic basis of white plumage color in Liancheng ducks.<br>Results: A de novo genome of Liancheng duck was assembled, with a genome size of 1.29G. The Scaffold N50 reached 83.98 Mb, and the Contig N50 was 12.17 Mb. Beside the epistatic effect gene MITF, GWAS analysis harbored 0.8Mb candidate region containing PMEL gene, previously uncharacterized in previous duck (Anas platyrhynchos) genome, as the key determinant of white plumage formation in Liancheng ducks. The PMEL gene encodes a pigment cell-specific protein that plays a pivotal role in the formation of fibrillar sheets within the melanosome, the pigment organelle. Meanwhile, we pinpointed two closely linked SNP variations (Chr33:5,303,994A>G; 5,303,997A>G) whose variations may alter PMEL transcription activity.<br>Conclusions: This study constructed a high-quality Liancheng duck genome, and uncovers two causal genes of white plumage color in ducks. Furthermore, it provides valuable insights and guidance for future research on avian plumage coloration.
**Corresponding Author:**	Zhengkui Zhou<br>CAAS IAS: Chinese Academy of Agricultural Sciences Institute of Animal Science<br>Beijing, CHINA
**Corresponding Author Secondary Information:**	
**Corresponding Author's Institution:**	CAAS IAS: Chinese Academy of Agricultural Sciences Institute of Animal Science
**Corresponding Author's Secondary Institution:**	
**First Author:**	Zhen Wang
**First Author Secondary Information:**	
**Order of Authors:**	Zhen Wang
	Zhanbao Guo
	Hongfei Liu
	Tong Liu
	Dapeng Liu
	Simeng Yu
	Hehe Tang
	He Zhang

Powered by Editorial Manager® and ProduXion Manager® from Aries Systems Corporation

	Qiming Mou
	Bo Zhang
	Junting Cao
	Martine Schroyen
	Shuisheng Hou
	Zhengkui Zhou
**Order of Authors Secondary Information:**	
**Additional Information:**	
**Question**	**Response**
Are you submitting this manuscript to a special series or article collection?	No
**Experimental design and statistics**<br><br>Full details of the experimental design and statistical methods used should be given in the Methods section, as detailed in our Minimum Standards Reporting Checklist. Information essential to interpreting the data presented should be made available in the figure legends.<br><br>Have you included all the information requested in your manuscript?	Yes
**Resources**<br><br>A description of all resources used, including antibodies, cell lines, animals and software tools, with enough information to allow them to be uniquely identified, should be included in the Methods section. Authors are strongly encouraged to cite Research Resource Identifiers (RRIDs) for antibodies, model organisms and tools, where possible.<br><br>Have you included the information requested as detailed in our Minimum Standards Reporting Checklist?	Yes
**Availability of data and materials**<br><br>All datasets and code on which the	Yes

conclusions of the paper rely must be either included in your submission or deposited in publicly available repositories (where available and ethically appropriate), referencing such data using a unique identifier in the references and in the "Availability of Data and Materials" section of your manuscript.<br><br>Have you have met the above requirement as detailed in our Minimum Standards Reporting Checklist?	

Powered by Editorial Manager® and ProduXion Manager® from Aries Systems Corporation

Manuscript Click here to access/download;Manuscript;20240611.docx

A high-quality assembly reveals causal gene for unique plumage phenotype of Liancheng ducks

Zhen Wang[1,2], Zhanbao Guo[1], Hongfei Liu[1], Tong Liu[1], Dapeng Liu[1], Simeng Yu[1], Hehe Tang[1], He Zhang[1], Qiming Mou[1], Bo Zhang[1], Junting Cao[1], Martine Schroyen[2], Shuisheng Hou[1], Zhengkui Zhou[1]*

[1] State Key Laboratory of Animal Biotech Breeding, Institute of Animal Science, Chinese Academy of Agricultural Sciences, Beijing, China.

[2] Precision Livestock and Nutrition Unit, Gembloux Agro-Bio Tech, TERRA Teaching and Research Centre, University of Liège, Passage des Déportés 2, Gembloux 5030, Belgium.

Correspondence:

Zhengkui Zhou, zhouzhengkui@caas.cn

Institute of Animal Sciences, Chinese Academy of Agricultural Sciences, No. 2 Yuanmingyuan West Rd., Beijing 100193, China.

Abstract

Background: Plumage color is a notable characteristic that has captured the attention of researchers worldwide, particularly the distinctive white plumage and black markings on the beak and feet of the Liancheng duck. However, the genetic basis of duck plumage coloration continues to present a puzzling enigma. By generating F2 segregating populations from Liancheng (LC) and Pekin (PK) ducks and examining plumage color traits alongside the newly assembled high-quality HiFi genome of the Liancheng duck (GCA_039998735.1). This study aims to identify the genetic basis of white

plumage color in Liancheng ducks.

Results: A *de novo* genome of Liancheng duck was assembled, with a genome size of 1.29G. The Scaffold N50 reached 83.98 Mb, and the Contig N50 was 12.17 Mb. Beside the epistatic effect gene *MITF*, GWAS analysis harbored 0.8Mb candidate region containing *PMEL* gene, previously uncharacterized in previous duck (*Anas platyrhynchos*) genome, as the key determinant of white plumage formation in Liancheng ducks. The *PMEL* gene encodes a pigment cell-specific protein that plays a pivotal role in the formation of fibrillar sheets within the melanosome, the pigment organelle. Meanwhile, we pinpointed two closely linked SNP variations (Chr33:5,303,994A>G; 5,303,997A>G) whose variations may alter *PMEL* transcription activity.

Conclusions: This study constructed a high-quality Liancheng duck genome, and uncovers two causal genes of white plumage color in ducks. Furthermore, it provides valuable insights and guidance for future research on avian plumage coloration.

Keywords: duck, genome assembly, plumage color, *PMEL*, melanin

Background

Plumage color is a visually striking trait found in a diverse range of bird species, making it a valuable topic for investigating natural and artificial selection. Melanin, the primary pigment influencing avian plumage color, typically exists in bird tissues as a mixture of eumelanin and phaeomelanin, resulting in a wide variety of colors [1,2]. Eumelanin deposition in plumage leads to black or brown hues, acting as the predominant pigment in bird feathers [3]. Extensive research on eumelanin and melanin-related genes has significantly enhanced our understanding of avian plumage coloration,

which is a captivating ornamental feature. As a typical bird, ducks (*Anas platyrhynchos*) (NCBI:txid8839) have successfully spread worldwide, with plumage colors ranging from white to black, potentially adapting to different ecological environments. Due to the diverse plumage color patterns seen in ducks, they are a key animal model for studying pigmentation patterns. Despite advancements in understanding the biological and evolutionary aspects of plumage color, the genetic basis of plumage colors in ducks remains poorly understood.

The initial draft of the duck assembly was first reported in 2013 [4]. Subsequent enhancements to the duck genomes have led to a scaffold N50 length of up to 76.3 Mb [5]; however, further improvements in assembly quality are still needed. Previous research on ducks has primarily focused on meat quality [6], adipose deposition [7], and muscle weight [8]. Therefore, an upgraded duck genome is crucial to provide foundational data for future studies on this species. The accuracy of gene localization greatly depends on the quality of the genome assembly [7]. Based on the improvement quality of duck genome, recent studies have identified a 6.6kb intronic insertion in *MITF* that likely affects splicing, leading to white duck down feathers [9], as well as four new single nucleotide polymorphisms in the *MC1R* regulator region associated with black plumage in ducks [10]. To date, HiFi sequencing can provide more accurate, more continuous and more complete genetic information, which is a key enabling research technology [11]. Meanwhile, with advancements in gene chips and genome re-sequencing technologies, genome-wide association studies (GWAS) have become a powerful tool for identifying genetic variations linked to phenotypes. GWAS analysis has uncovered mutations in the *MuPKS* gene responsible for yellow and blue plumage in parrots [12] and pinpointed the *SLC2A11B* gene with a nonsense mutation (W49X)

causing the white eye trait in pigeons [13]. Therefore, based on a high-quality *de novo* genome by HiFi sequencing, this study aims to investigate the genetic mechanisms underlying the distinctive appearance phenotype of Liancheng ducks through a combination of GWAS analysis and functional experiments.

The Liancheng duck is a world famous duck breed by its unique combination of white feathers, but black beak, and feet. This duck species is known for its significant melanin deposition in beak and feet, primarily due to the involvement of eumelanin as the main pigment [14]. The biosynthesis of eumelanin involves three key steps: tyrosinase converts tyrosine to dihydroxyphenylalanine (DOPA) through oxidation, oxidase then changes DOPA into dopaquinone, and finally, dopaquinone undergoes cyclic transformations to produce pigment, leading to melanin formation [15]. This synthetic pathway plays a vital role in eumelanin synthesis, particularly in the pigmentation of skin and hair in animals. In this process, *MITF* is highlighted as a key target of various signal transduction pathways and acts as the main regulator of melanin production. However, the genetic basis of melanin deposition and which genes are involved in the formation of white feathers in Liancheng ducks remain unknown.

The study presents a high-quality *de novo* genome assembly of Liancheng duck, revealing the presence of the *PMEL* gene which was previously thought to be “missing” in ducks. Furthermore, the research identifies two closely linked single nucleotide polymorphisms (SNPs) in the regulatory region that may influence *PMEL* transcription, leading to the white plumage seen in Liancheng ducks. Overall, this investigation offers a valuable genome assembly, molecular markers for duck

breeding, and insights into plumage color patterns in avian species.

Data Description

To understand the genetic basis of unique plumage phenotype of Liancheng ducks, we utilized a high-quality *de novo* assembly of Liancheng duck genome (GCA_039998735.1), and four different plumage color phenotypes from a crossbreeding study involving Liancheng (LC) and Pekin (PK) ducks. We conducted whole-genome resequencing of 366 ducks and aligned the resequencing data to Liancheng duck genome assembly generated in this study. Through transcriptome sequencing analysis across multiple breeds and tissues, we have identified economically significant gene associated with plumage color in Liancheng ducks and elucidated the genetic mechanisms underlying the formation of duck plumage color.

Analyses

A newly assembled high-quality Liancheng duck genome

In order to better analyze the white plumage Liancheng duck, we constructed a *de novo* genome, HiFi long-read sequence data with 88× genome coverage and 584.72 Gb of Hi-C data were generated (Table S1). These datasets were then utilized for assembling the new duck genome (IASCAAS_LianchengWhiteDuck, GCA_039998735.1) (Figure 1A), resulting in a final assembled genome length of 1.29 Gb (Table S2). Chromosome assembly was conducted using hifisam (RRID:SCR_021069). The Scaffold N50 of the *de novo* genome assembly reached 83.98 Mb, while the Contig N50 was 12.17 Mb (Table S2). Collinearity analysis with Mallards (GCA_008746955.3) and the Pekin duck reference genome (GCA_015476345.1) demonstrated the high quality of the

Liancheng duck genome assembly (Figure 1B). The Scaffold N50 length of Liancheng duck in this study was the longest among all previously published duck (*Anas platyrhynchos*) genomes (Figure 1C, 1D, Table S5). A thorough evaluation of the assembly quality post-assembly, using BUSCO (RRID:SCR_015008) [8], revealed 96.7% genome completeness and 96.2% protein integrity (Table S3). After annotating the newly assembled reference genome, 33 chromosomes were identified (Figure 1C, Table S4) and a total of 18,819 genes were annotated (Table S4), marking the first genome annotation for the Liancheng duck.

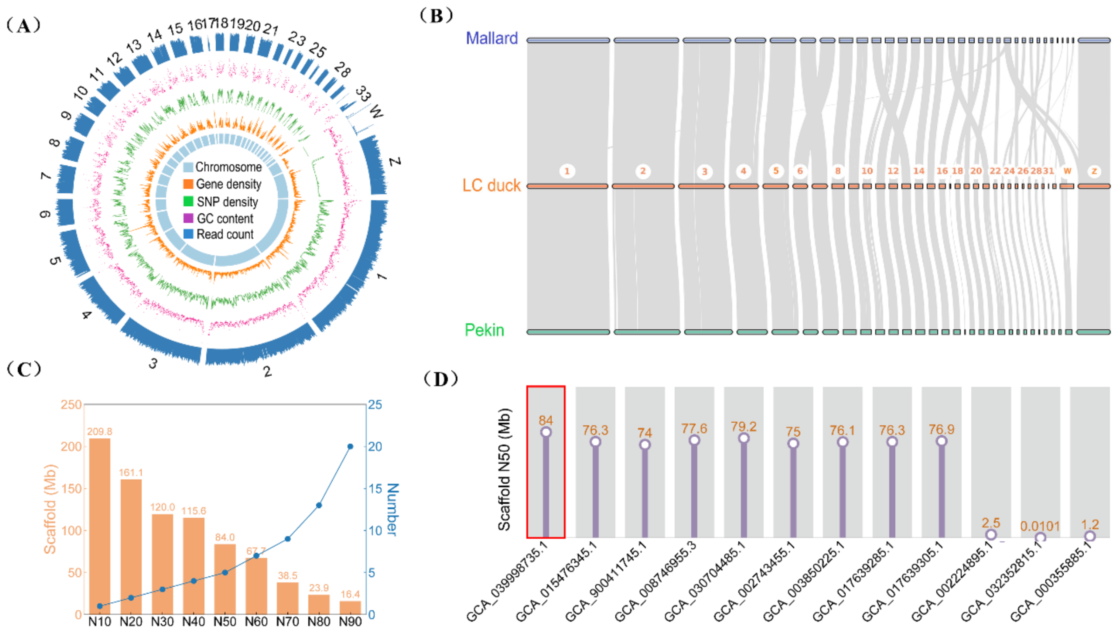

Figure 1. Overview of the assembly quality and characteristics of the Liancheng duck genome. (A) Circular diagram illustrating the characteristics of the genome assembly. The tracks from the inner to outer circles represent the following: chromosomes, gene density, SNP density, GC content (%), and read count. The window size of each circle was 200 kb. (B) Genome synteny analysis between the Liancheng duck and Mallard, Pekin duck. Chromosomes 1-33, as well as two sex chromosomes. (C) Genome statistics for the HiFiasm genome assemblies of the Liancheng duck genome in this study. (D) The length of Scaffold N50 (Mb) of Liancheng duck in this study (GCA_039998735.1)

was compared with all previously published duck (*Anas platyrhynchos*) genomes.

The inheritance of F2 population traits conforms to the law of independent assortment

A crossbreeding study was conducted involving 30 Pekin and 120 Liancheng ducks. All F1 individuals (1,260/1,260) displayed a grey plumage color and pattern. In the F2 populations, four phenotypes were observed: black feather beak and feet (BF), grey feather black beak and feet (GF), white feather black beak and feet (WB), and white feather yellow beak and feet (WY) ducks (Figure 2). The ratio of BF: GF: WB: WY ducks in the F2 population was 3:5.8:3:4.6, which closely matched the theoretical ratio of 3:6:3:4 ($p<0.05$). The phenotypic ratio discovered adheres to Mendel's law of independent assortment of two genes (Table 1 and Figure S1). Recent research has indicated that the *MITF* gene is responsible for white plumage color in ducks. It is hypothesized that the genetic mechanism controlling plumage color in Liancheng ducks is governed by two sites (*Bb* and *Rr* sites), where the allele at the *Rr* site, in interaction with the *Bb* site, determines white plumage in Liancheng ducks (Table 1). Within the F2 population, two alleles (*B*-dominant, enabling melanin synthesis, and *b*, which inhibits melanin synthesis) segregated at the *Bb* locus. Another locus, denoted as *Rr*, possesses two alleles that regulate melanin accumulation in the feather: *R* (dominant, allowing melanin synthesis in the feather) and *r* (repressing melanin synthesis in the feather). The *B* allele at the *Bb* locus displayed an epistatic effect, while the *R* allele at the *Rr* locus demonstrated incomplete dominance effect. The cross between the Liancheng duck (*BBrr*) and Pekin duck (*bbRR*) resulted in the production of grey feather ducks (*BbRr*), with the genotypes of BF, GF, WB, and WY being *B_RR*, *B_Rr*, *B_rr*, and *bb_ _*, respectively (refer to Table 1, Figure 2). Importantly, there was no significant difference observed between the actual and expected numbers within the F2 population

(n=1,281, p=0.345), with a squared value of 3.322 ($\chi2_{0.05(3)}$=7.81, $\chi2_{0.01(3)}$=11.34). The *Bb* locus has previously been identified as the primary gene responsible for white plumage in ducks [2].

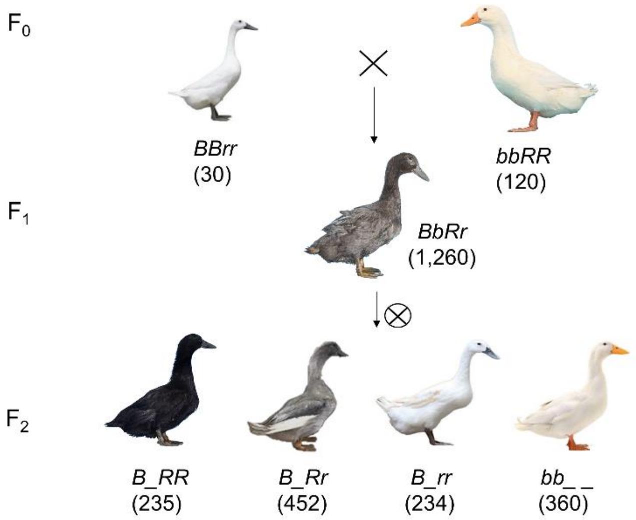

Figure 2. The diagram depicts the segregation of plumage colors in the F2 population. The Liancheng duck showed the white feather black beak and feet (WB) phenotype, whereas the Pekin duck exhibited the white feather yellow beak and feet (WY) phenotype. The F1 generation displayed the gray feather black beak and feet phenotype (GF) with black spots on the feet. In the subsequent F2 generations, ducks were observed with phenotypes including black feather black beak and feet (BF), GF, WB, and WY ducks.

Table 1 The number of F2 populations in different phenotypes and the Chi-squared test.

Comparison	BF duck (*B_RR*)	GF duck (*B_Rr*)	WB duck (*B_rr*)	WY duck (*bb_ _*)	Ratios	χ^2 value	*P*-value
Observed number	235	452	234	360	3:5.8:3:4.6	3.322	0.345 (ns)
Expected number	240	481	240	320	3:6:3:4		

Notes: BF represents ducks with black feathers, beaks, and feet in the F2 population; GF represents ducks with grey feathers, black beaks, and feet in the F2 population; WB represents ducks with white feathers, black beaks, and feet in the F2 population; WY represents ducks with white feathers, yellow beaks, and feet in the F2 population. $\chi^2_{0.05(3)}$=7.81, $\chi^2_{0.01(3)}$=11.34; ns, not significant difference.

Genome-wide association analysis for segregating population duck plumage color

The duck samples were re-sequenced at a depth of 10×. A cohort of 190 ducks from a segregating population derived from Liancheng ducks and Pekin ducks was selected for GWAS analysis. Initially, using the genome of the Liancheng duck as a reference, the study identified two sites controlling the white feather phenotype on Chromosomes 13 and 33 (refer to Figure 3A). This research highlighted a specific gene on Chromosome 13 that regulates melanin synthesis in Liancheng ducks (refer to Figure S2, S3). Since the white plumage phenotype in Liancheng ducks did not show sex-related patterns, cytoplasmic inheritance considerations for white plumage were deemed unnecessary. Subsequently, the *Rr* gene was pinpointed to the 5.24-5.32Mb region of Chromosome 33 in Liancheng ducks (refer to Figure 3B), which contains potential candidate genes like *PMEL*, *RAB5B*, *IKZF4*, *ERBB3*, *PA2G4*, *ZC3H10*, and *ESYT1* (refer to Figure 3C). Within the candidate region (Chr33: 5.24-5.32 Mb), four minimal recombination haplotypes were identified based on the parents and segregating populations from 117 SNPs with an Fst >0.8 (PK vs LC ducks). Only the haplotypes in block 4 (Chr33: 5,303,111-5,304,416, 101,305 bp) located upstream of the *PMEL* gene corresponded to the observed phenotypes (refer to Figure 3D). Additionally, a significantly high peak in the Fst value was observed in the *RR* vs *rr* duck populations within the selected candidate region, while no peak was seen in *rr* vs *rr* duck populations (refer to Figure 3E).

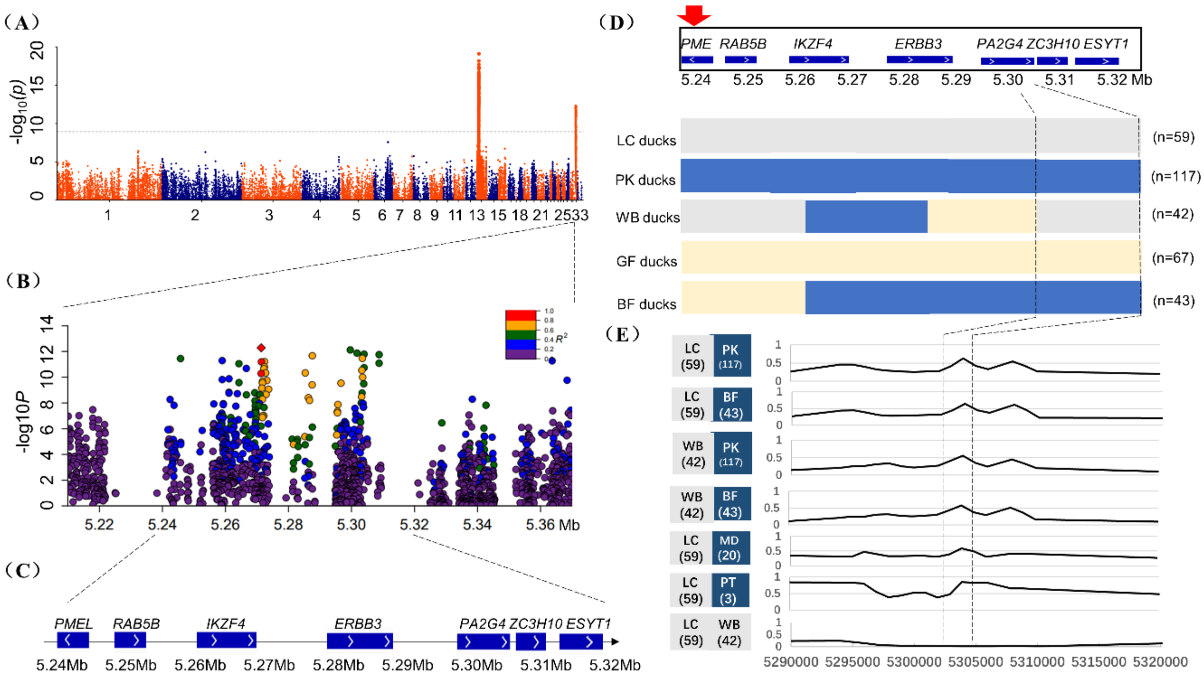

Figure 3. Screening for the candidate region associated with the white plumage of Liancheng ducks involved a GWAS on a cohort of 190 ducks from a cross between Pekin ducks (PK) and Liancheng ducks (LC). (A) Manhattan plot was created to show the genetic effects on plumage color. (B) Locuszoom results highlighted site on chromosome 33 (5.21-5.37 Mb) linked to white plumage in Liancheng ducks. Genotypic SNPs were identified based on linkage imbalance values compared to the leading SNP in the intercross population duck (Chr33: 5,303,413). (C) Candidate genes in the region of interest (5.24-5.32 Mb) were identified, with white and black arrows indicating gene orientation and Chromosome 33 direction, respectively. (D) Identity by Descent (IBD) analysis used color schemes to refine candidate regions, with blue for Pekin ducks and black plumage ducks, grey for Liancheng ducks and white plumage black beak ducks, and yellow for grey plumage ducks (LC vs PK, Fst> 0.8). (E) Genome divergence analysis among six duck breeds, including LC vs PK ducks, Black feather ducks (BF), Mallards (MD), and Putian (PT) ducks within the candidate region (Chr33: 5.29-5.32 Mb), averaged Fst values over SNPs in 10kb increments in each comparison group.

***PMEL* causes melanin deposition in duck plumages**

The region on Chromosome 13 was found to encompass the *MITF* gene in the GWAS analysis (refer to Figure S2). Upon comparison with the previous Pekin duck genome assembly (GCA_003850225.1), a 6.6 kb insertion within the *MITF* gene was identified, showing a strong correlation with melanin synthesis in ducks (refer to Table S6). Subsequent GWAS analysis, after removing the *MITF* signal, revealed a single signal on Chromosome 33 (refer to Figure S4). Expression analysis of various genes, including *PMEL*, *RAB5B*, *IKZF4*, *ERBB3*, *PA2G4*, *ZC3H10*, and *ESYT1*, was conducted on hair follicles from white-, grey-feather, and black-feather ducks. Results indicated that only the *PMEL* gene showed significant differential expression (refer to Figure 4A, 4B), with higher expression levels in black plumage ducks compared to grey plumage ducks (-Log10(p)>30). RNA-seq results showed no expression of the *PMEL* gene in hair follicles of white plumage ducks (refer to Figure 4C, 4D). Other genes within the GWAS candidate range (Chr33: 5.24-5.32Mb) were excluded due to similar gene expression levels in different plumage populations or inconsistent gene expression patterns with melanin regulation. qPCR results confirmed *PMEL* as the *Rr* gene (refer to Figure 4C). Additionally, *PMEL* gene expression correlated with the plumage color phenotype of Liancheng ducks at various developmental stages, with high expression levels observed in skin tissue (refer to Figure 4B, S5). Notably, the *PMEL* gene exhibited an elevated average GC content of 72.4% (refer to Figure S6), which explains the lack of a signal in the previous Pekin duck reference genome. This underscores the importance of establishing a high-quality genome for Liancheng ducks.

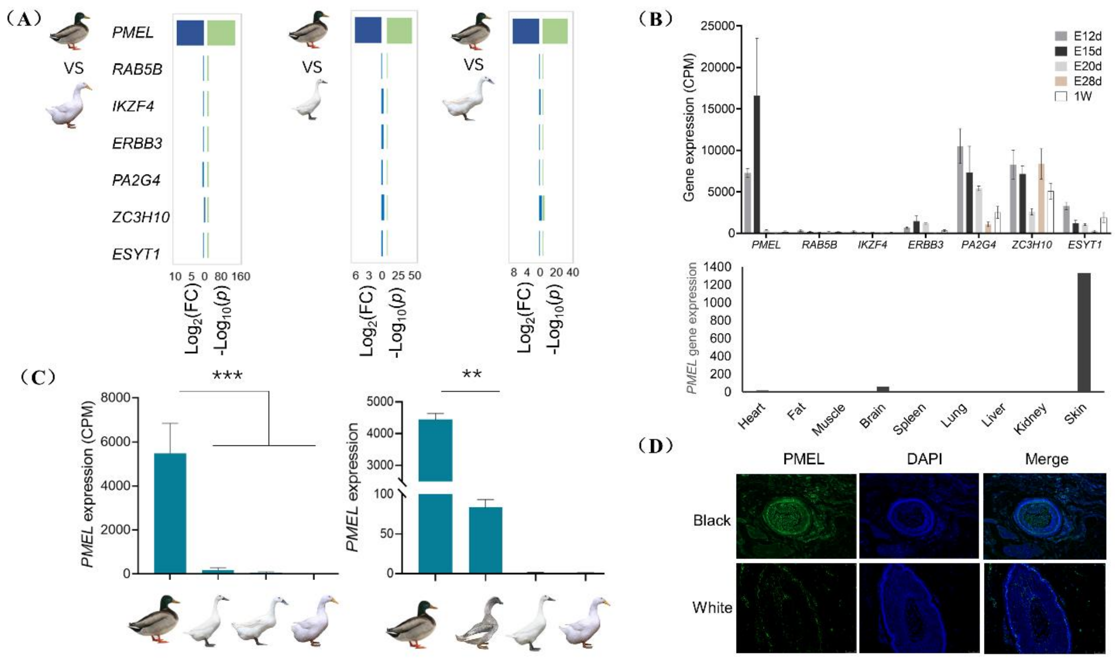

Figure 4. Identification of the candidate gene for white plumage in Liancheng ducks (A) Gene expression of seven GWAS candidate region genes (*PMEL*, *RAB5B*, *IKZF4*, *ERBB3*, *PA2G4*, *ZC3H10*, *ESYT1*) in 1-week-old hair follicle samples of white and black-feather ducks, with three replicates per sample. Log_2(FC) values were used to analyze gene expression differences, and values where -$Log_{10}(p) > 30$ were shown in green. (B) Analysis of the expression levels of the seven candidate genes in skin tissues of Liancheng ducks at different developmental stages. E12d, E15d, E20d, and E28d (also the first day of birth) represent 12, 15, 20, and 28 days of the embryonic period, respectively. (C) CPM and qPCR results of *PMEL* expression in 1-week-old hair follicle samples of ducks. Data were presented as mean ± SD (three replicates per group). (D) Immunofluorescence results showing PMEL distribution in hair follicle samples of black- and white-plumage ducks. Black (Mallards) and White (Liancheng ducks).

The *Rr* variation was fine mapped to *PMEL* upstream regulatory region

The results of the IBD analysis indicated that only the haplotypes in block 4 (Chr33: 5,303,111-

5,304,416, 101,305 bp) located upstream of the *PMEL* gene were associated with the observed phenotypes (refer to Figure 3D). Furthermore, a significantly high peak in the Fst value was observed in the *RR* vs *rr* duck populations within the selected candidate region (refer to Figure 3E), providing supporting evidence for this region (Chr33: 5,303,111-5,304,416, 101,305 bp) as the locus of interest for the *Rr* site. Among the identified candidate variations in this region, site with genotype distributions inconsistent with the intercross generation phenotype, as well as one copy number variation (CNV) (refer to Figure S7 and S8), were initially excluded. Applying a threshold of Fst > 0.8 (PK vs LC ducks), only 12 SNP variants and 2 Indels were retained (refer to Table S7). It is noteworthy that all 12 SNP variations were found in the upstream regulatory region of the *PMEL* gene (Chr33: 5,239,969-5,244,318). Upon careful consideration, two SNPs (Chr33: 5,303,994A>G; 5,303,997A>G) were identified as the potential causal variants across all duck breeds in this study (Table S7). Intriguingly, these two SNPs were observed to be in complete linkage disequilibrium. Additionally, the Hi-C results illustrated that the *PMEL* gene and its upstream region, which encompassed the two candidate SNPs, were situated within a topologically associated domain (TAD) region (refer to Figure 5).

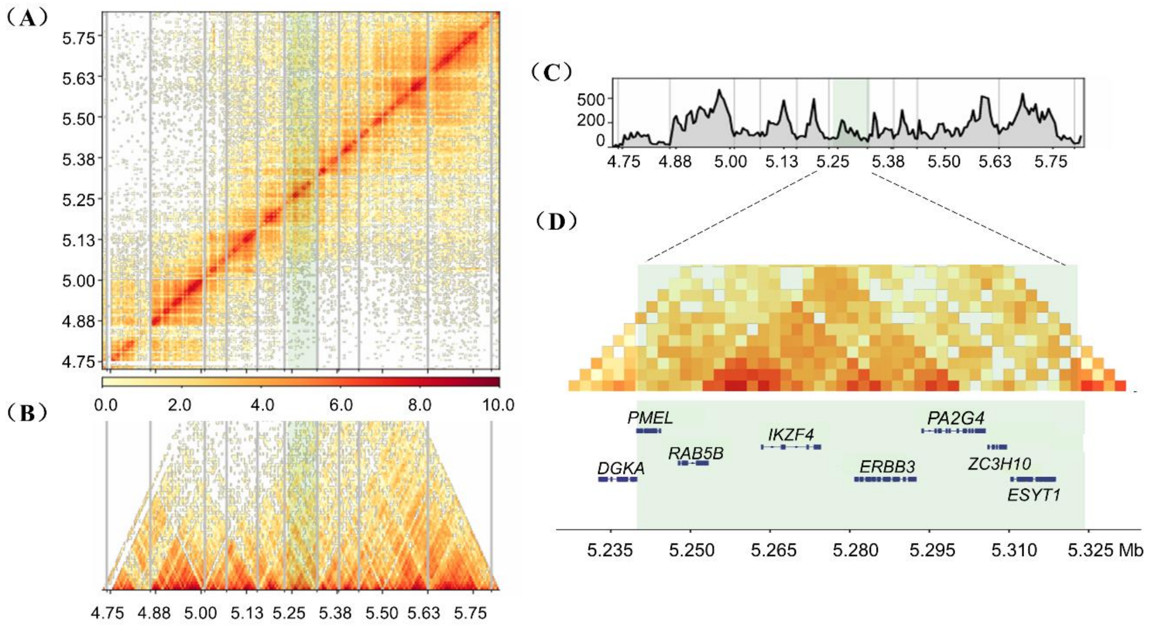

Figure 5. Chromosome interaction mapping (Hi-C) result of the end of chromosome 33 (4.75-5.8Mb) in skin fat of Liancheng ducks. (A) Log_2(interaction matrix) analysis of Chr33 (resolution:5kb). Strong contacts were shown in red, and weak contacts were shown in white. The heat map showed a normalized contact matrix in 5kb bins. Light green indicated the *Rr* site candidate region by GWAS analysis. (B) Triangular result of Log_2 (interaction matrix) of Chr33 (4.75-5.75Mb) and the (C) topologically associated domain (TAD)-like and boundary-like regions were identified with the default algorithm built in HiCPlotter at a resolution of 5kb. (D) Log_2 (interaction matrix) of Chr33 (5.235-5.325Mb). Candidate regions identified from Chr33:5.24-5.32Mb by GWAS result and genes within it (light green regions).

Functional analysis of two candidate SNPs

Considering the location of the two candidate SNPs (SNP1 and SNP2) (Chr33: 5,303,994A>G; 5,303,997A>G) in the noncoding upstream region of the *PMEL* gene, we conducted an assessment of their promoter and enhancer effects using pGL3 luciferase vectors (refer to Figure 6A, 6B). Both pGL3 vectors (pGL3-basic-white and pGL3-basic-black) with inserts showed minimal luciferase

activity in duck embryo fibroblast (DEF) cells and Human melanoma cells (A375) (refer to Figure 6C), indicating no promoter activity at these SNP sites. However, for enhancer activity, the vectors with inserts (pGL3-promoter-white and pGL3-promoter-black) displayed significantly different luciferase activity in DEF cells and A375 cells ($p<0.01$) and SNP2 (Chr33: 5,303,997A>G) revealed that both pGL3-promoter-white-1mut and pGL3-promoter-white-2mut exhibited higher luciferase activity than pGL3-promoter-white (refer to Figure 6D), indicating a synergistic enhancement activity by the black alleles of variations SNP1 and SNP2.

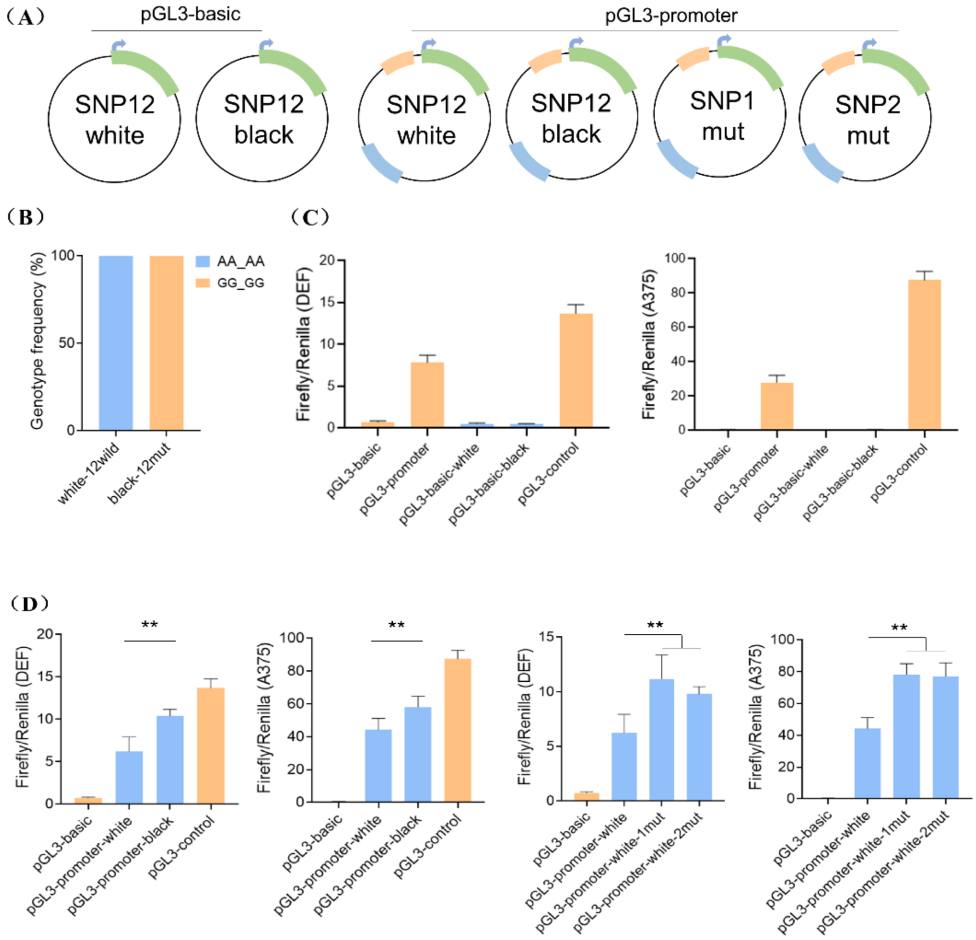

Figure 6. Functional analysis of the candidate variation controlling the white plumage phenotype of Liancheng duck. (A) Diagram of six pGL3 vectors for luciferase reporter gene experiment. Candidate SNP1 and SNP2 (Chr33:5,303,994 and 5,303,997) of Liancheng duck and Pekin duck

were inserted into empty pGL3-basic and pGL3- promoter vectors. pGL3-basic, pGL3-promoter and pGL3-control were used as negative and positive control group. Insertion fragment were marked in yellow so as to verify the promoter activity. The blue box represented insertion fragment of the PGL3-promoter to verify enhancer activity. Additionally, vectors also contained the green box (luciferase reporter gene) and the blue arrow (indicate transcription site). (B) Genotype distribution of SNP1 (Chr33:5,303,994A>G), SNP2 (Chr33:5,303,997A>G) in Liancheng duck (AA_AA, n=59) and Pekin duck (GG_GG, n=117). (C) Validation of promoter activity of homozygous wildtype (AA_AA) and homozygous mutation (GG_GG) in DEF cells and A375 cells. (D) Enhancer activity of AA_AA, GG_GG, GG_AA, and AA_GG vectors in DEF cells and A375 cells. Each group had 8 replicates (**$p<0.01$).

Analysis conducted on the JASPAR transcription prediction website (https://jaspar.elixir.no/) revealed that multiple transcription factors may bind differently to sequences surrounding candidate SNPs located at Chr33: 5,303,944-5,304,098. The results suggested that variations in SNP1 and SNP2 could impact the binding of various transcription factors, as illustrated in Figure S9. Furthermore, differential expression of the sox5 transcription factor was observed in the hair follicles of white- and black-feathered ducks. These findings led to the hypothesis that variations in SNP1 and SNP2 could potentially be key mutations responsible for the white feather phenotype.

Discussion

The genetic basis of white plumage color in Liancheng ducks

The white plumage phenotype is a common trait observed in various avian species such as chickens

[16,17], ducks [2,9], peafowl [18], geese [19], and other birds. Particularly, the white plumage phenotype in ducks is highly valued for textile purposes, leading researchers to investigate its genetic basis. Extensive research has focused on the unique appearance of Liancheng ducks [20-22], yet the genetic mechanisms behind their plumage color remain unclear. This study categorized plumage color subjectivity (white, grey, black) and establishes it as a classic Mendelian trait in the F2 duck population (refer to Figure 2). A genetic hypothesis is proposed for the white feather phenotype in Liancheng ducks (refer to Figure 2,3). The mating of Liancheng duck (*BBrr*) and Pekin duck (*bbRR*) produced grey feathered ducks (*BbRr*) in the F1 generation, with genotypes *B_RR*, *B_Rr*, *B_rr*, *bb_ _* observed in the F2 population. Our findings suggested that the inheritance of plumage color phenotype in Liancheng ducks was likely governed by two autosomal genes, independent of sex chromosomes. Despite using the Pekin duck reference genome (IASCAAS_PekingDuck_PBH1.5, GCA_003850225.1), our GWAS analysis identified three signals (refer to Figure S3), which we believed may be due to incomplete assembly. To further investigate the white feather phenotype of Liancheng ducks, we first established a high-quality genome for Liancheng duck (refer to Figure 1). This new reference genome has a size of 1.29 Gb, with contig and scaffold N50 values of 12.17 and 83.98Mb, respectively (refer to Table S2). Comparative analysis with previously published duck (*Anas platyrhynchos*) genomes revealed a significant improvement in assembly quality with the new Liancheng duck reference genome (refer to Table S4). GWAS results from this genome highlighted two distinct signals on chromosomes 13 and 33 (refer to Figure 3A), supporting our initial hypothesis. Our data showed two strong signals on autosomal chromosomes associated with the white feather phenotype. The presence of specific alleles (*Bb* and *Rr*) at different autosomal sites in Pekin and Liancheng ducks determined the unique

appearance of Liancheng ducks (refer to Figure 2). These findings emphasize the enhanced contiguity and completeness achieved with the newly generated genome compared to previously published duck reference genomes.

MITF acts as a key regulator of melanin synthesis in ducks

MITF, a gene implicated in familial melanoma and Waardenburg syndrome, produces various isoforms through alternative promoters with shared coding exons but distinct amino termini [23]. While *MITF* variants are known to influence melanin regulation, the regulation of these isoforms remained unclear. *MITF-M* isoforms have been shown to regulate white coloration in the fur of dogs [24], llamas [25], and mice [26]. Studies on *MITF-M*-null mice revealed a lack of neural crest-derived melanocytes in certain tissues, yet pigmentation was maintained in other areas [26]. In ducks, we discovered the expression of two *MITF* isoforms, *MITF-B* and *MITF-M*, with only the latter being crucial for melanin synthesis in duck plumage [2,9].

Based on GWAS analysis, it was confirmed that the *MITF* gene acts as an epistatic gene controlling melanin synthesis in Liancheng ducks, aligning with previous research findings [21]. Specifically, a 6.6 kb insertion within the *MITF* gene demonstrated a strong correlation with melanin production in ducks, with homozygous individuals displaying white coloring and heterozygous or non-insertion individuals showing coloration (Table S6). *MITF* plays a critical role in regulating melanocyte development, function, and survival, influencing melanin formation and potentially impacting cell transformation [27]. This highlights the significant regulatory role of *MITF* in melanin synthesis in Liancheng ducks and underscores its importance as a key genetic factor in pigmentation.

***PMEL* causes melanin deposition in duck plumages**

PMEL, a type I transmembrane transport glycoprotein, is synthesized in the endoplasmic reticulum and plays a crucial role in amyloid fiber formation during stages I and II of melanosome formation in the L-DOPA pathway [28,29]. After synthesis, PMEL is transported to melanosomes where it undergoes proteolytic processing to form fibrils [30]. These fibrils act as a scaffold for the deposition of melanin pigments, catalyzed by enzymes like tyrosinase [31,32]. Mutations in the *PMEL* gene can lead to abnormalities in melanosome formation and melanin deposition, impacting plumage coloration in various bird species, including chickens [16,33], Junco hyemalis [34], and Indian peafowl [18]. However, the association between the *PMEL* gene and duck plumage color phenotype has not been previously explored. Within the GWAS candidate region on Chr33, the *PMEL* gene was found to be significantly differentially expressed between hair follicles of white- and black-feathered ducks (p<0.001). Immunofluorescence results indicated high expression of the PMEL protein in hair follicles of black and grey plumage ducks, contrasting with low expression in white plumage ducks. To date, only 21 bird species have annotated the *PMEL* gene among 120 bird genomes (Table S8). We have provided the first annotation of the *PMEL* gene in the newly sequenced genome of ducks (Figure S6, Table S9), previously believed to lack this gene in ducks.

Previous research has demonstrated that *PMEL* is transcriptionally regulated by *MITF* in both melanocytes and melanomas. Alterations in *MITF* expression levels have been shown to impact endogenous *PMEL* regulation in melanoma cells [35]. However, the specific interplay between these two genes in determining the plumage color of Liancheng ducks requires further investigation.

Overall, within the melanin synthesis pathway of duck hair follicles, *MITF* functions as a key gene that governs melanin production. Subsequent inactivation of the *PMEL* gene, situated in the hair follicles, results in the distinctive white feather and black feathers observed in Liancheng ducks.

Two fully linked SNP variations synergistically associated with white feather of Liancheng ducks

In the candidate region identified through GWAS analysis, we investigated a total of 12 SNPs, 2 Indel variations, and 1 CNV variation (refer to Table S7; Figures S7 and S8). Among these variants, only 2 SNPs (Chr33:5,303,994A>G and 5,303,997A>G) were found to be consistently associated with the observed plumage color phenotypes across multiple breeds. Experimental data from promoter activity assays suggested that these SNPs may not act as promoters controlling *PMEL* gene expression (refer to Figure 6C). Instead, our genetic findings indicated that these two linked SNP variations, located in the upstream region of the *PMEL* gene, exhibited functional enhancer activity that might remotely regulate *PMEL* gene expression (refer to Figures 5 and 6). This remote regulation likely impacts *PMEL* gene expression, resulting in the black plumage coloration seen in ducks, which aligns with the melanin phenotype of duck feathers.

Remote regulation elements are thought to interact with target promoters through physical proximity [36,37], but the exact impact of this proximity on function remains uncertain. In our research, we identified potential regions encompassing *PMEL* and two candidate SNPs within a single topologically associated domain (TAD) region (Figure 5), suggesting that this area may be part of a genomic region with frequent interactions. The process of loop extrusion not only promotes

interactions within the TAD but also shields the TAD from external influences [38]. Furthermore, enhancer-promoter interactions may intensify during mammalian development [39], potentially accounting for the variation in plumage colors of Liancheng ducks from embryonic to postnatal stages (Figure S5). We also noted that only sox5, the predicted transcription factor, displayed varying expression levels in hair follicles of different plumage colors (Figure S9). However, it is worth mentioning that other transcription factors could also play a role in the regulatory mechanism. Additionally, the gray plumage, an intermediate phenotype observed in our study, may be linked to a haploinsufficiency effect [40].

Materials and Methods

Ducks and sampling

All animal procedures in this study were carried out in accordance with the guidelines for the care and use of experimental animals set by the Chinese Academy of Agricultural Sciences (IAS2022-105). Approval for this study was granted by the ethics committee of the Chinese Academy of Agricultural Sciences. A blood sample from a female Liancheng duck was used for genome *de novo* assembly. A total of 366 parents and intercross population duck plumage color phenotypes were recorded from a previous gradient consanguinity population [6], which included 117 Pekin ducks, 59 Liancheng ducks, 38 white feathered ducks with yellow beaks and feet (WY), 42 white feathered ducks with black beaks and feet (WB), 67 gray plumage ducks with black beaks and feet (GF), and 43 black feathered ducks with black beaks and feet (BF) underwent whole-genome resequencing (Table S10). Additionally, genome data from 23 black ducks, comprising 20 Mallards (MD) and 3 Putian (PT) ducks, were used for comparative analysis [2].

Hair follicle tissues from 1-week-old black- and grey-feathered ducks, Liancheng ducks, and Pekin ducks were collected and stored at -80°C for RNA or protein extraction. These tissues were then used for transcriptome and proteome analyses following animal welfare ethics standards. Skin tissues from Liancheng ducks were collected at embryonic stages of 12 days, 15 days, 20 days, 28 days, and one week after birth, with each sample group consisting of three biological replicates, except for the four 12-day embryo samples (Table S11). Additionally, heart, fat, muscle, brain, spleen, lung, liver, kidney, and skin tissues from an 8-week-old black-feathered Mallard duck were collected and processed for RNA-seq analysis [2].

Genome assembly and Gene annotation

To conduct the *de novo* assembly of the Liancheng duck genome, we utilized a combination of PacBio long-read HiFi sequencing and chromosome interaction mapping (Hi-C) technologies. The project used long-read and long-HiFi (RRID:SCR_021966) sequencing data (PacBio, Beijing, China) to assemble the species [41]. Using Pacbio Sequel II platform sequencing, the HiFi-asm (https://github.com/chhylp123/hifiasm) versions (v0.19.3 NOV-2023), assembling, genome Contig. Through Juicer (NOV-2023), Hi-C data of quality control was compared back to the assembled Contig sequence [42,43]. By calculating the Unique Mapped Read Pairs in the Hi-C sequencing data to the assembled genome, the valid and Invalid Hi-C Interaction Pairs were calculated Pairs) ratio to complete Hi-C library evaluation and comparative analysis. The 3D-DNA software (RRID:SCR_017227) (NOV-2023) was used to divide, sequence and Orient the genome sequences [44]. The results of BUSCO assembly were evaluated [45].

The structure prediction of genes was performed by combining evidence-based prediction and *de novo* prediction. We used RNA-seq data as evidence files for evidence-based annotation through *Maker*. With the comparison information between RNA-seq data and genome, PASA (Program to Assemble Spliced Alignments) (RRID:SCR_014656) was used to construct the training model of *Augustus* (http://bioinf.uni-greifswald.de/augustus/), and then *de novo* annotation was performed through *Augustus*. Then we integrated the results of *Maker* and *De novo* based on the principle that evidence results were better than prediction results. Finally, genes with repeat sequences less than 50%, protein length greater than 50 amino acids, and expression greater than 0 in at least one transcriptome sequencing sample were retained. Based on sequence similarity, direct homologous genes can be found, and the function of new genes can be predicted by proteins with known functions. Functional annotation of genes in GO, KEGG, NR, swissprot and other databases was performed by eggNOG software (RRID:SCR_002456) and blast sequence comparison software.

Whole-genome resequencing

A total of 366 DNA samples, consisting of Pekin ducks, Liancheng ducks, and intercross populations were selected for resequencing (Table S10). The genome data of 23 black-feather ducks includes 20 Mallards, and 3 Putian ducks. DNA eligible samples were identified for further testing. Libraries were established for the samples, and the average Reads length was 150 bp. The average original read sequence coverage for ducks was 10×. This depth ensures the accuracy of variation calling and genotyping, and met the requirements of genetic analysis [6]. Following the elimination of read pairs containing adapter sequences, a quality assessment of the raw reads was performed utilizing

TRIMMOMATIC (RRID:SCR_011848) (version 0.36) [46]. Subsequently, the high-quality reads were aligned to the Liancheng duck reference genome (IASCAAS_LianchengWhiteDuck, GCA_039998735.1) using the Burrow–Wheeler Aligner (bwaaln) [47] with default settings. SNPs underwent filtration based on the following criteria: (i) SNPs were required to demonstrate a minor allele frequency (MAF) >0.05 and a maximum allele frequency <0.99; (ii) the maximum missing rate was set at <0.7; and (iii) SNPs were restricted to possessing only two alleles.

Genome-wide association analysis

The GWAS was conducted utilizing a mixed linear model implemented through the EMMAX program (RRID:SCR_024012) [48] with genome-wide single nucleotide polymorphism data and the plumage color phenotype observed in 366 individuals from the resequencing population. The analytical model adopted the form y = Xb + Ga + e, where y represented the phenotypic value (plumage color of each duck), X denoted the matrix corresponding to fixed effects, and b signified the magnitude of the fixed effects. The fixed effects encompassed sex-related influences. G represented the genetic matrix associated with population kinship, while e stood for the random residual. Principal component analysis (PCA) was executed using all SNPs, with the top three components incorporated as fixed effects within the mixed model to adjust for population stratification. A Bonferroni correction threshold of 0.01/N ($-\log_{10}P$ = 8.95) was established to pinpoint significant site in the GWAS findings, where N indicated the total number of whole-genome SNPs (8,887,194). IBD analysis was used to carry out the fine-mapped analyses in 328 ducks. IBD fragments can reflect the genetic relationship between individuals and detect trait variation, and the correlation between IBD fragments and phenotype was used to detect whether

there were regions affecting trait variation in the genome [48,49]. To conduct this analysis, the filtered SNPs (n= 117) reached the standard allele frequency difference (ΔAF) >0.8 between the Liancheng ducks and Pekin ducks. In the candidate region (Chr33:5.1-5.5Mb), we identified four recombination breakpoints across the 36 SNPs, and we subsequently classified the segregating individuals by using the four recombinant breakpoints.

Transcriptome sequencing and analysis

12 hair follicle tissues of black-and grey-feather ducks, Liancheng ducks, and Pekin ducks at 1 week after birth, and the heart, fat, muscle, brain, spleen, lung, liver, kidney, and skin tissues of an 8-week-old black-feathered mallard were collected. Total amounts of RNA from above tissues were first extracted using Vazyme's Trizol reagent. The RNase enzyme was inactivated by the addition of pyrrole diethyl carbonate. Final RNA-seq libraries for the experiment totaled 12 and were sequenced on an Illumina platform utilizing the 150-bp paired-end sequencing module. The effective read length was increased by illumina sequencing. Per library, the average production was 6 Gb. Using TopHat (RRID:SCR_013035), RNA-seq paired-end reads from each library were mapped to the reference genome of the aforementioned Liancheng duck. Different gene expression levels were obtained based on the Liancheng duck reference genome. After that, expression was calculated by using TopHat, and read counts per million (CPM) values for the genes were obtained by running htseq-count [6,50].

qPCR for transcript of *PMEL* in hair follicles

cDNA of hair follicles, including black-, grey-feather ducks, Liancheng ducks, Pekin ducks, was

reversely transcribed with HiScript III All-in-one RT SuperMix Perfect for qPCR (Vazyme). The reverse transcription quantitative PCR (qPCR) was conducted in a total volume of 10 μl including 5 μl Tap Pro Universal SYBR qPCR Master Mix (Vazyme), 0.8 μl forward and reverse primers, 0.5μl cDNA, 3.7 μl distilled water. β-actin was selected as the internal reference gene. Primer sequence was shown in Table S12. All of the reactions were run in triplicate. The relative mRNA expression levels were calculated by using the normalized relative quantification method, followed by the $2^{-\Delta\Delta CT}$ calculation [51].

Immunofluorescence experiment

The hair follicles of ducks with black and white feathers were embedded in paraffin, fixed in 4% buffered paraformaldehyde, and sectioned into 5 μm slices. Following fixation overnight at 4°C, it was ensured that the duration of fixation did not exceed 24 hours to maintain tissue integrity effectively. Subsequently, the sections were dewaxed and rehydrated to enhance adhesion and facilitate dewaxing processes. The sections underwent antigen retrieval by incubating in EDTA (Servicebio) at 100°C for 20 minutes. Post-retrieval, the antigens were fixed in Tris-EDTA and the sections were washed thrice with phosphate-buffered saline (PBS) followed by an additional wash. For immunostaining, the sections were incubated with the PMEL antibody (Abclonal) at 4°C for 12 hours after pre-treatment with 3% bovine serum albumin (Solarbio) for 30 minutes. The PMEL antibody utilized was an anti-rabbit antibody. Subsequently, the hair follicle tissues exhibiting various plumage colors were counterstained with DAPI. Experimental outcomes were documented through photographic and record-keeping procedures.

Causative mutation screening and identification

Based on the Liancheng duck genome, we compared the candidate regions (Chr33:5.24-5.32Mb) among 117 Pekin ducks, 59 Liancheng ducks, and 152 intercross population ducks. Within the IBD candidate region, all candidate SNP sites, Indels, and CNV variations were identified. Multi-alleles were disregarded in further analyses, as the phenotype separation ratio in the F2 population suggested that a single allele determined the plumage color phenotype. Only the genotypes of the variants associated with phenotypes with a reliability exceeding 80% in Liancheng ducks and Pekin ducks were considered as candidate causative variations. Among the candidate IBD fragments, only the regions where genotypes and phenotypes were consistent with each other were further investigated as candidate regions. To eliminate variations with a lower likelihood of being causally involved, we implemented the following three steps. Firstly, we utilized the genotype and phenotype information from the 366 parents and intercross ducks to exclude SNPs based on the standard Fst < 0.8. Secondly, we utilized the genotypes and phenotypic information of 117 Pekin ducks, 59 Liancheng ducks, 20 Mallards, 42 WB ducks, 43 BF ducks, and 3 Putian ducks. The high Fst values shared by Liancheng ducks and the other breeds were chosen as further candidate regions. Thirdly, only genotypes consistent with the phenotypes from the Mallard and Putian duck populations were considered as candidate causative mutations. Finally, all indel and CNV variations within the candidate region were also analyzed and excluded using the aforementioned method. Only variations whose genotype and phenotype were consistent across multiple duck breeds could be regarded as causative variations for the *Rr* locus.

Hi-C sequencing and analysis

Skin fat tissue samples from a Liancheng duck were subjected to cross-linking in 20 ml of fresh ice-cold nuclear isolation buffer. The chromatin extraction methodology mirrored previous protocols [2]. Subsequently, the purified DNA underwent digestion and fragmentation using the *DpnII* restriction enzyme, followed by repair of DNA ends. Biotin-labeled DNA fragments were then isolated using streptavidin C1 beads. Library preparation was conducted utilizing an Illumina TruSeq DNA Sample Prep Kit following the manufacturer's guidelines. Quality assessment of the Hi-C library was performed through TA cloning. Sequencing of the Hi-C libraries took place on an Illumina HiSeq X Ten system (RRID:SCR_016385). The Hi-C experiments were independently conducted twice, with the experimental and sequencing procedures executed by Gene Technology Co., Ltd., located in Beijing, China.

Raw Hi-C data underwent processing to eliminate low-quality reads and trim adapters using TRIMMOMATIC (RRID:SCR_011848) [46]. All reads were trimmed to 50 bp, and clean reads were aligned to the duck genome employing a two-step approach integrated into the HiC-Pro (RRID:SCR_017643) software [52]. Reads of low mapping quality, multiple mappings, and singletons were excluded. Subsequently, uniquely mapped reads were consolidated into a single file. Read pairs that did not align proximal to a restriction site or failed to meet the anticipated fragment size after shearing were filtered out. Further filtering steps were implemented to discard read pairs stemming from invalid ligation products, such as dangling-end and self-ligation products, as well as PCR artifacts. The remaining valid read pairs were categorized into intrachromosomal and interchromosomal pairs. Contact maps were generated using chromosome bins of uniform sizes ranging from 3 kb to 1 Mb. The initial contact maps were normalized utilizing a sparse-based

iteration correction method within HiC-Pro and visualized using HiCPlotter [53]. Finally, regions resembling topologically associated domains (TADs) and boundaries were delineated employing the default algorithm within HiCPlotter at a resolution of 5 kb [53].

Structural variation detection

In the GWAS candidate region, we analyzed all CNV structural variations in randomly selected populations, including Pekin ducks, Liancheng ducks, WB ducks, GF ducks, and BF ducks. Ducks from these different feather groups were randomly chosen to represent their respective feather groups. As no structural variations other than CNV were identified, we utilized CNVcaller (RRID:SCR_015752) software (version 0.11) to investigate and genotype all individual CNVs [54]. The CNV calling and genotyping procedures were consistent with those described in previous studies [55]. Log_2 fold change values reflected the ratio of sequencing read depths in the 1,000bp window region to that of Pekin duck reads. Within the candidate region of 5.24-5.32Mb, only CNV variants consistent with the phenotype were considered as candidate variations. Therefore, we examined the distribution of all CNV genotypes in the aforementioned populations. A copy number of 1 indicated a normal diploid state, 0.5 denoted loss of heterozygosity, 0 signified homozygous loss, 1.5 indicated heterozygous duplication, and 2 represented homozygous duplication. An absolute copy number exceeding 2 indicated complex duplications [55].

Luciferase reporter assay

Four haplotypes of candidate variations SNP1 and SNP2 and their upstream and downstream were cloned into the pGL3-basic and pGL3-promoter vector. In this study, the *XhoI* and *KpnI* sites as the

insertion sites were cloned in pGL3-basic for analyzing promoter activity, and *BamHI* and *SalI* sites were selected in pGL3-promoter vector for analyzing enhancer activity. A375 and DEF cells were plated in 48-well plates, with a density of $0.5x10^5$ per well, and cultured in cell dishes for 24h in DMEM (Pricella, China), mixed with 10% FBS (Pricella, China). A375 and DEF cells were transfected with Lipofectamine 8000 (Beyotime), ensuring that each well contained the same 237.5ng of DNA, which was 4 sequences containning SNP1 and SNP2 sites. At the same time, 12.5ng of pRL-TK vector was added to each well. According to the instructions, after lysis of cells, cell lysate was collected and luciferase activity was measured. The instrument was the Veritas Microplate Luminometer (Promega). Each sample was repeated three times, and Renilla fluorescence was used to normalize firefly fluorescence [56,57].

Data Availability

The whole-genome sequence data reported in this article have been deposited in the NCBI under accession number No. PRJNA1107839. The resequencing raw data have been deposited in the NCBI SRA under accession No. PRJNA844232. The transcriptomic raw data have been deposited in the NCBI under accession No. PRJNA1109286.

Additional Files

Supplementary Figure 1. Phenotypic characteristics of 1,281 F2 ducks from Liancheng and Pekin ducks.

Supplementary Figure 2. Screening for the candidate region associated with the white plumage of Liancheng ducks by GWAS in 190 ducks from a cross between Liancheng and Pekin ducks.

Supplementary Figure 3. Manhattan plot showing the genetic effects on the plumage color according to a GWAS in ducks from a cross of Liancheng and Pekin ducks based on previous reference genome of Pekin duck (GCA_015476345.1).

Supplementary Figure 4. Manhattan plot showing the genetic effects on the plumage color according to a GWAS from a cross of Liancheng and Pekin ducks except WY ducks based on Liancheng duck genome (GCA_039998735.1).

Supplementary Figure 5. Phenotypic characteristics of Liancheng ducks, Pekin ducks, Mallards during embryonic and postnatal periods.

Supplementary Figure 6. The collinearity analysis of comparison between the *PMEL* gene and its upstream and downstream 100kb region in Liancheng duck and other birds.

Supplementary Figure 7. Illustration of the read depth analysis that confirmed the copy number variations on GWAS candidate region (Chr33: 5.24-5.32Mb).

Supplementary Figure 8. Genotypes of candidate CNV variation (Chr33:5,282,001- 5,284,500) in different plumage color populations.

Supplementary Figure 9. Heatmap cluster analysis of transcription factors.

Supplementary Table 1. Table Summary of Hi-C reads mapping results.

Supplementary Table 2. The Genome Scaffolds and Contigs information of Liancheng duck genome (GCA_039998735.1).

Supplementary Table 3. Complete evaluation table of Liancheng duck genome BUSCOs.

Supplementary Table 4. Summary of chromosome regions on the genome.

Supplementary Table 5. Assembly statistics of the Liancheng duck genome and previous duck genome.

Supplementary Table 6. Frequency distribution of 6.6kb insertion within *MITF* gene in duck population.

Supplementary Table 7. Genotypic distribution of SNP candidate variations in different duck breeds based on reference genome Liancheng duck.

Supplementary Table 8. All birds with *PMEL* genes in their avian genomes.

Supplementary Table 9. The blast results of sequence identity between the newly annotated mRNA sequence of *PMEL* gene in Liancheng duck and other birds.

Supplementary Table 10. List of 366 duck genome resequencing used in the study.

Supplementary Table 11. List of 37 ducks for RNA-seq used in the study.

Supplementary Table 12. The information on the primers used for qPCR.

Abbreviations

LC: Liancheng duck; PK: Pekin duck; WB: The white feather black beak and feet duck; GF: The gray feather black beak and feet duck; BF: The black feather black beak and feet duck; WY: The white feather yellow beak and feet duck; MD: Mallard; PT: Putian ducks; BLAST: Basic Local Alignment Search Tool; bp: Base pairs; kb: Kilobase pairs; Mb: Megabase pairs; Gb: Gigabase pairs; FC: Fold change; IBD: Identity By Descent; BUSCO: Benchmarking Universal Single Copy Orthologs; BWA: Burrows Wheeler Aligner; NCBI: National Center for Biotechnology Information; PacBio: Pacific Biosciences; HiFi: High Fidelity reads; Hi-C: High-throughput/resolution chromosome conformation capture; RNA-seq: RNA sequencing; SNP: Single nucleotide polymorphism; SRA: Sequence Read Archive; PASA: Program to Assemble Spliced Alignments; Go: Gene Ontology; KEGG: Kyoto Encyclopedia of Genes and Genomes; NR: Non-Redundant

Protein Database; eggNOG: Evolutionary genealogy of genes: Non-supervised Orthologous; MAF: Minor allele frequency; PCA: Principal component analysis; ΔAF: Allele frequency difference; CPM: Counts per million; Indel: Insertion and deletion; CNV: Copy number variation; Fst: Fixation index; GC: Guanine-cytosine; GWAS: Genome-wide association study; TAD: Topologically associated domain; DEF: Duck embryo fibroblast; A375: Human melanoma cells.

Funding

This work was supported by grants from the National Science Fund for Distinguished Young Scholars (32325047), the Innovation Program of Chinese Academy of Agricultural Sciences(CAAS--SCAB-202302), the China Agriculture Research System of MOF and MARA (CARS-42-05), and the National Key R&D Program of China (2022YFF1000102). The authors were grateful to Shandong Rongda Agricultural Development Co., Ltd for their help in this sample collection.

Competing Interests

The authors have declared no competing interests.

Ethics Statement

All animals used in the study were treated following the guidelines for the experimental animals established by the Council of China Animal Welfare. Protocols of the experiments were ap-proved by the Science Research Department of the Institute of Animal Sciences, Chinese Academy of Agricultural Sciences (CAAS) (Beijing, China).

Authors' Contributions

Z.Zhou. and S.Hou conceived the project, designed the research, and managed the project. Z.Guo., Z.Wang., H.Tang., H.Zhang. constructed the population. T.Liu., D.Liu. and Z.Wang. collected the phenotype data. Z.Wang., S.Yu. and H.Liu. performed the genome assembly. Z.Wang., B.Zhang. and J.Cao. performed the experiments. Z.Wang., H.Liu., D.Liu. and Q.Mou. performed bioinformatics analysis. Z.Wang., M.Schroyen. and Z.Zhou. wrote the manuscript.

References

1.Ito S, Wakamatsu K. Quantitative analysis of eumelanin and pheomelanin in humans, mice, and other animals: a comparative review. Pigment Cell Res. 2003;16:523-531.

2.Zhou Z, Li M, Cheng H, et al. An intercross population study reveals genes associated with body size and plumage color in ducks. Nat Commun. 2018;9:2648.

3.Land EJ, Riley PA. Spontaneous redox reactions of dopaquinone and the balance between the eumelanic and phaeomelanic pathways. Pigment Cell Res. 2000;13:273-277.

4.Huang Y, Li Y, Burt DW, et al. The duck genome and transcriptome provide insight into an avian influenza virus reservoir species. *Nat Genet*. 2013;**45**:776-783.

5.Li J, Zhang J, Liu J, et al. A new duck genome reveals conserved and convergently evolved chromosome architectures of birds and mammals. *Gigascience*. 2021;**10**:giaa142.

6.Liu D, Zhang H, Yang Y, et al. Metabolome-Based Genome-Wide Association Study of Duck Meat Leads to Novel Genetic and Biochemical Insights. *Adv Sci*. 2023;**10**:e2300148.

7.Zhu F, Yin ZT, Wang Z, et al. Three chromosome-level duck genome assemblies provide insights into genomic variation during domestication. *Nat Commun*. 2021;**12**:5932.

8.Yu S, Liu Z, Li M, et al. Resequencing of a Pekin duck breeding population provides insights into the genomic response to short-term artificial selection. *Gigascience*. 2023;**12**:giad016.

9.Wang K, Hua G, Li J, et al. Duck pan-genome reveals two transposon insertions caused bodyweight enlarging and white plumage phenotype formation during evolution. *IMeta*. 2024;**3**,e154.

10.Liu H, Xi Y, Tang Q, et al. Genetic fine-mapping reveals single nucleotide polymorphism mutations in the MC1R regulatory region associated with duck melanism. *Mol Ecol*. 2023;**32**:3076-3088.

11.Wenger, A.M., Peluso, P., Rowell, W.J. et al. Accurate circular consensus long-read sequencing improves variant detection and assembly of a human genome. *Nat Biotechnol.* 2019; **37**:1155–1162.

12.Cooke TF, Fischer CR, Wu P, et al. Genetic Mapping and Biochemical Basis of Yellow Feather Pigmentation in Budgerigars. *Cell*. 2017;**171**:427-439.e21.

13.Si S, Xu X, Zhuang Y, et al. The genetics and evolution of eye color in domestic pigeons (Columba livia). *PLoS Genet*. 2021;**17**:e1009770.

14.Wang Z, Guo Z, Mou Q, et al. Unique feather color characteristics and transcriptome analysis of hair follicles in Liancheng White ducks. *Poult Sci*, 2024;**103**:103794.

15.Haase E, Ito S, Wakamatsu K. Influences of sex, castration, and androgens on the eumelanin and pheomelanin contents of different feathers in wild mallards. *Pigment Cell Res*. 1995;**8**:164-170.

16.Keeling L, Andersson L, Schütz KE, et al. Chicken genomics: feather-pecking and victim pigmentation. *Nature*. 2004;**431**:645-646.

17.Gunnarsson U, Kerje S, Bed'hom B, et al. The Dark brown plumage color in chickens is caused

by an 8.3-kb deletion upstream of SOX10. *Pigment Cell Melanoma Res*. 2011;**24**:268-274.

18.Liu S, Chen H, Ouyang J, et al. A high-quality assembly reveals genomic characteristics, phylogenetic status, and causal genes for leucism plumage of Indian peafowl. *Gigascience*. 2022;**11**:giac018.

19. Xi Y, Wang L, Liu H, et al. A 14-bp insertion in endothelin receptor B-like (EDNRB2) is associated with white plumage in Chinese geese. *BMC Genomics*. 2020;**21**:162.

20.Gong Y, Yang Q, Li S, et al. Grey plumage colouration in the duck is genetically determined by the alleles on two different, interacting loci. *Anim Genet*. 2010;**41**:105-108.

21.Yang L, Mo C, Shen W, et al. The recessive C locus in the MITF gene plays a key regulatory role in the plumage colour pattern of duck (Anas platyrhynchos). *Br Poult Sci*. 2019;**60**:105-108.

22. Wang L, Yang L, Yang S, et al. Identification of genes associated with feather color in Liancheng white duck using FST analysis. *Anim Genet*. 2022;**53**:518-521.

23.Karlsson EK, Baranowska I, Wade CM, et al. Efficient mapping of mendelian traits in dogs through genome-wide association. *Nat Genet*. 2007;**39**:1321-1328.

24.Baranowska Körberg I, Sundström E, Meadows JR, et al. A simple repeat polymorphism in the MITF-M promoter is a key regulator of white spotting in dogs. *PLoS One*. 2014;**9**:e104363.

25.Anello M, Daverio MS, Silbestro MB, Vidal-Rioja L, Di Rocco F. Characterization and expression analysis of KIT and MITF-M genes in llamas and their relation to white coat color. *Anim Genet*. 2019;**50**:143-149.

26.Flesher JL, Paterson-Coleman EK, Vasudeva P, et al. Delineating the role of MITF isoforms in pigmentation and tissue homeostasis. *Pigment Cell Melanoma Res*. 2020;**33**:279-292.

27.Hershey CL, Fisher DE. Genomic analysis of the Microphthalmia locus and identification of the MITF-J/Mitf-J isoform. *Gene*. 2005;**347**:73-82.

28.Kerje S, Sharma P, Gunnarsson U, et al. The Dominant white, Dun and Smoky color variants in chicken are associated with insertion/deletion polymorphisms in the PMEL17 gene. *Genetics*. 2004;**168**:1507-1518.

29.Batai K, Cui Z, Arora A, et al. Genetic loci associated with skin pigmentation in African Americans and their effects on vitamin D deficiency. *PLoS Genet*. 2021;**17**:e1009319.

30.Watt B, Tenza D, Lemmon MA, et al. Mutations in or near the transmembrane domain alter PMEL amyloid formation from functional to pathogenic. *PLoS Genet*. 2011;**7**:e1002286.

31.Hurbain I, Geerts WJ, Boudier T, et al. Electron tomography of early melanosomes: implications for melanogenesis and the generation of fibrillar amyloid sheets. *Proc Natl Acad Sci U S A*. 2008;**105**:19726-19731.

32.Watt B, van Niel G, Raposo G, Marks MS. PMEL: a pigment cell-specific model for functional amyloid formation. *Pigment Cell Melanoma Res*. 2013;**26**:300-315.

33.Deng Y, Qu X, Yao Y, Li M, He C, Guo S. Investigating the impact of pigmentation variation of breast muscle on growth traits, melanin deposition, and gene expression in Xuefeng black-bone chickens. *Poult Sci*. 2024;**103**:103691.

34.Abolins-Abols M, Kornobis E, Ribeca P, et al. Differential gene regulation underlies variation in melanic plumage coloration in the dark-eyed junco (Junco hyemalis). *Mol Ecol*. 2018;**27**:4501-4515.

35.Falletta P, Bagnato P, Bono M, et al. Melanosome-autonomous regulation of size and number: the OA1 receptor sustains PMEL expression. *Pigment Cell Melanoma Res*. 2014;**27**:565-579.

36.Soldner F, Stelzer Y, Shivalila CS, et al. Parkinson-associated risk variant in distal enhancer of α-synuclein modulates target gene expression. *Nature*. 2016;**533**:95-99.

37.Bergman DT, Jones TR, Liu V, et al. Compatibility rules of human enhancer and promoter sequences. *Nature*. 2022;**607**:176-184.

38.Hung TC, Kingsley DM, Boettiger AN. Boundary stacking interactions enable cross-TAD enhancer-promoter communication during limb development. *Nat Genet*. 2024;**56**:306-314.

39.Chen Z, Snetkova V, Bower G, et al. Increased enhancer-promoter interactions during developmental enhancer activation in mammals. *Nat Genet*. 2024;**56**:675-685.

40.Billiard S, Castric V, Llaurens V. The integrative biology of genetic dominance. *Biol Rev Camb Philos Soc*. 2021;**96**:2925-2942.

41.Cheng H, Concepcion GT, Feng X, Zhang H, Li H. Haplotype-resolved de novo assembly using phased assembly graphs with hifiasm. *Nat Methods*. 2021;**18**:170-175.

42.Durand NC, Shamim MS, Machol I, et al. Juicer Provides a One-Click System for Analyzing Loop-Resolution Hi-C Experiments. *Cell Syst*. 2016;**3**:95-98.

43.Zheng Z, Lai Z, Wu B, et al. The first high-quality chromosome-level genome of the Sipuncula Sipunculus nudus using HiFi and Hi-C data. *Sci Data*. 2023;**10**:317.

44.Dudchenko O, Batra SS, Omer AD, et al. De novo assembly of the Aedes aegypti genome using Hi-C yields chromosome-length scaffolds. *Science*. 2017;**356**:92-95.

45.Manni M, Berkeley MR, Seppey M, Zdobnov EM. BUSCO: Assessing Genomic Data Quality and Beyond. *Curr Protoc*. 2021;**1**:e323.

46.Bolger AM, Lohse M, Usadel B. Trimmomatic: a flexible trimmer for Illumina sequence data. *Bioinformatics*. 2014;**30**:2114-2120.

47.Li H, Durbin R. Fast and accurate short read alignment with Burrows-Wheeler transform. *Bioinformatics*. 2009;**25**:1754-1760.

48.Kang HM, Sul JH, Service SK, et al. Variance component model to account for sample structure in genome-wide association studies. *Nat Genet*. 2010;**42**:348-354.

49.Price AL, Patterson NJ, Plenge RM, Weinblatt ME, Shadick NA, Reich D. Principal components analysis corrects for stratification in genome-wide association studies. *Nat Genet*. 2006;**38**:904-909.

50.Anders S, Pyl PT, Huber W. HTSeq--a Python framework to work with high-throughput sequencing data. *Bioinformatics*. 2015;**31**:166-169.

51.Schmittgen TD, Livak KJ. Analyzing real-time PCR data by the comparative C(T) method. *Nat Protoc*. 2008;**3**:1101-1108.

52.Servant N, Varoquaux N, Lajoie BR, et al. HiC-Pro: an optimized and flexible pipeline for Hi-C data processing. *Genome Biol*. 2015;**16**:259.

53.Akdemir KC, Chin L. HiCPlotter integrates genomic data with interaction matrices. *Genome Biol*. 2015;**16**:198.

54.Guo Y, Gu X, Sheng Z, et al. A Complex Structural Variation on Chromosome 27 Leads to the Ectopic Expression of HOXB8 and the Muffs and Beard Phenotype in Chickens. *PLoS Genet*. 2016;**12**:e1006071.

55.Wang X, Zheng Z, Cai Y, et al. CNVcaller: highly efficient and widely applicable software for detecting copy number variations in large populations. *Gigascience*. 2017;**6**:1-12.

56.Chen L, Gu X, Huang X, et al. Two cis-regulatory SNPs upstream of ABCG2 synergistically cause the blue eggshell phenotype in the duck. *PLoS Genet*. 2020;**16**:e1009119.

57.Liu H, Hu J, Guo Z, et al. A single nucleotide polymorphism variant located in the cis-regulatory region of the ABCG2 gene is associated with mallard egg colour. *Mol Ecol*. 2021;**30**:1477-1491.

Click here to access/download
Supplementary Material
Supplementary Figures.docx

Supplementary Tables

Click here to access/download
Supplementary Material
Supplementary Tables.xlsx
