# Supplementary material for: A high-quality assembly revealing the PMEL gene for the unique plumage phenotype in Liancheng ducks
Source: Gigascience. 2025 Jan 13;14:giae114. doi: 10.1093/gigascience/giae114 (PMC11727711; doi:10.1093/gigascience/giae114)
Supplement: giae114_GIGA-D-24-00213_Revision_1 [file giae114_giga-d-24-00213_revision_1.pdf]

GigaScience

A high-quality assembly reveals PMEL gene for unique plumage phenotype of Liancheng ducks

--Manuscript Draft--

**Manuscript Number:**	GIGA-D-24-00213R1
**Full Title:**	A high-quality assembly reveals PMEL gene for unique plumage phenotype of Liancheng ducks
**Article Type:**	Research
**Funding Information:**	National Science Fund for Distinguished Young Scholars (32325047) — Prof. Zhengkui Zhou
**Abstract:**	Background: Plumage color is a notable characteristic that has captured the attention of researchers worldwide, particularly the distinctive white plumage and black markings on the beak and feet of the Liancheng duck. However, the genetic basis of duck plumage coloration continues to present a puzzling enigma. By generating F2 segregating populations from Liancheng (LC) and Pekin (PK) ducks and examining plumage color traits alongside the newly assembled high-quality HiFi genome of the Liancheng duck (GCA_039998735.1). This study aims to identify the genetic basis of white plumage color in Liancheng ducks.<br>Results: A de novo genome of Liancheng duck was assembled, with a genome size of 1.29G. The Scaffold N50 reached 83.98 Mb, and the Contig N50 was 12.17 Mb. Beside the epistatic effect gene MITF, GWAS analysis harbored 0.8Mb candidate region containing PMEL gene, previously uncharacterized in previous duck (Anas platyrhynchos) genome, as the key determinant of white plumage formation in Liancheng ducks. The PMEL gene encodes a pigment cell-specific protein that plays a pivotal role in the formation of fibrillar sheets within the melanosome, the pigment organelle. Meanwhile, we pinpointed two closely linked SNP variations (Chr33:5,303,994A>G; 5,303,997A>G) whose variations may alter PMEL transcription activity.<br>Conclusions: This study constructed a high-quality Liancheng duck genome, and uncovers two causal genes of white plumage color in ducks. Furthermore, it provides valuable insights and guidance for future research on avian plumage coloration.
**Corresponding Author:**	Zhengkui Zhou<br>CAAS IAS: Chinese Academy of Agricultural Sciences Institute of Animal Science<br>Beijing, CHINA
**Corresponding Author Secondary Information:**	
**Corresponding Author's Institution:**	CAAS IAS: Chinese Academy of Agricultural Sciences Institute of Animal Science
**Corresponding Author's Secondary Institution:**	
**First Author:**	Zhen Wang
**First Author Secondary Information:**	
**Order of Authors:**	Zhen Wang
	Zhanbao Guo
	Hongfei Liu
	Tong Liu
	Dapeng Liu
	Simeng Yu
	Hehe Tang
	He Zhang

Powered by Editorial Manager® and ProduXion Manager® from Aries Systems Corporation

	Qiming Mou
	Bo Zhang
	Junting Cao
	Martine Schroyen
	Shuisheng Hou
	Zhengkui Zhou
**Order of Authors Secondary Information:**	
**Response to Reviewers:**	Dear editors,<br><br>Thank you for handing our manuscript. We also thank two reviewers for their critical comments and valuable suggestions, which have significantly improved our manuscript. In the revised version, we have adjusted the logic of the results section, conducted additional analyses.<br><br>Below, we offer a point-by-point response and indicate the corresponding modified sections in the revised manuscript, which are marked in green.<br><br>We hope that our current manuscript addresses the reviewer's concerns.<br><br>Sincerely,<br><br>All authors<br><br>Reviewer 1#<br>The manuscript "A high-quality assembly reveals causal gene for unique plumage phenotype of Liancheng ducks" focuses on uncovering the genetic basis of the distinctive white plumage seen in Liancheng ducks. The study aims to understand the genetic determinants of plumage color in Liancheng ducks, a breed known for its unique combination of white feathers with black beaks and feet. The primary focus is on identifying genetic variants that contribute to this phenotype.<br><br>The authors constructed a de novo genome assembly for the Liancheng duck using PacBio HiFi sequencing and Hi-C data to achieve this. The assembly achieved significant quality, with a genome size of approximately 1.29 Gb. Additionally, a genome-wide association study (GWAS) was conducted using a mixed linear model to identify significant single nucleotide polymorphisms (SNPs) associated with the white plumage phenotype. The analysis involved 364 individuals from an F2 population derived from crossbreeding Liancheng and Pekin ducks.<br><br>The GWAS identified a 0.8 Mb candidate region on Chromosome 33 containing the PMEL gene, a key determinant of plumage color. This region includes two closely linked SNPs (Chr33:5,303,994A>G; 5,303,997A>G) that may alter PMEL gene transcription. Functional assays were performed to validate the impact of these SNPs on gene expression, suggesting that they significantly influence the white plumage phenotype.<br><br>The study presents the first high-quality genome assembly of the Liancheng duck and identifies novel genetic variants in the PMEL gene associated with white plumage. These findings provide valuable insights into the genetic mechanisms underlying plumage coloration and offer promising practical implications for selective breeding and conservation efforts.<br><br>Specific comments:<br><br>1) Discuss how your findings compare with the genetic and molecular basis of feather diversity as highlighted in these review papers:<br><br>Terrill RS, Shultz AJ. Feather function and the evolution of birds. Biol Rev Camb Philos Soc. 2023 Apr;98(2):540-566. doi: 10.1111/brv.12918. Epub 2022 Nov 24.

Powered by Editorial Manager® and ProduXion Manager® from Aries Systems Corporation

Ng CS, Li WH. Genetic and Molecular Basis of Feather Diversity in Birds. Genome Biol Evol. 2018 Oct 1;10(10):2572-2586. doi: 10.1093/gbe/evy180.

Boer EF, Van Hollebeke HF, Shapiro MD. Genomic determinants of epidermal appendage patterning and structure in domestic birds. Dev Biol. 2017 Sep 15;429(2):409-419.

Chen CF, Foley J, Tang PC, Li A, Jiang TX, Wu P, Widelitz RB, Chuong CM. Development, regeneration, and evolution of feathers. Annu Rev Anim Biosci. 2015;3:169-95. doi: 10.1146/annurev-animal-022513-114127.
Response: Thank you for your comments. We have revised the discussion section and added those references to highlight the different and innovation of our new findings.

“Feather phenotype is a complex trait composed of a series of stratified modules [81]. Birds can be highly decorated with distinct and colorful pigmentation patterns, which are used to attract a mate or to hide from or frighten a potential predator. Melanin plays an important role in the formation of feather pigments, which is achieved through the regulation of the presence, distribution, and differentiation of these melanocytes. Recent studies have reported that the variation of MITF, PMEL, TYR, EDNRB2, SLC45A2, MC1R genes and Agouti signaling protein can regulate the production of feather melanin in ducks [2, 82]. However, the role of PMEL gene in feather melanin formation in ducks has not been confirmed. Our study is the first to report the role of the PMEL gene in pigment formation in duck feathers and provide valuable insights into the genetic mechanisms underlying plumage coloration, as well as promising practical implications for selective breeding and conservation efforts” (Line 583-593 in the revised manuscript).

2) Integrate these studies to provide a broader context for the role of PMEL in different bird species and its implications for plumage color:

Yuan Z, Zhang X, Pang Y, Qi Y. Association analysis of PMEL gene expression and single nucleotide polymorphism with plumage color in quail. Anim Biotechnol. 2023 Dec;34(9):5001-5010. doi: 10.1080/10495398.2023.2221697.

Ishishita S, Takahashi M, Yamaguchi K, Kinoshita K, Nakano M, Nunome M, Kitahara S, Tatsumoto S, Go Y, Shigenobu S, Matsuda Y. Nonsense mutation in PMEL is associated with yellowish plumage colour phenotype in Japanese quail. Sci Rep. 2018 Nov 13;8(1):16732. doi: 10.1038/s41598-018-34827-4.

Liu X, Zhou R, Peng Y, Zhang C, Li L, Lu C, Li X. Feather follicles transcriptome profiles in Bashang long-tailed chickens with different plumage colors. Genes Genomics. 2019 Nov;41(11):1357-1367. doi: 10.1007/s13258-018-0740-y.

Zheng X, Zhang B, Zhang Y, Zhong H, Nie R, Li J, Zhang H, Wu C. Transcriptome analysis of feather follicles reveals candidate genes and pathways associated with pheomelanin pigmentation in chickens. Sci Rep. 2020 Jul 21;10(1):12088. doi: 10.1038/s41598-020-68931-1.

Hua G, Chen J, Wang J, Li J, Deng X. Genetic basis of chicken plumage color in artificial population of complex epistasis. Anim Genet. 2021 Oct;52(5):656-666. doi: 10.1111/age.13094.

Weng Z, Xu Y, Li W, Chen J, Zhong M, Zhong F, Du B, Zhang B, Huang X. Genomic variations and signatures of selection in Wuhua yellow chicken. PLoS One. 2020 Oct 23;15(10):e0241137. doi: 10.1371/journal.pone.0241137.

Heo S, Cho S, Dinh PTN, Park J, Jin DH, Cha J, Kim YK, Koh YJ, Lee SH, Lee JH. A genome-wide association study for eumelanin pigmentation in chicken plumage using a computer vision approach. Anim Genet. 2023 Jun;54(3):355-362. doi: 10.1111/age.13303.

Abolins-Abols M, Kornobis E, Ribeca P, Wakamatsu K, Peterson MP, Ketterson ED, Milá B. Differential gene regulation underlies variation in melanic plumage coloration in the dark-eyed junco (Junco hyemalis). Mol Ecol. 2018 Nov;27(22):4501-4515. doi:

10.1111/mec.14878.

Yuan H, Zhang X, Zhang Q, Wang Y, Wang S, Li Y, Zhang Y, Jing J, Qiu J, Wang Z, Leng L. Comparative transcriptome profiles of Lindian chicken eyelids identify melanin genes controlling eyelid pigmentation. Br Poult Sci. 2019 Feb;60(1):15-22. doi: 10.1080/00071668.2018.1544414.
Response: Thank you for your suggestions. References are included in the discussion section of the revised manuscript. “PMEL, a type I transmembrane transport glycoprotein, is synthesized in the endoplasmic reticulum and plays a crucial role in amyloid fiber formation during stages I and II of melanosome formation in the L-DOPA pathway [63, 64]. After synthesis, PMEL is transported to melanosomes, where it undergoes proteolytic processing to form fibrils [65]. These fibrils act as a scaffold for the deposition of melanin pigments, catalyzed by enzymes like tyrosinase [66, 67]. Mutations in the PMEL gene can lead to abnormalities in melanosome formation and melanin deposition, impacting plumage coloration in various bird species, including chickens [38, 68], Junco hyemalis [69], Japanese quail [70] and Indian peafowl [40]. To date, only 21 bird species have annotated the PMEL gene among 120 bird genomes (Table S11). However, the association between the PMEL gene and duck plumage color phenotype has not been previously explored.

In this study, the PMEL gene was found to be significantly differentially expressed between the feather bulb specimens of white- and black-feathered ducks (ANOVA, $p<0.001$). Immunofluorescence results indicated high expression of the PMEL protein in feather follicle specimens of black and grey plumage ducks, contrasting with low expression in white plumage ducks. Many studies suggested that the deposition of feather melanin may involve the PMEL gene [69, 71, 72, 73]. Meanwhile, the PMEL gene is also implicated in the formation of white feathers in quail [74] and in the white feathers of chickens at the hatch stage [75]. Endogenous PMEL expression is regulated by MITF, with alterations observed in melanoma cells [76]. However, the specific interplay between these two genes in determining the plumage color of Liancheng ducks requires further investigation. Overall, we have provided the first annotation of the PMEL gene in the newly sequenced duck genome (Figure S4, Table S12), previously believed to lack this gene. MITF functions as a key gene that governs melanin production in ducks. Subsequent inactivation of the PMEL gene, situated in the feather bulb specimens resulting in the distinctive white feather and black feathers observed in Liancheng ducks.” (Line 532-556 in the revised manuscript).

3) Discuss the potential role of MITF in plumage coloration, referencing these studies to highlight its regulatory mechanisms and effects:

Goding CR, Arnheiter H. MITF-the first 25 years. Genes Dev. 2019 Aug 1;33(15-16):983-1007. doi: 10.1101/gad.324657.119. Epub 2019 May 23.

Yuan B, Qi Y, Zhang X, Hu J, Fan Y, Ji X. The relationship of MITF gene expression and promoter methylation with plumage colour in quail. Br Poult Sci. 2024 Jun;65(3):259-264. doi: 10.1080/00071668.2024.2326962.

Pan R, Hua T, Guo Q, Bai H, Jiang Y, Wang Z, Bi Y, Chen G, Wu X, Chang G. Identification of SNPs in MITF associated with beak color of duck. Front Genet. 2023 Aug 21;14:1161396. doi: 10.3389/fgene.2023.1161396.

Lin R, Lin W, Zhou S, Chen Q, Pan J, Miao Y, Zhang M, Huang Z, Xiao T. Integrated Analysis of mRNA Expression, CpG Island Methylation, and Polymorphisms in the MITF Gene in Ducks (Anas platyrhynchos). Biomed Res Int. 2019 Sep 23;2019:8512467. doi: 10.1155/2019/8512467.

Guo Q, Jiang Y, Wang Z, Bi Y, Chen G, Bai H, Chang G. Genome-Wide Analysis Identifies Candidate Genes Encoding Feather Color in Ducks. Genes (Basel). 2022 Jul 14;13(7):1249. doi: 10.3390/genes13071249.

Ren S, Lyu G, Irwin DM, Liu X, Feng C, Luo R, Zhang J, Sun Y, Shang S, Zhang S, Wang Z. Pooled Sequencing Analysis of Geese (Anser cygnoides) Reveals Genomic Variations Associated With Feather Color. Front Genet. 2021 Jun 18;12:650013. doi: 10.3389/fgene.2021.650013.

Lin R, Zhao F, Xiong T, Lai L, Li H, Lin W, Xiao T, Lin W. Genetic mapping identifies SNP mutations in MITF-M promoter associated with melanin formation in Putian black duck. Poult Sci. 2024 Jan;103(1):103191. doi: 10.1016/j.psj.2023.103191.

Sultana H, Seo D, Choi NR, Bhuiyan MSA, Lee SH, Heo KN, Lee JH. Identification of polymorphisms in MITF and DCT genes and their associations with plumage colors in Asian duck breeds. Asian-Australas J Anim Sci. 2018 Feb;31(2):180-188. doi: 10.5713/ajas.17.0298.

Zhang X, Zhu T, Wang L, Lv X, Yang W, Qu C, Li H, Wang H, Ning Z, Qu L. Genome-Wide Association Study Reveals the Genetic Basis of Duck Plumage Colors. Genes (Basel). 2023 Mar 31;14(4):856. doi: 10.3390/genes14040856.

Li S, Wang C, Yu W, Zhao S, Gong Y. Identification of genes related to white and black plumage formation by RNA-Seq from white and black feather bulbs in ducks. PLoS One. 2012;7(5):e36592. doi: 10.1371/journal.pone.0036592.

Wang L, Guo J, Xi Y, Ma S, Li Y, He H, Wang J, Han C, Bai L, Mustafa A, Liu H, Li L. Understanding the Genetic Domestication History of the Jianchang Duck by Genotyping and Sequencing of Genomic Genes Under Selection. G3 (Bethesda). 2020 May 4;10(5):1469-1476. doi: 10.1534/g3.119.400893.

Response: Thank you for your comments. We have supplemented the mechanism of MITF in the formation of feather color in the discussion section. "MITF regulates the expression of enzymes responsible for melanin synthesis and the expression of receptors involved in melanocyte function [52, 53, 54]. MITF produces various isoforms through alternative promoters with shared coding exons but distinct amino termini [55]. While MITF variants are known to influence melanin regulation, the regulation of these isoforms remained unclear. In ducks, we discovered the expression of two MITF isoforms, MITF-B and MITF-M, with only the latter being crucial for melanin synthesis in duck plumage [2,9, 56]. MITF-M isoforms also have been shown to regulate white coloration in the fur of dogs [57], llamas [58], and mice [59]. Previous research found SNPs, indels, and structural variants in MITF as possible causes of white plumage in ducks [60]. Two synonymous SNPs (c.114T>G and c.147T>C) and a 14-bp indel (GCTGCAAAC AGATG) in intron 7 of duck MITF were significantly associated with the black- and white-colored breeds ($p<0.001$) [61]. One variant in the 5'UTR of MITF were significantly associated with feather color phenotypes in geese [62]. A 6.6 kb insertion within the MITF gene demonstrated a strong correlation with melanin production in ducks [2] and indicated that MITF played an on-off role in the melanin generation pathway of Pekin ducks. MITF can promote differentiation-related functions, including regulation of genes involved in pigmentation, such as PMEL, TYR, TYRP1, DCT, MLANA, SILV, and SLC24A5 [60]. In the years following the separation of MITF gene, the number of potential target genes increased sharply. Based on GWAS analysis, it was confirmed that the MITF gene acts as an epistatic gene controlling melanin synthesis in Liancheng ducks, aligning with previous research findings [44]. This highlights the significant regulatory role of MITF in melanin synthesis in Liancheng ducks and underscores its importance as a key genetic factor in pigmentation.
" (Line 510-530 in the revised manuscript).

4) Compare your genome assembly results with these recently published assemblies to provide a comprehensive understanding of the advancements and differences:

Xu MM, Gu LH, Lv WY, Duan SC, Li LW, Du Y, Lu LZ, Zeng T, Hou ZC, Ma ZS, Chen W, Adeola AC, Han JL, Xu TS, Dong Y, Zhang YP, Peng MS. Chromosome-level genome assembly of the Muscovy duck provides insight into fatty liver susceptibility. Genomics. 2022 Nov;114(6):110518. doi: 10.1016/j.ygeno.2022.110518.

Ng CS, Lai CK, Ke HM, Lee HH, Chen CF, Tang PC, Cheng HC, Lu MJ, Li WH, Tsai IJ. Genome Assembly and Evolutionary Analysis of the Mandarin Duck Aix galericulata Reveal Strong Genome Conservation among Ducks. Genome Biol Evol. 2022 May 31;14(6):evac083. doi: 10.1093/gbe/evac083.

Che T, Li J, Li X, Wang Z, Zhang X, Yang W, Liu T, Wang Y, Wang K, Gao T, Shen G, Qiu W, Li Z, Zhang W. Haplotype-resolved assembly of the mule duck genome using

Powered by Editorial Manager® and ProduXion Manager® from Aries Systems Corporation

high-fidelity sequencing technology. PLoS One. 2024 Jul 1;19(7):e0305914. doi: 10.1371/journal.pone.0305914.

Jiang F, Jiang Y, Wang W, Xiao C, Lin R, Xie T, Sung WK, Li S, Jakovlić I, Chen J, Du X. A chromosome-level genome assembly of Cairina moschata and comparative genomic analyses. BMC Genomics. 2021 Jul 30;22(1):581. doi: 10.1186/s12864-021-07897-4.

Mueller RC, Ellström P, Howe K, Uliano-Silva M, Kuo RI, Miedzinska K, Warr A, Fedrigo O, Haase B, Mountcastle J, Chow W, Torrance J, Wood JMD, Järhult JD, Naguib MM, Olsen B, Jarvis ED, Smith J, Eöry L, Kraus RHS. A high-quality genome and comparison of short- versus long-read transcriptome of the palaearctic duck Aythya fuligula (tufted duck). Gigascience. 2021 Dec 20;10(12):giab081. doi: 10.1093/gigascience/giab081.

Hu J, Song L, Ning M, Niu X, Han M, Gao C, Feng X, Cai H, Li T, Li F, Li H, Gong D, Song W, Liu L, Pu J, Liu J, Smith J, Sun H, Huang Y. A new chromosome-scale duck genome shows a major histocompatibility complex with several expanded multigene families. BMC Biol. 2024 Feb 5;22(1):31. doi: 10.1186/s12915-024-01817-0.

Lavretsky P, Hernández F, Swale T, Mohl JE. Chromosomal-level reference genome of a wild North American mallard (Anas platyrhynchos). G3 (Bethesda). 2023 Sep 30;13(10):jkad171. doi: 10.1093/g3journal/jkad171.

Wang K, Hua G, Li J, Yang Y, Zhang C, Yang L, Hu X, Scheben A, Wu Y, Gong P, Zhang S, Fan Y, Zeng T, Lu L, Gong Y, Jiang R, Sun G, Tian Y, Kang X, Hu H, Li W. Duck pan-genome reveals two transposon insertions caused bodyweight enlarging and white plumage phenotype formation during evolution. Imeta. 2023 Dec 17;3(1):e154. doi: 10.1002/imt2.154.

Chang G, Yuan X, Guo Q, Bai H, Cao X, Liu M, Wang Z, Li B, Wang S, Jiang Y, Wang Z, Zhang Y, Xu Q, Song Q, Pan R, Qiu L, Gu T, Wu X, Bi Y, Cao Z, Zhang Y, Chen Y, Li H, Liu J, Dai W, Chen G. The First Crested Duck Genome Reveals Clues to Genetic Compensation and Crest Cushion Formation. Genomics Proteomics Bioinformatics. 2023 Jun;21(3):483-500. doi: 10.1016/j.gpb.2023.08.002.
Response: Thank you for your suggestions. We have added this content. By comparing the new genome assembly results with these recently published assemblies, we want to provide a comprehensive understanding of the advancements and differences. References are included in the discussion section of the revised manuscript. “The white plumage phenotype is a common trait observed in various avian species, such as chickens [38, 39] , peafowl [40] , geese [41] , also in ducks [2, 42]. Extensive research has focused on the unique white plumage phenotype of Liancheng ducks [42, 43, 44]; however, the genetic mechanisms behind its plumage color remain unclear. Our findings suggest that the inheritance of plumage color phenotype in Liancheng ducks is likely governed by two autosomal genes, independent of sex chromosomes (Table 1). Despite using the previous Pekin duck reference genome (IASCAAS_PekingDuck_PBH1.5, GCA_003850225.1), our GWAS analysis identified three signals (refer to Figure S8), which we believed may be due to incomplete assembly. To further investigate the white feather phenotype of Liancheng ducks, we first established a high-quality genome for Liancheng duck (refer to Figure 1). This new reference genome has a size of 1.29 Gb, with contig and scaffold N50 values of 12.17 and 83.98Mb, respectively (refer to Table S5). The scaffold N50 length of the Liancheng duck genome is higher than that of other duck genomes [45, 46, 47, 48], representing a more complete genome assembly that better reflects the continuity of the duck genome. However, the chromosomes number identified from this newly genome still needs improvement compared to those of the Muscovy duck and Crested Duck [49, 50]. Further pan-genome and functional gene-mining analysis can be conducted in the future [29, 47, 51]. Taken together, these findings firstly represent the first construction of the Liancheng duck genome, resulting in enhanced genome contiguity compared to previous duck genomes.” (Line 491-508 in the revised manuscript).

5) Replace "hair follicle" with "feather follicle" throughout the manuscript. Hair is a mammalian-specific skin appendage; birds have feathers, not hair.

Response: Thank you for your valuable suggestions. We have replaced "hair follicle" with "feather follicle" throughout the manuscript.

6) Specify which part of the feather follicle was used for RNA-seq extraction.
Response: We have elaborated the sample tissue for feather follicle RNA extraction in whole manuscript. Feather bulb specimens from feather follicle tissues of black-, grey- and white-feathered ducks were collected in this study.

7) Show the statistical test used along with the p-value in parentheses. For example, (ANOVA, $p < 0.05$).
Response: Thank you for your suggestions. We have showed the statistical test used along with the p-value in parentheses throughout the manuscript.

8) Figure 4B: Clarify what the error bars represent. Figure 4C: Explain the asterisk (*) and indicate which statistical test was applied. Figure 6: Specify what the error bars represent and the statistical test used.
Response: The error bars in the manuscript are mean ± standard error, while the ** and *** value symbols represent $p<0.01$ and $p<0.001$, respectively. We have reviewed the full manuscript and made revisions.

9) Clearly state the threshold after Bonferroni correction and justify choosing it over other methods such as FDR.

Response: Bonferroni correction is a conservative correction method. Based on a large amount of data, this method is used in this study to strictly control the error rate and minimize the false positive results. The FDR correction allows some control over the proportion of false positives, so we chose to use the Bonferroni correction. At the same time, many recent gene mining papers have used this method, and I have quoted the references that use this method (Zhou et al., 2018; Liu et al., 2023; Wang et al., 2023) (Line 184 in the revised manuscript).
References:
Zhou Z, Li M, Cheng H, et al. An intercross population study reveals genes associated with body size and plumage color in ducks. Nat Commun. 2018;9:2648.
Wang W, Zhang Y, Zhang X, et al. Heritability and recursive influence of host genetics on the rumen microbiota drive body weight variance in male Hu sheep lambs. Microbiome. 2023;11(1):197.
Liu D, Zhang H, Yang Y, et al. Metabolome-Based Genome-Wide Association Study of Duck Meat Leads to Novel Genetic and Biochemical Insights. Adv Sci. 2023;10:e2300148.

Reviewer 2#
Title: "A high-quality assembly reveals causal gene for unique plumage phenotype of Liancheng ducks"

- English is suboptimal, needs to be improved
- a major comment is that I think that you should place the Methods section before the Results section: your article is methods intensive (de novo genome assembly, resequencing, RNA-seq, GWAS etc.), therefore it is very difficult to fully understand and interpret your work without reading first the Materials and Methods. Please change the structure of your article.

Response: Thank you for your comments. We agree with you very much, and have put the Materials and Methods section behind the Background and the Analyses in front, which makes the manuscript more coherent. At the same time, we have invited professors in the field to revise and polish the paper, hoping that the manuscript can meet the requirements for publication in Gigascience. Thank you again for your suggestions.

Background

L40: phenotype instead of topic
Response: We have replaced “phenotype” with “topic” in the manuscript.

	L53: in which year was N50 = 76.3 Mb reached?<br>Response: We have added the year in which the duck genome was published in this part, and you also can find this part in the Supplement Table 6.<br><br>L54: why do you think you need more assembly quality in ducks? Please justify<br>Response: Thank you for your comments. We believe that a high-quality reference genome is particularly important for the genetic analysis of complex traits, especially the lack of genome information of Liancheng ducks. We believe that our study is valuable for the study of feather color in birds. “The initial draft of the duck assembly was first reported in 2013 [6]. Subsequent enhancements to the Pekin (PK) duck genomes have led to a scaffold N50 length of up to 76.3 Mb by 2020, which now is widely used for duck genome analysis [7]. Compared to the high quality and variety of genome assembly in other birds, especially chickens, further improvements in duck assembly quality are still needed. Previous research related on population genetics of ducks has primarily focused on meat quality [8], adipose deposition [9], and muscle weight [10], but genetic mechanisms of plumage color remain largely unexplored. Meanwhile, no reference genome of the famous LC duck has been published. Therefore, an upgraded LC duck genome is crucial to provide foundational data for future studies on this species. The accuracy of gene localization greatly depends on the quality of the genome assembly [9]”. (Line 62-71 in the revised manuscript).<br><br>L54: Previous research in which area? Animal breeding and genetics? Population genetics? Other?<br>Response: We have already supplemented this section. “Previous research related on animal breeding and genetics of ducks has primarily focused on meat quality [8], adipose deposition [9], and muscle weight [10], but genetic mechanisms of plumage color remain largely unexplored. Meanwhile, no reference genome of the famous LC duck has been published. Therefore, an upgraded LC duck genome is crucial to provide foundational data for future studies on this species.” (Line 66-70 in the revised manuscript).<br><br>L58: maybe it's improved quality of the duck genome?<br>Response: We have revised this sentence. Thanks!<br><br>L59: white duck down feathers? Maybe it's duck white down (?) feathers? What do you mean by "down feathers"?<br>Response: We have revised and improved this part. Thanks.<br><br>L61: what is HiFi sequencing? Are you referring to long-read sequencing? Be specific<br>Response: We have revised and improved this part. “To date, long-read sequencing technology (or third-generation sequencing) can produce reads longer than 10 kb, spanning highly repetitive genomic regions and bridging previously intractable assembly gaps to improve overall continuity. As an alternative to relying on short-read data polishing, PacBio introduced high-fidelity reads (HiFi reads) can provide more accurate, continuous and complete genetic information, making them a key enabling technology for research [13]” (Line 76-81 in the revised manuscript).<br><br>L69: by appearance you mean plumage? Please avoid generic terms<br>Response: We have revised this part. Thanks.<br><br>L72: ... famous duck breed characterized by its ...<br>Response: We have revised this sentence. Thanks.<br><br>L72: replace replace but with and: white feathers and black beak and feet (is feet the right word in birds? Please check)<br>Response: We have revised this word. The description of the duck appearance phenotype has been modified to make it more professional. “38 white-feathered ducks with yellow beaks and webbing (WY), 42 white-feathered ducks with black beaks and webbing (WB), 67 gray-feathered ducks with black beaks and webbing (GF), and 41 black-feathered ducks with black beaks and webbing (BF) underwent whole-genome resequencing (Table S1)” (Line 112-115 in the revised manuscript).<br>References:<br>Han D, Liu H, Ren L, Hu J, Yang Q. From the Analysis of Anatomy and Locomotor Function of Biological Foot Systems to the Design of Bionic Foot: An Example of the

Webbed Foot of the Mallard. Biomimetics. 2023;8(8):592.
Jaekel M, Wake DB. Developmental processes underlying the evolution of a derived foot morphology in salamanders. Proc Natl Acad Sci U S A. 2007;104(51):20437-20442.

L73: Liancheng is still a breed, not a distinct species
Response: We have revised this description.

L84: This study, right?
Response: We have revised this word.

L86: this research identified
Response: We have revised this description. Thanks.

Data description

L95-96: it is not clear whether you generated a de novo assembly of the duck genome in this study, or if you aligned your reads from resequencing to an existing build of the genome
Response: Thank you for your comments. To understand the genetic basis of unique plumage phenotype of Liancheng ducks, we utilized a high-quality de novo assembly of Liancheng duck genome (GCA_039998735.1). Additionally, we performed whole-genome resequencing of 366 ducks and aligned the resequencing data to the Liancheng duck genome assembly (GCA_039998735.1) generated in this study for GWAS analysis. Through transcriptome sequencing analysis across black-, and white-feathered ducks and nine duck tissues, we identified PMEL gene associated with plumage color in Liancheng ducks and elucidated the genetic mechanisms underlying the formation of duck plumage color.

L97: which are these multiple breeds?
Response: We have revised this description.

L97: one single gene? Or genes?
Response: We have revised this description. Through transcriptome sequencing analysis across black-, and white-feathered ducks and nine duck tissues, we identified PMEL gene associated with plumage color in Liancheng ducks and elucidated the genetic mechanisms underlying the formation of duck plumage color. Thanks!

Materials and Methods

L411: why these 5 timepoints? What was the rationale behind it?
Response: The 12d, 15d and 20d of duck embryo development were nodes with obvious changes in feather color of Liancheng ducks. Related studies on feather follicle development of ducks also focus on these stages (Chen et al., 2017). We believed that these three stages represent the three most obvious color phenotypes of Liancheng duck feathers during embryonic stage. The time of duck shell breaking is the 28th day of embryo development, and the feather color phenotype can be clearly identified after 1 week, so we selected these five nodes for phenotype collection and RNA-seq analysis.
References:
Chen X, Ge K, Wang M, Zhang C, Geng Z. Integrative analysis of the Pekin duck (Anas anas) MicroRNAome during feather follicle development. BMC Dev Biol. 2017;17(1):12.

L408-415: it is not clear how many samples you used for RNA-seq (and proteomics?): 227 ducks (Pekin + grey and black feathered ducks) times 5 timepoints times 3 replicates + heart, fat, muscle, brain, spleen, lung, liver, kidney, and skin tissues: please provide the total number of samples for transcriptomics, and the breakdown by timepoint/tissue
Response: Thank you for advice. We have described the sample size for RNA sequencing in detail and presented it in Supplement Table 11. A total of 37 samples were used for RNA-seq (Table S2) (Line 124-125 in the revised manuscript).

Powered by Editorial Manager® and ProduXion Manager® from Aries Systems Corporation

L428: It is not clear what you did with BUSCO: this is a tool usually employed to assess the quality of genome assemblies. Please add details of what you did, why and how you did it
Response: Thank you for comments. The quality of the genome assembly is assessed based on the completeness metrics from BUSCO. Generally, a higher proportion of complete BUSCOs indicates better genome quality. A thorough evaluation of the assembly quality post-assembly, using BUSCO, revealed 96.7% genome completeness (Table S7). Run BUSCO using the command line with the following basic command format: busco -i <input_genome.fasta> -o <output_name> -l <lineage> --mode genome.

L430: "The structure prediction of gene": do you mean the prediction of the structure of genes?
Response: Thank you for comments. We have rephased the sentence to be clear for the Prediction of protein-coding genes (Line 141-142 in the revised manuscript).

L431: delete "as evidence files"
Response: We have deleted this part. Thanks.

L434: please explain (also in the text) what Augustus is
Response: We have revised and improved this part. Augustus is a program that predicts genes in eukaryotic genomic sequences. In this study, "Augustus (http://bioinf.uni-greifswald.de/augustus/) that predicts genes in genomic sequences, and then de novo annotation was performed through Augustus." (Line 145-147 in the revised manuscript).

L435: also here, explain what Maker and De novo are and/or refer to
Response: We used RNA-seq data for evidence-based annotation through Maker (Version 2.31.10), which is a powerful open source genome annotation tool (Line 149-150 in the revised manuscript). De novo genome assembly refers to a method of piecing together a complete sequence of a new genome, rather than comparing it based on a known genome." (Line 147-149 in the revised manuscript). We have already supplemented and explained these parts in the text. Thanks!

L436: repeated sequwnces
Response: We have revised this word.

L437: encoded protein longer than
Response: We have revised this part.

L437: at least one count from gene expression / RNAseq
Response: We have revised the sentence. Thanks.

L441: please add references to eggNOG and BLAST
Response: We have added references to this part. Thanks.

L447: average read length
Response: We have revised these words. At the same time, we have polished the language and modification of the full text, hoping to meet the requirements of the magazine publication.

L448: what is the original coverage? You mean the target coverage "a priori"?
Response: We have revised and improved this part. All libraries were sequenced on an Illumina HiSeq X-Ten platform, achieving an average raw read sequence coverage of 5×. Based on Liancheng duck reference genome (IASCAAS_LianchengWhiteDuck, GCA_039998735.1), this ensures the accuracy of variant calling and genotyping [6].

L448: it is usually known as "variant calling"
Response: We have revised these words. Thanks.

L449: which requirements?
Response: We have revised these parts. A detailed description of this section can be found in the following section (Line 175-193 in the revised manuscript).

Powered by Editorial Manager® and ProduXion Manager® from Aries Systems Corporation

	L450: you threw away the entire reads containing the adapter sequence? Or did you remove just the adapter using tools like Cutadapt? How did you identify and eliminate these reads? More details are needed<br>Response: We have revised this part. “Based on Liancheng duck reference genome (IASCAAS_LianchengWhiteDuck, GCA_039998735.1), this ensures the accuracy of variant calling and genotyping [6]. Following the elimination of read pairs containing adapter sequences, a quality assessment of the raw reads was performed utilizing TRIMMOMATIC (RRID:SCR_011848) (version 0.36) and Cutadapt [24,25]. Subsequently, the high-quality reads were aligned to the Liancheng duck reference genome (IASCAAS_LianchengWhiteDuck, GCA_039998735.1) using the Burrow–Wheeler Aligner (BWA-aln) with parameter ‘bwa aln genome.fa sample.fastq > sample.sai, bwa sampe genome.fa sample.sai sample.fastq > sample.sam’ [26].<br><br>L453: please report the default settings, this will aid the reader<br>Response: Thank you for your advice. The answer is shown in the last answer.<br><br>L453-454: did you perform variant calling with BWA? How?<br>Response: Thank you for comments. The Genome Analysis Toolkit (GATK) (McKenna et al., 2010) was used to identify genetic variants from the sequencing data in this study.<br><br>L455: I don't understand the filter on max allele frequency: SNPs are biallelic, if the frequency of one allele is 0.99 then MAF = 0.01, so this criterion is useless and redundant<br>Response: Thank you for your revisions. We have supplemented and revised the description of this part. “SNPs underwent filtration based on the following criteria: (i) SNPs were required to demonstrate a minor allele frequency (MAF) >0.05; (ii) the maximum missing rate was set at <0.7; and (iii) SNPs were restricted to possessing only two alleles” (Line 169-172 in the revised manuscript).<br><br>L456: max missing rate per SNP, ok: what about sample missing rate?<br>Response: Thank you for your valuable comments on this paper, so that the expression and logic of the manuscript are more rigorous. In this study, we completed SNP filtering and missing rate analysis, but did not carry out sample missing rate analysis. We have the following reasons: Firstly, we found the PMEL gene controlling the white feather phenotype of Liancheng duck through GWAS analysis after SNP filtering, and found two completely linked SNP variations, indicating that the analysis method is effective in this study. Secondly, we found that some papers did not carry out sample missing rate analysis in GWAS genome-wide association (Zhu et al., 2016; Zhou et al., 2018). Finally, we believe that sample missing rate detection is very meaningful, but the sample size in this study is small. In the future, we hope to increase the sample size and conduct sample missing rate detection, hoping to further verify our experimental results through a large number of samples.<br>References:<br>Zhu, Z., Zhang, F., Hu, H. et al. Integration of summary data from GWAS and eQTL studies predicts complex trait gene targets. Nat Genet 48, 481–487 (2016).<br>Zhou, Z., Li, M., Cheng, H. et al. An intercross population study reveals genes associated with body size and plumage color in ducks. Nat Commun 9, 2648 (2018).
**Additional Information:**	
**Question**	**Response**
Are you submitting this manuscript to a special series or article collection?	No
**Experimental design and statistics**<br><br>Full details of the experimental design and statistical methods used should be given in the Methods section, as detailed in our Minimum Standards Reporting Checklist.	Yes

Information essential to interpreting the data presented should be made available in the figure legends.<br><br>Have you included all the information requested in your manuscript?	
**Resources**<br><br>A description of all resources used, including antibodies, cell lines, animals and software tools, with enough information to allow them to be uniquely identified, should be included in the Methods section. Authors are strongly encouraged to cite Research Resource Identifiers (RRIDs) for antibodies, model organisms and tools, where possible.<br><br>Have you included the information requested as detailed in our Minimum Standards Reporting Checklist?	Yes
**Availability of data and materials**<br><br>All datasets and code on which the conclusions of the paper rely must be either included in your submission or deposited in publicly available repositories (where available and ethically appropriate), referencing such data using a unique identifier in the references and in the "Availability of Data and Materials" section of your manuscript.<br><br>Have you have met the above requirement as detailed in our Minimum Standards Reporting Checklist?	Yes

Powered by Editorial Manager® and ProduXion Manager® from Aries Systems Corporation

Manuscript Click here to access/download;Manuscript;20241005RevisedManuscript.do

A high-quality assembly reveals *PMEL* gene for unique plumage phenotype of Liancheng ducks

Zhen Wang[1,2], Zhanbao Guo[1], Hongfei Liu[1], Tong Liu[1], Dapeng Liu[1], Simeng Yu[1], Hehe Tang[1], He Zhang[1], Qiming Mou[1], Bo Zhang[1], Junting Cao[1], Martine Schroyen[2], Shuisheng Hou[1], Zhengkui Zhou[1]*

[1] State Key Laboratory of Animal Biotech Breeding, Institute of Animal Science, Chinese Academy of Agricultural Sciences, Beijing, China.

[2] Precision Livestock and Nutrition Unit, Gembloux Agro-Bio Tech, TERRA Teaching and Research Centre, University of Liège, Passage des Déportés 2, Gembloux 5030, Belgium.

Correspondence:

Zhengkui Zhou, zhouzhengkui@caas.cn

Institute of Animal Sciences, Chinese Academy of Agricultural Sciences, No. 2 Yuanmingyuan West Rd., Beijing 100193, China.

Abstract

Background: Plumage color is a notable characteristic and has widely studied. Liancheng duck white plumage and black markings on the beak and webbing, provide an ideal model for studying this topic. Our understanding of the genetic basis of duck plumage coloration remains limited. Therefore, we *de novo* assembled Liancheng duck genome (GCA_039998735.1) using HIFI reads and generated F2 segregating populations from Liancheng and Pekin ducks, aiming to identify the genetic mechanism of white plumage in Liancheng ducks.

Results: In this study, we *de novo* assembled 1.29 G Liancheng duck genome, with contig N50 of 12.17 Mb and scaffold N50 of 83.98 Mb. Beside the epistatic effect gene *MITF*, the GWAS analysis identified a 0.8Mb region, which includes the *PMEL* gene that encodes a pigment cell-specific protein that plays a pivotal role in the formation of fibrillar sheets within the melanosome, the pigment organelle. Additionally, based on Linkage Disequilibrium (LD) analysis, we identified two candidate SNPs (Chr33:5,303,994A>G; 5,303,997A>G) that may alter *PMEL* transcription activity, resulting in a change of plumage color in Liancheng duck.

Conclusions: Our study constructed a high-quality Liancheng duck genome, and presented strong evidence that white plumage in Liancheng duck is caused by the *PMEL* gene, providing valuable insights and guidance for future research and breeding effort on avian plumage coloration.

Keywords: duck, genome assembly, plumage color, *PMEL*, melanin

Background

Plumage color is a visually striking trait found in a diverse range of bird species, making it a valuable phenotype for investigating natural and artificial selection. Melanin, the primary pigment influencing avian plumage color, typically exists in bird tissues as a mixture of eumelanin and phaeomelanin, resulting in a wide variety of colors [1,2]. Eumelanin deposition in plumage leads to black or brown hues, serving as the predominant pigment in bird feathers [3]. Extensive research on eumelanin and melanin-related genes has significantly enhanced our understanding of avian plumage coloration, which is a captivating ornamental feature. Duck (*Anas platyrhynchos*) (NCBI:txid8839), as a typical bird, have successfully spread worldwide, exhibiting plumage colors

ranging from white to black, potentially adapting to different ecological environments. Due to the diverse plumage color patterns, ducks are a key animal model for studying pigmentation. Despite advancements in understanding the biological and evolutionary aspects of plumage color, the genetic basis of these colors in ducks remains poorly understood.

The Liancheng (LC) duck is a well-known breed characterized by its unique combination of white feathers, and black beak, and webbing. It is recognized for its significant melanin deposition in the beak and webbing, primarily due to the involvement of eumelanin as the main pigment [4]. The biosynthesis of eumelanin involves three key steps: tyrosinase converts tyrosine to dihydroxyphenylalanine (DOPA) through oxidation, then oxidase transforms DOPA into dopaquinone, and finally, dopaquinone undergoes cyclic transformations to produce pigment, leading to melanin formation [5]. This synthetic pathway plays a vital role in eumelanin synthesis, particularly in the pigmentation of skin and feather in ducks. In this process, *MITF* is highlighted as a key target of various signal transduction pathways and serves as the main regulator of melanin production. However, the genetic basis of melanin deposition and the specific genes involved in the formation of white feathers in LC ducks remain unknown.

The initial draft of the duck assembly was first reported in 2013 [6]. Subsequent enhancements to the Pekin (PK) duck genomes have led to a scaffold N50 length of up to 76.3 Mb by 2020, which now is widely used for duck genome analysis [7]. Compared to the high quality and variety of genome assembly in other birds, especially chickens, further improvements in duck assembly quality are still needed. Previous research related on animal breeding and genetics of ducks has

primarily focused on meat quality [8], adipose deposition [9], and muscle weight [10], but genetic mechanisms of plumage color remain largely unexplored. Meanwhile, no reference genome of the famous LC duck has been published. Therefore, an upgraded LC duck genome is crucial to provide foundational data for future studies on this species. The accuracy of gene localization greatly depends on the quality of the genome assembly [9]. Based on widely used duck genome (GCA_003850225.1), a study identified a 6.6kb intronic insertion in *MITF* that likely affects splicing, leading to white feathers in ducks [11]. Additionally, four new single nucleotide polymorphisms in the *MC1R* regulator region associated with black plumage were discovered in ducks [12]. This partially explains the mechanism of melanin formation in duck feathers, but many questions about feather color remain unanswered. To date, long-read sequencing technology (or third-generation sequencing) can produce reads longer than 10 kb, spanning highly repetitive genomic regions and bridging previously intractable assembly gaps to improve overall continuity. As an alternative to relying on short-read data polishing, PacBio introduced high-fidelity reads (HiFi reads) can provide more accurate, continuous and complete genetic information, making them a key enabling technology for research [13]. Meanwhile, advancements in gene chips and genome re-sequencing technologies, have made genome-wide association studies (GWAS) powerful tools for identifying genetic variations linked to phenotypes. GWAS analysis has uncovered mutations in the *MuPKS* gene responsible for yellow and blue plumage in parrots [14] and pinpointed the *SLC2A11B* gene with a nonsense mutation (W49X) causing the white eye trait in pigeons [15]. Therefore, based on a high-quality *de novo* genome by HiFi sequencing, this study reveals the presence of the *PMEL* gene, which was previously thought to be “missing” gene in ducks. Furthermore, this research identified two closely linked single nucleotide polymorphisms (SNPs) in the regulatory region that

may influence *PMEL* transcription, leading to the white plumage observed in LC ducks. Overall, this investigation provided a valuable genome assembly, molecular markers for duck breeding, and insights into plumage color patterns in avian species.

Data Description

To understand the genetic basis of unique plumage phenotype of Liancheng ducks, we utilized a high-quality *de novo* assembly of Liancheng duck genome (GCA_039998735.1), and collected four different plumage color phenotypes from a crossbreeding population involving Liancheng and PK ducks. Additionally, we performed whole-genome resequencing of 366 ducks and aligned the resequencing data to the Liancheng duck genome assembly (GCA_039998735.1) generated in this study for GWAS analysis. Through transcriptome sequencing analysis across black-, and white-feathered ducks and nine duck tissues, we identified *PMEL* gene associated with plumage color in Liancheng ducks and elucidated the genetic mechanisms underlying the formation of duck plumage color.

Materials and Methods

Ducks and sampling

All animal procedures in this study were carried out in accordance with the guidelines for the care and use of experimental animals set by the Chinese Academy of Agricultural Sciences (IAS2022-105). Approval for this study was granted by the ethics committee of the Chinese Academy of Agricultural Sciences. A blood sample from a female Liancheng duck was used for *de novo* genome assembly. A total of 366 parents and intercross population duck plumage color phenotypes were

recorded from a previous gradient consanguinity population [6], which included 117 PK ducks, 59 LC ducks, 38 white-feathered ducks with yellow beaks and webbing (WY), 42 white-feathered ducks with black beaks and webbing (WB), 67 gray-feathered ducks with black beaks and webbing (GF), and 41 black-feathered ducks with black beaks and webbing (BF) underwent whole-genome resequencing (Table S1). Additionally, genome data from 23 black-feathered ducks, comprising 20 Mallards (MD) and 3 Putian (PT) ducks, were used for comparative analysis [2].

For transcriptomics analysis, skin tissues from LC ducks were collected at embryonic stages of 12 days, 15 days, 20 days, 28 days, and one week after birth, with each sample group consisting of three biological replicates, except for the four 12-day embryo samples. Additionally, heart, fat, muscle, brain, spleen, lung, liver, kidney, and skin tissues from an 8-week-old black-feathered MD duck were collected, and one replicate per tissue (Table S2) [2]. Feather bulb specimens from feather follicle from 1-week-old black-feathered MD ducks, LC ducks, PK ducks, and WB ducks were also collected, with each sample group consisting of three biological replicates [16,17]. A total of 37 samples were used for RNA-seq (Table S2). For immunofluorescence assay, skins containing hair follicles from three 1-week-old MD ducks and three LC ducks were collected for protein analysis. These tissues were then used for transcriptome and proteome analyses following animal welfare ethics standards.

Genome assembly and Gene annotation

To conduct the *de novo* assembly of the Liancheng duck genome, we utilized a combination of PacBio long-read HiFi sequencing and chromosome interaction mapping (Hi-C) technologies. The

project used long-read and long-HiFi (RRID:SCR_021966) sequencing data (PacBio, Beijing, China) to assemble the species [18]. Using Pacbio Sequel II platform sequencing, the HiFi-asm (https://github.com/chhylp123/hifiasm) versions (v0.19.3 NOV-2023), assembling, genome Contig. Through Juicer (NOV-2023), Hi-C data of quality control was compared back to the assembled Contig sequence [19,20]. The 3D-DNA software (RRID:SCR_017227) (NOV-2023) was used to divide, sequence and Orient the genome sequences [21]. The results of BUSCO assembly were evaluated to assess the completeness and quality of genome assembly [22].

Prediction of protein-coding genes was performed by combining evidence-based prediction and *de novo* prediction. We used RNA-seq data for evidence-based annotation through Maker (Version 2.31.10), which is a powerful open source genome annotation tool. With the comparison information between RNA-seq data and genome, PASA (Program to Assemble Spliced Alignments) (RRID:SCR_014656) was used to construct the training model of *Augustus* (http://bioinf.uni-greifswald.de/augustus/) that predicts genes in eukaryotic genomic sequences, and then *de novo* annotation was performed through *Augustus*. *De novo* genome assembly refers to a method of piecing together a complete sequence of a new genome, rather than comparing it based on a known genome. Then we integrated the results of Maker and *de novo* based on the principle that evidence results were better than prediction results. Finally, genes with repeated sequences less than 50%, encoded protein longer than 50 amino acids, and at least one count from gene expression sample were retained. Functional annotation of genes was performed by eggNOG software (RRID:SCR_002456) [23] against databases of GO, KEGG, NR, SWISS-PROT.

Whole-genome resequencing

A total of 366 samples, consisting of Pekin ducks, Liancheng ducks, and intercross populations, were selected for resequencing (Table S1). The genome data of 23 black-feathered ducks includes 20 Mallards, and 3 Putian ducks. DNA-eligible samples were identified for further testing. Libraries were established for the samples, with an average read length of 150 bp. All libraries were sequenced on an Illumina HiSeq X-Ten platform, achieving an average raw read sequence coverage of 5×. Based on Liancheng duck reference genome (IASCAAS_LianchengWhiteDuck, GCA_039998735.1), this ensures the accuracy of variant calling and genotyping [6]. Following the elimination of read pairs containing adapter sequences, a quality assessment of the raw reads was performed utilizing TRIMMOMATIC (RRID:SCR_011848) (version 0.36) and Cutadapt [24,25]. Subsequently, the high-quality reads were aligned to the Liancheng duck reference genome (IASCAAS_LianchengWhiteDuck, GCA_039998735.1) using the Burrow–Wheeler Aligner (BWA-aln) with parameter 'bwa aln genome.fa sample.fastq > sample.sai, bwa sampe genome.fa sample.sai sample.fastq > sample.sam' [26]. The Genome Analysis Toolkit (GATK) [27] was used to identify genetic variants from the sequencing data in this study. SNPs underwent filtration based on the following criteria: (i) SNPs were required to demonstrate a minor allele frequency (MAF) >0.05; (ii) the maximum missing rate was set at <0.7; and (iii) SNPs were restricted to possessing only two alleles.

Genome-wide association analysis

The GWAS was conducted utilizing a mixed linear model implemented through the EMMAX program (RRID:SCR_024012) [28] with genome-wide single nucleotide polymorphism data and

the plumage color phenotype observed in 366 individuals from the resequencing population. The analytical model adopted the form y = Xb + Ga + e, where y represented the phenotypic value (plumage color of each duck), X denoted the matrix corresponding to fixed effects, and b signified the magnitude of the fixed effects. The fixed effects encompassed sex-related influences. G represented the genetic matrix associated with population kinship, while e stood for the random residual. Principal component analysis (PCA) was executed using all SNPs, with the top three components incorporated as fixed effects within the mixed model to adjust for population stratification. A Bonferroni correction threshold of 0.01/N ($-\log_{10}P = 8.95$) was established to pinpoint significant sites in the GWAS findings [2,8,29], where N indicated the total number of whole-genome SNPs (8,887,194). IBD analysis was used to conduct the fine-mapped analyses in 328 ducks. IBD fragments can reflect the genetic relationship between individuals and detect trait variation, and the correlation between IBD fragments and phenotype was used to identify regions affecting trait variation in the genome [28,30]. For this analysis, the filtered SNPs (n= 117) met the standard allele frequency difference (ΔAF) >0.8 between the Liancheng ducks and Pekin ducks. In the candidate region (Chr33:5.1-5.5Mb), we identified four recombination breakpoints across the 36 SNPs and subsequently classified the segregating individuals using these four recombinant breakpoints.

Transcriptome sequencing and analysis

Twelve feather bulb specimens were collected from feather follicle tissues of Mallards, WB ducks, Liancheng ducks, and Pekin ducks at one week after birth, as well as heart, fat, muscle, brain, spleen, lung, liver, kidney, and skin tissues from an 8-week-old black-feathered mallard. Total RNA was

first extracted from the above tissues using Vazyme's Trizol reagent. The RNase enzyme was inactivated by the addition of pyrrole diethyl carbonate. A total of 12 final RNA-seq libraries were prepared for the experiment and sequenced on an Illumina platform using the 150-bp paired-end sequencing module. The effective read length was increased by illumina sequencing, with an average production of 6 Gb per library. Using TopHat (RRID:SCR_013035), RNA-seq paired-end reads from each library were mapped to the newly reference genome of Liancheng duck. Expression was calculated by using TopHat, and read counts per million (CPM) values for the genes were obtained by running htseq-count [6,31].

qPCR for transcript of *PMEL* in feather bulb specimens

cDNA from feather bulb specimens, including those of black-feathered, grey-feathered, Liancheng, and Pekin ducks, was reversely transcribed using HiScript III All-in-one RT SuperMix Perfect for qPCR (Vazyme). The reverse transcription quantitative PCR (qPCR) was conducted in a total volume of 10 μl, which included 5 μl Tap Pro Universal SYBR qPCR Master Mix (Vazyme), 0.8 μl forward and reverse primers, 0.5μl cDNA, 3.7 μl of distilled water. β-actin was selected as the internal reference gene. The primer sequence was shown in Table S3. All reactions were run in triplicate. The relative mRNA expression levels were calculated using the normalized relative quantification method, followed by the $2^{-\Delta\Delta CT}$ calculation [32].

Immunofluorescence experiment

The skin samples of ducks with black and white feathers were embedded in paraffin, fixed in 4% buffered paraformaldehyde, and sectioned into 5 μm slices. After overnight fixation at 4°C, it was

ensured that the duration of fixation did not exceed 24 hours to effectively maintain tissue integrity. Subsequently, the sections were dewaxed and rehydrated to enhance adhesion and facilitate the dewaxing process. Antigen retrieval was performed by incubating the sections in EDTA (Servicebio) at 100°C for 20 minutes. Following retrieval, the antigens were fixed in Tris-EDTA, and the sections were washed three times with phosphate-buffered saline (PBS), followed by an additional wash. For immunostaining, the sections were incubated with the PMEL antibody (Abclonal) at 4°C for 12 hours after pre-treatment with 3% bovine serum albumin (Solarbio) for 30 minutes. The PMEL antibody used was an anti-rabbit antibody. Finally, the feather follicle tissues exhibiting various plumage colors were counterstained with DAPI. Experimental outcomes were documented through photographic and record-keeping procedures.

Causative mutation screening and identification

Based on the Liancheng duck genome, we compared the candidate regions (Chr33:5.24-5.32Mb) among 117 Pekin ducks, 59 Liancheng ducks, and 152 intercross population ducks. Among the candidate IBD fragments, only the regions where genotypes and phenotypes were consistent were further investigated as *Rr* candidate regions. To eliminate variations with a lower likelihood of being causally involved, we implemented the following three steps. Firstly, we utilized the genotype and phenotype information from the 328 parents and intercross ducks to exclude SNPs based on the standard Fst < 0.8 (LC vs PK duck). Secondly, we utilized the genotypes and phenotypic information of 117 Pekin ducks, 59 Liancheng ducks, 20 Mallards, 42 WB ducks, 43 BF ducks, and 3 Putian ducks. The high Fst values shared by Liancheng ducks and the other breeds were chosen as further candidate regions. Thirdly, only genotypes totally consistent with the phenotypes from the Mallard

and Putian duck populations were considered as candidate causative mutations. Finally, all indel within the candidate region were excluded using the aforementioned method. Only 2 SNP variations whose genotype and phenotype were consistent across multiple duck breeds could be regarded as causative variations for the *Rr* locus.

Hi-C sequencing and analysis

Skin fat tissue samples from a Liancheng duck were subjected to cross-linking in 20 ml of fresh ice-cold nuclear isolation buffer. The chromatin extraction methodology followed previous protocols [2]. Subsequently, the purified DNA underwent digestion and fragmentation using the *DpnII* restriction enzyme, followed by repair of DNA ends. Biotin-labeled DNA fragments were then isolated using streptavidin C1 beads. Library preparation was conducted utilizing an Illumina TruSeq DNA Sample Prep Kit following the manufacturer's guidelines. Quality assessment of the Hi-C library was performed through TA cloning. Sequencing of the Hi-C libraries was conducted on an Illumina HiSeq X Ten system. The Hi-C experiments were independently performed twice, with the experimental and sequencing procedures executed by Gene Technology Co., Ltd., located in Beijing, China.

Raw Hi-C data underwent processing to eliminate low-quality reads and trim adapters using TRIMMOMATIC (RRID:SCR_011848) [24]. All reads were trimmed to 50 bp, and clean reads were aligned to the duck genome using a two-step approach integrated into the HiC-Pro (RRID:SCR_017643) software [33]. Reads of low mapping quality, multiple mappings, and singletons were excluded. Subsequently, uniquely mapped reads were consolidated into a single file.

Read pairs that did not align near a restriction site or failed to meet the anticipated fragment size after shearing were filtered out. Further filtering steps were implemented to discard read pairs originating from invalid ligation products, such as dangling-end and self-ligation products, as well as PCR artifacts. The remaining valid read pairs were categorized into intrachromosomal and interchromosomal pairs. Contact maps were generated using chromosome bins of uniform sizes ranging from 3 kb to 1 Mb. The initial contact maps were normalized using a sparse-based iteration correction method within HiC-Pro and visualized using HiCPlotter [34]. Finally, regions resembling topologically associated domains (TADs) and boundaries were delineated using the default algorithm within HiCPlotter at a resolution of 5 kb [34].

Structural variation detection

In the GWAS candidate region, we analyzed all CNV structural variations in selected populations, including Pekin ducks, Liancheng ducks, WB ducks, GF ducks, and BF ducks. Ducks from these different feather groups were randomly chosen. We utilized CNVcaller (RRID:SCR_015752) software (version 0.11) to investigate the genotype of all individual CNVs [35]. The CNV calling and genotyping procedures were consistent with those described in previous studies [36]. Log_2 fold change values reflected the ratio of sequencing read depths in the 1,000bp window region to that of Pekin duck reads. Therefore, we examined the distribution of all CNV genotypes in the aforementioned populations. A copy number of 1 indicated a normal diploid state, 0.5 denoted loss of heterozygosity, 0 signified homozygous loss, 1.5 indicated heterozygous duplication, and 2 represented homozygous duplication. An absolute copy number exceeding 2 indicated complex duplications [36].

Luciferase reporter assay

Four haplotypes of candidate variations SNP1 and SNP2, along with their upstream and downstream regions, were cloned into the pGL3-basic and pGL3-promoter vector. In this study, the *XhoI* and *KpnI* sites were used as insertion sites in pGL3-basic for analyzing promoter activity, while *BamHI* and *SalI* sites were selected in pGL3-promoter vector for analyzing enhancer activity. A375 and DEF cells were plated in 48-well plates at a density of $0.5x10^5$ per well and cultured for 24h in DMEM (Pricella, China) mixed with 10% FBS (Pricella, China). A375 and DEF cells were transfected with Lipofectamine 8000 (Beyotime), ensuring that each well contained the same 237.5ng of DNA, which included the four sequences containing SNP1 and SNP2 sites. At the same time, 12.5ng of pRL-TK vector was added to each well. According to the instructions, after lysis of cells, the cell lysate was collected and luciferase activity was measured using the Veritas Microplate Luminometer (Promega). Each sample was repeated three times, and Renilla fluorescence was used to normalize firefly fluorescence [12, 37].

Analyses

A newly assembled high-quality Liancheng duck genome

In order to better analyze the white plumage Liancheng duck, we constructed a *de novo* genome, HiFi long-read sequence data with 88× genome coverage and 584.72 Gb of Hi-C data were generated (Table S4). These datasets were then utilized for assembling the new duck genome (IASCAAS_LianchengWhiteDuck, GCA_039998735.1) (Figure 1A), resulting in a final assembled genome length of 1.29 Gb (Table S5). Chromosome assembly was conducted using hifisam

(RRID:SCR_021069). The Scaffold N50 of the *de novo* genome assembly reached 83.98 Mb, while the Contig N50 was 12.17 Mb (Table S5). Collinearity analysis with Mallards (GCA_008746955.3) and the Pekin duck reference genome (GCA_015476345.1) demonstrated the high quality of the Liancheng duck genome assembly (Figure 1B). The Scaffold N50 length of Liancheng duck in this study was the longest among all previously published duck (*Anas platyrhynchos*) genomes (Figure 1C, 1D, Table S6). A thorough evaluation of the assembly quality post-assembly, using BUSCO (RRID:SCR_015008) [8], revealed 96.7% genome completeness (Table S7). After annotating the newly assembled reference genome, 33 chromosomes were identified (Figure 1C, Table S8) and a total of 18,819 genes were annotated (Table S8), marking the first genome annotation for the Liancheng duck.

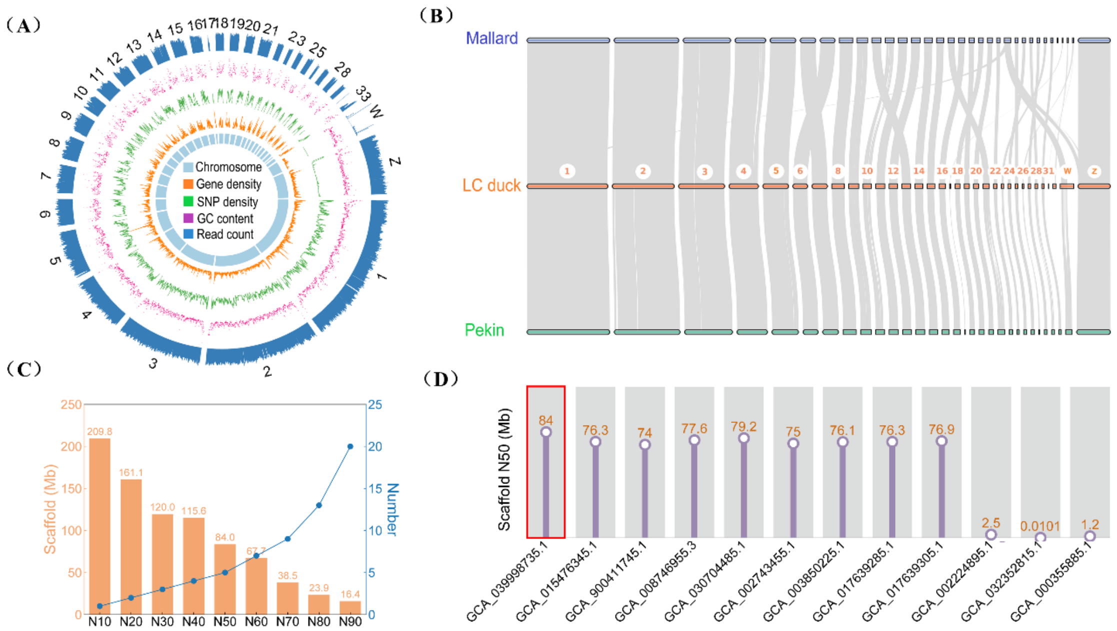

Figure 1. Overview of the assembly quality and characteristics of the Liancheng duck genome. (A) Circular diagram illustrating the characteristics of the genome assembly. The tracks from the inner to outer circles represent the following: chromosomes, gene density, SNP density, GC content (%), and read count. The window size of each circle was 200 kb. (B) Genome synteny analysis between

the Liancheng duck and Mallard, Pekin duck. Chromosomes 1-33, as well as two sex chromosomes. (C) Genome statistics for the HiFiasm genome assemblies of the Liancheng duck genome in this study. (D) The length of Scaffold N50 (Mb) of Liancheng duck in this study (GCA_039998735.1) was compared with all previously published duck (*Anas platyrhynchos*) genomes.

The inheritance of F2 population traits conforms to the law of independent assortment

A crossbreeding study was conducted involving 30 Pekin and 120 Liancheng ducks. All F1 individuals (1,260/1,260) displayed a grey plumage color and pattern. In the F2 populations, four phenotypes were observed: BF, GF, WB, and WY ducks (Figure 2). The ratio of BF: GF: WB: WY ducks in the F2 population was 235:452:234:360, closely matched the theoretical ratio of 3:6:3:4 (Table 1 and Figure S1). The phenotypic ratio discovered adheres to Mendel's law of independent assortment of two genes. It is hypothesized that the genetic mechanism controlling plumage color in Liancheng ducks is governed by two sites (*Bb* and *Rr* sites), where the allele at the *Rr* site, in interaction with the *Bb* site, determines white plumage in Liancheng ducks (Table 1). Within the F2 population, two alleles (*B*-dominant, enabling melanin synthesis, and *b*, which inhibits melanin synthesis) segregated at the *Bb* locus. The other locus, denoted as *Rr*, possesses two alleles that regulate melanin accumulation in the feather: *R* (dominant, allowing melanin synthesis in the feather) and *r* (repressing melanin synthesis). The *B* allele at the *Bb* locus displayed an epistatic effect, while the *R* allele at the *Rr* locus demonstrated incomplete dominance effect. The cross between the Liancheng duck (*BBrr*) and Pekin duck (*bbRR*) resulted in the production of grey feather ducks (*BbRr*), with the genotypes of BF, GF, WB, and WY being *B_RR*, *B_Rr*, *B_rr*, and *bb_ _*, respectively (refer to Table 1, Figure 2). Importantly, there was no significant difference observed

between the actual and expected numbers within the F2 population (n=1,281, p=0.345), with a squared value of 3.322 ($\chi 2_{0.05(3)}$=7.81, $\chi 2_{0.01(3)}$=11.34). The *Bb* locus has previously been identified as the primary gene responsible for white plumage in ducks [2].

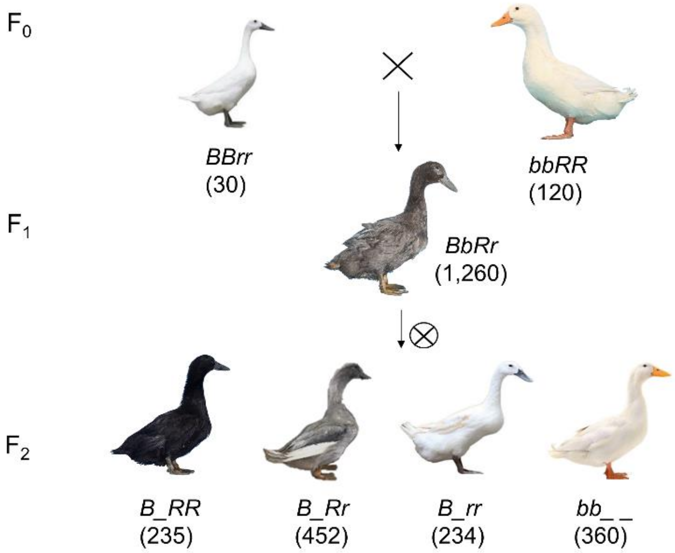

Figure 2. The diagram depicted the segregation of plumage colors in the F2 population. The Liancheng duck showed the white feather black beak and webbing (WB) phenotype, whereas the Pekin duck exhibited the white feather yellow beak and webbing (WY) phenotype. The F1 generation displayed the gray feather black beak and webbing phenotype (GF) with black spots on the webbing. In the subsequent F2 generations, ducks were observed with phenotypes including black feather black beak and webbing (BF), GF, WB, and WY ducks.

Table 1 The number of F2 populations in different phenotypes and the Chi-squared test.

Comparison	BF duck (*B_RR*)	GF duck (*B_Rr*)	WB duck (*B_rr*)	WY duck (*bb_ _*)	Ratios	χ^2 value	*P*-value
Observed number	235	452	234	360	3:5.8:3:4.6	3.322	0.345 (ns)

Expected number	240	481	240	320	3:6:3:4

Notes: BF represents ducks with black-feathered, beaks, and webbing in the F2 population; GF represents ducks with grey-feathered, black beaks, and webbing in the F2 population; WB represents ducks with white-feathered, black beaks, and webbing in the F2 population; WY represents ducks with white-feathered, yellow beaks, and webbing in the F2 population. $\chi^2_{0.05(3)}=7.81$, $\chi^2_{0.01(3)}=11.34$; ns, not significant difference.

Genome-wide association analysis for segregating population duck plumage color

The duck samples were re-sequenced at a depth of 5×. A cohort of 188 ducks from a segregating population derived from Liancheng ducks and Pekin ducks was selected for GWAS analysis. Initially, using the genome of the Liancheng duck as a reference, the study identified two sites controlling the white feather phenotype on Chromosomes 13 and 33 (refer to Figure 3A). This research highlighted a specific gene on Chromosome 13 that regulates melanin synthesis in Liancheng ducks (refer to Figure S2, S3). Since the white plumage phenotype in Liancheng ducks did not show sex-related patterns, cytoplasmic inheritance considerations for white plumage were deemed unnecessary. Subsequently, the *Rr* gene was pinpointed to the 5.24-5.32Mb region of Chromosome 33 in Liancheng ducks (refer to Figure 3B), which contains potential candidate genes such as *PMEL*, *RAB5B*, *IKZF4*, *ERBB3*, *PA2G4*, *ZC3H10*, and *ESYT1* (refer to Figure 3C). Within the candidate region (Chr33: 5.24-5.32 Mb), four minimal recombination haplotypes were identified based on the parents and segregating populations from 117 SNPs with an Fst >0.8 (PK vs LC ducks). Only the haplotypes in block 4 (Chr33: 5,303,111-5,304,416, 101,305 bp) located upstream of the *PMEL* gene corresponded to the observed phenotypes (refer to Figure 3D). Additionally, a

significantly high peak in the Fst value was observed in the *RR* vs *rr* duck populations within the selected candidate region, while no peak was seen in *rr* vs *rr* duck populations (refer to Figure 3E).

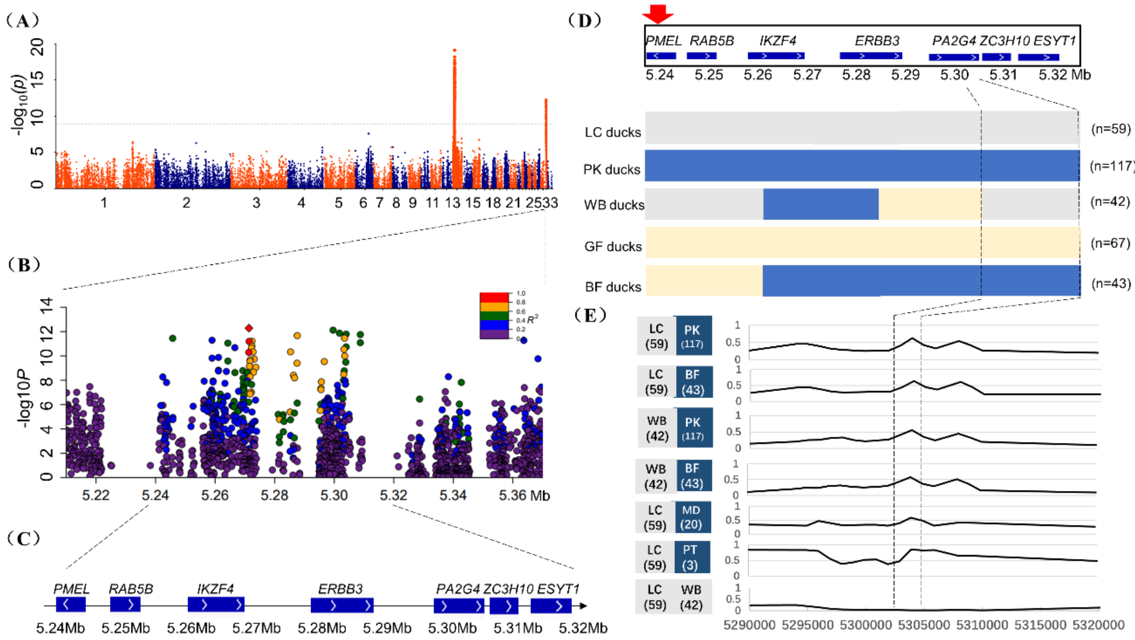

Figure 3. Screening for the candidate region associated with the white plumage of Liancheng ducks involved a GWAS on a cohort of 188 ducks from a cross between Pekin ducks and Liancheng ducks. (A) Manhattan plot showed the genetic effects on plumage color. (B) Locuszoom results highlighted site on chromosome 33 (5.21-5.37 Mb) linked to white plumage in Liancheng ducks. Genotypic SNPs were identified based on linkage imbalance values compared to the leading SNP in the intercross population duck (Chr33: 5,303,413). (C) Candidate genes in the region (5.24-5.32 Mb) were identified, with white and black arrows indicating gene orientation and Chromosome 33 direction, respectively. (D) Identity by Descent (IBD) analysis used color schemes to refine candidate regions, with blue for Pekin ducks and black-feathered ducks, grey for Liancheng ducks and white-feathered black beak ducks, and yellow for grey plumage ducks (LC vs PK, Fst> 0.8). (E) Genome divergence analysis among six duck breeds, including LC vs PK ducks, Black feather ducks (BF), Mallards (MD), and Putian (PT) ducks within the candidate region (Chr33: 5.29-5.32 Mb),

averaged Fst values in 10kb region in each comparison group.

PMEL causes melanin deposition in duck plumages

The region on Chromosome 13 was found to encompass the *MITF* gene in the GWAS analysis (refer to Figure S2). Upon comparison with the previous Pekin duck genome assembly (GCA_003850225.1), a 6.6 kb insertion within the *MITF* gene was identified, showing a strong correlation with melanin synthesis in ducks (refer to Table S9). Subsequent GWAS analysis, after removing the *MITF* signal, revealed a single signal on Chromosome 33 (refer to Figure S3). Candidate region included *PMEL*, *RAB5B*, *IKZF4*, *ERBB3*, *PA2G4*, *ZC3H10*, and *ESYT1*. Results indicated that only the *PMEL* gene showed significant differential expression (refer to Figure 4A, 4B), with higher expression levels in black-feathered ducks compared to grey-feathered ducks (-Log10(p)>30). RNA-seq results showed no expression of the *PMEL* gene in feather bulb specimens of white-feathered ducks (refer to Figure 4C, 4D). Other genes within the GWAS candidate range (Chr33: 5.24-5.32Mb) were excluded due to similar gene expression levels in different plumage populations or inconsistent gene expression patterns related to melanin regulation. qPCR results confirmed *PMEL* as the *Rr* gene (refer to Figure 4C). Notably, the *PMEL* gene exhibited an elevated average GC content of 72.4% (refer to Figure S4). Additionally, *PMEL* gene expression correlated with the plumage color phenotype of Liancheng ducks at various developmental stages, with high expression levels observed in skin tissue (refer to Figure 4B). This underscores the importance of establishing a high-quality genome for Liancheng ducks.

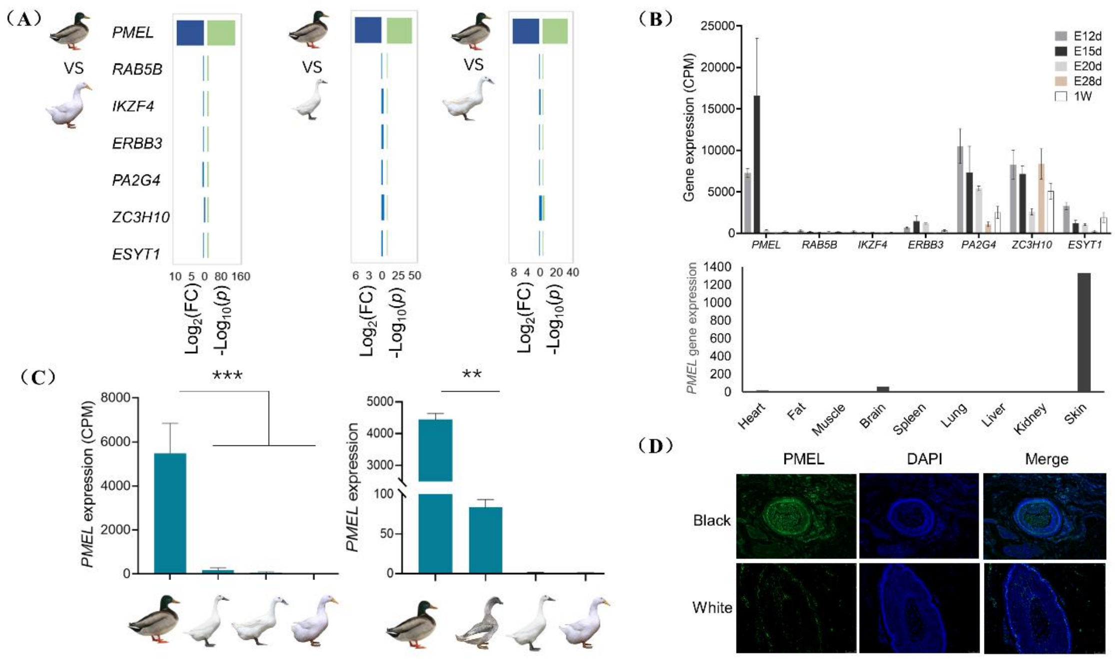

Figure 4. Identification of the candidate gene for white plumage in Liancheng ducks (A) Gene expression of seven GWAS candidate region genes (*PMEL*, *RAB5B*, *IKZF4*, *ERBB3*, *PA2G4*, *ZC3H10*, *ESYT1*) in 1-week-old feather follicle samples of white and black-feathered ducks, with three replicates per sample. $Log_2(FC)$ values were used to analyze gene expression differences in blue, and values where $-Log_{10}(p) > 30$ were shown in green. (B) Analysis of the expression levels of the seven candidate genes in skin tissues of Liancheng ducks at different developmental stages. E12d, E15d, E20d, and E28d (also the first day of birth) represent 12, 15, 20, and 28 days of the embryonic period, respectively. Data were shown as mean ± standard error (n=3). (C) CPM and qPCR results of *PMEL* expression in 1-week-old feather bulb samples of ducks. Data were presented as mean ± standard error (n=3). ** $p<0.01$; *** $p<0.001$. (D) Immunofluorescence results showing PMEL distribution in feather bulb specimens of black- and white-feathered ducks. Black (Mallards) and White (Liancheng ducks).

The *Rr* variation was fine mapped to *PMEL* upstream regulatory region

The results of the IBD analysis indicated that only the haplotypes in block 4 (Chr33: 5,303,111-5,304,416, 101,305 bp) located upstream of the *PMEL* gene were associated with the observed phenotypes (refer to Figure 3D). Furthermore, a significantly high peak in the *RR* vs *rr* duck populations within the selected candidate region (refer to Figure 3E), providing supporting evidence for this region (Chr33: 5,303,111-5,304,416, 101,305 bp) as the *Rr* locus. Among the identified candidate variations in this region, one copy number variation (CNV) (refer to Figure S5 and S6), were initially excluded. Applying a threshold of Fst > 0.8 (PK vs LC ducks), only 12 SNP variants and 2 Indels were retained (refer to Table S10). It is noteworthy that all 12 SNP variations were found in the upstream regulatory region of the *PMEL* gene (Chr33: 5,239,969-5,244,318). Finally, two SNPs (Chr33: 5,303,994A>G; 5,303,997A>G) were identified as the potential causal variants across all duck breeds in this study (Table S10). Intriguingly, these two SNPs were observed to be in complete linkage disequilibrium. Additionally, the Hi-C results illustrated that the *PMEL* gene and its upstream region, which encompassed the two candidate SNPs, were situated within a topologically associated domain (TAD) region (refer to Figure 5).

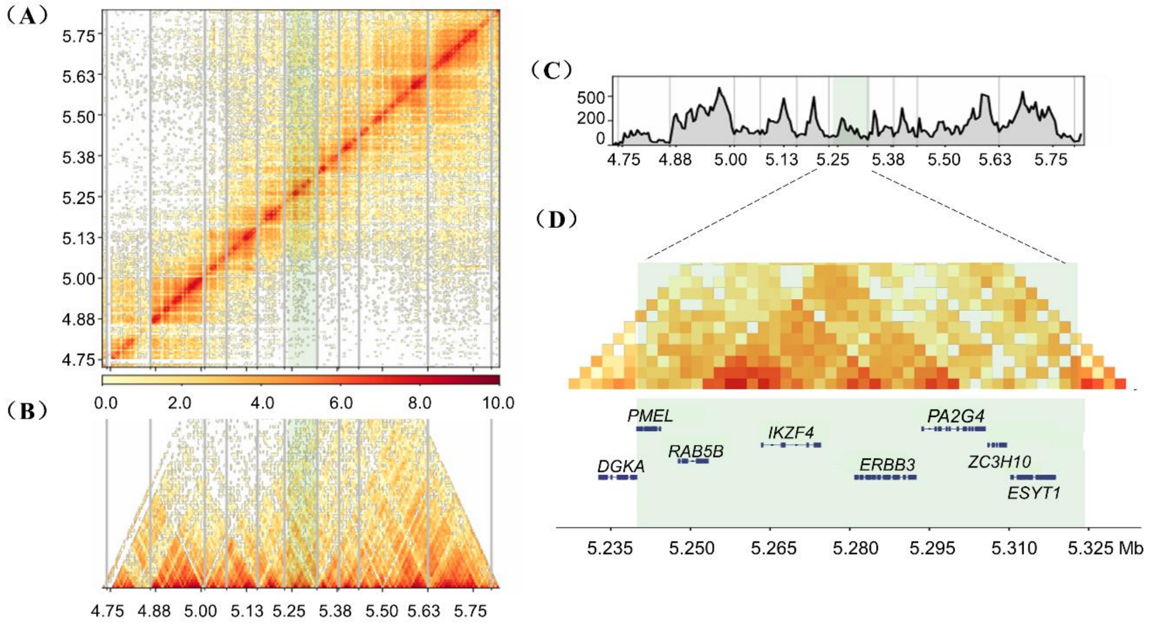

Figure 5. Chromosome interaction mapping (Hi-C) result of the end of chromosome 33 (4.75-

5.8Mb) in skin fat of Liancheng ducks. (A) Log_2(interaction matrix) analysis of Chr33 (resolution:5kb). Strong contacts were shown in red, and weak contacts were shown in white. The heat map showed a normalized contact matrix in 5kb bins. Light green indicated the *Rr* site candidate region by GWAS analysis. (B) Triangular result of Log_2 (interaction matrix) of Chr33 (4.75-5.75Mb) and the (C) topologically associated domain (TAD)-like and boundary-like regions were identified with the default algorithm built in HiCPlotter at a resolution of 5kb. (D) Log_2 (interaction matrix) of Chr33 (5.235-5.325Mb). Candidate regions identified from Chr33:5.24-5.32Mb by GWAS result and genes within it (light green regions).

Functional analysis of two candidate SNPs

Considering the location of the two candidate SNPs (SNP1 and SNP2) (Chr33: 5,303,994A>G; 5,303,997A>G) in the noncoding upstream region of the *PMEL* gene, we assessed their promoter and enhancer effects using pGL3 luciferase vectors (refer to Figure 6A, 6B). Both pGL3 vectors (pGL3-basic-white and pGL3-basic-black) with inserts showed minimal luciferase activity in duck embryo fibroblast (DEF) cells and human melanoma cells (A375) (refer to Figure 6C), indicating no promoter activity at these SNP sites. However, for enhancer activity, the vectors with inserts (pGL3-promoter-white and pGL3-promoter-black) displayed significantly different luciferase activity in DEF cells and A375 cells (ANOVA, $p<0.01$). Notably, SNP2 (Chr33: 5,303,997A>G) revealed that both pGL3-promoter-white-1mut and pGL3-promoter-white-2mut exhibited higher luciferase activity than pGL3-promoter-white (refer to Figure 6D), indicating a synergistic enhancement activity by the black alleles of variations SNP1 and SNP2.

Analysis conducted on the JASPAR transcription prediction website (https://jaspar.elixir.no/) revealed that multiple transcription factors may bind differently to sequences surrounding candidate SNPs located at Chr33: 5,303,944-5,304,098. The results suggested that variations in SNP1 and SNP2 could impact the binding of various transcription factors, as illustrated in Figure S7. Furthermore, differential expression of the sox5 transcription factor was observed in the feather follicles of white- and black-feathered ducks. These findings led to the hypothesis that variations in SNP1 and SNP2 could potentially be key mutations responsible for the white feather phenotype.

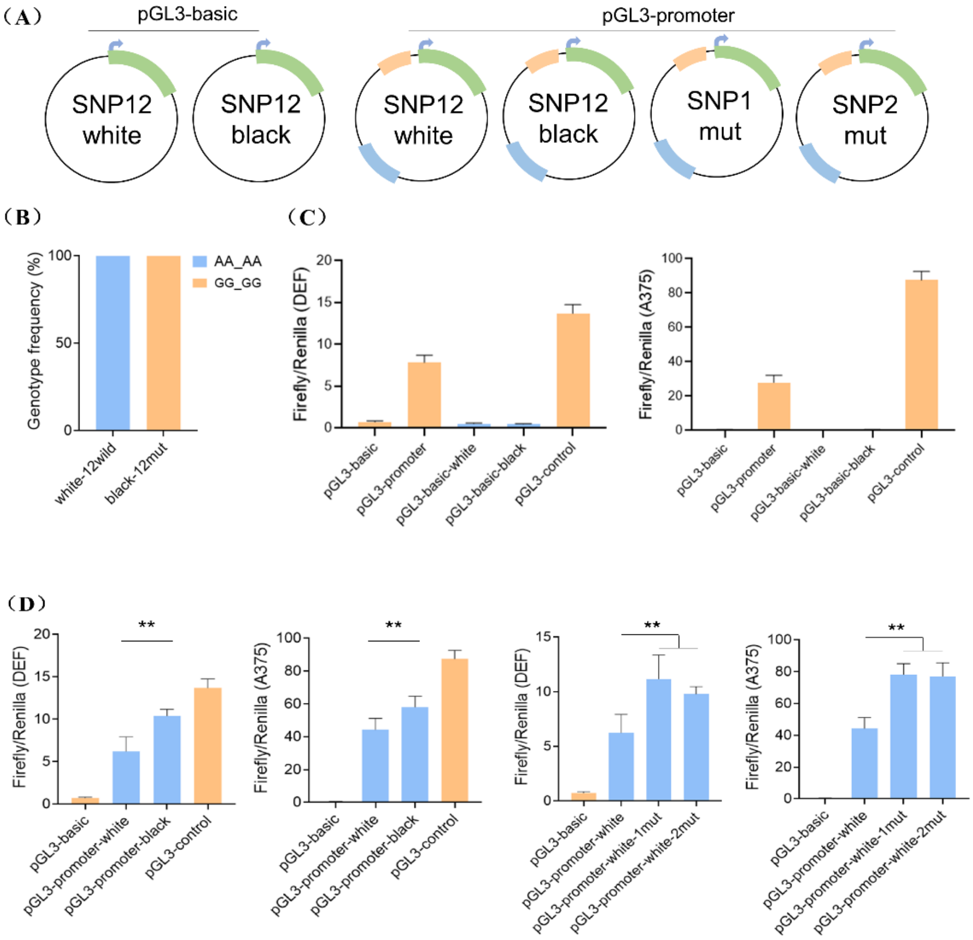

Figure 6. Functional analysis of the candidate variation controlling the white plumage phenotype of Liancheng duck. (A) Diagram of six pGL3 vectors for luciferase reporter gene experiment. Candidate SNP1 and SNP2 (Chr33:5,303,994A>G and 5,303,997A>G) of Liancheng duck and

Pekin duck were inserted into empty pGL3-basic and pGL3- promoter vectors. pGL3-basic, pGL3-promoter and pGL3-control were used as negative and positive control group. Insertion fragment were marked in yellow so as to verify the promoter activity. The blue box represented insertion fragment of the PGL3-promoter to verify enhancer activity. Additionally, vectors also contained the green box (luciferase reporter gene) and the blue arrow (indicate transcription site). (B) Genotype distribution of SNP1, SNP2 in Liancheng duck (AA_AA, n=59) and Pekin duck (GG_GG, n=117). (C) Validation of promoter activity of homozygous wildtype (AA_AA) and homozygous mutation (GG_GG) in DEF cells and A375 cells. Data were presented as mean ± standard error (n=8), (ANOVA, p>0.05). (D) Enhancer activity of AA_AA, GG_GG, GG_AA, and AA_GG vectors in DEF cells and A375 cells. Data were presented as mean ± standard error (n=8), (ANOVA, ** p<0.01).

Discussion

The white plumage phenotype is a common trait observed in various avian species, such as chickens [38, 39], peafowl [40], geese [41], also in ducks [2, 42]. Extensive research has focused on the unique white plumage phenotype of Liancheng ducks [42, 43, 44]; however, the genetic mechanisms behind its plumage color remain unclear. Our findings suggest that the inheritance of plumage color phenotype in Liancheng ducks is likely governed by two autosomal genes, independent of sex chromosomes (Table 1). Despite using the previous Pekin duck reference genome (IASCAAS_PekingDuck_PBH1.5, GCA_003850225.1), our GWAS analysis identified three signals (refer to Figure S8), which we believed may be due to incomplete assembly. To further investigate the white feather phenotype of Liancheng ducks, we first established a high-quality

genome for Liancheng duck (refer to Figure 1). This new reference genome has a size of 1.29 Gb, with contig and scaffold N50 values of 12.17 and 83.98Mb, respectively (refer to Table S5). The scaffold N50 length of the Liancheng duck genome is higher than that of other duck genomes [45, 46, 47, 48], representing a more complete genome assembly that better reflects the continuity of the duck genome. However, the chromosomes number identified from this newly genome still needs improvement compared to those of the Muscovy duck and Crested Duck [49, 50]. Further pan-genome and functional gene-mining analysis can be conducted in the future [29, 47, 51]. Taken together, these findings firstly represent the first construction of the Liancheng duck genome, resulting in enhanced genome contiguity compared to previous duck genomes.

MITF regulates the expression of enzymes responsible for melanin synthesis and the expression of receptors involved in melanocyte function [52, 53, 54]. *MITF* produces various isoforms through alternative promoters with shared coding exons but distinct amino termini [55]. While *MITF* variants are known to influence melanin regulation, the regulation of these isoforms remained unclear. In ducks, we discovered the expression of two *MITF* isoforms, *MITF-B* and *MITF-M*, with only the latter being crucial for melanin synthesis in duck plumage [2,9, 56]. *MITF-M* isoforms also have been shown to regulate white coloration in the fur of dogs [57], llamas [58], and mice [59]. Previous research found SNPs, indels, and structural variants in *MITF* as possible causes of white plumage in ducks [60]. Two synonymous SNPs (c.114T>G and c.147T>C) and a 14-bp indel (GCTGCAAAC AGATG) in intron 7 of duck *MITF* were significantly associated with the black- and white-colored breeds (*p*<0.001) [61]. One variant in the 5'UTR of *MITF* were significantly associated with feather color phenotypes in geese [62]. A 6.6 kb insertion within the *MITF* gene

demonstrated a strong correlation with melanin production in ducks [2] and indicated that *MITF* played an on-off role in the melanin generation pathway of Pekin ducks. *MITF* can promote differentiation-related functions, including regulation of genes involved in pigmentation, such as *PMEL*, *TYR*, *TYRP1*, *DCT*, *MLANA*, *SILV*, and *SLC24A5* [60]. In the years following the separation of *MITF* gene, the number of potential target genes increased sharply. Based on GWAS analysis, it was confirmed that the *MITF* gene acts as an epistatic gene controlling melanin synthesis in Liancheng ducks, aligning with previous research findings [44]. This highlights the significant regulatory role of *MITF* in melanin synthesis in Liancheng ducks and underscores its importance as a key genetic factor in pigmentation.

PMEL, a type I transmembrane transport glycoprotein, is synthesized in the endoplasmic reticulum and plays a crucial role in amyloid fiber formation during stages I and II of melanosome formation in the L-DOPA pathway [63, 64]. After synthesis, PMEL is transported to melanosomes, where it undergoes proteolytic processing to form fibrils [65]. These fibrils act as a scaffold for the deposition of melanin pigments, catalyzed by enzymes like tyrosinase [66, 67]. Mutations in the *PMEL* gene can lead to abnormalities in melanosome formation and melanin deposition, impacting plumage coloration in various bird species, including chickens [38, 68], Junco hyemalis [69], Japanese quail [70] and Indian peafowl [40]. To date, only 21 bird species have annotated the *PMEL* gene among 120 bird genomes (Table S11). However, the association between the *PMEL* gene and duck plumage color phenotype has not been previously explored.

In this study, the *PMEL* gene was found to be significantly differentially expressed between the

feather bulb specimens of white- and black-feathered ducks (ANOVA, $p<0.001$). Immunofluorescence results indicated high expression of the PMEL protein in feather follicle specimens of black and grey plumage ducks, contrasting with low expression in white plumage ducks. Many studies suggested that the deposition of feather melanin may involve the *PMEL* gene [69, 71, 72, 73]. Meanwhile, the *PMEL* gene is also implicated in the formation of white feathers in quail [74] and in the white feathers of chickens at the hatch stage [75]. Endogenous *PMEL* expression is regulated by *MITF*, with alterations observed in melanoma cells [76]. However, the specific interplay between these two genes in determining the plumage color of Liancheng ducks requires further investigation. Overall, we have provided the first annotation of the *PMEL* gene in the newly sequenced duck genome (Figure S4, Table S12), previously believed to lack this gene. *MITF* functions as a key gene that governs melanin production in ducks. Subsequent inactivation of the *PMEL* gene, situated in the feather bulb specimens resulting in the distinctive white feather and black feathers observed in Liancheng ducks.

In the candidate region identified through GWAS analysis, we investigated a total of 12 SNPs, 2 Indel variations, and 1 CNV variation (refer to Table S10; Figures S7 and S8). Among these variants, only 2 SNPs (Chr33:5,303,994A>G and 5,303,997A>G) were found to be consistently associated with the observed plumage color phenotypes across multiple breeds. Experimental data from promoter activity assays suggested that these SNPs may not act as promoters controlling *PMEL* gene expression (refer to Figure 6C). Instead, our genetic findings indicated that these two linked SNP variations, located in the upstream region of the *PMEL* gene, exhibited functional enhancer activity that might remotely regulate *PMEL* gene expression (refer to Figures 5 and 6). This remote

regulation likely impacts *PMEL* gene expression, resulting in the black-feather coloration seen in ducks, which aligns with the melanin phenotype of duck feathers.

Remote regulation elements are thought to interact with target promoters through physical proximity [77], but the exact impact of this proximity on function remains uncertain. In our research, we identified potential regions encompassing *PMEL* and two candidate SNPs within a single topologically associated domain region (Figure 5), suggesting that this area may be part of a genomic region with frequent interactions. The process of loop extrusion not only promotes interactions within the TAD but also shields the TAD from external influences [78]. Furthermore, enhancer-promoter interactions may intensify during mammalian development [79], potentially accounting for the variation in plumage colors of Liancheng ducks from embryonic to postnatal stages. We also noted that only sox5, the predicted transcription factor, displayed varying expression levels in feather bulbs of different plumage colors (Figure S7). However, it is worth mentioning that other transcription factors could also play a role in the regulatory mechanism. Additionally, the gray plumage, an intermediate phenotype observed in our study, may be linked to a haploinsufficiency effect [80].

Feather phenotype is a complex trait composed of a series of stratified modules [81]. Birds can be highly decorated with distinct and colorful pigmentation patterns, which are used to attract a mate or to hide from or frighten a potential predator. Melanin plays an important role in the formation of feather pigments, which is achieved through the regulation of the presence, distribution, and differentiation of these melanocytes. Recent studies have reported that the variation of *MITF*, *PMEL*,

TYR, *EDNRB2*, *SLC45A2*, *MC1R* genes and Agouti signaling protein can regulate the production of feather melanin in ducks [2, 82]. However, the role of *PMEL* gene in feather melanin formation in ducks has not been confirmed. Our study is the first to report the role of the *PMEL* gene in pigment formation in duck feathers and provide valuable insights into the genetic mechanisms underlying plumage coloration, as well as promising practical implications for selective breeding and conservation efforts.

Data Availability

The whole-genome sequence data reported in this article have been deposited in the NCBI under accession number No. PRJNA1107839. The resequencing raw data have been deposited in the NCBI SRA under accession No. PRJNA844232. The transcriptomic raw data have been deposited in the NCBI under accession No. PRJNA1109286.

Additional Files

Supplementary Figure 1. Phenotypic characteristics of 1,281 F2 ducks from Liancheng and Pekin ducks.

Supplementary Figure 2. Screening for the candidate region associated with the white plumage of Liancheng ducks by GWAS in 188 ducks from a cross between Liancheng and Pekin ducks.

Supplementary Figure 3. Manhattan plot showing the genetic effects on the plumage color according to a GWAS from a cross of Liancheng and Pekin ducks except WY ducks based on Liancheng duck genome (GCA_039998735.1).

Supplementary Figure 4. The collinearity analysis of comparison between the *PMEL* gene and its

upstream and downstream 100kb region in Liancheng duck and other birds.

Supplementary Figure 5. Illustration of the read depth analysis that confirmed the copy number variations on GWAS candidate region (Chr33: 5.24-5.32Mb).

Supplementary Figure 6. Genotypes of candidate CNV variation (Chr33:5,282,001- 5,284,500) in different plumage color populations.

Supplementary Figure 7. Heatmap cluster analysis of transcription factors.

Supplementary Figure 8. Manhattan plot showing the genetic effects on the plumage color according to a GWAS in ducks from a cross of Liancheng and Pekin ducks based on previous reference genome of Pekin duck (GCA_015476345.1).

Supplementary Figure 9. Phenotypic characteristics of Liancheng ducks, Pekin ducks, Mallards during embryonic and postnatal periods.

Supplementary Table 1. List of 366 duck genome resequencing used in the study.

Supplementary Table 2. List of 37 ducks for RNA-seq used in the study.

Supplementary Table 3. The information on the primers used for qPCR.

Supplementary Table 4. Table Summary of Hi-C reads mapping results.

Supplementary Table 5. The Genome Scaffolds and Contigs information of Liancheng duck genome (GCA_039998735.1).

Supplementary Table 6. Assembly statistics of the Liancheng duck genome and previous duck genomes.

Supplementary Table 7. Complete evaluation table of Liancheng duck genome BUSCOs.

Supplementary Table 8. Summary of chromosome regions on the genome.

Supplementary Table 9. Frequency distribution of 6.6kb insertion within *MITF* gene in duck population.

Supplementary Table 10. Genotypic distribution of SNP candidate variations in different duck breeds based on reference genome Liancheng duck.

Supplementary Table 11. All birds with *PMEL* genes in their avian genomes.

Supplementary Table 12. The blast results of sequence identity between the newly annotated mRNA sequence of *PMEL* gene in Liancheng duck and other birds.

Abbreviations

LC: Liancheng duck; PK: Pekin duck; WB: The white-feathered black beak and webbing duck; GF: The gray-feathered black beak and webbing duck; BF: The black-feathered black beak and webbing duck; WY: The white-feathered yellow beak and webbing duck; MD: Mallard; PT: Putian ducks; BLAST: Basic Local Alignment Search Tool; bp: Base pairs; kb: Kilobase pairs; Mb: Megabase pairs; Gb: Gigabase pairs; FC: Fold change; IBD: Identity By Descent; BUSCO: Benchmarking universal single copy orthologs; BWA: Burrows wheeler aligner; NCBI: National Center for Biotechnology Information; PacBio: Pacific Biosciences; HiFi: High Fidelity reads; Hi-C: High-throughput/resolution chromosome conformation capture; RNA-seq: RNA sequencing; SNP: Single nucleotide polymorphism; SRA: Sequence Read Archive; PASA: Program to Assemble Spliced Alignments; Go: Gene Ontology; KEGG: Kyoto Encyclopedia of Genes and Genomes; NR: Non-Redundant Protein Database; eggNOG: Evolutionary genealogy of genes: Non-supervised Orthologous; SWISS-PROT: Swiss-Prot Protein Sequence Database; MAF: Minor allele frequency; PCA: Principal component analysis; ΔAF: Allele frequency difference; CPM: Counts per million;

Indel: Insertion and deletion; CNV: Copy number variation; Fst: Fixation index; GC: Guanine-cytosine; GWAS: Genome-wide association study; TAD: Topologically associated domain; DEF: Duck embryo fibroblast; A375: Human melanoma cells.

Funding

This work was supported by grants from the National Science Fund for Distinguished Young Scholars (32325047), the Innovation Program of Chinese Academy of Agricultural Sciences(CAAS--SCAB-202302), the China Agriculture Research System of MOF and MARA (CARS-42-05), and the National Key R&D Program of China (2022YFF1000102). The authors were grateful to Shandong Rongda Agricultural Development Co., Ltd for their help in this sample collection.

Competing Interests

The authors have declared no competing interests.

Ethics Statement

All animals used in the study were treated following the guidelines for the experimental animals established by the Council of China Animal Welfare. Protocols of the experiments were ap-proved by the Science Research Department of the Institute of Animal Sciences, Chinese Academy of Agricultural Sciences (CAAS) (Beijing, China).

Authors' Contributions

Z.Zhou. and S.Hou conceived the project, designed the research, and managed the project. Z.Guo., Z.Wang., H.Tang., H.Zhang. constructed the population. T.Liu., D.Liu. and Z.Wang. collected the phenotype data. Z.Wang., S.Yu. and H.Liu. performed the genome assembly. Z.Wang., B.Zhang. and J.Cao. performed the experiments. Z.Wang., H.Liu., D.Liu. and Q.Mou. performed bioinformatics analysis. Z.Wang., M.Schroyen. and Z.Zhou. wrote the manuscript.

References

1.Ito S, Wakamatsu K. Quantitative analysis of eumelanin and pheomelanin in humans, mice, and other animals: a comparative review. *Pigment Cell Res*. 2003;**16**:523-531.

2.Zhou Z, Li M, Cheng H, et al. An intercross population study reveals genes associated with body size and plumage color in ducks. *Nat Commun*. 2018;**9**:2648.

3.Land EJ, Riley PA. Spontaneous redox reactions of dopaquinone and the balance between the eumelanic and phaeomelanic pathways. *Pigment Cell Res*. 2000;**13**:273-277.

4.Wang Z, Guo Z, Mou Q, et al. Unique feather color characteristics and transcriptome analysis of hair follicles in Liancheng White ducks. *Poult Sci*, 2024;**103**:103794.

5.Haase E, Ito S, Wakamatsu K. Influences of sex, castration, and androgens on the eumelanin and pheomelanin contents of different feathers in wild mallards. *Pigment Cell Res*. 1995;**8**:164-170.

6.Huang Y, Li Y, Burt DW, et al. The duck genome and transcriptome provide insight into an avian influenza virus reservoir species. *Nat Genet*. 2013;**45**:776-783.

7.Li J, Zhang J, Liu J, et al. A new duck genome reveals conserved and convergently evolved chromosome architectures of birds and mammals. *Gigascience*. 2021;**10**:giaa142.

8.Liu D, Zhang H, Yang Y, et al. Metabolome-Based Genome-Wide Association Study of Duck

Meat Leads to Novel Genetic and Biochemical Insights. *Adv Sci*. 2023;**10**:e2300148.

9.Zhu F, Yin ZT, Wang Z, et al. Three chromosome-level duck genome assemblies provide insights into genomic variation during domestication. *Nat Commun*. 2021;**12**:5932.

10.Yu S, Liu Z, Li M, et al. Resequencing of a Pekin duck breeding population provides insights into the genomic response to short-term artificial selection. *Gigascience*. 2023;**12**:giad016.

11.Wang K, Hua G, Li J, et al. Duck pan-genome reveals two transposon insertions caused bodyweight enlarging and white plumage phenotype formation during evolution. *IMeta*. 2024;**3**,e154.

12.Liu H, Xi Y, Tang Q, et al. Genetic fine-mapping reveals single nucleotide polymorphism mutations in the MC1R regulatory region associated with duck melanism. *Mol Ecol*. 2023;**32**:3076-3088.

13.Wenger, A.M., Peluso, P., Rowell, W.J. et al. Accurate circular consensus long-read sequencing improves variant detection and assembly of a human genome. *Nat Biotechnol.* 2019; **37**:1155–1162.

14.Cooke TF, Fischer CR, Wu P, et al. Genetic Mapping and Biochemical Basis of Yellow Feather Pigmentation in Budgerigars. *Cell*. 2017;**171**:427-439.

15.Si S, Xu X, Zhuang Y, et al. The genetics and evolution of eye color in domestic pigeons (Columba livia). *PLoS Genet*. 2021;**17**:e1009770.

16. Li S, Wang C, Yu W, et al. Identification of genes related to white and black plumage formation by RNA-Seq from white and black feather bulbs in ducks. *PLoS One*. 2012;**7**:e36592.

17. Lin R, Lin W, Zhou S, et al. In'tegrated Analysis of mRNA Expression, CpG Island Methylation, and Polymorphisms in the MITF Gene in Ducks (Anas platyrhynchos). *Biomed Res Int*.

2019;**2019**:8512467.

18. Cheng H, Concepcion GT, Feng X, Zhang H, Li H. Haplotype-resolved de novo assembly using phased assembly graphs with hifiasm. *Nat Methods*. 2021;**18**:170-175.

19. Durand NC, Shamim MS, Machol I, et al. Juicer Provides a One-Click System for Analyzing Loop-Resolution Hi-C Experiments. *Cell Syst*. 2016;**3**:95-98.

20. Zheng Z, Lai Z, Wu B, et al. The first high-quality chromosome-level genome of the Sipuncula Sipunculus nudus using HiFi and Hi-C data. *Sci Data*. 2023;**10**:317.

21. Dudchenko O, Batra SS, Omer AD, et al. De novo assembly of the Aedes aegypti genome using Hi-C yields chromosome-length scaffolds. *Science*. 2017;**356**:92-95.

22. Manni M, Berkeley MR, Seppey M, Zdobnov EM. BUSCO: Assessing Genomic Data Quality and Beyond. *Curr Protoc*. 2021;**1**:e323.

23. Powell S, Forslund K, Szklarczyk D, et al. eggNOG v4.0: nested orthology inference across 3686 organisms. *Nucleic Acids Res*. 2014;**42**:D231-D239.

24. Bolger AM, Lohse M, Usadel B. Trimmomatic: a flexible trimmer for Illumina sequence data. *Bioinformatics*. 2014;**30**:2114-2120.

25. Grewal S, Yang CY, Scholefield D, et al. Chromosome-scale genome assembly of bread wheat's wild relative Triticum timopheevii. *Sci Data*. 2024;**11**:420.

26. Li H, Durbin R. Fast and accurate short read alignment with Burrows-Wheeler transform. *Bioinformatics*. 2009;**25**:1754-1760.

27. McKenna A, Hanna M, Banks E, et al. The Genome Analysis Toolkit: a MapReduce framework for analyzing next-generation DNA sequencing data. *Genome Res*. 2010;**20**:1297-1303.

28. Kang HM, Sul JH, Service SK, et al. Variance component model to account for sample structure

in genome-wide association studies. *Nat Genet*. 2010;**42**:348-354.

29. Wang K, Hua G, Li J, et al. Duck pan-genome reveals two transposon insertions caused bodyweight enlarging and white plumage phenotype formation during evolution. *Imeta*. 2023;**3**:e154.

30. Price AL, Patterson NJ, Plenge RM, Weinblatt ME, Shadick NA, Reich D. Principal components analysis corrects for stratification in genome-wide association studies. *Nat Genet*. 2006;**38**:904-909.

31. Anders S, Pyl PT, Huber W. HTSeq--a Python framework to work with high-throughput sequencing data. *Bioinformatics*. 2015;**31**:166-169.

32. Schmittgen TD, Livak KJ. Analyzing real-time PCR data by the comparative C(T) method. *Nat Protoc*. 2008;**3**:1101-1108.

33. Servant N, Varoquaux N, Lajoie BR, et al. HiC-Pro: an optimized and flexible pipeline for Hi-C data processing. *Genome Biol*. 2015;**16**:259.

34. Akdemir KC, Chin L. HiCPlotter integrates genomic data with interaction matrices. *Genome Biol*. 2015;**16**:198.

35. Guo Y, Gu X, Sheng Z, et al. A Complex Structural Variation on Chromosome 27 Leads to the Ectopic Expression of HOXB8 and the Muffs and Beard Phenotype in Chickens. *PLoS Genet*. 2016;**12**:e1006071.

36. Wang X, Zheng Z, Cai Y, et al. CNVcaller: highly efficient and widely applicable software for detecting copy number variations in large populations. *Gigascience*. 2017;**6**:1-12.

37. Chen L, Gu X, Huang X, et al. Two cis-regulatory SNPs upstream of ABCG2 synergistically cause the blue eggshell phenotype in the duck. *PLoS Genet*. 2020;**16**:e1009119.

38. Keeling L, Andersson L, Schütz KE, et al. Chicken genomics: feather-pecking and victim pigmentation. *Nature*. 2004;**431**:645-646.

39. Gunnarsson U, Kerje S, Bed'hom B, et al. The Dark brown plumage color in chickens is caused by an 8.3-kb deletion upstream of SOX10. *Pigment Cell Melanoma Res*. 2011;**24**:268-274.

40. Liu S, Chen H, Ouyang J, et al. A high-quality assembly reveals genomic characteristics, phylogenetic status, and causal genes for leucism plumage of Indian peafowl. *Gigascience*. 2022;**11**:giac018.

41. Xi Y, Wang L, Liu H, et al. A 14-bp insertion in endothelin receptor B-like (EDNRB2) is associated with white plumage in Chinese geese. *BMC Genomics*. 2020;**21**:162.

42. Wang L, Yang L, Yang S, et al. Identification of genes associated with feather color in Liancheng white duck using FST analysis. *Anim Genet*. 2022;**53**:518-521.

43. Gong Y, Yang Q, Li S, et al. Grey plumage colouration in the duck is genetically determined by the alleles on two different, interacting loci. *Anim Genet*. 2010;**41**:105-108.

44. Yang L, Mo C, Shen W, et al. The recessive C locus in the MITF gene plays a key regulatory role in the plumage colour pattern of duck (Anas platyrhynchos). *Br Poult Sci*. 2019;**60**:105-108.

45. Jiang F, Jiang Y, Wang W, et al. A chromosome-level genome assembly of Cairina moschata and comparative genomic analyses. *BMC Genomics*. 2021;**22**:581

46. Hu J, Song L, Ning M, et al. A new chromosome-scale duck genome shows a major histocompatibility complex with several expanded multigene families. *BMC Biol*. 2024;**22**:31.

47. Ng CS, Lai CK, Ke HM, et al. Genome Assembly and Evolutionary Analysis of the Mandarin Duck Aix galericulata Reveal Strong Genome Conservation among Ducks. *Genome Biol Evol*.

2022;**14**:evac083.

48. Lavretsky P, Hernández F, Swale T, et al. Chromosomal-level reference genome of a wild North American mallard (Anas platyrhynchos). *G3*. 2023;**13**:jkad171.

49. Xu MM, Gu LH, Lv WY, et al. Chromosome-level genome assembly of the Muscovy duck provides insight into fatty liver susceptibility. *Genomics*. 2022;**114**:110518.

50. Chang G, Yuan X, Guo Q, et al. The first crested duck genome reveals clues to genetic compensation and crest cushion formation. *Genomics Proteomics Bioinformatics*. 2023;**21**:483-500.

51. Mueller RC, Ellström P, Howe K, et al. A high-quality genome and comparison of short- versus long-read transcriptome of the palaearctic duck Aythya fuligula (tufted duck). *Gigascience*. 2021;**10**:giab081.

52. Dürig N, Letko A, Lepori V, et al. Two MC1R loss-of-function alleles in cream-coloured Australian Cattle Dogs and white Huskies. *Anim Genet*. 2018;**49**:284-290.

53. Guo Q, Jiang Y, Wang Z, et al. Genome-Wide Analysis Identifies Candidate Genes Encoding Feather Color in Ducks. *Genes*. 2022;**13**:1249.

54. Pan R, Hua T, Guo Q, et al. Identification of SNPs in MITF associated with beak color of duck. *Front Genet*. 2023;**14**:1161396.

55. Karlsson EK, Baranowska I, Wade CM, et al. Efficient mapping of mendelian traits in dogs through genome-wide association. *Nat Genet*. 2007;**39**:1321-1328.

56. Lin R, Zhao F, Xiong T, et al. Genetic mapping identifies SNP mutations in MITF-M promoter associated with melanin formation in Putian black duck. *Poult Sci*. 2024;**103**:103191.

57. Baranowska Körberg I, Sundström E, Meadows JR, et al. A simple repeat polymorphism in the

MITF-M promoter is a key regulator of white spotting in dogs. *PLoS One*. 2014;**9**:e104363.

58. Anello M, Daverio MS, Silbestro MB, Vidal-Rioja L, Di Rocco F. Characterization and expression analysis of KIT and MITF-M genes in llamas and their relation to white coat color. *Anim Genet*. 2019;**50**:143-149.

59. Flesher JL, Paterson-Coleman EK, Vasudeva P, et al. Delineating the role of MITF isoforms in pigmentation and tissue homeostasis. *Pigment Cell Melanoma Res*. 2020;**33**:279-292.

60. Coding CR, Arnheiter H. MITF-the first 25 years. *Genes Dev*. 2019;**33**:983-1007.

61. Sultana H, Seo D, Choi NR, et al. Identification of polymorphisms in MITF and DCT genes and their associations with plumage colors in Asian duck breeds. *Asian-Australas J Anim Sci*. 2018;**31**:180-188.

62. Ren S, Lyu G, Irwin DM, et al. Pooled sequencing analysis of geese (Anser cygnoides) reveals genomic variations associated with feather color. *Front Genet*. 2021;**12**:650013.

63. Kerje S, Sharma P, Gunnarsson U, et al. The Dominant white, Dun and Smoky color variants in chicken are associated with insertion/deletion polymorphisms in the PMEL17 gene. *Genetics*. 2004;**168**:1507-1518.

64. Batai K, Cui Z, Arora A, et al. Genetic loci associated with skin pigmentation in African Americans and their effects on vitamin D deficiency. *PLoS Genet*. 2021;**17**:e1009319.

65. Watt B, Tenza D, Lemmon MA, et al. Mutations in or near the transmembrane domain alter PMEL amyloid formation from functional to pathogenic. *PLoS Genet*. 2011;**7**:e1002286.

66. Hurbain I, Geerts WJ, Boudier T, et al. Electron tomography of early melanosomes: implications for melanogenesis and the generation of fibrillar amyloid sheets. *Proc Natl Acad Sci U S A*. 2008;**105**:19726-19731.

67. Watt B, van Niel G, Raposo G, Marks MS. PMEL: a pigment cell-specific model for functional amyloid formation. *Pigment Cell Melanoma Res*. 2013;**26**:300-315.

68. Deng Y, Qu X, Yao Y, Li M, He C, Guo S. Investigating the impact of pigmentation variation of breast muscle on growth traits, melanin deposition, and gene expression in Xuefeng black-bone chickens. *Poult Sci*. 2024;**103**:103691.

69. Abolins-Abols M, Kornobis E, Ribeca P, et al. Differential gene regulation underlies variation in melanic plumage coloration in the dark-eyed junco (Junco hyemalis). *Mol Ecol*. 2018;**27**:4501-4515.

70. Ishishita S, Takahashi M, Yamaguchi K, et al. Nonsense mutation in PMEL is associated with yellowish plumage colour phenotype in Japanese quail. *Sci Rep*. 2018;**8**:16732.

71. Liu X, Zhou R, Peng Y, et al. Feather follicles transcriptome profiles in Bashang long-tailed chickens with different plumage colors. *Genes Genomics*. 2019;**41**:1357-1367.

72. Zheng X, Zhang B, Zhang Y, et al. Transcriptome analysis of feather follicles reveals candidate genes and pathways associated with pheomelanin pigmentation in chickens. *Sci Rep*. 2020;**10**:12088.

73. Heo S, Cho S, Dinh PTN, et al. A genome-wide association study for eumelanin pigmentation in chicken plumage using a computer vision approach. *Anim Genet*. 2023;**54**:355-362.

74. Yuan Z, Zhang X, Pang Y, et al. Association analysis of PMEL gene expression and single nucleotide polymorphism with plumage color in quail. *Anim Biotechnol*. 2023;**34**:5001-5010.

75. Hua G, Chen J, Wang J, et al. Genetic basis of chicken plumage color in artificial population of complex epistasis. *Anim Genet*. 2021;**52**:656-666.

76. Falletta P, Bagnato P, Bono M, et al. Melanosome-autonomous regulation of size and number:

the OA1 receptor sustains PMEL expression. *Pigment Cell Melanoma Res*. 2014;**27**:565-579.

77. Soldner F, Stelzer Y, Shivalila CS, et al. Parkinson-associated risk variant in distal enhancer of α-synuclein modulates target gene expression. *Nature*. 2016;**533**:95-99.

78. Hung TC, Kingsley DM, Boettiger AN. Boundary stacking interactions enable cross-TAD enhancer-promoter communication during limb development. *Nat Genet*. 2024;**56**:306-314.

79. Chen Z, Snetkova V, Bower G, et al. Increased enhancer-promoter interactions during developmental enhancer activation in mammals. *Nat Genet*. 2024;**56**:675-685.

80. Billiard S, Castric V, Llaurens V. The integrative biology of genetic dominance. *Biol Rev Camb Philos Soc*. 2021;**96**:2925-2942.

81. Terrill RS, Shultz AJ. Feather function and the evolution of birds. *Biol Rev Camb Philos Soc*. 2023;**98**:540-566.

82. Ng CS, Li WH. Genetic and Molecular Basis of Feather Diversity in Birds. *Genome Biol Evol*. 2018;**10**:2572-2586.

Click here to access/download
Supplementary Material
Responses to Review Comments.docx

Click here to access/download
Supplementary Material
Supplementary-Figures.docx

Click here to access/download
Supplementary Material
Supplementary-Tables.xlsx
