# Supplementary material for: A high-quality assembly revealing the PMEL gene for the unique plumage phenotype in Liancheng ducks
Source: Gigascience. 2025 Jan 13;14:giae114. doi: 10.1093/gigascience/giae114 (PMC11727711; doi:10.1093/gigascience/giae114)
Supplement: giae114_GIGA-D-24-00213_Revision_2 [file giae114_giga-d-24-00213_revision_2.pdf]

GigaScience

A high-quality assembly revealing PMEL gene for unique plumage phenotype of Liancheng ducks

--Manuscript Draft--

**Manuscript Number:**	GIGA-D-24-00213R2
**Full Title:**	A high-quality assembly revealing PMEL gene for unique plumage phenotype of Liancheng ducks
**Article Type:**	Research
**Funding Information:**	National Science Fund for Distinguished Young Scholars (32325047) — Prof. Zhengkui Zhou
**Abstract:**	Background: Plumage coloration is a distinctive trait in ducks, and the Liancheng duck, characterized by its white plumage and black beak and webbed feet, serves as an excellent subject for such studies. However, academic comprehension of the genetic mechanisms underlying duck plumage coloration remains limited. To this end, the Liancheng duck genome (GCA_039998735.1) was hereby de novo assembled using HiFi reads, and F2 segregating populations were generated from Liancheng and Pekin ducks. The aim was to identify the genetic mechanism of white plumage in Liancheng ducks.<br>Results: In this study, 1.29 Gb Liancheng duck genome was de novo assembled, involving a contig N50 of 12.17 Mb and a scaffold N50 of 83.98 Mb. Beyond the epistatic effect of the MITF gene, GWAS analysis pinpointed a 0.8Mb genomic region encompassing the PMEL gene. This gene encoded a protein specific to pigment cells and was essential for the formation of fibrillar sheets within melanosomes, the organelles responsible for pigmentation. Additionally, Linkage Disequilibrium (LD) analysis revealed two candidate SNPs (Chr33:5,303,994A>G; 5,303,997A>G) that might alter PMEL transcription, potentially influencing plumage coloration in Liancheng ducks.<br>Conclusions: Our study has assembled a high-quality genome for the Liancheng duck and has presented compelling evidence that the white plumage characteristic of this breed is attributable to the PMEL gene. Overall, these findings offer significant insights and direction for future studies and breeding programs aimed at understanding and manipulating avian plumage coloration.<br>Keywords: duck, genome assembly, plumage color, PMEL, melanin
**Corresponding Author:**	Zhengkui Zhou<br>CAAS IAS: Chinese Academy of Agricultural Sciences Institute of Animal Science<br>Beijing, CHINA
**Corresponding Author Secondary Information:**	
**Corresponding Author's Institution:**	CAAS IAS: Chinese Academy of Agricultural Sciences Institute of Animal Science
**Corresponding Author's Secondary Institution:**	
**First Author:**	Zhen Wang
**First Author Secondary Information:**	
**Order of Authors:**	Zhen Wang
	Zhanbao Guo
	Hongfei Liu
	Tong Liu
	Dapeng Liu
	Simeng Yu
	Hehe Tang

Powered by Editorial Manager® and ProduXion Manager® from Aries Systems Corporation

	He Zhang
	Qiming Mou
	Bo Zhang
	Junting Cao
	Martine Schroyen
	Shuisheng Hou
	Zhengkui Zhou
**Order of Authors Secondary Information:**	
**Response to Reviewers:**	Dear Reviewer and editors,<br><br>Thank you for handing our manuscript. We thank you for their critical comments and valuable suggestions, which have significantly improved our manuscript. In the revised version, the language has been carefully revised based on your comments. Meanwhile, the sentences and grammar were checked by all authors and language editing company, hoping to meet the publication requirements of Gigascience magazine.<br><br>Below, we offer a point-by-point response and indicate the corresponding modified sections in the revised manuscript.<br><br>We hope that our current manuscript addresses the reviewer's concerns.<br><br>Sincerely,<br><br>All authors<br><br>Reviewer 2#<br>Reviewer #2: - English still below what is acceptable for publication<br>Response: Thank you for your comments. We have invited professors in the field to revise and polish the paper. Meanwhile, the sentences and grammar were rechecked by a language editing company, hoping to meet the publication requirements of Gigascience magazine. Thank you again for your suggestions.<br><br>Abstract<br>--------<br>L17: "Plumage color is a notable characteristic and has widely studied": has BEEN widely studied<br>Response: Thank you for your reminder. We have revised and polished this sentence.<br><br>L18: it is not clear what webbing is: the webbing of the duck's feet/paws? Please explain<br>Response: Thank you for your comments. The Liancheng (LC) duck possesses a distinctive phenotype marked by white feathers, black beak, and black webbed feet. It is recognized for its significant melanin deposition in the beak and webbed feet, primarily due to the involvement of eumelanin as the main pigment. We mainly focus on the melanin deposition of Liancheng duck beaks and webbed (the skin on the feet), revised the full text, similar papers use the same expression.<br>References:<br>Johansson, L., Norberg, R. Delta-wing function of webbed feet gives hydrodynamic lift for swimming propulsion in birds. Nature 424, 65–68 (2003).<br>S. B. A. Kashem, M. Tabassum and M. Chai, "A novel design of an amphibious robot having webbed feet as duck," 2017 International Conference on Computer and Drone Applications (IConDA), Kuching, Malaysia, 2017, pp. 17-21.<br><br>Background<br>----------<br>L65: genome assemblies (plural)<br>Response: Thank you for your comments. We have revised this sentence.

Powered by Editorial Manager® and ProduXion Manager® from Aries Systems Corporation

	L66: delete "related"<br>Response: We have revised this sentence.<br><br>L66: replace of with in: in ducks<br>Response: We have revised this sentence.<br><br>L67: adipose TISSUE deposition (or fat deposition)<br>Response: We have revised this sentence.<br><br>L79-80: "PacBio introduced high-fidelity reads (HiFi reads) WHICH can provide more accurate, continuous and complete genetic information, making THIS a key enabling technology for research"<br>Response: Thank you for your comments. We have revised this sentence.<br><br>M&M<br>---<br>L138-139: quality of which genome assembly? Please be specific and clear<br>Response: Thank you for your reminding. We have revised this sentence.<br><br>L153: against the databases GO, KEGG etc. (delete of)<br>Response: We have revised this sentence.<br><br>L170: delete "ere required to demonstrate a" SNPs with minor allele ...<br>Response: We have revised this sentence.<br><br>L171: I guess this is missing rate per SNP: please specify<br>Response: Thank you for your comments. We have revised this sentence.
**Additional Information:**	
**Question**	**Response**
Are you submitting this manuscript to a special series or article collection?	No
**Experimental design and statistics**<br><br>Full details of the experimental design and statistical methods used should be given in the Methods section, as detailed in our Minimum Standards Reporting Checklist. Information essential to interpreting the data presented should be made available in the figure legends.<br><br>Have you included all the information requested in your manuscript?	Yes
**Resources**<br><br>A description of all resources used, including antibodies, cell lines, animals and software tools, with enough information to allow them to be uniquely identified, should be included in the Methods section. Authors are strongly	Yes

Powered by Editorial Manager® and ProduXion Manager® from Aries Systems Corporation

encouraged to cite Research Resource Identifiers (RRIDs) for antibodies, model organisms and tools, where possible.<br><br>Have you included the information requested as detailed in our Minimum Standards Reporting Checklist?	
**Availability of data and materials**<br><br>All datasets and code on which the conclusions of the paper rely must be either included in your submission or deposited in publicly available repositories (where available and ethically appropriate), referencing such data using a unique identifier in the references and in the “Availability of Data and Materials” section of your manuscript.<br><br>Have you have met the above requirement as detailed in our Minimum Standards Reporting Checklist?	Yes

Manuscript
Click here to access/download;Manuscript;Manuscript_Dec 5.docx

A high-quality assembly revealing *PMEL* gene for unique plumage phenotype of Liancheng ducks

Zhen Wang[1,2], Zhanbao Guo[1], Hongfei Liu[1], Tong Liu[1], Dapeng Liu[1], Simeng Yu[1], Hehe Tang[1], He Zhang[1], Qiming Mou[1], Bo Zhang[1], Junting Cao[1], Martine Schroyen[2], Shuisheng Hou[1], Zhengkui Zhou[1]*

[1] State Key Laboratory of Animal Biotech Breeding, Institute of Animal Science, Chinese Academy of Agricultural Sciences, Beijing, China.

[2] Precision Livestock and Nutrition Unit, Gembloux Agro-Bio Tech, TERRA Teaching and Research Centre, University of Liège, Passage des Déportés 2, Gembloux 5030, Belgium.

Zhen Wang [0009-0002-8240-5249]; Hongfei Liu [0000-0003-0683-5415]; Dapeng Liu [0000-0001-5865-4937]; Simeng Yu [0000-0003-1093-546X]; Bo Zhang [0000-0003-0405-7664]; Junting Cao [0000-0001-9937-8107]; Martine Schroyen [0000-0003-3855-8141]; Shuisheng Hou; Zhengkui Zhou [0000-0002-5192-2465].

Abstract

Background: Plumage coloration is a distinctive trait in ducks, and the Liancheng duck, characterized by its white plumage and black beak and webbed feet, serves as an excellent subject for such studies. However, academic comprehension of the genetic mechanisms underlying duck plumage coloration remains limited. To this end, the Liancheng duck genome (GCA_039998735.1) was hereby *de novo* assembled using HiFi reads, and F2 segregating populations were generated from Liancheng and Pekin ducks. The aim was to identify the genetic mechanism of white plumage in Liancheng ducks.

Results: In this study, 1.29 Gb Liancheng duck genome was *de novo* assembled, involving a contig N50 of 12.17 Mb and a scaffold N50 of 83.98 Mb. Beyond the epistatic effect of the *MITF* gene, GWAS analysis pinpointed a 0.8Mb genomic region encompassing the *PMEL* gene. This gene encoded a protein specific to pigment cells and was essential for the formation of fibrillar sheets within melanosomes, the organelles responsible for pigmentation. Additionally, Linkage Disequilibrium (LD) analysis revealed two candidate SNPs (Chr33:5,303,994A>G; 5,303,997A>G) that might alter *PMEL* transcription, potentially influencing plumage coloration in Liancheng ducks.

Conclusions: Our study has assembled a high-quality genome for the Liancheng duck and has presented compelling evidence that the white plumage characteristic of this breed is attributable to the *PMEL* gene. Overall, these findings offer significant insights and direction for future studies and breeding programs aimed at understanding and manipulating avian plumage coloration.

Keywords: duck, genome assembly, plumage color, *PMEL*, melanin

Background

Plumage coloration, a diverse and conspicuous trait among bird species, is a valuable attribute for investigating natural and artificial selection. Melanin, the predominant pigment in avian plumage, occurs as a mix of eumelanin and phaeomelanin across various tissues, leading to the extensive color variation observed in birds [1,2]. Eumelanin deposition in plumage is responsible for black and brown coloration, constituting the predominant pigment in bird feathers [3]. Extensive research on eumelanin and melanin-related genes has significantly enhanced public understanding of avian plumage coloration, which is a captivating ornamental feature. The duck (*Anas platyrhynchos*)

(NCBI: txid8839), widely distributed globally, displays a continuum of plumage colors spanning white to black, possibly as an adaptation to varied ecological environments. Those diverse plumage color patterns make ducks a key animal model for pigmentation studies. Despite advancements in understanding the biological and evolutionary aspects of plumage coloration, the genetic basis of these colors in ducks remains poorly understood.

The Liancheng (LC) duck possesses a distinctive phenotype marked by white feathers, black beak, and black webbed feet. It is recognized for its significant melanin deposition in the beak and webbed feet, primarily due to the involvement of eumelanin as the main pigment [4]. The biosynthesis of eumelanin is a complex process that involves three critical steps: Initially, tyrosinase catalyzes the oxidation of tyrosine to dihydroxyphenylalanine (DOPA). Subsequently, an oxidase enzyme converts DOPA into dopaquinone. Ultimately, dopaquinone undergoes a series of cyclic transformations, resulting in the production of pigment and the formation of melanin [5]. This synthetic pathway holds much significance in eumelanin synthesis, particularly in the pigmentation of skin and feathers in ducks. In this process, *MITF* serves as a key target of various signal transduction pathways, also the main regulator of melanin production. However, the genetic basis of melanin deposition and the specific genes involved in the formation of white feathers in LC ducks remain to be further explored.

The initial draft of the duck assembly was first reported in 2013 [6]. Refinements to the Pekin (PK) duck genome have achieved a scaffold N50 length of up to 76.3 Mb by 2020, which now serves as a foundational resource for duck genome analysis [7]. Compared to the high quality and variety of the genome assemblies of other birds, especially the one for chickens, necessitate further improvements in duck assembly quality. Previous research on animal breeding and genetics in ducks

has primarily focused on meat quality [8], adipose tissue deposition [9], and muscle weight [10]. However, genetic mechanisms of plumage coloration remain largely unexplored, and no reference genome of the famous LC duck has been published, making an upgraded LC duck genome necessarily important to provide foundational data for relevant future studies. The accuracy of gene localization greatly depends on the quality of the genome assembly [9]. A recent study leveraging the widely utilized duck genome assembly (GCA_003850225.1) has pinpointed a 6.6kb intronic insertion within *MITF*. This insertion is suspected to influence splicing events, consequently resulting in the white feather phenotype observed in ducks [11]. Additionally, another study has uncovered four new single nucleotide polymorphisms (SNPs) in the *MC1R* regulator region associated with black plumage in ducks [12]. This partially explains the mechanism of melanin formation in duck feathers. However, questions regarding feather coloration remain unanswered. Long-read sequencing technology, also known as third-generation sequencing, is capable of generating reads exceeding 10 kb in length. This capability allows it to span highly repetitive genomic regions and resolve assembly gaps previously intractable, thereby enhancing the continuity of genomic assemblies. As an alternative to relying on short-read data polishing, PacBio introduces high-fidelity reads (HiFi reads) which can provide more accurate, continuous, and complete genetic information. It has thus become a key technology for research [13]. Meanwhile, advancements in gene chips and genome re-sequencing technologies have made genome-wide association studies (GWAS) powerful tools for identifying genetic variations linked to phenotypes. GWAS analysis has uncovered mutations in the *MuPKS* gene responsible for yellow and blue plumage in parrots [14]. Additionally, GWAS has localized a nonsense mutation (W49X) in the *SLC2A11B* gene, which is associated with the white eye trait in pigeons [15]. To this end, the presence of the *PMEL* gene,

which was previously considered "missing" gene in ducks, was hereby explored based on a high-quality *de novo* genome by HiFi sequencing. Building on previous findings, this research identified two closely linked SNPs in the regulatory region that might influence *PMEL* transcription, leading to the white plumage observed in LC ducks. Overall, this investigation provides a valuable genome assembly, molecular markers for duck breeding, and offers insights into plumage color patterns in avian species.

Data Description

To understand the genetic basis of the unique plumage phenotype of Liancheng ducks, a high-quality *de novo* assembly of the Liancheng duck genome (GCA_039998735.1) was hereby utilized, and four different plumage color phenotypes were collected from a crossbreeding population involving Liancheng and PK ducks. Meanwhile, whole-genome resequencing of 366 ducks was performed, and the resequencing data were aligned to our assembled Liancheng duck reference genome for variation calling and subsequently GWAS analysis. In the present study, the *PMEL* gene associated with plumage coloration in Liancheng ducks was successfully identified through transcriptome sequencing analysis across black-, and white-feathered ducks and nine duck tissues, and the genetic mechanisms underlying the formation of duck plumage coloration were evidently elucidated.

Materials and Methods

Ducks and sampling

Herein, all animal procedures adhered to the guidelines for the care and use of experimental animals set by the Chinese Academy of Agricultural Sciences (IAS2022-105). The study was approved by

the Ethics Committee of the Chinese Academy of Agricultural Sciences. A blood sample was obtained from a female Liancheng duck for the purpose of conducting a *de novo* genome assembly. A total of 366 parental and intercross population duck plumage color phenotypes were recorded from a previous gradient consanguinity population [6]. These included 117 PK ducks, 59 LC ducks, 38 white-feathered ducks with yellow beaks (WY), 42 white-feathered ducks with black beaks (WB), 67 gray-feathered ducks with black beaks (GF), and 41 black-feathered ducks with black beaks (BF). All underwent whole-genome resequencing (Supplementary Table S1). Additionally, genome data from 23 black-feathered ducks, comprising 20 Mallards (MD) and 3 Putian (PT) ducks, were used for comparative analysis [2].

For transcriptomics analysis, skin tissues from LC ducks were collected at embryonic stages of 12 days, 15 days, 20 days, 28 days, and one week after birth. Each sample group, except for the four 12-day embryo samples, consisted of three biological replicates. Additionally, tissues from an 8-week-old black-feathered Mallard (MD) duck, including heart, fat, muscle, brain, spleen, lung, liver, kidney, and skin, were collected, with one replicate per tissue (Supplementary Table S2) [2]. Feather bulb specimens were also collected from feather follicle from 1-week-old black-feathered MD ducks, LC ducks, PK ducks, and WB ducks, with three biological replicates in each sample group [16,17]. A total of 37 samples were used for RNA-seq (Supplementary Table S2). For the immunofluorescence assay, skins containing hair follicles from three 1-week-old MD ducks and three LC ducks were collected for protein analysis.

Genome assembly and gene annotation

A combination of PacBio long-read HiFi sequencing and chromosome interaction mapping (Hi-C) technologies was hereby utilized to conduct the *de novo* assembly of the Liancheng duck genome. Long-read and long-HiFi (RRID:SCR_021966) sequencing data (PacBio, Beijing, China) were used for the species assembly [18]. Sequencing was performed on the PacBio Sequel II platform, and genome assembly was conducted using the hifiasm versions (v0.19.3 NOV-2023) for Contig generation. The 3D-DNA pipeline (RRID:SCR_017227) (NOV-2023) was utilized for manual curation and orient the genome sequences based on Hi-C contact map [19,20,21]. Ultimately, the BUSCO was used to evaluate the completeness and quality of Liancheng duck genome assembly (IASCAAS_LianchengWhiteDuck, GCA_039998735.1) [22].

Protein-coding genes were predicted via the combination of evidence-based prediction and *de novo* prediction. RNA-seq data were used for evidence-based annotation through Maker2 (Version 2.31.10), i.e., a powerful open-source genome annotation tool. RNA-seq data were aligned against the genome using PASA (Program to Assemble Spliced Alignments) (RRID:SCR_014656) to construct a training model for *Augustus* (RRID:SCR_008417), a tool designed to predict genes in eukaryotic genomic sequences. Subsequently, *de novo* annotation of the genome was performed utilizing *Augustus*. *De novo* genome assembly refers to a method of piecing together a complete sequence of a new genome, rather than comparing it based on a known genome. Subsequently, the results of the RNA-seq based annotated and the *de novo* based annotation were integrated on the principle that the evidence results were better than those prediction ones. In the final step, genes were filtered to retain those with less than 50% repeated sequences, encoding proteins longer than 50 amino acids, and having at least one count of expression. Functional annotation of genes was

performed using eggNOG software (RRID:SCR_002456) [23].

Whole-genome resequencing

A total of 366 samples, consisting of Pekin ducks, Liancheng ducks, and intercross populations, were hereby selected for resequencing (Supplementary Table S1). The genome data of 23 black-feathered ducks included 20 Mallards, and 3 Putian ducks. DNA-eligible samples were identified for further testing. Libraries were established, involving an average read length of 150 bp for all samples. Subsequently, they were sequenced on an Illumina HiSeq X-Ten platform, and an average raw read sequence coverage of 5x was yielded. This 5x coverage ensured the accuracy of variant calling and genotyping when linking back to the Liancheng duck reference genome (IASCAAS_LianchengWhiteDuck, GCA_039998735.1) [6]. Following the elimination of read pairs containing adapter sequences, a quality assessment of the raw reads was performed using TRIMMOMATIC (RRID:SCR_011848) (version 0.36) and Cutadapt [24,25]. Following that, the high-quality reads were aligned to the Liancheng duck reference genome using the Burrow–Wheeler Aligner (BWA-aln) with parameter 'bwa aln genome.fa sample.fastq > sample.sai, bwa sampe genome.fa sample.sai sample.fastq > sample.sam' [26]. Besides, genetic variants were identified from the sequencing data in this study using Genome Analysis Toolkit (GATK) [27]. SNPs underwent filtration based on the following criteria: (i) SNPs with minor allele frequency (MAF) >0.05; (ii) the maximum missing rate per SNP set at <0.7; and (iii) SNPs restricted to possessing only two alleles.

Genome-wide association analysis

The GWAS was conducted utilizing a mixed linear model implemented through the EMMAX program (RRID:SCR_024012) [28] with genome-wide SNP data and the plumage color phenotype observed in 366 individuals from the resequencing population. The analytical model adopted the form y = Xb + Ga + e, with y representing the phenotypic value (plumage color of each duck), X denoting the matrix corresponding to fixed effects, and b signifying the magnitude of the fixed effects. The fixed effects included sex-related influences. G represents the genetic matrix associated with population kinship, while e stands for the random residual. Furthermore, principal component analysis (PCA) was executed using all SNPs, with the top three components incorporated as fixed effects within the mixed model to adjust for population stratification. A Bonferroni correction threshold of 0.01/N ($-\log_{10}P = 8.95$) was established to pinpoint significant sites in the GWAS findings [2,8,29], where N indicates the total number of whole-genome SNPs (8,887,194). In addition, fine-mapped analyses were conducted in 328 ducks via IBD analysis, for the efficiency of IBD fragments in reflecting the genetic relationship between individuals and detecting trait variation. The correlation between IBD fragments and phenotype was used to identify regions affecting trait variation in the genome [28,30]. For this analysis, the filtered SNPs (n= 117) met the standard allele frequency difference (ΔAF) >0.8 between the Liancheng ducks and Pekin ducks. In the candidate region (Chr33:5.1-5.5Mb), four recombination breakpoints were identified across the 36 SNPs, and the segregating individuals were subsequently classified using these four recombinant breakpoints.

Transcriptome sequencing and analysis

Skin tissues from LC ducks were collected at embryonic stages of 12 days, 15 days, 20 days, 28 days, and one week after birth. Additionally, tissues including heart, fat, muscle, brain, spleen, lung,

liver, kidney, and skin were collected from an 8-week-old black-feathered MD duck. Feather bulb specimens were also obtained from feather follicles of 1-week-old black-feathered MD ducks, LC ducks, PK ducks, and WB ducks. The total RNA was initially extracted from the above tissues using Trizol reagent (Vazyme, Nanjing, China). The RNase enzyme was inactivated by adding pyrrole diethyl carbonate. A total of 12 final RNA-seq libraries were prepared for the experiment and sequenced on an Illumina platform using the 150-bp paired-end sequencing module. The effective read length was increased by Illumina sequencing, yielding an average production of 6 Gb per library. Using TopHat (RRID:SCR_013035), RNA-seq paired-end reads from each library were mapped to the aforementioned reference genome of the Liancheng duck. Besides, the expression was calculated by using TopHat, and read counts per million (CPM) values for the genes were obtained running htseq-count in figshare database (10.6084/m9.figshare.27311937) [6,31].

qPCR analysis on *PMEL* in feather bulb specimens

cDNA from feather bulb specimens, including those of black-feathered, grey-feathered, Liancheng, and Pekin ducks, was reversely transcribed using HiScript III All-in-one RT SuperMix Perfect for qPCR (Vazyme). The quantitative PCR (qPCR) was conducted in a total volume of 10 μl, which included 5 μl of Tap Pro Universal SYBR qPCR Master Mix (Vazyme), 0.8 μl of forward and reverse primers, 0.5μl of cDNA, and 3.7 μl of distilled water. β-actin was selected as the internal reference gene. The primer sequence is shown in Supplementary Table S3. All reactions were run in triplicate. The relative mRNA expression levels were calculated using the normalized relative quantification method, followed by the $2^{-\Delta\Delta CT}$ calculation [32].

Immunofluorescence experiment

The skin samples of ducks with black and white feathers were embedded in paraffin, fixed in 4% buffered paraformaldehyde, and sectioned into 5 μm slices. Upon overnight fixation at 4°C, the duration was kept within 24 hours to ensure effective preservation of tissue integrity. Subsequently, the sections were dewaxed and rehydrated to enhance adhesion and facilitate the dewaxing process. Antigen retrieval was performed by incubating the sections in EDTA (Servicebio) at 100°C for 20 minutes. Following retrieval, antigens were fixed with Tris-EDTA, and sections were washed thrice in phosphate-buffered saline (PBS), followed by a further rinse. For immunostaining, sections were incubated with a PMEL antibody (ABclonal) at 4°C for 12 hours post pre-treatment with 3% bovine serum albumin (Solarbio) for 30 minutes. The PMEL antibody, an anti-rabbit species, was hereby utilized for immunodetection. Ultimately, feather follicle tissues displaying diverse plumage colors were counterstained with DAPI.

Causative mutation screening and identification

The candidate regions (Chr33:5.24-5.32Mb) among 117 Pekin ducks, 59 Liancheng ducks, and 152 intercross population ducks (excepting 38 WY ducks) were compared based on the Liancheng duck genome. Among the candidate IBD fragments, only regions featuring consistent genotypes and phenotypes were further investigated as *Rr* candidate regions. To eliminate variations with a lower likelihood of being causally involved, the following three-step procedures were conducted: Firstly, the genotype and phenotype information from the 328 parents and intercross ducks was utilized to exclude SNPs based on the standard $F_{ST} < 0.8$ (LC vs PK duck). Secondly, information of 117 Pekin ducks, 59 Liancheng ducks, 20 Mallards, 42 WB ducks, 43 BF ducks, and 3 Putian ducks was

applied. Candidate regions were selected based on the highest F_{ST} values shared between Liancheng ducks and other breeds. Thirdly, only genotypes that were completely concordant with the phenotypes from the Mallard and Putian duck populations were retained as candidate causative mutations. Subsequently, all indels within the candidate regions were excluded using the method described above. Ultimately, only two SNP variations that showed genotype-phenotype concordance across multiple duck breeds were considered causative variations for the *Rr* locus.

Hi-C sequencing and analysis

Skin fat tissue samples from a Liancheng duck were subjected to cross-linking in 20 ml of fresh ice-cold nuclear isolation buffer. The chromatin extraction methodology adhered to previous protocols [2]. Subsequently, the purified DNA underwent digestion and fragmentation using the *DpnII* restriction enzyme, followed by a repair of the DNA ends, and biotin-labeled DNA fragments were then isolated using streptavidin C1 beads. Afterwards, library preparation was conducted utilizing an Illumina TruSeq DNA Sample Prep Kit following the manufacturer's guidelines. Quality assessment of the Hi-C library was performed through TA cloning, and the Hi-C libraries were sequenced on an Illumina HiSeq X Ten system. The Hi-C experiments were performed independently on two occasions, with the experimental and sequencing procedures executed by Gene Technology Co., Ltd., located in Beijing, China.

Raw Hi-C data were processed to eliminate low-quality reads and trim adapters using TRIMMOMATIC (RRID:SCR_011848) [24]. All reads were trimmed to 150 bp, and clean reads were aligned to the duck genome using a two-step approach integrated into the HiC-Pro

(RRID:SCR_017643) software [33]. Reads of low mapping quality, multiple mappings, and singletons were excluded. Subsequently, uniquely mapped reads were consolidated into a single file. Read pairs were filtered out if they did not align near a restriction site or did not meet the expected fragment size post-shearing. Subsequent filtering steps were applied to eliminate read pairs derived from invalid ligation products, such as dangling-end and self-ligation products, as well as PCR artifacts. The remaining valid read pairs were categorized into intrachromosomal and interchromosomal pairs, and contact maps were then generated using chromosome bins of uniform sizes ranging from 3 kb to 1 Mb. The initial contact maps were normalized using a sparse-based iteration correction method within HiC-Pro and visualized utilizing HiCPlotter [34]. Finally, regions resembling topologically associated domains (TADs) and boundaries were delineated using the default algorithm within HiCPlotter at a resolution of 5 kb [34].

Structural variation detection

In the GWAS candidate region, all CNV structural variations in the selected populations were analyzed, including Pekin ducks, Liancheng ducks, WB ducks, GF ducks, and BF ducks. Ducks from these different feather groups were randomly selected, and the genotype of all individual CNVs was investigated using CNVcaller (RRID:SCR_015752) software (version 0.11) [35]. The CNV calling and genotyping procedures were consistent with those previously described [36]. Log_2 fold change values reflected the ratio of sequencing read depths in the 1,000bp window region to that of Pekin duck reads. Therefore, the distribution of all CNV genotypes in the aforementioned populations was examined. Copy numbers of 1, 0.5, 0, 1.5, and 2 corresponded to normal diploidy, loss of heterozygosity, homozygous loss, heterozygous duplication, and homozygous duplication,

respectively. Absolute copy numbers above 2 suggested the presence of complex duplications [36].

Luciferase reporter assay

Herein, a total of four haplotypes of candidate variations SNP1 and SNP2, along with their upstream and downstream regions, were cloned into the pGL3-basic and pGL3-promoter vector. The *XhoI* and *KpnI* restriction sites were utilized as insertion points in the pGL3-basic vector for promoter activity analysis, whereas the *BamHI* and *SalI* sites were chosen in the pGL3-promoter vector for enhancer activity assessment. A375 and DEF cells were plated in 48-well plates at a density of 0.5×10^5 per well and cultured for 24 hours in DMEM (Pricella, China) mixed with 10% FBS (Pricella, China). Subsequently, the cells were transfected using Lipofectamine 8000 (Beyotime), with each well receiving an equal amount of DNA (237.5 ng), encompassing the four sequences that included the SNP1 and SNP2 sites. Meanwhile, 12.5ng of pRL-TK vector was added to each well. Following the manufacturer's protocol, cell lysates were harvested post-lysis, and luciferase activity was measured using the Veritas Microplate Luminometer (Promega). Each sample was assayed in triplicate, with Renilla luciferase activity employed to normalize the firefly luciferase readings [12, 37].

Analyses

A newly assembled high-quality Liancheng duck genome

To facilitate a comprehensive analysis of the white plumage Liancheng duck, a *de novo* genome was constructed utilizing HiFi long-read sequencing data, achieving an 88x genome coverage, and 584.72 Gb of Hi-C data were generated (Supplementary Table S4). These datasets were then utilized

for *de novo* assembly for Liancheng duck genome with contig N50 of 12.17 Mb. The contigs were further scaffolded, corrected and ordered based on Hi-C contact map with scaffold N50 of 83.98 Mb (IASCAAS_LianchengWhiteDuck, GCA_039998735.1) (Fig. 1A Supplementary Table S5). Finally, our *de novo* assembled 1.29 Gb Liancheng duck genome exhibited perfect collinearity with Mallards (GCA_008746955.3) and the Pekin duck reference genome (GCA_015476345.1) demonstrated the high quality of our genome assembly (Fig. 1B). The length of scaffold N50 in our Liancheng duck genome assembly was the longest among all previously published duck (*Anas platyrhynchos*) genomes, indicating high continuity of our assembly (Fig. 1C, 1D, Supplementary Table S6). Benchmarking Universal Single-Copy Orthologs analysis (BUSCO, RRID:SCR_015008) [8] revealed 96.7% universal single-copy orthologues, suggesting high completeness of our genome assembly (Supplementary Table S7). Based on the high-quality *de novo* assembled of our Liancheng duck genome, a total of 18,819 genes were predicted (Supplementary Table S8).

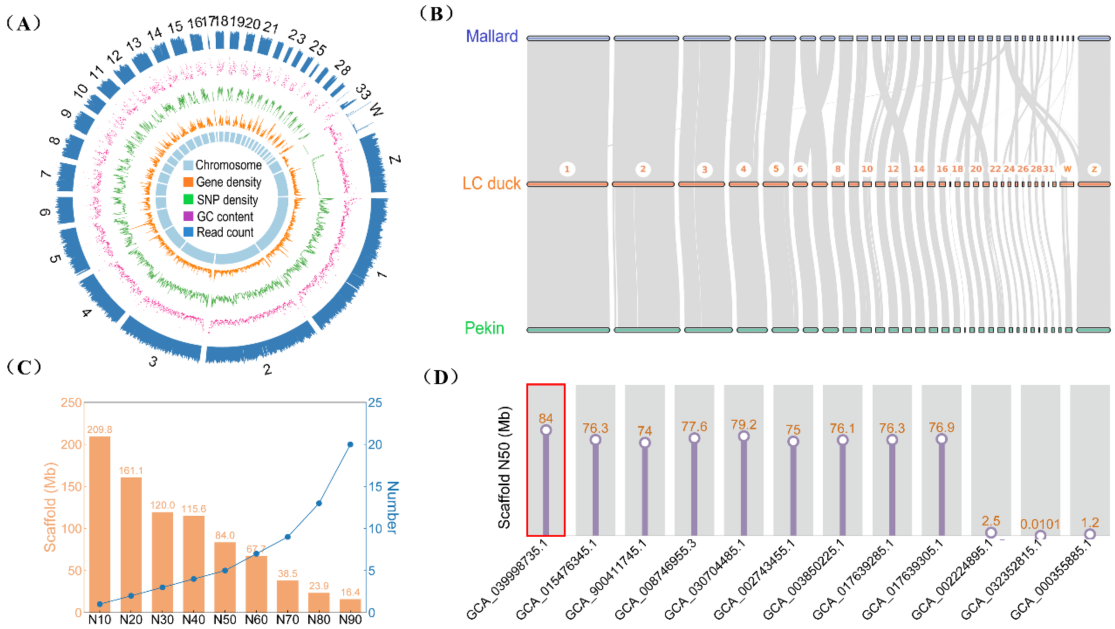

Figure 1. Overview of the assembly quality and characteristics of the Liancheng duck genome. (A)

Circular diagram illustrating the characteristics of the genome assembly. The tracks from the inner to outer circles represent the following: chromosomes, gene density, SNP density, GC content (%), and read count. The window size of each circle was 200 kb. (B) Genome synteny analysis between the Liancheng duck and Mallard, as well as Pekin duck. Chromosomes 1-33, as well as the two sex chromosomes. (C) Genome statistics for the hifiasm genome assemblies of the Liancheng duck genome in this study. (D) The length of scaffold N50 (Mb) of Liancheng duck in this study (GCA_039998735.1) was compared with all previously published duck (*Anas platyrhynchos*) genomes.

Inheritance of F2 population traits conforming to the law of independent assortment

A crossbreeding study involving 30 Pekin and 120 Liancheng ducks was conducted. All F1 individuals (1,260) displayed a grey plumage color and pattern. In the F2 populations, four phenotypes were observed, namely BF, GF, WB, and WY ducks (Fig. 2). The ratio of BF: GF: WB: WY ducks in the F2 population was 235:452:234:360, which closely approximated the theoretical ratio of 3:6:3:4 (Table 1 and Supplementary Fig. S1). The phenotypic ratio observed followed Mendel's law of independent assortment for two genes. It was hypothesized that the genetic mechanism controlling plumage color in Liancheng ducks was governed by two sites (*Bb* and *Rr* sites), with the allele at the *Rr* site, in interaction with the *Bb* site, determining white plumage in Liancheng ducks (Table 1). Within the F2 population, two alleles (*B*, dominant, enabling melanin synthesis, and *b*, recessive, inhibiting melanin synthesis) segregated at the *Bb* locus. The other locus, denoted as *Rr*, possessed two alleles that regulated melanin accumulation in the feather: *R* (dominant, allowing melanin synthesis in the feather) and *r* (recessive, repressing melanin synthesis). The *B*

allele at the *Bb* locus displayed an epistatic effect, while the *R* allele at the *Rr* locus demonstrated an incomplete dominance effect. The cross between the Liancheng duck (*BBrr*) and the Pekin duck (*bbRR*) resulted in the production of grey feather ducks (*BbRr*), with the genotypes of BF, GF, WB, and WY being *B_RR*, *B_Rr*, *B_rr*, and *bb_ _*, respectively (Table 1, Fig. 2). Importantly, no significant difference was observed between the actual and expected numbers within the F2 population (n=1,281, p=0.345), with a squared value of 3.322 ($\chi2_{0.05(3)}$ =7.81, $\chi2_{0.01(3)}$ =11.34). The *Bb* locus had previously been identified as the primary gene responsible for white plumage in ducks [2].

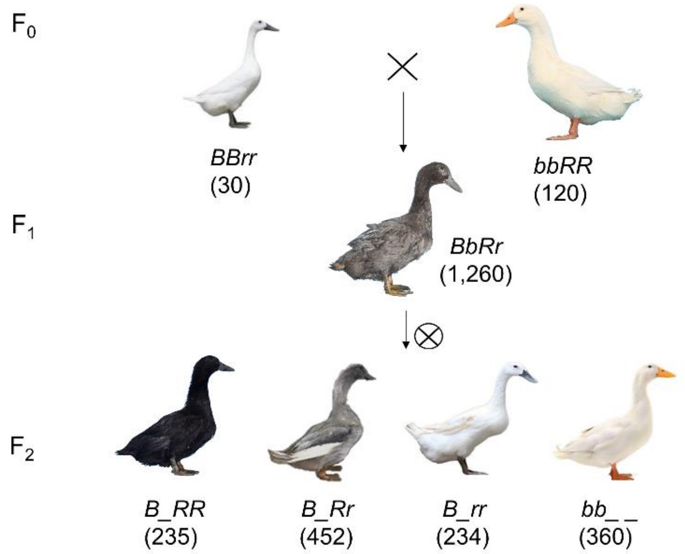

Figure 2. The diagram depicted the segregation of plumage colors in the F2 population. The Liancheng duck showed the white-feathered ducks with black beaks (WB), whereas the Pekin duck exhibited the white-feathered ducks with yellow beaks (WY). The F1 generation displayed the gray-feather ducks with black beaks (GF). In the subsequent F2 generations, ducks were observed with phenotypes including black-feathered ducks with black beaks (BF), GF, WB, and WY ducks.

Table 1 The number of F2 populations in different phenotypes and the Chi-squared test.

Comparison	BF duck (*B_RR*)	GF duck (*B_Rr*)	WB duck (*B_rr*)	WY duck (*bb_ _*)	Ratios	χ^2 value	*P*-value
Observed number	235	452	234	360	3:5.8:3:4.6	3.322	0.345 (ns)
Expected number	240	481	240	320	3:6:3:4		

χ 2 0 . 0 5 (3) = 7 . 8 1 ,

Genome-wide association analysis for segregating population duck plumage color

The duck samples were re-sequenced with the average depth of 5x. A cohort of 188 ducks from a segregating population derived from Liancheng ducks and Pekin ducks was hereby selected for GWAS analysis. Initially, using the genome of the Liancheng duck as a reference, the present study identified two sites controlling the white feather phenotype on chromosomes 13 and 33 (Fig. 3A). This research highlighted a specific gene on chromosome 13 that regulated melanin synthesis in Liancheng ducks (Supplementary Fig. S2, S3). Given that the white plumage phenotype in Liancheng ducks did not show sex-related patterns, cytoplasmic inheritance considerations for white plumage were deemed unnecessary. Subsequently, the *Rr* gene was pinpointed to the 5.24-5.32Mb region of chromosome 33 in Liancheng ducks (Fig. 3B), which contained potential candidate genes such as *PMEL*, *RAB5B*, *IKZF4*, *ERBB3*, *PA2G4*, *ZC3H10*, and *ESYT1* (Fig. 3C). Within the candidate region (Chr33: 5.24-5.32 Mb), four minimal recombination haplotypes were identified based on the parents and segregating populations from 117 SNPs with an F_{ST} >0.8 (PK vs LC ducks). Only the haplotypes in block 4 (Chr33: 5,303,111-5,304,416, 101,305 bp) located upstream of the *PMEL* gene corresponded to the observed phenotypes (Fig. 3D). Additionally, a significantly high peak in the F_{ST} value was observed in the *RR* vs *rr* duck populations within the selected candidate

χ 2 0

region, while no peak was spotted in *rr* vs *rr* duck populations (Fig. 3E).

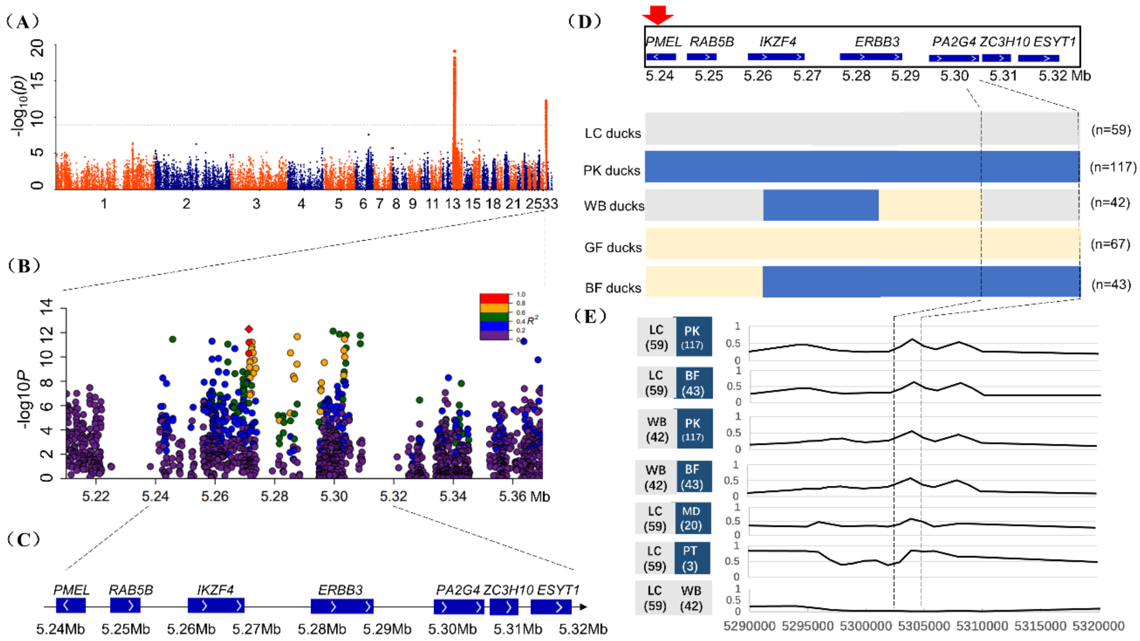

Figure 3. Screening for the candidate region associated with the white plumage of Liancheng ducks involved a GWAS on a cohort of 188 ducks from a cross between Pekin ducks and Liancheng ducks. (A) A Manhattan plot showed the genetic effects on plumage color. (B) Zoom in the regions on chromosome 33 (5.21-5.37 Mb) linked to white plumage in Liancheng ducks. Genotypic SNPs were identified based on linkage imbalance values compared to the leading SNP in the intercross population duck (Chr33: 5,303,413). (C) Candidate genes in the region (5.24-5.32 Mb) were identified, with white and black arrows indicating gene orientation and chromosome 33 direction, respectively. (D) Identity by Descent (IBD) analysis used color schemes to refine candidate regions, with blue for Pekin ducks and black-feathered ducks, grey for Liancheng ducks and white-feathered black beak ducks, and yellow for grey plumage ducks (LC vs PK, F_{ST}> 0.8). (E) Genome divergence analysis among six duck breeds, including LC vs PK ducks, BF ducks, Mallards (MD), and Putian (PT) ducks within the candidate region (Chr33: 5.29-5.32 Mb), averaged F_{ST} values in 10kb region

in each comparison group.

PMEL causes melanin deposition in duck plumages

The region on chromosome 13 was found to encompass the *MITF* gene in the GWAS analysis (Supplementary Fig. S2). Comparison with the Pekin duck genome assembly (GCA_003850225.1) revealed a 6.6 kb insertion in the *MITF* gene, strongly associated with melanin synthesis in ducks (Supplementary Table S9). Following the exclusion of the MITF signal, subsequent GWAS analysis identified a single signal on Chromosome 33 (Supplementary Fig. S3). Candidate region included *PMEL*, *RAB5B*, *IKZF4*, *ERBB3*, *PA2G4*, *ZC3H10*, and *ESYT1*. Results indicated that only the *PMEL* gene showed significant differential expression (Fig. 4A, 4B), with higher expression levels in black-feathered ducks compared to grey-feathered ducks (-Log10(p)>30). RNA-seq results showed no expression of the *PMEL* gene in feather bulb specimens of white-feathered ducks (Fig. 4C, 4D). Other genes within the GWAS candidate range (Chr33: 5.24-5.32Mb) were excluded due to similar gene expression levels in different plumage populations or inconsistent gene expression patterns related to melanin regulation. Furthermore, qPCR results confirmed *PMEL* as the *Rr* gene (Fig. 4C). Notably, the *PMEL* gene exhibited an elevated average GC content of 72.4% (Supplementary Fig. S4). Additionally, the *PMEL* gene expression correlated with the plumage color phenotype of Liancheng ducks at various developmental stages, with high expression levels observed in the skin tissue (Fig. 4B), further underscoring the importance of establishing a high-quality genome for Liancheng ducks.

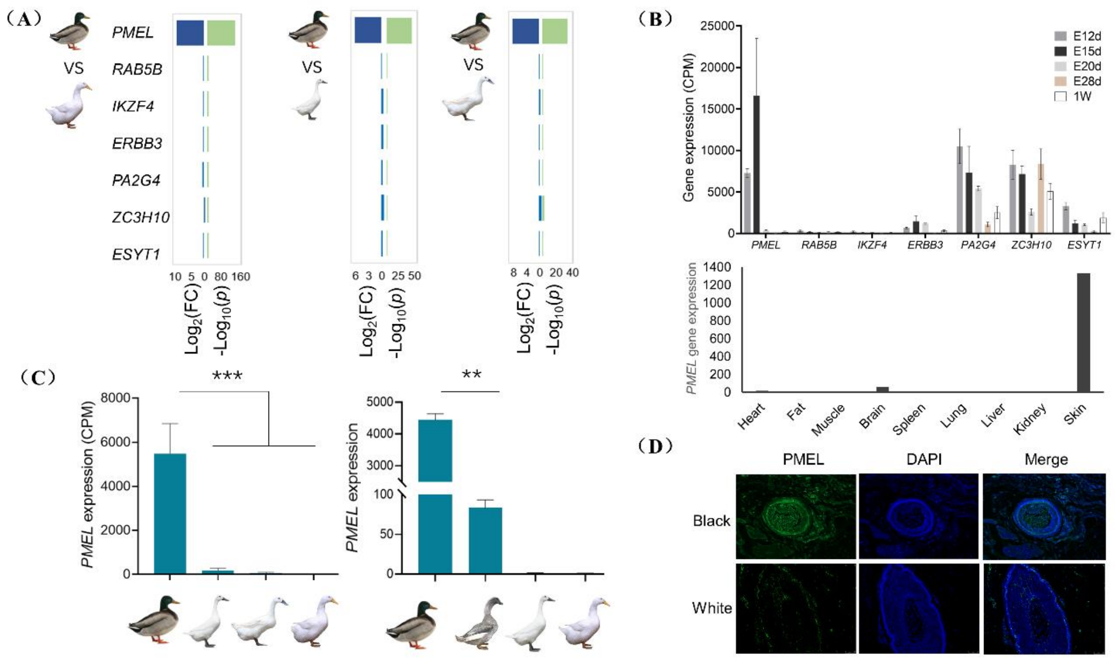

Figure 4. Identification of the candidate gene for white plumage in Liancheng ducks (A) Gene expression of seven GWAS candidate region genes (*PMEL*, *RAB5B*, *IKZF4*, *ERBB3*, *PA2G4*, *ZC3H10*, *ESYT1*) in 1-week-old feather follicle samples of white and black-feathered ducks, with three replicates per sample. $Log_2(FC)$ values were used to analyze gene expression differences in blue, and values where $-Log_{10}(p) > 30$ were shown in green. (B) Analysis of the expression levels of the seven candidate genes in skin tissues of Liancheng ducks at different developmental stages. E12d, E15d, E20d, and E28d (also the first day of birth) represent 12, 15, 20, and 28 days of the embryonic period, respectively. Data were shown as mean ± standard error (n=3). (C) CPM and qPCR results of *PMEL* in 1-week-old feather bulb samples of ducks. Data were presented as mean ± standard error (n=3). ** $p<0.01$; *** $p<0.001$. (D) Immunofluorescence results showing PMEL distribution in feather bulb specimens of black- and white-feathered ducks. Black (Mallards) and White (Liancheng ducks).

***Rr* variation being fine mapped to *PMEL* upstream regulatory region**

The results of the IBD analysis indicated that only the haplotypes in block 4 (Chr33: 5,303,111-5,304,416, 101,305 bp), positioned upstream of the *PMEL* gene, were associated with the observed phenotypes (Fig. 3D). Moreover, a pronounced peak was observed in the *RR* vs *rr* duck populations within the candidate region (Fig. 3E), further substantiating this segment (Chr33: 5,303,111-5,304,416, 101,305 bp) as the *Rr* locus. Among the identified candidate variations in this region, one copy number variation (CNV) (Supplementary Fig. S5 and S6) was initially excluded. Only 12 SNP variants and two Indels were retained after applying a threshold of $F_{ST} > 0.8$ (PK vs LC ducks) (Supplementary Table S10). It should be noted that all 12 SNP were found in the upstream regulatory region of the *PMEL* gene (Chr33: 5,239,969-5,244,318). Finally, two SNPs (Chr33: 5,303,994A>G; 5,303,997A>G) were hereby identified as the potential causal variants across all duck breeds (Supplementary Table S10). Intriguingly, these two SNPs were observed to be in complete linkage disequilibrium. Additionally, Hi-C data revealed that the PMEL gene, along with its upstream region harboring the two candidate SNPs, resided within a topologically associating domain (TAD) (Fig. 5).

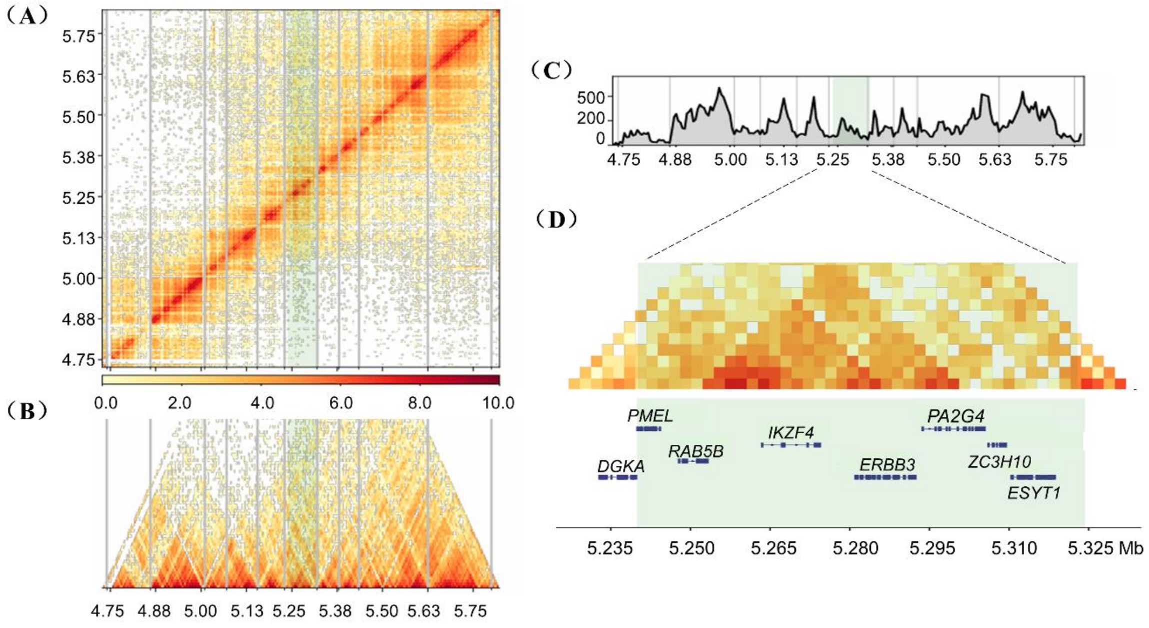

Figure 5. Chromosome interaction mapping (Hi-C) result of the end of chromosome 33 (4.75-

5.8Mb) in skin fat of Liancheng ducks. (A) Log_2(interaction matrix) analysis of Chr33 (resolution:5kb). Strong contacts are shown in red, and weak contacts are shown in white. The heat map shows a normalized contact matrix in 5kb bins. Light green indicates the *Rr* site candidate region by GWAS analysis. (B) Triangular result of Log_2 (interaction matrix) of Chr33 (4.75-5.75Mb) and the (C) topologically associated domain (TAD)-like and boundary-like regions were identified with the default algorithm built in HiCPlotter at a resolution of 5kb. (D) Log_2 (interaction matrix) of Chr33 (5.235-5.325Mb). Candidate regions identified from Chr33:5.24-5.32Mb by GWAS result and genes within it (light green regions).

Functional analysis of two candidate SNPs

Considering the location of the two candidate SNPs (SNP1 and SNP2) (Chr33: 5,303,994A>G; 5,303,997A>G) in the noncoding upstream region of the *PMEL* gene, their promoter and enhancer effects were assessed using pGL3 luciferase vectors (Fig. 6A, 6B). Both pGL3 vectors (pGL3-basic-white and pGL3-basic-black) with inserts showed minimal luciferase activity in duck embryo fibroblast (DEF) cells and human melanoma cells (A375) (Fig. 6C), indicating no promoter activity at these SNP sites. However, for enhancer activity, those vectors with inserts (pGL3-promoter-white and pGL3-promoter-black) displayed significantly different luciferase activity in DEF cells and A375 cells (ANOVA, $p<0.01$). Notably, SNP2 (Chr33: 5,303,997A>G) revealed the higher luciferase activity of both pGL3-promoter-white-1mut and pGL3-promoter-white-2mut compared with pGL3-promoter-white (Fig. 6D). This indicated a synergistic enhancement activity by the black alleles of variations SNP1 and SNP2.

Analysis on the JASPAR transcription prediction website revealed that multiple transcription factors might bind differently to sequences surrounding candidate SNPs located at Chr33: 5,303,944-5,304,098. The results suggested that variations in SNP1 and SNP2 could impact the binding of various transcription factors, as illustrated in Supplementary Fig. S7. Furthermore, differential expression of the SOX5 transcription factor was observed in the feather follicles of white- and black-feathered ducks. It was thereby hypothesized through these findings that variations in SNP1 and SNP2 could potentially be key mutations responsible for the white feather phenotype.

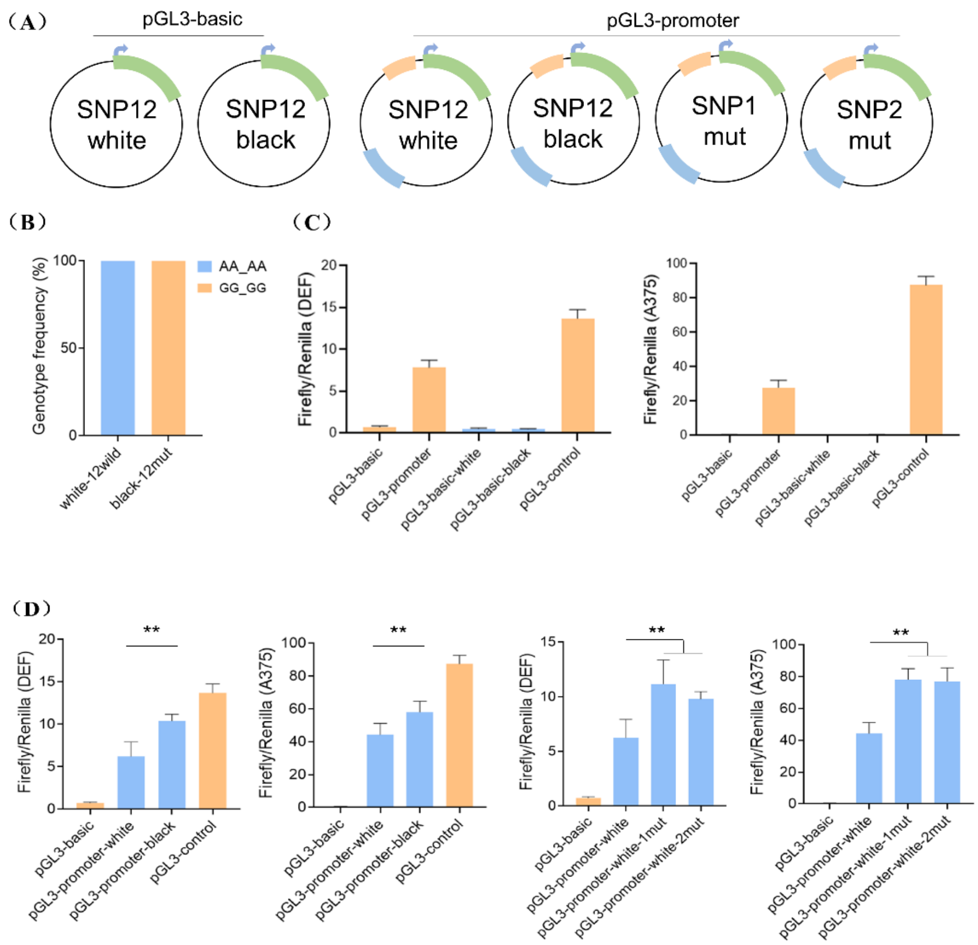

Figure 6. Functional analysis of the candidate variation controlling the white plumage phenotype of Liancheng duck. (A) Diagram of six pGL3 vectors for the luciferase reporter gene experiment. Candidate SNP1 and SNP2 (Chr33:5,303,994A>G and 5,303,997A>G) of Liancheng duck and

Pekin duck were inserted into empty pGL3-basic and pGL3- promoter vectors. pGL3-basic, pGL3-promoter and pGL3-control were used as negative and positive control. Insertion fragments are marked in yellow to verify the promoter activity. The blue box represents insertion fragment of the PGL3-promoter to verify enhancer activity. Additionally, vectors also contained a green box (luciferase reporter gene) and a blue arrow (indicate transcription site). (B) Genotype distribution of SNP1, SNP2 in Liancheng duck (AA_AA, n=59) and Pekin duck (GG_GG, n=117). (C) Validation of promoter activity of homozygous wildtype (AA_AA) and homozygous mutation (GG_GG) in DEF cells and A375 cells. Data are presented as mean ± standard error (n=8), (ANOVA, p>0.05). (D) Enhancer activity of AA_AA, GG_GG, GG_AA, and AA_GG vectors in DEF cells and A375 cells. Data are presented as mean ± standard error (n=8), (ANOVA, ** p<0.01).

Discussion

The white plumage phenotype is considered a common trait in various avian species, such as chickens [38, 39], peafowl [40], geese [41], and ducks [2, 42]. The unique white plumage phenotype of Liancheng ducks has been extensively delved into [42, 43, 44]. however, the genetic mechanisms behind remain unclear. The present findings suggested that the inheritance of the plumage color phenotype in Liancheng ducks was likely governed by two autosomal genes, independent of the sex chromosomes (Table 1). Despite using the previous Pekin duck reference genome (IASCAAS_PekingDuck_PBH1.5, GCA_003850225.1), three signals were identified through the current GWAS analysis (Supplementary Fig. S8), which was considered to be attributed to an incomplete assembly. To further investigate the white feather phenotype of Liancheng ducks, a high-quality genome was therefore first established for Liancheng ducks (Fig. 1). This new reference

genome featured a size of 1.29 Gb, with contig and scaffold N50 values of 12.17 and 83.98Mb, respectively (Supplementary Table S5). The scaffold N50 length of the Liancheng duck genome was higher than that of other duck genomes [45, 46, 47, 48], representing a more complete and better continuity of the duck genome assembly. However, the chromosomes number identified from this newly genome should still be further improved compared to those of the Muscovy duck and Crested Duck [49, 50]. Further pan-genome and functional gene-mining analysis could be conducted in the future [29, 47, 51]. Taken together, these findings represent the first construction of the Liancheng duck genome, resulting in enhanced genome contiguity compared to previous duck genomes.

MITF is a key regulator of melanin synthesis, controlling the expression of enzymes involved in this process as well as receptors critical to melanocyte function [52, 53, 54]. This gene produces multiple isoforms through the use of alternative promoters, which share coding exons but feature different amino termini [55]. While *MITF* variants are known to influence melanin regulation, the regulation of these isoforms remains to be explored. Herein, the expression of two *MITF* isoforms, *MITF-B* and *MITF-M*, were discovered in ducks, with only the latter being crucial for melanin synthesis in duck plumage [2, 9, 56]. *MITF-M* isoforms have also been shown to regulate white coloration in the fur of dogs [57], llamas [58], and mice [59]. SNPs, indels, and structural variants were previously found in *MITF* as possible causes of white plumage in ducks [60]. Two synonymous SNPs (c.114T>G and c.147T>C) and a 14-bp indel (GCTGCAAACAGATG) in intron 7 of duck *MITF* were significantly associated with the black- and white-colored breeds (p<0.001) [61]. One variant in the 5’UTR of *MITF* was significantly associated with feather color phenotypes in geese

[62]. A 6.6 kb insertion within the *MITF* gene demonstrated a strong correlation with melanin production in ducks [2], and indicated the on-off role of *MITF* in the melanin generation pathway of Pekin ducks. *MITF* could promote differentiation-related functions, including regulation of genes involved in pigmentation, such as *PMEL*, *TYR*, *TYRP1*, *DCT*, *MLANA*, *SILV*, and *SLC24A5* [60]. In the years following the separation of the *MITF* gene, the number of potential target genes increased sharply. Based on GWAS analysis, the role of the *MITF* gene as an epistatic gene controlling melanin synthesis in Liancheng ducks was hereby confirmed, aligning with previous research findings [44]. Overall, this highlights the significant regulatory role of *MITF* in melanin synthesis in Liancheng ducks and underscores its importance as a key genetic factor in pigmentation.

PMEL, a type I transmembrane transport glycoprotein, is synthesized in the endoplasmic reticulum and plays a crucial role in amyloid fiber formation during stages I and II of melanosome formation in the L-DOPA pathway [63, 64]. Upon synthesis, PMEL is transported to the melanosomes, where it undergoes proteolytic processing to form fibrils [65]. These fibrils act as a scaffold for the deposition of melanin pigments, catalyzed by enzymes like tyrosinase [66, 67]. Mutations in the *PMEL* gene can lead to abnormalities in melanosome formation and melanin deposition, impacting plumage coloration in various bird species, including chickens [38, 68], Junco hyemalis [69], Japanese quail [70] and Indian peafowl [40]. To date, only 21 bird species have annotated the *PMEL* gene among 120 bird genomes (Supplementary Table S11). However, the association between the *PMEL* gene and duck plumage color phenotype has not been previously reported.

In this study, the *PMEL* gene was found to be significantly differentially expressed between the

feather bulb specimens of white- and black-feathered ducks (ANOVA, $p<0.001$). The immunofluorescence results indicated high expression of the PMEL protein in feather follicle specimens of black and grey plumage ducks, contrasting with low expression in white plumage ducks. Many other studies suggested the possible involvement of the *PMEL* gene in the deposition of feather melanin [69, 71, 72, 73]. Meanwhile, the *PMEL* gene is also implicated in the formation of white feathers in quail [74] and in the white feathers of chickens at the hatch stage [75]. Endogenous *PMEL* expression is regulated by *MITF*, with alterations observed in melanoma cells [76]. However, the specific interplay between these two genes in determining the plumage color of Liancheng ducks requires further investigation. Overall, this research has annotated the PMEL gene in the duck genome for the first time (Supplementary Fig. S4, Supplementary Table S12), a gene previously thought to be absent. *MITF*, a key regulator of melanin production in ducks, was also identified. The inactivation of *PMEL* in feather bulb specimens led to the distinct white and black feathering characteristic of Liancheng ducks.

In the candidate region identified through GWAS analysis, a total of 12 SNPs, 2 Indel variations, and 1 CNV variation were investigated (Supplementary Table S10; Fig. S7 and S8). Among these variants, only 2 SNPs (Chr33:5,303,994A>G and 5,303,997A>G) were found to be consistently associated with the observed plumage color phenotypes across multiple breeds. Data from promoter activity assays indicated that the SNPs in question were unlikely to serve as direct pro *PMEL* moters for gene expression (Fig. 6C). Instead, the present genetic findings indicated that these two linked SNP variations, located in the upstream region of the *PMEL* gene, exhibited functional enhancer activity that might remotely regulate *PMEL* gene expression (Fig. S5 and 6). The long-range

regulation probably modulates PMEL gene expression, affecting the black feather pigmentation in ducks, aligning with the melanin phenotype of duck feathers.

Remote regulation elements are thought to engage with target promoters through physical proximity [77], yet the precise functional implications of this proximity are not well defined. In the current research, potential regions encompassing *PMEL* and two candidate SNPs were identified within a single topologically associated domain region (Fig. 5), demonstrating this area as a possible part of a genomic region with frequent interactions. The process of loop extrusion not only promoted interactions within the TAD but also shielded the TAD from external influences [78]. Furthermore, enhancer-promoter interactions might intensify during mammalian development [79], potentially accounting for the variation in plumage colors of Liancheng ducks from embryonic to postnatal stages. It was also noted that only SOX5, the predicted transcription factor, displayed varying expression levels in feather bulbs of different plumage colors (Supplementary Fig. S7). Notably, other transcription factors might also contribute to the regulatory mechanism. Moreover, the gray plumage, an intermediate phenotype observed in this study, could be related to a haploinsufficiency effect [80].

Feather phenotype is a complex trait that encompasses a series of interlinked modules [81]. Birds exhibit a wide array of elaborate pigmentation patterns, which serve various functions such as attracting mates or providing camouflage or intimidation against predators. Melanin is a crucial pigment in feather coloration, and its role is mediated through the regulated presence, distribution, and differentiation of these melanocytes. Recent studies have reported that the variation of *MITF*,

PMEL, *TYR*, *EDNRB2*, *SLC45A2*, *MC1R* genes and Agouti signaling protein can regulate the production of feather melanin in ducks [2, 82]. However, the role of the *PMEL* gene in feather melanin formation in ducks has not been confirmed. The present study makes the first attempt to elucidate the role of the *PMEL* gene in pigmentation within duck feathers, offering valuable insights into the genetic mechanisms behind plumage coloration. It also holds practical implications for selective breeding and conservation initiatives.

Data Availability

The whole-genome sequence data reported in this article have been deposited in the NCBI under accession number No. PRJNA1107839. The resequencing raw data have been deposited in the NCBI SRA under accession No. PRJNA844232. The transcriptomic raw data have been deposited in the NCBI under accession No. PRJNA1109286. All additional supporting data are available in the *GigaScience* repository, GigaDB [83].

Additional Files

Supplementary Figure 1. Phenotypic characteristics of 1,281 F2 ducks from Liancheng and Pekin ducks.

Supplementary Figure 2. Screening for the candidate region associated with the white plumage of Liancheng ducks by GWAS in 188 ducks from a cross between Liancheng and Pekin ducks.

Supplementary Figure 3. Manhattan plot showing the genetic effects on the plumage color according to a GWAS from a cross of Liancheng and Pekin ducks except WY ducks based on Liancheng duck genome (GCA_039998735.1).

Supplementary Figure 4. The collinearity analysis of comparison between the *PMEL* gene and its upstream and downstream 100kb region in Liancheng duck and other birds.

Supplementary Figure 5. Illustration of the read depth analysis that confirmed the copy number variations on GWAS candidate region (Chr33: 5.24-5.32Mb).

Supplementary Figure 6. Genotypes of candidate CNV variation (Chr33:5,282,001- 5,284,500) in different plumage color populations.

Supplementary Figure 7. Heatmap cluster analysis of transcription factors.

Supplementary Figure 8. Manhattan plot showing the genetic effects on the plumage color according to a GWAS in ducks from a cross of Liancheng and Pekin ducks based on previous reference genome of Pekin duck (GCA_015476345.1).

Supplementary Figure 9. Phenotypic characteristics of Liancheng ducks, Pekin ducks, Mallards during embryonic and postnatal periods.

Supplementary Table 1. List of 366 duck genome resequencing used in the study.

Supplementary Table 2. List of 37 ducks for RNA-seq used in the study.

Supplementary Table 3. The information on the primers used for qPCR.

Supplementary Table 4. Table Summary of Hi-C reads mapping results.

Supplementary Table 5. The Genome Scaffolds and Contigs information of Liancheng duck genome (GCA_039998735.1).

Supplementary Table 6. Assembly statistics of the Liancheng duck genome and previous duck genomes.

Supplementary Table 7. Complete evaluation table of Liancheng duck genome BUSCOs.

Supplementary Table 8. Summary of chromosome regions on the genome.

Supplementary Table 9. Frequency distribution of 6.6kb insertion within *MITF* gene in duck population.

Supplementary Table 10. Genotypic distribution of SNP candidate variations in different duck breeds based on reference genome Liancheng duck.

Supplementary Table 11. All birds with *PMEL* genes in their avian genomes.

Supplementary Table 12. The blast results of sequence identity between the newly annotated mRNA sequence of *PMEL* gene in Liancheng duck and other birds.

Abbreviations

LC: Liancheng duck; PK: Pekin duck; WB: White-feathered ducks with black beaks; GF: Gray-feathered ducks with black beaks; BF: Black-feathered ducks with black beaks; WY: White-feathered ducks with yellow beaks; MD: Mallard; PT: Putian ducks; BLAST: Basic Local Alignment Search Tool; bp: Base pairs; kb: Kilobase pairs; Mb: Megabase pairs; Gb: Gigabase pairs; FC: Fold change; IBD: Identity By Descent; BUSCO: Benchmarking universal single copy orthologs; BWA: Burrows wheeler aligner; NCBI: National Center for Biotechnology Information; PacBio: Pacific Biosciences; HiFi: High Fidelity reads; Hi-C: High-throughput/resolution chromosome conformation capture; RNA-seq: RNA sequencing; SNP: Single nucleotide polymorphism; SRA: Sequence Read Archive; PASA: Program to Assemble Spliced Alignments; Go: Gene Ontology; KEGG: Kyoto Encyclopedia of Genes and Genomes; NR: Non-Redundant Protein Database; eggNOG: Evolutionary genealogy of genes: Non-supervised Orthologous; SWISS-PROT: Swiss-Prot Protein Sequence Database; MAF: Minor allele frequency; PCA: Principal component analysis; ΔAF: Allele frequency difference; CPM: Counts per million; Indel:

Insertion and deletion; CNV: Copy number variation; F_{ST}: Fixation index; GC: Guanine-cytosine; GWAS: Genome-wide association study; TAD: Topologically associated domain; DEF: Duck embryo fibroblast; A375: Human melanoma cells.

Funding

This work was supported by grants from the National Science Fund for Distinguished Young Scholars (32325047), the Innovation Program of Chinese Academy of Agricultural Sciences(CAAS--SCAB-202302), the China Agriculture Research System of MOF and MARA (CARS-42-05), and the National Key R&D Program of China (2022YFF1000102). The authors were grateful to Shandong Rongda Agricultural Development Co., Ltd for their help in this sample collection.

Competing Interests

The authors have declared no competing interests.

Ethics Statement

All animals used in the study were treated following the guidelines for the experimental animals established by the Council of China Animal Welfare. Protocols of the experiments were ap-proved by the Science Research Department of the Institute of Animal Sciences, Chinese Academy of Agricultural Sciences (CAAS) (Beijing, China).

Authors' Contributions

Z.Zhou. and S.Hou conceived the project, designed the research, and managed the project. Z.Guo., Z.Wang., H.Tang., H.Zhang. constructed the population. T.Liu., D.Liu. and Z.Wang. collected the phenotype data. Z.Wang., S.Yu. and H.Liu. performed the genome assembly. Z.Wang., B.Zhang. and J.Cao. performed the experiments. Z.Wang., H.Liu., D.Liu. and Q.Mou. performed bioinformatics analysis. Z.Wang., M.Schroyen. and Z.Zhou. wrote the manuscript.

References

1.Ito S, Wakamatsu K. Quantitative analysis of eumelanin and pheomelanin in humans, mice, and other animals: a comparative review. *Pigment Cell Res*. 2003;**16**:523-531.

2.Zhou Z, Li M, Cheng H, et al. An intercross population study reveals genes associated with body size and plumage color in ducks. *Nat Commun*. 2018;**9**:2648.

3.Land EJ, Riley PA. Spontaneous redox reactions of dopaquinone and the balance between the eumelanic and phaeomelanic pathways. *Pigment Cell Res*. 2000;**13**:273-277.

4.Wang Z, Guo Z, Mou Q, et al. Unique feather color characteristics and transcriptome analysis of hair follicles in Liancheng White ducks. *Poult Sci*, 2024;**103**:103794.

5.Haase E, Ito S, Wakamatsu K. Influences of sex, castration, and androgens on the eumelanin and pheomelanin contents of different feathers in wild mallards. *Pigment Cell Res*. 1995;**8**:164-170.

6.Huang Y, Li Y, Burt DW, et al. The duck genome and transcriptome provide insight into an avian influenza virus reservoir species. *Nat Genet*. 2013;**45**:776-783.

7.Li J, Zhang J, Liu J, et al. A new duck genome reveals conserved and convergently evolved chromosome architectures of birds and mammals. *Gigascience*. 2021;**10**:giaa142.

8.Liu D, Zhang H, Yang Y, et al. Metabolome-Based Genome-Wide Association Study of Duck

Meat Leads to Novel Genetic and Biochemical Insights. *Adv Sci*. 2023;**10**:e2300148.

9.Zhu F, Yin ZT, Wang Z, et al. Three chromosome-level duck genome assemblies provide insights into genomic variation during domestication. *Nat Commun*. 2021;**12**:5932.

10.Yu S, Liu Z, Li M, et al. Resequencing of a Pekin duck breeding population provides insights into the genomic response to short-term artificial selection. *Gigascience*. 2023;**12**:giad016.

11.Wang K, Hua G, Li J, et al. Duck pan-genome reveals two transposon insertions caused bodyweight enlarging and white plumage phenotype formation during evolution. *IMeta*. 2024;**3**,e154.

12.Liu H, Xi Y, Tang Q, et al. Genetic fine-mapping reveals single nucleotide polymorphism mutations in the MC1R regulatory region associated with duck melanism. *Mol Ecol*. 2023;**32**:3076-3088.

13.Wenger, A.M., Peluso, P., Rowell, W.J. et al. Accurate circular consensus long-read sequencing improves variant detection and assembly of a human genome. *Nat Biotechnol.* 2019; **37**:1155–1162.

14.Cooke TF, Fischer CR, Wu P, et al. Genetic Mapping and Biochemical Basis of Yellow Feather Pigmentation in Budgerigars. *Cell*. 2017;**171**:427-439.

15.Si S, Xu X, Zhuang Y, et al. The genetics and evolution of eye color in domestic pigeons (*Columba livia*). *PLoS Genet*. 2021;**17**:e1009770.

16. Li S, Wang C, Yu W, et al. Identification of genes related to white and black plumage formation by RNA-Seq from white and black feather bulbs in ducks. *PLoS One*. 2012;**7**:e36592.

17. Lin R, Lin W, Zhou S, et al. In'tegrated Analysis of mRNA Expression, CpG Island Methylation, and Polymorphisms in the *MITF* Gene in Ducks (*Anas platyrhynchos*). *Biomed Res Int*.

2019;**2019**:8512467.

18. Cheng H, Concepcion GT, Feng X, et al. Haplotype-resolved *de novo* assembly using phased assembly graphs with hifiasm. *Nat Methods*. 2021;**18**:170-175.

19. Durand NC, Shamim MS, Machol I, et al. Juicer Provides a One-Click System for Analyzing Loop-Resolution Hi-C Experiments. *Cell Syst*. 2016;**3**:95-98.

20. Zheng Z, Lai Z, Wu B, et al. The first high-quality chromosome-level genome of the Sipuncula *Sipunculus nudus* using HiFi and Hi-C data. *Sci Data*. 2023;**10**:317.

21. Dudchenko O, Batra SS, Omer AD, et al. *De novo* assembly of the *Aedes aegypti* genome using Hi-C yields chromosome-length scaffolds. *Science*. 2017;**356**:92-95.

22. Manni M, Berkeley MR, Seppey M, et al. BUSCO: Assessing Genomic Data Quality and Beyond. *Curr Protoc*. 2021;**1**:e323.

23. Powell S, Forslund K, Szklarczyk D, et al. eggNOG v4.0: nested orthology inference across 3686 organisms. *Nucleic Acids Res*. 2014;**42**:D231-D239.

24. Bolger AM, Lohse M, Usadel B. Trimmomatic: a flexible trimmer for Illumina sequence data. *Bioinformatics*. 2014;**30**:2114-2120.

25. Grewal S, Yang CY, Scholefield D, et al. Chromosome-scale genome assembly of bread wheat's wild relative *Triticum timopheevii*. *Sci Data*. 2024;**11**:420.

26. Li H, Durbin R. Fast and accurate short read alignment with Burrows-Wheeler transform. *Bioinformatics*. 2009;**25**:1754-1760.

27. McKenna A, Hanna M, Banks E, et al. The Genome Analysis Toolkit: a MapReduce framework for analyzing next-generation DNA sequencing data. *Genome Res*. 2010;**20**:1297-1303.

28. Kang HM, Sul JH, Service SK, et al. Variance component model to account for sample structure

in genome-wide association studies. *Nat Genet*. 2010;**42**:348-354.

29. Wang K, Hua G, Li J, et al. Duck pan-genome reveals two transposon insertions caused bodyweight enlarging and white plumage phenotype formation during evolution. *Imeta*. 2023;**3**:e154.

30. Price AL, Patterson NJ, Plenge RM, et al. Principal components analysis corrects for stratification in genome-wide association studies. *Nat Genet*. 2006;**38**:904-909.

31. Anders S, Pyl PT, Huber W. HTSeq--a Python framework to work with high-throughput sequencing data. *Bioinformatics*. 2015;**31**:166-169.

32. Schmittgen TD, Livak KJ. Analyzing real-time PCR data by the comparative C(T) method. *Nat Protoc*. 2008;**3**:1101-1108.

33. Servant N, Varoquaux N, Lajoie BR, et al. HiC-Pro: an optimized and flexible pipeline for Hi-C data processing. *Genome Biol*. 2015;**16**:259.

34. Akdemir KC, Chin L. HiCPlotter integrates genomic data with interaction matrices. *Genome Biol*. 2015;**16**:198.

35. Guo Y, Gu X, Sheng Z, et al. A Complex Structural Variation on Chromosome 27 Leads to the Ectopic Expression of HOXB8 and the Muffs and Beard Phenotype in Chickens. *PLoS Genet*. 2016;**12**:e1006071.

36. Wang X, Zheng Z, Cai Y, et al. CNVcaller: highly efficient and widely applicable software for detecting copy number variations in large populations. *Gigascience*. 2017;**6**:1-12.

37. Chen L, Gu X, Huang X, et al. Two cis-regulatory SNPs upstream of ABCG2 synergistically cause the blue eggshell phenotype in the duck. *PLoS Genet*. 2020;**16**:e1009119.

38. Keeling L, Andersson L, Schütz KE, et al. Chicken genomics: feather-pecking and victim

pigmentation. *Nature*. 2004;**431**:645-646.

39. Gunnarsson U, Kerje S, Bed'hom B, et al. The Dark brown plumage color in chickens is caused by an 8.3-kb deletion upstream of SOX10. *Pigment Cell Melanoma Res*. 2011;**24**:268-274.

40. Liu S, Chen H, Ouyang J, et al. A high-quality assembly reveals genomic characteristics, phylogenetic status, and causal genes for leucism plumage of Indian peafowl. *Gigascience*. 2022;**11**:giac018.

41. Xi Y, Wang L, Liu H, et al. A 14-bp insertion in endothelin receptor B-like (EDNRB2) is associated with white plumage in Chinese geese. *BMC Genomics*. 2020;**21**:162.

42. Wang L, Yang L, Yang S, et al. Identification of genes associated with feather color in Liancheng white duck using F_{ST} analysis. *Anim Genet*. 2022;**53**:518-521.

43. Gong Y, Yang Q, Li S, et al. Grey plumage colouration in the duck is genetically determined by the alleles on two different, interacting loci. *Anim Genet*. 2010;**41**:105-108.

44. Yang L, Mo C, Shen W, et al. The recessive C locus in the *MITF* gene plays a key regulatory role in the plumage colour pattern of duck (*Anas platyrhynchos*). *Br Poult Sci*. 2019;**60**:105-108.

45. Jiang F, Jiang Y, Wang W, et al. A chromosome-level genome assembly of Cairina moschata and comparative genomic analyses. *BMC Genomics*. 2021;**22**:581

46. Hu J, Song L, Ning M, et al. A new chromosome-scale duck genome shows a major histocompatibility complex with several expanded multigene families. *BMC Biol*. 2024;**22**:31.

47. Ng CS, Lai CK, Ke HM, et al. Genome Assembly and Evolutionary Analysis of the Mandarin Duck Aix galericulata Reveal Strong Genome Conservation among Ducks. *Genome Biol Evol*. 2022;**14**:evac083.

48. Lavretsky P, Hernández F, Swale T, et al. Chromosomal-level reference genome of a wild North American mallard (*Anas platyrhynchos*). *G3*. 2023;**13**:jkad171.

49. Xu MM, Gu LH, Lv WY, et al. Chromosome-level genome assembly of the Muscovy duck provides insight into fatty liver susceptibility. *Genomics*. 2022;**114**:110518.

50. Chang G, Yuan X, Guo Q, et al. The first crested duck genome reveals clues to genetic compensation and crest cushion formation. *Genomics Proteomics Bioinformatics*. 2023;**21**:483-500.

51. Mueller RC, Ellström P, Howe K, et al. A high-quality genome and comparison of short- versus long-read transcriptome of the palaearctic duck Aythya fuligula (tufted duck). *Gigascience*. 2021;**10**:giab081.

52. Dürig N, Letko A, Lepori V, et al. Two MC1R loss-of-function alleles in cream-coloured Australian Cattle Dogs and white Huskies. *Anim Genet*. 2018;**49**:284-290.

53. Guo Q, Jiang Y, Wang Z, et al. Genome-Wide Analysis Identifies Candidate Genes Encoding Feather Color in Ducks. *Genes*. 2022;**13**:1249.

54. Pan R, Hua T, Guo Q, et al. Identification of SNPs in MITF associated with beak color of duck. *Front Genet*. 2023;**14**:1161396.

55. Karlsson EK, Baranowska I, Wade CM, et al. Efficient mapping of mendelian traits in dogs through genome-wide association. *Nat Genet*. 2007;**39**:1321-1328.

56. Lin R, Zhao F, Xiong T, et al. Genetic mapping identifies SNP mutations in MITF-M promoter associated with melanin formation in Putian black duck. *Poult Sci*. 2024;**103**:103191.

57. Baranowska Körberg I, Sundström E, Meadows JR, et al. A simple repeat polymorphism in the MITF-M promoter is a key regulator of white spotting in dogs. *PLoS One*. 2014;**9**:e104363.

58. Anello M, Daverio MS, Silbestro MB, et al. Characterization and expression analysis of KIT and MITF-M genes in llamas and their relation to white coat color. *Anim Genet*. 2019;**50**:143-149.

59. Flesher JL, Paterson-Coleman EK, Vasudeva P, et al. Delineating the role of *MITF* isoforms in pigmentation and tissue homeostasis. *Pigment Cell Melanoma Res*. 2020;**33**:279-292.

60. Coding CR, Arnheiter H. MITF-the first 25 years. *Genes Dev*. 2019;**33**:983-1007.

61. Sultana H, Seo D, Choi NR, et al. Identification of polymorphisms in MITF and DCT genes and their associations with plumage colors in Asian duck breeds. *Asian-Australas J Anim Sci*. 2018;**31**:180-188.

62. Ren S, Lyu G, Irwin DM, et al. Pooled sequencing analysis of geese (Anser cygnoides) reveals genomic variations associated with feather color. *Front Genet*. 2021;**12**:650013.

63. Kerje S, Sharma P, Gunnarsson U, et al. The Dominant white, Dun and Smoky color variants in chicken are associated with insertion/deletion polymorphisms in the PMEL17 gene. *Genetics*. 2004;**168**:1507-1518.

64. Batai K, Cui Z, Arora A, et al. Genetic loci associated with skin pigmentation in African Americans and their effects on vitamin D deficiency. *PLoS Genet*. 2021;**17**:e1009319.

65. Watt B, Tenza D, Lemmon MA, et al. Mutations in or near the transmembrane domain alter PMEL amyloid formation from functional to pathogenic. *PLoS Genet*. 2011;**7**:e1002286.

66. Hurbain I, Geerts WJ, Boudier T, et al. Electron tomography of early melanosomes: implications for melanogenesis and the generation of fibrillar amyloid sheets. *Proc Natl Acad Sci U S A*. 2008;**105**:19726-19731.

67. Watt B, van Niel G, Raposo G, Marks MS. PMEL: a pigment cell-specific model for functional

amyloid formation. *Pigment Cell Melanoma Res*. 2013;**26**:300-315.

68. Deng Y, Qu X, Yao Y, et al. Investigating the impact of pigmentation variation of breast muscle on growth traits, melanin deposition, and gene expression in Xuefeng black-bone chickens. *Poult Sci*. 2024;**103**:103691.

69. Abolins-Abols M, Kornobis E, Ribeca P, et al. Differential gene regulation underlies variation in melanic plumage coloration in the dark-eyed junco (Junco hyemalis). *Mol Ecol*. 2018;**27**:4501-4515.

70. Ishishita S, Takahashi M, Yamaguchi K, et al. Nonsense mutation in PMEL is associated with yellowish plumage colour phenotype in Japanese quail. *Sci Rep*. 2018;**8**:16732.

71. Liu X, Zhou R, Peng Y, et al. Feather follicles transcriptome profiles in Bashang long-tailed chickens with different plumage colors. *Genes Genomics*. 2019;**41**:1357-1367.

72. Zheng X, Zhang B, Zhang Y, et al. Transcriptome analysis of feather follicles reveals candidate genes and pathways associated with pheomelanin pigmentation in chickens. *Sci Rep*. 2020;**10**:12088.

73. Heo S, Cho S, Dinh PTN, et al. A genome-wide association study for eumelanin pigmentation in chicken plumage using a computer vision approach. *Anim Genet*. 2023;**54**:355-362.

74. Yuan Z, Zhang X, Pang Y, et al. Association analysis of PMEL gene expression and single nucleotide polymorphism with plumage color in quail. *Anim Biotechnol*. 2023;**34**:5001-5010.

75. Hua G, Chen J, Wang J, et al. Genetic basis of chicken plumage color in artificial population of complex epistasis. *Anim Genet*. 2021;**52**:656-666.

76. Falletta P, Bagnato P, Bono M, et al. Melanosome-autonomous regulation of size and number: the OA1 receptor sustains PMEL expression. *Pigment Cell Melanoma Res*. 2014;**27**:565-579.

77. Soldner F, Stelzer Y, Shivalila CS, et al. Parkinson-associated risk variant in distal enhancer of α-synuclein modulates target gene expression. *Nature*. 2016;**533**:95-99.

78. Hung TC, Kingsley DM, Boettiger AN. Boundary stacking interactions enable cross-TAD enhancer-promoter communication during limb development. *Nat Genet*. 2024;**56**:306-314.

79. Chen Z, Snetkova V, Bower G, et al. Increased enhancer-promoter interactions during developmental enhancer activation in mammals. *Nat Genet*. 2024;**56**:675-685.

80. Billiard S, Castric V, Llaurens V. The integrative biology of genetic dominance. *Biol Rev Camb Philos Soc*. 2021;**96**:2925-2942.

81. Terrill RS, Shultz AJ. Feather function and the evolution of birds. *Biol Rev Camb Philos Soc*. 2023;**98**:540-566.

82. Ng CS, Li WH. Genetic and Molecular Basis of Feather Diversity in Birds. *Genome Biol Evol*. 2018;**10**:2572-2586.

83. Wang Z, Guo Z, Liu H, et al. Supporting data for "A high-quality assembly revealing PMEL gene for unique plumage phenotype of Liancheng ducks" GigaScience Database. 2024. https://doi.org/10.5524/102602.

Supplementary Figures

Click here to access/download
Supplementary Material
Supplementary Figures.docx

Supplementary Tables

Click here to access/download
Supplementary Material
Supplementary Tables.xlsx
