# Supplementary material for: A high-quality assembly revealing the PMEL gene for the unique plumage phenotype in Liancheng ducks
Source: Gigascience. 2025 Jan 13;14:giae114. doi: 10.1093/gigascience/giae114 (PMC11727711; doi:10.1093/gigascience/giae114)
Supplement: giae114_Response_to_Reviewer_Comments_Original_Submission [file giae114_response_to_reviewer_comments_original_submission.pdf]

Author's Response To Reviewer Comments

Dear editors,

Thank you for handing our manuscript. We also thank two reviewers for their critical comments and valuable suggestions, which have significantly improved our manuscript. In the revised version, we have adjusted the logic of the results section, conducted additional analyses.

Below, we offer a point-by-point response and indicate the corresponding modified sections in the revised manuscript, which are marked in green.

We hope that our current manuscript addresses the reviewer's concerns.

Sincerely,

All authors

Reviewer 1#
The manuscript "A high-quality assembly reveals causal gene for unique plumage phenotype of Liancheng ducks" focuses on uncovering the genetic basis of the distinctive white plumage seen in Liancheng ducks. The study aims to understand the genetic determinants of plumage color in Liancheng ducks, a breed known for its unique combination of white feathers with black beaks and feet. The primary focus is on identifying genetic variants that contribute to this phenotype.

The authors constructed a de novo genome assembly for the Liancheng duck using PacBio HiFi sequencing and Hi-C data to achieve this. The assembly achieved significant quality, with a genome size of approximately 1.29 Gb. Additionally, a genome-wide association study (GWAS) was conducted using a mixed linear model to identify significant single nucleotide polymorphisms (SNPs) associated with the white plumage phenotype. The analysis involved 364 individuals from an F2 population derived from crossbreeding Liancheng and Pekin ducks.

The GWAS identified a 0.8 Mb candidate region on Chromosome 33 containing the PMEL gene, a key determinant of plumage color. This region includes two closely linked SNPs (Chr33:5,303,994A>G; 5,303,997A>G) that may alter PMEL gene transcription. Functional assays were performed to validate the impact of these SNPs on gene expression, suggesting that they significantly influence the white plumage phenotype.

The study presents the first high-quality genome assembly of the Liancheng duck and identifies novel genetic variants in the PMEL gene associated with white plumage. These findings provide valuable insights into the genetic mechanisms underlying plumage coloration and offer promising practical implications for selective breeding and conservation efforts.

Specific comments:

1) Discuss how your findings compare with the genetic and molecular basis of feather diversity as highlighted in these review papers:

Terrill RS, Shultz AJ. Feather function and the evolution of birds. Biol Rev Camb Philos Soc. 2023 Apr;98(2):540-566. doi: 10.1111/brv.12918. Epub 2022 Nov 24.

Ng CS, Li WH. Genetic and Molecular Basis of Feather Diversity in Birds. Genome Biol Evol. 2018 Oct 1;10(10):2572-2586. doi: 10.1093/gbe/evy180.

Boer EF, Van Hollebeke HF, Shapiro MD. Genomic determinants of epidermal appendage patterning and structure in domestic birds. Dev Biol. 2017 Sep 15;429(2):409-419.

Chen CF, Foley J, Tang PC, Li A, Jiang TX, Wu P, Widelitz RB, Chuong CM. Development, regeneration, and evolution of feathers. Annu Rev Anim Biosci. 2015;3:169-95. doi: 10.1146/annurev-animal-022513-114127.
Response: Thank you for your comments. We have revised the discussion section and added those references to highlight the different and innovation of our new findings.

"Feather phenotype is a complex trait composed of a series of stratified modules [81]. Birds can be highly decorated with distinct and colorful pigmentation patterns, which are used to attract a mate or to hide from or frighten a potential predator. Melanin plays an important role in the formation of feather pigments, which is achieved through the regulation of the presence, distribution, and differentiation of these melanocytes. Recent studies have reported that the variation of MITF, PMEL, TYR, EDNRB2, SLC45A2, MC1R genes and Agouti signaling protein can regulate the production of feather melanin in ducks [2, 82]. However, the role of PMEL gene in feather melanin formation in ducks has not been confirmed. Our study is the first to report the role of the PMEL gene in pigment formation in duck feathers and provide valuable insights into the genetic mechanisms underlying plumage coloration, as well as promising practical implications for selective breeding and conservation efforts" (Line 583-593 in the revised manuscript).

2) Integrate these studies to provide a broader context for the role of PMEL in different bird species and its implications for plumage color:

Yuan Z, Zhang X, Pang Y, Qi Y. Association analysis of PMEL gene expression and single nucleotide polymorphism with plumage color in quail. Anim Biotechnol. 2023 Dec;34(9):5001-5010. doi: 10.1080/10495398.2023.2221697.

Ishishita S, Takahashi M, Yamaguchi K, Kinoshita K, Nakano M, Nunome M, Kitahara S, Tatsumoto S, Go Y, Shigenobu S, Matsuda Y. Nonsense mutation in PMEL is associated with yellowish plumage colour phenotype in Japanese quail. Sci Rep. 2018 Nov 13;8(1):16732. doi: 10.1038/s41598-018-34827-4.

Liu X, Zhou R, Peng Y, Zhang C, Li L, Lu C, Li X. Feather follicles transcriptome profiles in Bashang long-tailed chickens with different plumage colors. Genes Genomics. 2019 Nov;41(11):1357-1367. doi: 10.1007/s13258-018-0740-y.

Zheng X, Zhang B, Zhang Y, Zhong H, Nie R, Li J, Zhang H, Wu C. Transcriptome analysis of feather follicles reveals candidate genes and pathways associated with pheomelanin pigmentation in chickens. Sci Rep. 2020 Jul 21;10(1):12088. doi: 10.1038/s41598-020-68931-1.

Hua G, Chen J, Wang J, Li J, Deng X. Genetic basis of chicken plumage color in artificial population of complex epistasis. Anim Genet. 2021 Oct;52(5):656-666. doi: 10.1111/age.13094.

Weng Z, Xu Y, Li W, Chen J, Zhong M, Zhong F, Du B, Zhang B, Huang X. Genomic variations and signatures of selection in Wuhua yellow chicken. PLoS One. 2020 Oct 23;15(10):e0241137. doi: 10.1371/journal.pone.0241137.

Heo S, Cho S, Dinh PTN, Park J, Jin DH, Cha J, Kim YK, Koh YJ, Lee SH, Lee JH. A genome-wide association study for eumelanin pigmentation in chicken plumage using a computer vision approach. Anim Genet. 2023 Jun;54(3):355-362. doi: 10.1111/age.13303.

Abolins-Abols M, Kornobis E, Ribeca P, Wakamatsu K, Peterson MP, Ketterson ED, Milá B. Differential gene regulation underlies variation in melanic plumage coloration in the dark-eyed junco (Junco hyemalis). Mol Ecol. 2018 Nov;27(22):4501-4515. doi: 10.1111/mec.14878.

Yuan H, Zhang X, Zhang Q, Wang Y, Wang S, Li Y, Zhang Y, Jing J, Qiu J, Wang Z, Leng L. Comparative transcriptome profiles of Lindian chicken eyelids identify melanin genes controlling eyelid pigmentation. Br Poult Sci. 2019 Feb;60(1):15-22. doi: 10.1080/00071668.2018.1544414.
Response: Thank you for your suggestions. References are included in the discussion section of the revised manuscript. "PMEL, a type I transmembrane transport glycoprotein, is synthesized in the endoplasmic

reticulum and plays a crucial role in amyloid fiber formation during stages I and II of melanosome formation in the L-DOPA pathway [63, 64]. After synthesis, PMEL is transported to melanosomes, where it undergoes proteolytic processing to form fibrils [65]. These fibrils act as a scaffold for the deposition of melanin pigments, catalyzed by enzymes like tyrosinase [66, 67]. Mutations in the PMEL gene can lead to abnormalities in melanosome formation and melanin deposition, impacting plumage coloration in various bird species, including chickens [38, 68], Junco hyemalis [69], Japanese quail [70] and Indian peafowl [40]. To date, only 21 bird species have annotated the PMEL gene among 120 bird genomes (Table S11). However, the association between the PMEL gene and duck plumage color phenotype has not been previously explored.

In this study, the PMEL gene was found to be significantly differentially expressed between the feather bulb specimens of white- and black-feathered ducks (ANOVA, $p<0.001$). Immunofluorescence results indicated high expression of the PMEL protein in feather follicle specimens of black and grey plumage ducks, contrasting with low expression in white plumage ducks. Many studies suggested that the deposition of feather melanin may involve the PMEL gene [69, 71, 72, 73]. Meanwhile, the PMEL gene is also implicated in the formation of white feathers in quail [74] and in the white feathers of chickens at the hatch stage [75]. Endogenous PMEL expression is regulated by MITF, with alterations observed in melanoma cells [76]. However, the specific interplay between these two genes in determining the plumage color of Liancheng ducks requires further investigation. Overall, we have provided the first annotation of the PMEL gene in the newly sequenced duck genome (Figure S4, Table S12), previously believed to lack this gene. MITF functions as a key gene that governs melanin production in ducks. Subsequent inactivation of the PMEL gene, situated in the feather bulb specimens resulting in the distinctive white feather and black feathers observed in Liancheng ducks." (Line 532-556 in the revised manuscript).

3) Discuss the potential role of MITF in plumage coloration, referencing these studies to highlight its regulatory mechanisms and effects:

Goding CR, Arnheiter H. MITF-the first 25 years. Genes Dev. 2019 Aug 1;33(15-16):983-1007. doi: 10.1101/gad.324657.119. Epub 2019 May 23.

Yuan B, Qi Y, Zhang X, Hu J, Fan Y, Ji X. The relationship of MITF gene expression and promoter methylation with plumage colour in quail. Br Poult Sci. 2024 Jun;65(3):259-264. doi: 10.1080/00071668.2024.2326962.

Pan R, Hua T, Guo Q, Bai H, Jiang Y, Wang Z, Bi Y, Chen G, Wu X, Chang G. Identification of SNPs in MITF associated with beak color of duck. Front Genet. 2023 Aug 21;14:1161396. doi: 10.3389/fgene.2023.1161396.

Lin R, Lin W, Zhou S, Chen Q, Pan J, Miao Y, Zhang M, Huang Z, Xiao T. Integrated Analysis of mRNA Expression, CpG Island Methylation, and Polymorphisms in the MITF Gene in Ducks (Anas platyrhynchos). Biomed Res Int. 2019 Sep 23;2019:8512467. doi: 10.1155/2019/8512467.

Guo Q, Jiang Y, Wang Z, Bi Y, Chen G, Bai H, Chang G. Genome-Wide Analysis Identifies Candidate Genes Encoding Feather Color in Ducks. Genes (Basel). 2022 Jul 14;13(7):1249. doi: 10.3390/genes13071249.

Ren S, Lyu G, Irwin DM, Liu X, Feng C, Luo R, Zhang J, Sun Y, Shang S, Zhang S, Wang Z. Pooled Sequencing Analysis of Geese (Anser cygnoides) Reveals Genomic Variations Associated With Feather Color. Front Genet. 2021 Jun 18;12:650013. doi: 10.3389/fgene.2021.650013.

Lin R, Zhao F, Xiong T, Lai L, Li H, Lin W, Xiao T, Lin W. Genetic mapping identifies SNP mutations in MITF-M promoter associated with melanin formation in Putian black duck. Poult Sci. 2024 Jan;103(1):103191. doi: 10.1016/j.psj.2023.103191.

Sultana H, Seo D, Choi NR, Bhuiyan MSA, Lee SH, Heo KN, Lee JH. Identification of polymorphisms in MITF and DCT genes and their associations with plumage colors in Asian duck breeds. Asian-Australas J Anim Sci. 2018 Feb;31(2):180-188. doi: 10.5713/ajas.17.0298.

Zhang X, Zhu T, Wang L, Lv X, Yang W, Qu C, Li H, Wang H, Ning Z, Qu L. Genome-Wide Association Study Reveals the Genetic Basis of Duck Plumage Colors. Genes (Basel). 2023 Mar 31;14(4):856. doi: 10.3390/genes14040856.

Li S, Wang C, Yu W, Zhao S, Gong Y. Identification of genes related to white and black plumage formation by RNA-Seq from white and black feather bulbs in ducks. PLoS One. 2012;7(5):e36592. doi: 10.1371/journal.pone.0036592.

Wang L, Guo J, Xi Y, Ma S, Li Y, He H, Wang J, Han C, Bai L, Mustafa A, Liu H, Li L. Understanding the Genetic Domestication History of the Jianchang Duck by Genotyping and Sequencing of Genomic Genes Under Selection. G3 (Bethesda). 2020 May 4;10(5):1469-1476. doi: 10.1534/g3.119.400893.
Response: Thank you for your comments. We have supplemented the mechanism of MITF in the formation of feather color in the discussion section. "MITF regulates the expression of enzymes responsible for melanin synthesis and the expression of receptors involved in melanocyte function [52, 53, 54]. MITF produces various isoforms through alternative promoters with shared coding exons but distinct amino termini [55]. While MITF variants are known to influence melanin regulation, the regulation of these isoforms remained unclear. In ducks, we discovered the expression of two MITF isoforms, MITF-B and MITF-M, with only the latter being crucial for melanin synthesis in duck plumage [2,9, 56]. MITF-M isoforms also have been shown to regulate white coloration in the fur of dogs [57], llamas [58], and mice [59]. Previous research found SNPs, indels, and structural variants in MITF as possible causes of white plumage in ducks [60]. Two synonymous SNPs (c.114T>G and c.147T>C) and a 14-bp indel (GCTGCAAAC AGATG) in intron 7 of duck MITF were significantly associated with the black- and white-colored breeds ($p<0.001$) [61]. One variant in the 5'UTR of MITF were significantly associated with feather color phenotypes in geese [62]. A 6.6 kb insertion within the MITF gene demonstrated a strong correlation with melanin production in ducks [2] and indicated that MITF played an on-off role in the melanin generation pathway of Pekin ducks. MITF can promote differentiation-related functions, including regulation of genes involved in pigmentation, such as PMEL, TYR, TYRP1, DCT, MLANA, SILV, and SLC24A5 [60]. In the years following the separation of MITF gene, the number of potential target genes increased sharply. Based on GWAS analysis, it was confirmed that the MITF gene acts as an epistatic gene controlling melanin synthesis in Liancheng ducks, aligning with previous research findings [44]. This highlights the significant regulatory role of MITF in melanin synthesis in Liancheng ducks and underscores its importance as a key genetic factor in pigmentation.
" (Line 510-530 in the revised manuscript).

4) Compare your genome assembly results with these recently published assemblies to provide a comprehensive understanding of the advancements and differences:

Xu MM, Gu LH, Lv WY, Duan SC, Li LW, Du Y, Lu LZ, Zeng T, Hou ZC, Ma ZS, Chen W, Adeola AC, Han JL, Xu TS, Dong Y, Zhang YP, Peng MS. Chromosome-level genome assembly of the Muscovy duck provides insight into fatty liver susceptibility. Genomics. 2022 Nov;114(6):110518. doi: 10.1016/j.ygeno.2022.110518.

Ng CS, Lai CK, Ke HM, Lee HH, Chen CF, Tang PC, Cheng HC, Lu MJ, Li WH, Tsai IJ. Genome Assembly and Evolutionary Analysis of the Mandarin Duck Aix galericulata Reveal Strong Genome Conservation among Ducks. Genome Biol Evol. 2022 May 31;14(6):evac083. doi: 10.1093/gbe/evac083.

Che T, Li J, Li X, Wang Z, Zhang X, Yang W, Liu T, Wang Y, Wang K, Gao T, Shen G, Qiu W, Li Z, Zhang W. Haplotype-resolved assembly of the mule duck genome using high-fidelity sequencing technology. PLoS One. 2024 Jul 1;19(7):e0305914. doi: 10.1371/journal.pone.0305914.

Jiang F, Jiang Y, Wang W, Xiao C, Lin R, Xie T, Sung WK, Li S, Jakovlić I, Chen J, Du X. A chromosome-level genome assembly of Cairina moschata and comparative genomic analyses. BMC Genomics. 2021 Jul 30;22(1):581. doi: 10.1186/s12864-021-07897-4.

Mueller RC, Ellström P, Howe K, Uliano-Silva M, Kuo RI, Miedzinska K, Warr A, Fedrigo O, Haase B, Mountcastle J, Chow W, Torrance J, Wood JMD, Järhult JD, Naguib MM, Olsen B, Jarvis ED, Smith J, Eöry L, Kraus RHS. A high-quality genome and comparison of short- versus long-read transcriptome of the

palaearctic duck Aythya fuligula (tufted duck). Gigascience. 2021 Dec 20;10(12):giab081. doi: 10.1093/gigascience/giab081.

Hu J, Song L, Ning M, Niu X, Han M, Gao C, Feng X, Cai H, Li T, Li F, Li H, Gong D, Song W, Liu L, Pu J, Liu J, Smith J, Sun H, Huang Y. A new chromosome-scale duck genome shows a major histocompatibility complex with several expanded multigene families. BMC Biol. 2024 Feb 5;22(1):31. doi: 10.1186/s12915-024-01817-0.

Lavretsky P, Hernández F, Swale T, Mohl JE. Chromosomal-level reference genome of a wild North American mallard (Anas platyrhynchos). G3 (Bethesda). 2023 Sep 30;13(10):jkad171. doi: 10.1093/g3journal/jkad171.

Wang K, Hua G, Li J, Yang Y, Zhang C, Yang L, Hu X, Scheben A, Wu Y, Gong P, Zhang S, Fan Y, Zeng T, Lu L, Gong Y, Jiang R, Sun G, Tian Y, Kang X, Hu H, Li W. Duck pan-genome reveals two transposon insertions caused bodyweight enlarging and white plumage phenotype formation during evolution. Imeta. 2023 Dec 17;3(1):e154. doi: 10.1002/imt2.154.

Chang G, Yuan X, Guo Q, Bai H, Cao X, Liu M, Wang Z, Li B, Wang S, Jiang Y, Wang Z, Zhang Y, Xu Q, Song Q, Pan R, Qiu L, Gu T, Wu X, Bi Y, Cao Z, Zhang Y, Chen Y, Li H, Liu J, Dai W, Chen G. The First Crested Duck Genome Reveals Clues to Genetic Compensation and Crest Cushion Formation. Genomics Proteomics Bioinformatics. 2023 Jun;21(3):483-500. doi: 10.1016/j.gpb.2023.08.002.
Response: Thank you for your suggestions. We have added this content. By comparing the new genome assembly results with these recently published assemblies, we want to provide a comprehensive understanding of the advancements and differences. References are included in the discussion section of the revised manuscript. “The white plumage phenotype is a common trait observed in various avian species, such as chickens [38, 39] , peafowl [40] , geese [41] , also in ducks [2, 42]. Extensive research has focused on the unique white plumage phenotype of Liancheng ducks [42, 43, 44]; however, the genetic mechanisms behind its plumage color remain unclear. Our findings suggest that the inheritance of plumage color phenotype in Liancheng ducks is likely governed by two autosomal genes, independent of sex chromosomes (Table 1). Despite using the previous Pekin duck reference genome (IASCAAS_PekingDuck_PBH1.5, GCA_003850225.1), our GWAS analysis identified three signals (refer to Figure S8), which we believed may be due to incomplete assembly. To further investigate the white feather phenotype of Liancheng ducks, we first established a high-quality genome for Liancheng duck (refer to Figure 1). This new reference genome has a size of 1.29 Gb, with contig and scaffold N50 values of 12.17 and 83.98Mb, respectively (refer to Table S5). The scaffold N50 length of the Liancheng duck genome is higher than that of other duck genomes [45, 46, 47, 48], representing a more complete genome assembly that better reflects the continuity of the duck genome. However, the chromosomes number identified from this newly genome still needs improvement compared to those of the Muscovy duck and Crested Duck [49, 50]. Further pan-genome and functional gene-mining analysis can be conducted in the future [29, 47, 51]. Taken together, these findings firstly represent the first construction of the Liancheng duck genome, resulting in enhanced genome contiguity compared to previous duck genomes.” (Line 491-508 in the revised manuscript).

5) Replace "hair follicle" with "feather follicle" throughout the manuscript. Hair is a mammalian-specific skin appendage; birds have feathers, not hair.
Response: Thank you for your valuable suggestions. We have replaced "hair follicle" with "feather follicle" throughout the manuscript.

6) Specify which part of the feather follicle was used for RNA-seq extraction.
Response: We have elaborated the sample tissue for feather follicle RNA extraction in whole manuscript. Feather bulb specimens from feather follicle tissues of black-, grey- and white-feathered ducks were collected in this study.

7) Show the statistical test used along with the p-value in parentheses. For example, (ANOVA, $p < 0.05$).
Response: Thank you for your suggestions. We have showed the statistical test used along with the p-value in parentheses throughout the manuscript.

8) Figure 4B: Clarify what the error bars represent. Figure 4C: Explain the asterisk (*) and indicate which statistical test was applied. Figure 6: Specify what the error bars represent and the statistical test used.
Response: The error bars in the manuscript are mean ± standard error, while the ** and *** value symbols represent $p<0.01$ and $p<0.001$, respectively. We have reviewed the full manuscript and made revisions.

9) Clearly state the threshold after Bonferroni correction and justify choosing it over other methods such as FDR.

Response: Bonferroni correction is a conservative correction method. Based on a large amount of data, this method is used in this study to strictly control the error rate and minimize the false positive results. The FDR correction allows some control over the proportion of false positives, so we chose to use the Bonferroni correction. At the same time, many recent gene mining papers have used this method, and I have quoted the references that use this method (Zhou et al., 2018; Liu et al., 2023; Wang et al., 2023) (Line 184 in the revised manuscript).
References:
Zhou Z, Li M, Cheng H, et al. An intercross population study reveals genes associated with body size and plumage color in ducks. Nat Commun. 2018;9:2648.
Wang W, Zhang Y, Zhang X, et al. Heritability and recursive influence of host genetics on the rumen microbiota drive body weight variance in male Hu sheep lambs. Microbiome. 2023;11(1):197.
Liu D, Zhang H, Yang Y, et al. Metabolome-Based Genome-Wide Association Study of Duck Meat Leads to Novel Genetic and Biochemical Insights. Adv Sci. 2023;10:e2300148.

Reviewer 2#
Title: "A high-quality assembly reveals causal gene for unique plumage phenotype of Liancheng ducks"

- English is suboptimal, needs to be improved
- a major comment is that I think that you should place the Methods section before the Results section: your article is methods intensive (de novo genome assembly, resequencing, RNA-seq, GWAS etc.), therefore it is very difficult to fully understand and interpret your work without reading first the Materials and Methods. Please change the structure of your article.

Response: Thank you for your comments. We agree with you very much, and have put the Materials and Methods section behind the Background and the Analyses in front, which makes the manuscript more coherent. At the same time, we have invited professors in the field to revise and polish the paper, hoping that the manuscript can meet the requirements for publication in Gigascience. Thank you again for your suggestions.

Background

L40: phenotype instead of topic
Response: We have replaced “phenotype” with “topic” in the manuscript.

L53: in which year was N50 = 76.3 Mb reached?
Response: We have added the year in which the duck genome was published in this part, and you also can find this part in the Supplement Table 6.

L54: why do you think you need more assembly quality in ducks? Please justify
Response: Thank you for your comments. We believe that a high-quality reference genome is particularly important for the genetic analysis of complex traits, especially the lack of genome information of Liancheng ducks. We believe that our study is valuable for the study of feather color in birds. “The initial draft of the duck assembly was first reported in 2013 [6]. Subsequent enhancements to the Pekin (PK) duck genomes have led to a scaffold N50 length of up to 76.3 Mb by 2020, which now is widely used for duck genome analysis [7]. Compared to the high quality and variety of genome assembly in other birds, especially chickens, further improvements in duck assembly quality are still needed. Previous research related on population genetics of ducks has primarily focused on meat quality [8], adipose deposition [9], and muscle

weight [10], but genetic mechanisms of plumage color remain largely unexplored. Meanwhile, no reference genome of the famous LC duck has been published. Therefore, an upgraded LC duck genome is crucial to provide foundational data for future studies on this species. The accuracy of gene localization greatly depends on the quality of the genome assembly [9]”. (Line 62-71 in the revised manuscript).

L54: Previous research in which area? Animal breeding and genetics? Population genetics? Other?
Response: We have already supplemented this section. “Previous research related on animal breeding and genetics of ducks has primarily focused on meat quality [8], adipose deposition [9], and muscle weight [10], but genetic mechanisms of plumage color remain largely unexplored. Meanwhile, no reference genome of the famous LC duck has been published. Therefore, an upgraded LC duck genome is crucial to provide foundational data for future studies on this species.” (Line 66-70 in the revised manuscript).

L58: maybe it's improved quality of the duck genome?
Response: We have revised this sentence. Thanks!

L59: white duck down feathers? Maybe it's duck white down (?) feathers? What do you mean by "down feathers"?
Response: We have revised and improved this part. Thanks.

L61: what is HiFi sequencing? Are you referring to long-read sequencing? Be specific
Response: We have revised and improved this part. “To date, long-read sequencing technology (or third-generation sequencing) can produce reads longer than 10 kb, spanning highly repetitive genomic regions and bridging previously intractable assembly gaps to improve overall continuity. As an alternative to relying on short-read data polishing, PacBio introduced high-fidelity reads (HiFi reads) can provide more accurate, continuous and complete genetic information, making them a key enabling technology for research [13]” (Line 76-81 in the revised manuscript).

L69: by appearance you mean plumage? Please avoid generic terms
Response: We have revised this part. Thanks.

L72: ... famous duck breed characterized by its ...
Response: We have revised this sentence. Thanks.

L72: replace replace but with and: white feathers and black beak and feet (is feet the right word in birds? Please check)
Response: We have revised this word. The description of the duck appearance phenotype has been modified to make it more professional. “38 white-feathered ducks with yellow beaks and webbing (WY), 42 white-feathered ducks with black beaks and webbing (WB), 67 gray-feathered ducks with black beaks and webbing (GF), and 41 black-feathered ducks with black beaks and webbing (BF) underwent whole-genome resequencing (Table S1)” (Line 112-115 in the revised manuscript).
References:
Han D, Liu H, Ren L, Hu J, Yang Q. From the Analysis of Anatomy and Locomotor Function of Biological Foot Systems to the Design of Bionic Foot: An Example of the Webbed Foot of the Mallard. Biomimetics. 2023;8(8):592.
Jaekel M, Wake DB. Developmental processes underlying the evolution of a derived foot morphology in salamanders. Proc Natl Acad Sci U S A. 2007;104(51):20437-20442.

L73: Liancheng is still a breed, not a distinct species
Response: We have revised this description.

L84: This study, right?
Response: We have revised this word.

L86: this research identified
Response: We have revised this description. Thanks.

Data description

L95-96: it is not clear whether you generated a de novo assembly of the duck genome in this study, or if you aligned your reads from resequencing to an existing build of the genome
Response: Thank you for your comments. To understand the genetic basis of unique plumage phenotype of Liancheng ducks, we utilized a high-quality de novo assembly of Liancheng duck genome (GCA_039998735.1). Additionally, we performed whole-genome resequencing of 366 ducks and aligned the resequencing data to the Liancheng duck genome assembly (GCA_039998735.1) generated in this study for GWAS analysis. Through transcriptome sequencing analysis across black-, and white-feathered ducks and nine duck tissues, we identified PMEL gene associated with plumage color in Liancheng ducks and elucidated the genetic mechanisms underlying the formation of duck plumage color.

L97: which are these multiple breeds?
Response: We have revised this description.

L97: one single gene? Or genes?
Response: We have revised this description. Through transcriptome sequencing analysis across black-, and white-feathered ducks and nine duck tissues, we identified PMEL gene associated with plumage color in Liancheng ducks and elucidated the genetic mechanisms underlying the formation of duck plumage color. Thanks!

Materials and Methods

L411: why these 5 timepoints? What was the rationale behind it?
Response: The 12d, 15d and 20d of duck embryo development were nodes with obvious changes in feather color of Liancheng ducks. Related studies on feather follicle development of ducks also focus on these stages (Chen et al., 2017). We believed that these three stages represent the three most obvious color phenotypes of Liancheng duck feathers during embryonic stage. The time of duck shell breaking is the 28th day of embryo development, and the feather color phenotype can be clearly identified after 1 week, so we selected these five nodes for phenotype collection and RNA-seq analysis.
References:
Chen X, Ge K, Wang M, Zhang C, Geng Z. Integrative analysis of the Pekin duck (Anas anas) MicroRNAome during feather follicle development. BMC Dev Biol. 2017;17(1):12.

L408-415: it is not clear how many samples you used for RNA-seq (and proteomics?): 227 ducks (Pekin + grey and black feathered ducks) times 5 timepoints times 3 replicates + heart, fat, muscle, brain, spleen, lung, liver, kidney, and skin tissues: please provide the total number of samples for transcriptomics, and the breakdown by timepoint/tissue
Response: Thank you for advice. We have described the sample size for RNA sequencing in detail and presented it in Supplement Table 11. A total of 37 samples were used for RNA-seq (Table S2) (Line 124-125 in the revised manuscript).

L428: It is not clear what you did with BUSCO: this is a tool usually employed to assess the quality of genome assemblies. Please add details of what you did, why and how you did it
Response: Thank you for comments. The quality of the genome assembly is assessed based on the completeness metrics from BUSCO. Generally, a higher proportion of complete BUSCOs indicates better genome quality. A thorough evaluation of the assembly quality post-assembly, using BUSCO, revealed 96.7% genome completeness (Table S7). Run BUSCO using the command line with the following basic command format: busco -i -o -l --mode genome.

L430: "The structure prediction of gene": do you mean the prediction of the structure of genes?
Response: Thank you for comments. We have rephased the sentence to be clear for the Prediction of protein-coding genes (Line 141-142 in the revised manuscript).

L431: delete "as evidence files"
Response: We have deleted this part. Thanks.

L434: please explain (also in the text) what Augustus is
Response: We have revised and improved this part. Augustus is a program that predicts genes in eukaryotic genomic sequences. In this study, "Augustus (http://bioinf.uni-greifswald.de/augustus/) that predicts genes in genomic sequences, and then de novo annotation was performed through Augustus." (Line 145-147 in the revised manuscript).

L435: also here, explain what Maker and De novo are and/or refer to
Response: We used RNA-seq data for evidence-based annotation through Maker (Version 2.31.10), which is a powerful open source genome annotation tool (Line 149-150 in the revised manuscript). De novo genome assembly refers to a method of piecing together a complete sequence of a new genome, rather than comparing it based on a known genome." (Line 147-149 in the revised manuscript). We have already supplemented and explained these parts in the text. Thanks!

L436: repeated sequwnces
Response: We have revised this word.

L437: encoded protein longer than
Response: We have revised this part.

L437: at least one count from gene expression / RNAseq
Response: We have revised the sentence. Thanks.

L441: please add references to eggNOG and BLAST
Response: We have added references to this part. Thanks.

L447: average read length
Response: We have revised these words. At the same time, we have polished the language and modification of the full text, hoping to meet the requirements of the magazine publication.

L448: what is the original coverage? You mean the target coverage "a priori"?
Response: We have revised and improved this part. All libraries were sequenced on an Illumina HiSeq X-Ten platform, achieving an average raw read sequence coverage of 5×. Based on Liancheng duck reference genome (IASCAAS_LianchengWhiteDuck, GCA_039998735.1), this ensures the accuracy of variant calling and genotyping [6].

L448: it is usually known as "variant calling"
Response: We have revised these words. Thanks.

L449: which requirements?
Response: We have revised these parts. A detailed description of this section can be found in the following section (Line 175-193 in the revised manuscript).

L450: you threw away the entire reads containing the adapter sequence? Or did you remove just the adapter using tools like Cutadapt? How did you identify and eliminate these reads? More details are needed
Response: We have revised this part. "Based on Liancheng duck reference genome (IASCAAS_LianchengWhiteDuck, GCA_039998735.1), this ensures the accuracy of variant calling and genotyping [6]. Following the elimination of read pairs containing adapter sequences, a quality assessment of the raw reads was performed utilizing TRIMMOMATIC (RRID:SCR_011848) (version 0.36) and Cutadapt [24,25]. Subsequently, the high-quality reads were aligned to the Liancheng duck reference genome (IASCAAS_LianchengWhiteDuck, GCA_039998735.1) using the Burrow–Wheeler Aligner (BWA-aln) with parameter 'bwa aln genome.fa sample.fastq > sample.sai, bwa sampe genome.fa sample.sai sample.fastq > sample.sam' [26].

L453: please report the default settings, this will aid the reader
Response: Thank you for your advice. The answer is shown in the last answer.

L453-454: did you perform variant calling with BWA? How?
Response: Thank you for comments. The Genome Analysis Toolkit (GATK) (McKenna et al., 2010) was used to identify genetic variants from the sequencing data in this study.

L455: I don't understand the filter on max allele frequency: SNPs are biallelic, if the frequency of one allele is 0.99 then MAF = 0.01, so this criterion is useless and redundant
Response: Thank you for your revisions. We have supplemented and revised the description of this part. "SNPs underwent filtration based on the following criteria: (i) SNPs were required to demonstrate a minor allele frequency (MAF) >0.05; (ii) the maximum missing rate was set at <0.7; and (iii) SNPs were restricted to possessing only two alleles" (Line 169-172 in the revised manuscript).

L456: max missing rate per SNP, ok: what about sample missing rate?
Response: Thank you for your valuable comments on this paper, so that the expression and logic of the manuscript are more rigorous. In this study, we completed SNP filtering and missing rate analysis, but did not carry out sample missing rate analysis. We have the following reasons: Firstly, we found the PMEL gene controlling the white feather phenotype of Liancheng duck through GWAS analysis after SNP filtering, and found two completely linked SNP variations, indicating that the analysis method is effective in this study. Secondly, we found that some papers did not carry out sample missing rate analysis in GWAS genome-wide association (Zhu et al., 2016; Zhou et al., 2018). Finally, we believe that sample missing rate detection is very meaningful, but the sample size in this study is small. In the future, we hope to increase the sample size and conduct sample missing rate detection, hoping to further verify our experimental results through a large number of samples.
References:
Zhu, Z., Zhang, F., Hu, H. et al. Integration of summary data from GWAS and eQTL studies predicts complex trait gene targets. Nat Genet 48, 481–487 (2016).
Zhou, Z., Li, M., Cheng, H. et al. An intercross population study reveals genes associated with body size and plumage color in ducks. Nat Commun 9, 2648 (2018).
