# Supplementary material for: A high-quality assembly revealing the PMEL gene for the unique plumage phenotype in Liancheng ducks
Source: Gigascience. 2025 Jan 13;14:giae114. doi: 10.1093/gigascience/giae114 (PMC11727711; doi:10.1093/gigascience/giae114)
Supplement: giae114_Response_to_Reviewer_Comments_Revision_1 [file giae114_response_to_reviewer_comments_revision_1.pdf]

Author's Response To Reviewer Comments

Dear Reviewer and editors,

Thank you for handing our manuscript. We thank you for their critical comments and valuable suggestions, which have significantly improved our manuscript. In the revised version, the language has been carefully revised based on your comments. Meanwhile, the sentences and grammar were checked by all authors and language editing company, hoping to meet the publication requirements of Gigascience magazine.

Below, we offer a point-by-point response and indicate the corresponding modified sections in the revised manuscript.

We hope that our current manuscript addresses the reviewer's concerns.

Sincerely,

All authors

Reviewer 2#
Reviewer #2: - English still below what is acceptable for publication
Response: Thank you for your comments. We have invited professors in the field to revise and polish the paper. Meanwhile, the sentences and grammar were rechecked by a language editing company, hoping to meet the publication requirements of Gigascience magazine. Thank you again for your suggestions.

Abstract

L17: "Plumage color is a notable characteristic and has widely studied": has BEEN widely studied
Response: Thank you for your reminder. We have revised and polished this sentence.

L18: it is not clear what webbing is: the webbing of the duck's feet/paws? Please explain
Response: Thank you for your comments. The Liancheng (LC) duck possesses a distinctive phenotype marked by white feathers, black beak, and black webbed feet. It is recognized for its significant melanin deposition in the beak and webbed feet, primarily due to the involvement of eumelanin as the main pigment. We mainly focus on the melanin deposition of Liancheng duck beaks and webbed (the skin on the feet), revised the full text, similar papers use the same expression.
References:
Johansson, L., Norberg, R. Delta-wing function of webbed feet gives hydrodynamic lift for swimming propulsion in birds. Nature 424, 65–68 (2003).
S. B. A. Kashem, M. Tabassum and M. Chai, "A novel design of an amphibious robot having webbed feet as duck," 2017 International Conference on Computer and Drone Applications (IConDA), Kuching, Malaysia, 2017, pp. 17-21.

Background

L65: genome assemblies (plural)
Response: Thank you for your comments. We have revised this sentence.

L66: delete "related"
Response: We have revised this sentence.

L66: replace of with in: in ducks
Response: We have revised this sentence.

L67: adipose TISSUE deposition (or fat deposition)
Response: We have revised this sentence.

L79-80: "PacBio introduced high-fidelity reads (HiFi reads) WHICH can provide more accurate, continuous and complete genetic information, making THIS a key enabling technology for research"
Response: Thank you for your comments. We have revised this sentence.

M&M

L138-139: quality of which genome assembly? Please be specific and clear
Response: Thank you for your reminding. We have revised this sentence.

L153: against the databases GO, KEGG etc. (delete of)
Response: We have revised this sentence.

L170: delete "ere required to demonstrate a" SNPs with minor allele ...
Response: We have revised this sentence.

L171: I guess this is missing rate per SNP: please specify
Response: Thank you for your comments. We have revised this sentence.
