# Supplementary material for: A high-quality assembly revealing the PMEL gene for the unique plumage phenotype in Liancheng ducks
Source: Gigascience. 2025 Jan 13;14:giae114. doi: 10.1093/gigascience/giae114 (PMC11727711; doi:10.1093/gigascience/giae114)
Supplement: giae114_Reviewer_1_Report_Original_Submission — Gene Ng -- 7/25/2024 [file giae114_reviewer_1_report_original_submission.pdf]

Reviewer Report

Title: A high-quality assembly revealing PMEL gene for unique plumage phenotype of Liancheng ducks

Version: Original Submission **Date:** 7/25/2024

Reviewer name: Gene Ng

Reviewer Comments to Author:

The manuscript "A high-quality assembly reveals causal gene for unique plumage phenotype of Liancheng ducks" focuses on uncovering the genetic basis of the distinctive white plumage seen in Liancheng ducks. The study aims to understand the genetic determinants of plumage color in Liancheng ducks, a breed known for its unique combination of white feathers with black beaks and feet. The primary focus is on identifying genetic variants that contribute to this phenotype.

The authors constructed a de novo genome assembly for the Liancheng duck using PacBio HiFi sequencing and Hi-C data to achieve this. The assembly achieved significant quality, with a genome size of approximately 1.29 Gb. Additionally, a genome-wide association study (GWAS) was conducted using a mixed linear model to identify significant single nucleotide polymorphisms (SNPs) associated with the white plumage phenotype. The analysis involved 366 individuals from an F2 population derived from crossbreeding Liancheng and Pekin ducks.

The GWAS identified a 0.8 Mb candidate region on Chromosome 33 containing the PMEL gene, a key determinant of plumage color. This region includes two closely linked SNPs (Chr33:5,303,994A>G; 5,303,997A>G) that may alter PMEL gene transcription. Functional assays were performed to validate the impact of these SNPs on gene expression, suggesting that they significantly influence the white plumage phenotype.

The study presents the first high-quality genome assembly of the Liancheng duck and identifies novel genetic variants in the PMEL gene associated with white plumage. These findings provide valuable insights into the genetic mechanisms underlying plumage coloration and offer promising practical implications for selective breeding and conservation efforts.

Specific comments:

1) Discuss how your findings compare with the genetic and molecular basis of feather diversity as highlighted in these review papers:

Terrill RS, Shultz AJ. Feather function and the evolution of birds. Biol Rev Camb Philos Soc. 2023 Apr;98(2):540-566. doi: 10.1111/brv.12918. Epub 2022 Nov 24.

Ng CS, Li WH. Genetic and Molecular Basis of Feather Diversity in Birds. Genome Biol Evol. 2018 Oct 1;10(10):2572-2586. doi: 10.1093/gbe/evy180.

Boer EF, Van Hollebeke HF, Shapiro MD. Genomic determinants of epidermal appendage patterning and structure in domestic birds. Dev Biol. 2017 Sep 15;429(2):409-419.

Chen CF, Foley J, Tang PC, Li A, Jiang TX, Wu P, Widelitz RB, Chuong CM. Development, regeneration, and evolution of feathers. Annu Rev Anim Biosci. 2015;3:169-95. doi: 10.1146/annurev-animal-022513-114127.

2) Integrate these studies to provide a broader context for the role of PMEL in different bird species and

its implications for plumage color:
Yuan Z, Zhang X, Pang Y, Qi Y. Association analysis of PMEL gene expression and single nucleotide polymorphism with plumage color in quail. Anim Biotechnol. 2023 Dec;34(9):5001-5010. doi: 10.1080/10495398.2023.2221697.
Ishishita S, Takahashi M, Yamaguchi K, Kinoshita K, Nakano M, Nunome M, Kitahara S, Tatsumoto S, Go Y, Shigenobu S, Matsuda Y. Nonsense mutation in PMEL is associated with yellowish plumage colour phenotype in Japanese quail. Sci Rep. 2018 Nov 13;8(1):16732. doi: 10.1038/s41598-018-34827-4.
Liu X, Zhou R, Peng Y, Zhang C, Li L, Lu C, Li X. Feather follicles transcriptome profiles in Bashang long-tailed chickens with different plumage colors. Genes Genomics. 2019 Nov;41(11):1357-1367. doi: 10.1007/s13258-018-0740-y.
Zheng X, Zhang B, Zhang Y, Zhong H, Nie R, Li J, Zhang H, Wu C. Transcriptome analysis of feather follicles reveals candidate genes and pathways associated with pheomelanin pigmentation in chickens. Sci Rep. 2020 Jul 21;10(1):12088. doi: 10.1038/s41598-020-68931-1.
Hua G, Chen J, Wang J, Li J, Deng X. Genetic basis of chicken plumage color in artificial population of complex epistasis. Anim Genet. 2021 Oct;52(5):656-666. doi: 10.1111/age.13094.
Weng Z, Xu Y, Li W, Chen J, Zhong M, Zhong F, Du B, Zhang B, Huang X. Genomic variations and signatures of selection in Wuhua yellow chicken. PLoS One. 2020 Oct 23;15(10):e0241137. doi: 10.1371/journal.pone.0241137.
Heo S, Cho S, Dinh PTN, Park J, Jin DH, Cha J, Kim YK, Koh YJ, Lee SH, Lee JH. A genome-wide association study for eumelanin pigmentation in chicken plumage using a computer vision approach. Anim Genet. 2023 Jun;54(3):355-362. doi: 10.1111/age.13303.
Abolins-Abols M, Kornobis E, Ribeca P, Wakamatsu K, Peterson MP, Ketterson ED, MilÃ¡ B. Differential gene regulation underlies variation in melanic plumage coloration in the dark-eyed junco (Junco hyemalis). Mol Ecol. 2018 Nov;27(22):4501-4515. doi: 10.1111/mec.14878.
Yuan H, Zhang X, Zhang Q, Wang Y, Wang S, Li Y, Zhang Y, Jing J, Qiu J, Wang Z, Leng L. Comparative transcriptome profiles of Lindian chicken eyelids identify melanin genes controlling eyelid pigmentation. Br Poult Sci. 2019 Feb;60(1):15-22. doi: 10.1080/00071668.2018.1544414.
3) Discuss the potential role of MITF in plumage coloration, referencing these studies to highlight its regulatory mechanisms and effects:
Goding CR, Arnheiter H. MITF-the first 25 years. Genes Dev. 2019 Aug 1;33(15-16):983-1007. doi: 10.1101/gad.324657.119. Epub 2019 May 23.
Yuan B, Qi Y, Zhang X, Hu J, Fan Y, Ji X. The relationship of MITF gene expression and promoter methylation with plumage colour in quail. Br Poult Sci. 2024 Jun;65(3):259-264. doi: 10.1080/00071668.2024.2326962.
Pan R, Hua T, Guo Q, Bai H, Jiang Y, Wang Z, Bi Y, Chen G, Wu X, Chang G. Identification of SNPs in MITF associated with beak color of duck. Front Genet. 2023 Aug 21;14:1161396. doi: 10.3389/fgene.2023.1161396.
Lin R, Lin W, Zhou S, Chen Q, Pan J, Miao Y, Zhang M, Huang Z, Xiao T. Integrated Analysis of mRNA Expression, CpG Island Methylation, and Polymorphisms in the MITF Gene in Ducks (Anas platyrhynchos). Biomed Res Int. 2019 Sep 23;2019:8512467. doi: 10.1155/2019/8512467.
Guo Q, Jiang Y, Wang Z, Bi Y, Chen G, Bai H, Chang G. Genome-Wide Analysis Identifies Candidate Genes Encoding Feather Color in Ducks. Genes (Basel). 2022 Jul 14;13(7):1249. doi: 10.3390/genes13071249.

Ren S, Lyu G, Irwin DM, Liu X, Feng C, Luo R, Zhang J, Sun Y, Shang S, Zhang S, Wang Z. Pooled Sequencing Analysis of Geese (Anser cygnoides) Reveals Genomic Variations Associated With Feather Color. Front Genet. 2021 Jun 18;12:650013. doi: 10.3389/fgene.2021.650013.
Lin R, Zhao F, Xiong T, Lai L, Li H, Lin W, Xiao T, Lin W. Genetic mapping identifies SNP mutations in MITF-M promoter associated with melanin formation in Putian black duck. Poult Sci. 2024 Jan;103(1):103191. doi: 10.1016/j.psj.2023.103191.
Sultana H, Seo D, Choi NR, Bhuiyan MSA, Lee SH, Heo KN, Lee JH. Identification of polymorphisms in MITF and DCT genes and their associations with plumage colors in Asian duck breeds. Asian-Australas J Anim Sci. 2018 Feb;31(2):180-188. doi: 10.5713/ajas.17.0298.
Zhang X, Zhu T, Wang L, Lv X, Yang W, Qu C, Li H, Wang H, Ning Z, Qu L. Genome-Wide Association Study Reveals the Genetic Basis of Duck Plumage Colors. Genes (Basel). 2023 Mar 31;14(4):856. doi: 10.3390/genes14040856.
Li S, Wang C, Yu W, Zhao S, Gong Y. Identification of genes related to white and black plumage formation by RNA-Seq from white and black feather bulbs in ducks. PLoS One. 2012;7(5):e36592. doi: 10.1371/journal.pone.0036592.
Wang L, Guo J, Xi Y, Ma S, Li Y, He H, Wang J, Han C, Bai L, Mustafa A, Liu H, Li L. Understanding the Genetic Domestication History of the Jianchang Duck by Genotyping and Sequencing of Genomic Genes Under Selection. G3 (Bethesda). 2020 May 4;10(5):1469-1476. doi: 10.1534/g3.119.400893.
4) Compare your genome assembly results with these recently published assemblies to provide a comprehensive understanding of the advancements and differences:
Xu MM, Gu LH, Lv WY, Duan SC, Li LW, Du Y, Lu LZ, Zeng T, Hou ZC, Ma ZS, Chen W, Adeola AC, Han JL, Xu TS, Dong Y, Zhang YP, Peng MS. Chromosome-level genome assembly of the Muscovy duck provides insight into fatty liver susceptibility. Genomics. 2022 Nov;114(6):110518. doi: 10.1016/j.ygeno.2022.110518.
Ng CS, Lai CK, Ke HM, Lee HH, Chen CF, Tang PC, Cheng HC, Lu MJ, Li WH, Tsai IJ. Genome Assembly and Evolutionary Analysis of the Mandarin Duck Aix galericulata Reveal Strong Genome Conservation among Ducks. Genome Biol Evol. 2022 May 31;14(6):evac083. doi: 10.1093/gbe/evac083.
Che T, Li J, Li X, Wang Z, Zhang X, Yang W, Liu T, Wang Y, Wang K, Gao T, Shen G, Qiu W, Li Z, Zhang W.Haplotype-resolved assembly of the mule duck genome using high-fidelity sequencing technology. PLoS One. 2024 Jul 1;19(7):e0305914. doi: 10.1371/journal.pone.0305914.
Jiang F, Jiang Y, Wang W, Xiao C, Lin R, Xie T, Sung WK, Li S, JakovliÄ‡ I, Chen J, Du X. A chromosome-level genome assembly of Cairina moschata and comparative genomic analyses. BMC Genomics. 2021 Jul 30;22(1):581. doi: 10.1186/s12864-021-07897-4.
Mueller RC, EllstrÃ¶m P, Howe K, Uliano-Silva M, Kuo RI, Miedzinska K, Warr A, Fedrigo O, Haase B, Mountcastle J, Chow W, Torrance J, Wood JMD, JÃ¤rhult JD, Naguib MM, Olsen B, Jarvis ED, Smith J, EÃ¶ry L, Kraus RHS. A high-quality genome and comparison of short- versus long-read transcriptome of the palaearctic duck Aythya fuligula (tufted duck). Gigascience. 2021 Dec 20;10(12):giab081. doi: 10.1093/gigascience/giab081.
Hu J, Song L, Ning M, Niu X, Han M, Gao C, Feng X, Cai H, Li T, Li F, Li H, Gong D, Song W, Liu L, Pu J, Liu J, Smith J, Sun H, Huang Y. A new chromosome-scale duck genome shows a major histocompatibility complex with several expanded multigene families. BMC Biol. 2024 Feb 5;22(1):31. doi: 10.1186/s12915-024-01817-0.

Lavretsky P, HernÃ¡ndez F, Swale T, Mohl JE. Chromosomal-level reference genome of a wild North American mallard (Anas platyrhynchos). G3 (Bethesda). 2023 Sep 30;13(10):jkad171. doi: 10.1093/g3journal/jkad171.
Wang K, Hua G, Li J, Yang Y, Zhang C, Yang L, Hu X, Scheben A, Wu Y, Gong P, Zhang S, Fan Y, Zeng T, Lu L, Gong Y, Jiang R, Sun G, Tian Y, Kang X, Hu H, Li W. Duck pan-genome reveals two transposon insertions caused bodyweight enlarging and white plumage phenotype formation during evolution. Imeta. 2023 Dec 17;3(1):e154. doi: 10.1002/imt2.154.
Chang G, Yuan X, Guo Q, Bai H, Cao X, Liu M, Wang Z, Li B, Wang S, Jiang Y, Wang Z, Zhang Y, Xu Q, Song Q, Pan R, Qiu L, Gu T, Wu X, Bi Y, Cao Z, Zhang Y, Chen Y, Li H, Liu J, Dai W, Chen G. The First Crested Duck Genome Reveals Clues to Genetic Compensation and Crest Cushion Formation. Genomics Proteomics Bioinformatics. 2023 Jun;21(3):483-500. doi: 10.1016/j.gpb.2023.08.002.
5) Replace "hair follicle" with "feather follicle" throughout the manuscript. Hair is a mammalian-specific skin appendage; birds have feathers, not hair.
6) Specify which part of the feather follicle was used for RNA-seq extraction.
7) Show the statistical test used along with the p-value in parentheses. For example, (ANOVA, $p < 0.05$).
8) Figure 4B: Clarify what the error bars represent. Figure 4C: Explain the asterisk (*) and indicate which statistical test was applied. Figure 6: Specify what the error bars represent and the statistical test used.
9) Clearly state the threshold after Bonferroni correction and justify choosing it over other methods such as FDR.

Methods

Are the methods appropriate to the aims of the study, are they well described, and are necessary controls included? Choose an item.

Conclusions

Are the conclusions adequately supported by the data shown? Choose an item.

Reporting Standards

Does the manuscript adhere to the journal’s guidelines on minimum standards of reporting? Choose an item.

Choose an item.

Statistics

Are you able to assess all statistics in the manuscript, including the appropriateness of statistical tests used? Choose an item.

Quality of Written English

Please indicate the quality of language in the manuscript: Choose an item.

Declaration of Competing Interests

Please complete a declaration of competing interests, considering the following questions:

- Have you in the past five years received reimbursements, fees, funding, or salary from an organisation that may in any way gain or lose financially from the publication of this manuscript, either now or in the future?
- Do you hold any stocks or shares in an organisation that may in any way gain or lose financially from the publication of this manuscript, either now or in the future?
- Do you hold or are you currently applying for any patents relating to the content of the manuscript?
- Have you received reimbursements, fees, funding, or salary from an organization that holds or has applied for patents relating to the content of the manuscript?
- Do you have any other financial competing interests?
- Do you have any non-financial competing interests in relation to this paper?

If you can answer no to all of the above, write 'I declare that I have no competing interests' below. If your reply is yes to any, please give details below.

I declare that I have no competing interests

I agree to the open peer review policy of the journal. I understand that my name will be included on my report to the authors and, if the manuscript is accepted for publication, my named report including any attachments I upload will be posted on the website along with the authors' responses. I agree for my report to be made available under an Open Access Creative Commons CC-BY license (http://creativecommons.org/licenses/by/4.0/). I understand that any comments which I do not wish to be included in my named report can be included as confidential comments to the editors, which will not be published.

Choose an item.

To further support our reviewers, we have joined with Publons, where you can gain additional credit to further highlight your hard work (see: https://publons.com/journal/530/gigascience). On publication of this paper, your review will be automatically added to Publons, you can then choose whether or not to claim your Publons credit. I understand this statement.

Yes Choose an item.
