# Supplementary material for: A high-quality assembly revealing the PMEL gene for the unique plumage phenotype in Liancheng ducks
Source: Gigascience. 2025 Jan 13;14:giae114. doi: 10.1093/gigascience/giae114 (PMC11727711; doi:10.1093/gigascience/giae114)
Supplement: giae114_Reviewer_2_Report_Original_Submission — Filippo Biscarini -- 9/15/2024 [file giae114_reviewer_2_report_original_submission.pdf]

Reviewer Report

Title: A high-quality assembly revealing PMEL gene for unique plumage phenotype of Liancheng ducks

Version: Original Submission **Date:** 9/15/2024

Reviewer name: Filippo Biscarini

Reviewer Comments to Author:

Title : "A high-quality assembly reveals causal gene for unique plumage phenotype of Liancheng ducks"
- English is suboptimal, needs to be improved
- a major comment is that I think that you should place the Methods section before the Results section: your article is methods intensive (de novo genome assembly, resequencing, RNA-seq, GWAS etc.), therefore it is very difficult to fully understand and interpret your work without reading first the Materials and Methods. Please change the structure of your article.

Background

L40: phenotype instead of topic
L53: in which year was N50 = 76.3 Mb reached?
L54: why do you think you need more assembly quality in ducks? Please justify
L54: Previous research in which area? Animal breeding and genetics? Population genetics? Other?
L58: maybe it's improved quality of the duck genome?
L59: white duck down feathers? Maybe it's duck white down (?) feathers? What do you mean by "down feathers"?
L61: whhat is HiFi sequencing? Are you referring to long-read sequencing? Be specific
L69: by appearance you mean plumage? Please avoid generic terms
L72: ... famous duck breed characterized by its ...
L72: replace replace but with and: white feathers and black beak and feet (is feet the right word in birds? Please check)
L73: Liancheng is still a breed, not a distinct species
L84: This study, right?
L86: this research identified

Data description

L95-96: it is not clear whether you generated a de novo assembly of the duck genome in this study, or if you aligned your reads from resequencing to an existing build of the genome
L97: which are these multiple breeds?
L97: one single gene? Or genes?
[up to L101]

Materials and Methods

L411: why these 5 timepoints? What was the rationale behind it?

L408-415: it is not clear how many samples you used for RNA-seq (and proteomics?): 227 ducks (Pekin + grey and black feathered ducks) times 5 timepoints times 3 replicates + heart, fat, muscle, brain, spleen, lung, liver, kidney, and skin tissues: please provide the total number of samples for transcriptomics, and the breakdown by timepoint/tissue
L428: It is not clear what you did with BUSCO: this is a tool usually employed to assess the quality of genome assemblies. Please add details of what you did, why and how you did it
L430: "The structure prediction of gene": do you mean the prediction of the structure of genes?
L431: delete "as evidence files"
L434: please explain (also in the text) what Augustus is
L435: also here, explain what Maker and De novo are and/or refer to
L436: repeated sequwnces
L437: encoded protein longer than
L437: at least one count from gene expression / RNAseq
L441: please add references to eggNOG and BLAST
L447: average read length
L448: what is the original coverage? You mean the target coverage "a priori"?
L448: it is usually known as "variant calling"
L449: which requirements?
L450: you threw away the entire reads containing the adapter sequence? Or did you remove just the adapter using tools like Cutadapt? How did you identify and eliminate these reads? More details are needed
L453: please report the default settings, this will aid the reader
L453-454: did you perform variant calling with BWA? How?
L455: I don't understand the filter on max allele frequency: SNPs are biallelic, if the frequency of one allele is 0.99 then MAF = 0.01, so this criterion is useless and redundant
L456: max missing rate per SNP, ok: what about sample missing rate?
[up to L459]

Methods

Are the methods appropriate to the aims of the study, are they well described, and are necessary controls included? Choose an item.

Conclusions

Are the conclusions adequately supported by the data shown? Choose an item.

Reporting Standards

Does the manuscript adhere to the journal's guidelines on minimum standards of reporting? Choose an item.

Choose an item.

Statistics

Are you able to assess all statistics in the manuscript, including the appropriateness of statistical tests used? Choose an item.

Quality of Written English

Please indicate the quality of language in the manuscript: Choose an item.

Declaration of Competing Interests

Please complete a declaration of competing interests, considering the following questions:

- Have you in the past five years received reimbursements, fees, funding, or salary from an organisation that may in any way gain or lose financially from the publication of this manuscript, either now or in the future?
- Do you hold any stocks or shares in an organisation that may in any way gain or lose financially from the publication of this manuscript, either now or in the future?
- Do you hold or are you currently applying for any patents relating to the content of the manuscript?
- Have you received reimbursements, fees, funding, or salary from an organization that holds or has applied for patents relating to the content of the manuscript?
- Do you have any other financial competing interests?
- Do you have any non-financial competing interests in relation to this paper?

If you can answer no to all of the above, write 'I declare that I have no competing interests' below. If your reply is yes to any, please give details below.

I declare that I have no competing interests

I agree to the open peer review policy of the journal. I understand that my name will be included on my report to the authors and, if the manuscript is accepted for publication, my named report including any attachments I upload will be posted on the website along with the authors' responses. I agree for my report to be made available under an Open Access Creative Commons CC-BY license (http://creativecommons.org/licenses/by/4.0/). I understand that any comments which I do not wish to be included in my named report can be included as confidential comments to the editors, which will not be published.

Choose an item.

To further support our reviewers, we have joined with Publons, where you can gain additional credit to further highlight your hard work (see: https://publons.com/journal/530/gigascience). On publication of this paper, your review will be automatically added to Publons, you can then choose whether or not to claim your Publons credit. I understand this statement.

Yes Choose an item.
